# The Influence of the Number and Position of the Methoxy Group on the Physicochemical Properties and Possible Applications of Rhodanine-3-Acetic Acid Derivatives

**DOI:** 10.3390/ma19204287

**Published:** 2026-10-11

**Authors:** Agata Szlapa-Kula, Anna Biernasiuk, Łukasz Kaźmierski, Dariusz Kuśmierz, Justyna Płonka-Czerw, Malgorzata Latocha, Martyna Zegarska, Slawomir Kula, Przemysław Krawczyk

**Affiliations:** 1 Institute of Chemistry, Faculty of Science and Technology, University of Silesia in Katowice, Szkolna 9 St., 40-007 Katowice, Poland; slawomir.kula@us.edu.pl; 2 Department of Pharmaceutical Microbiology, Faculty of Pharmacy, Medical University of Lublin, Chodźki 1, 20-093 Lublin, Poland; anna.biernasiuk@umlub.pl; 3 Department of Urology and Andrology, Faculty of Medicine, Collegium Medicum, Nicolaus Copernicus University, Karłowicza 24, 85-092 Bydgoszcz, Poland; kazmierski.lkk@gmail.com; 4 Department of Cell Biology, Faculty of Pharmaceutical Sciences in Sosnowiec, Medical University of Silesia, Jednosci 9, 41-200 Sosnowiec, Poland; dkusmierz@sum.edu.pl (D.K.); jplonka@sum.edu.pl (J.P.-C.); mlatocha@sum.edu.pl (M.L.); 5 Antoni Jurasz Univeristy Hospital No. 1 in Bydgoszcz, Marii Skłodowskiej-Curie 9, 85-094 Bydgoszcz, Poland; zegarskamartyna@gmail.com; 6 Department of Physical Chemistry, Faculty of Pharmacy, Collegium Medicum, Nicolaus Copernicus University, Kurpińskiego 5, 85-950 Bydgoszcz, Poland; przemekk@cm.umk.pl

**Keywords:** rhodanine-3-acetic acid derivatives, fluorescent dyes, bioimaging, antibacterial and antifungal activity, anticancer activity

## Abstract

In this study, we investigated the effect of the number and position of the methoxy group substitution on selected physicochemical and biological properties of rhodanine-3-acetic acid derivatives. We conducted our studies on a group of eight compounds (**C-1**–**C-8**) obtained by Knoevenagel condensation, with yields ranging from 49% to 99%. All synthesized compounds were thermally stable above 240 °C. Luminescence studies revealed the ICT nature of the absorption and emission spectra. Derivative C-4 exhibited the greatest blue shift, while compounds **C-7** and **C-8** exhibited the greatest red shift. Furthermore, our results indicate that compound **C-2** is the best candidate from the entire group for reliable, artifact-free cytoplasmic staining. **C-2** achieves excellent contrast in the FITC/Alexa488 channel while maintaining statistically unchanged 3T3 metabolic activity. In terms of antibacterial activity, the best results were obtained for the **C-1** derivative, which demonstrated mild to moderate activity against Gram-positive bacteria. Compound **C-8** demonstrated the highest anticancer activity against cancer cells such as SNB-19, Caco-2, and A2058.

## 1. Introduction

Oxygen is an element found in the structure of countless chemical compounds. In organic compounds, the oxygen atom most often co-creates characteristic functional groups in ethers, alcohols, aldehydes, ketones, carboxylic acids, and esters. Ether and ester groups can act as linkers between two different molecular fragments. Furthermore, oxygen also successfully builds heterocyclic and heteroaromatic structures. The presence of oxygen in organic compounds often significantly modifies certain physicochemical properties compared to their oxygen-free analogs. A particularly interesting example of chemical compounds containing oxygen is derivatives of rhodanine-3-acetic acid [1,2,3,4,5,6,7,8,9,10,11,12,13,14,15,16,17,18,19]. In their case, the oxygen atom is located in the carboxyl group (at position 3) and in the ketone group directly connected to the heterocyclic ring at position 4. However, how will selected properties of rhodanine-3-acetic acid derivatives change if the substituent at position 5 contains additional functional groups rich in oxygen atoms? So far, a number of such compounds containing oxygen-containing moieties in the substituent under consideration have been described in the literature [1,2,3,4,5,6,7,8,9,10,11,12,13,14,15,16,17,18,19]. The vast majority of them were studied as enzyme inhibitors [1,3,6,7,8,10,13,14,16]. In particular, the studies described here focused on inhibiting aldose reductase. That is, blocking the enzyme that converts glucose into sorbitol [4,9,11,12,15,18,19]. This is crucial for preventing diabetes complications. Another example of research involved metallo-β-lactamase (MBL) inhibitors [13,14]. MBL is a group of bacterial enzymes that effectively destroy beta-lactam antibiotics, even carbapenems. Blocking these enzymes makes it possible to both reduce bacterial resistance and increase the effectiveness of antibiotics used. The considered rhodanine-3-acetic acid derivatives have also been studied as inhibitors of the following: DHDPS (dihydrodipicolinate synthase), to develop new antibacterial drugs (antibiotics) and herbicides [16]; iNOS, which blocks induced nitric oxide (NO) synthase produced during inflammation [10]; DXR (deoxyxylulose 5-phosphate reductoisomerase), the second enzyme of the MEP pathway [8]; ASK1, i.e., apoptosis signal regulating kinase 1 [7]; Dynamin GTPase, which blocks the enzymatic activity of dynamin and stops clathrin-dependent endocytosis [6]; hepatitis C virus NS5B polymerase [3] and DPMS (mannose dolichophosphate synthase) in Trypanosoma brucei, causing African sleeping sickness (trypanosomiasis) [1]. Moreover, selected derivatives of rhodanine-3-acetic acid containing additional functional groups based on an oxygen atom in the 5-position substituent were tested as antibacterial substances (antibiotics) [2,5]. The selected compounds showed activity mainly against certain Gram-positive bacteria [5]. According to the literature, compounds of this type were also tested for their ability to inhibit the ABCB1 efflux pump in mouse T-lymphoma cancer cells [17]. An analysis of the scientific literature regarding the influence of the methoxy group on the properties and potential applications of rhodanine-3-acetic acid derivatives reveals that the compounds in question have been investigated primarily as various types of inhibitors. This applies particularly to derivatives containing a single methoxy group attached to the phenyl ring at the 2- [16], 3- [4,15,20] or 4-position of the substituent [2,3,13,14,16,21,22]. The effect of a larger number of methoxy groups has so far been investigated to a limited extent [8,18]. Consequently, there is a significant lack of knowledge regarding how the number and substitution pattern of methoxy groups in the substituents affect the potential applications of rhodanine-3-acetic acid derivatives as fluorescent probes for bioimaging or as antibacterial, antifungal, and anticancer agents. Therefore, this article compares the influence of the substitution position and the number of methoxy groups attached to phenyl (derivatives **C-1**–**C-6**) and naphthalene (derivatives **C-7**–**C-8**) scaffolds on selected physicochemical and biological properties of rhodanine-3-acetic acid derivatives. First, all obtained derivatives were compared in terms of their thermal properties, specifically parameters such as melting point and thermal stability. Subsequently, the compounds under consideration were subjected to optical studies, which enabled the determination of absorption and emission maxima, quantum yields, and changes in dipole moments using the Lippert–Mataga equation. Based on the optical studies, all derivatives were tested as fluorescent probes for bioimaging. Furthermore, the synthesized compounds were tested for their antibacterial, antifungal, and anticancer properties. The experimental results were supported by detailed theoretical calculations.

## 2. Materials and Methods

Supplementary Materials contain all analyses and spectra supplementing the content in the main manuscript.

### 2.1. Materials

The list of reagents used is shown below: 4-methoxybenzaldehyde (98%, Merck (Sigma-Aldrich, Darmstadt, Germany)), 3,4-Dimethoxybenzaldehyde (>98%, TCI, Zwijndrecht, Belgium), 2,4-dimethoxybenzaldehyde (>98%, TCI, Zwijndrecht, Belgium), 3,5-dimethoxybenzaldehyde (>98%, TCI, Zwijndrecht, Belgium), 3,4,5-trimethoxybenzaldehyde (>98%, TCI, Zwijndrecht, Belgium), 2,4,6-trimethoxybenzaldehyde (>98%, TCI, Zwijndrecht, Belgium), 4-methoxy-1-naphthaldehyde (>97%, TCI, Zwijndrecht, Belgium), rhodanine-3-acetic acid (>98.0%, TCI, Zwijndrecht, Belgium), 6-methoxy-2-naphthaldehyde (98%, Merck (Sigma-Aldrich, Darmstadt, Germany)), ammonium acetate (puriss. p.a., ACS reagent, reag. Ph. Eur., ≥98%, Merck-Sigma-Aldrich, Darmstadt, Germany), glacial acetic acid (99,5% pure p. a, Chempur, Piekary Śląskie, Poland), DMSO-d_6_ (99.9 atom % D, Merck-Sigma-Aldrich, Darmstadt, Germany), ethanol (96% pure p. a, Stanlab Lublin, Poland), acetonitrile (for HPLC-GC, ≥99.8% (GC), Merck-Sigma-Aldrich, Darmstadt, Germany), chloroform (for HPLC, ≥99.8%, amylene stabilized, Merck-Sigma-Aldrich, Darmstadt, Germany), methanol (for HPLC, ≥99.9%, Merck-Sigma-Aldrich, Darmstadt, Germany), DMSO (pure for analysis, Chempur, Piekary Śląskie, Poland), THF (pure for analysis, Chempur, Piekary Śląskie, Poland), Toluene (pure for analysis, Chempur, Piekary Śląskie, Poland).

### 2.2. Measurements and General Methods

#### 2.2.1. General Apparatus

The equipment used for the measurements is presented below. Bruker Avance 500 MHz instrument (Billerica, MA, USA) was used to record the NMR spectra in DMSO-d6 (as solvent). Differential scanning calorimetry (DSC) and thermogravimetric analysis (TGA) were performed on the following devices: TA–DSC 25 (New Castle, DE, USA) and TA–TGA 55 (New Castle, DE, USA). UV/Vis spectra were recorded with a Biosens model UV 5600 UV/Vis spectrophotometer (Warszawa, Poland). Photoluminescence emission spectra were acquired using a Hitachi Fluorescence Spectrophotometer F-7100 (Tokyo, Japan). The photoluminescence quantum yield of all rhodanine-3-acetic acid derivatives (**C1**–**C8**) was measured using an integrating sphere at room temperature and using the solvent (DMSO) as a blank.

#### 2.2.2. In Vitro Antimicrobial Assay

The examined compounds **C-1**–**C-8** were screened in vitro for antibacterial and antifungal activities using the broth microdilution method according to European Committee on Antimicrobial Susceptibility Testing (EUCAST) [23] and Clinical and Laboratory Standards Institute guidelines [24] against a panel of 20 reference strains of microorganisms, including Gram-positive bacteria (*Staphylococcus aureus* ATCC 43300 (*Methicillin Resistant S. aureus*—MRSA), *Staphylococcus aureus* ATCC 29213 (*Methicillin Susceptible S. aureus*—MSSA), *Staphylococcus aureus* ATCC 25923 (*Methicillin Susceptible S. aureus*—MSSA), *Staphylococcus epidermidis* ATCC 12228, *Enterococcus faecalis* ATCC 29212, *Micrococcus luteus* ATCC 10240, *Bacillus subtilis* ATCC 6633 and *Bacillus cereus* ATCC 10876), Gram-negative bacteria (*Escherichia coli* ATCC 25922, *Klebsiella pneumoniae* ATCC 13883, *Proteus mirabilis* ATCC 12453, *Salmonella Typhimurium* ATCC 14028 and *Pseudomonas aeruginosa* ATCC 9027) and fungi belonging to yeasts (*Candida albicans* ATCC 10231, *Candida parapsilosis* ATCC 22019, *Candida glabrata* ATCC 90030, *Candida krusei* ATCC 14243, *Candida tropicalis* ATCC 1369, *Candida lusitaniae* ATCC 3449, and *Candida auris* CDC B11903). The microorganisms came from the American Type Culture Collection (ATCC) and CDC (Centers for Disease Control and Prevention), routinely used for the evaluation of antimicrobials. All the used microbial cultures were first subcultured on nutrient agar or Sabouraud agar at 35 °C for 18–24 h or 30 °C for 24–48 h for bacteria and fungi, respectively.

Samples containing the examined compounds **C-1**–**C-8** were first dissolved in dimethyl sulfoxide (DMSO) (20 mg/mL). Subsequently, the MIC (Minimal Inhibitory Concentration) of these substances was examined by the microdilution broth method, using their two-fold dilutions in Mueller-Hinton broth (for bacteria) and RPMI 1640 broth with MOPS (for fungi) prepared in 96-well polystyrene plates. Final concentrations of the compounds ranged from 2000 to 15.62 µg/mL. Microbial suspensions were prepared in 0.85% NaCl with an optical density of 0.5 McFarland standard. Next bacterial or fungal suspensions were added to each well containing broth and various concentrations of the examined compounds. After incubation (under the same conditions as before), the MIC was assessed spectrophotometrically as the lowest concentration of the samples showing complete bacterial or fungal growth inhibition. Appropriate growth and sterile controls were carried out. The medium with no tested substances was also used as a control. The inhibition of microbial growth was judged by comparison with a control culture prepared without any sample tested. Ciprofloxacin, vancomycin or nystatin (Sigma-Aldrich Chemicals, St. Louis, MO, USA) were used as reference antibacterial or antifungal compounds, respectively.

MBC (Minimal Bactericidal Concentration) and MFC (Minimal Fungicidal Concentration) are defined as the lowest concentration of the compounds that is required to kill a particular bacterial or fungal species. MBC or MFC was determined by removing the culture used for MIC determinations from each well and spotting onto appropriate agar medium. Next, the plates were incubated. The lowest compound concentrations with no visible growth observed were assessed as bactericidal or fungicidal concentrations. All the experiments were repeated three times, and representative data are presented [25,26].

In antimicrobial activity studies, no bioactivity was defined as a MIC > 1000 µg/mL, mild bioactivity as a MIC in the range 501–1000 µg/mL, moderate bioactivity with MIC from 126 to 500 µg/mL, good bioactivity as a MIC in the range 26–125 µg/mL, strong bioactivity with MIC between 10 and 25 µg/mL and very strong bioactivity as a MIC < 10 µg/mL [27]. The MBC/MIC or MFC/MIC ratios were calculated in order to determine bactericidal/fungicidal (MBC/MIC ≤ 4, MFC/MIC ≤ 4) or bacteriostatic/fungistatic (MBC/MIC > 4, MFC/MIC > 4) effect of the tested compounds.

#### 2.2.3. Theoretical Calculations—Methodology

All geometrical parameters of investigated molecules in their ground (SGS) and excited (SCT) states were calculated using a density functional theory (DFT) approach implemented in the Gaussian 16 program package [28] with the PBE0/6-311++G(d,p) basis set. In order to verify that all the structures correspond to the minima on the potential energy surface, an analysis of Hessians was performed. The electronic properties were characterized by computations of the vertical absorption and were obtained using the time-dependent density functional theory (TDDFT/PBE0) [29] and by including the state-specific (SS) corrected linear response (cLR) approach [30]. Based on previous experience with derivatives of this type [31,32,33], all calculations of linear and nonlinear optical properties were performed using the PBE0 functional.

The dipole moments and polarities of the charge-transfer state (CT) were evaluated by numerical differentiation of the excitation energies (E) in the presence of an electric field F of 0.001 a.u. strength:

(1) $$∆\mu_{\mathrm{CT}-\mathrm{GS}}^{i}=\frac{E_{\mathrm{CT}}\left(+F^{i}\right)-E_{\mathrm{CT}}\left(-F^{i}\right)}{-2F^{i}}-\frac{E_{\mathrm{GS}}\left(+F^{i}\right)-E_{\mathrm{GS}}\left(-F^{i}\right)}{-2F^{i}}$$

where i stands for the Cartesian component of the dipole moment difference.

The isotropic average polarizability (α) and first-order hyperpolarizability (*β*) were determined based on the Gaussian 16 program and defined as

(2) $$\alpha =\frac{\alpha_{\mathrm{xx}}+\alpha_{\mathrm{yy}}+\alpha_{\mathrm{zz}}}{3}$$

(3) $$\beta =\sum_{i=x,y,z}\frac{\mu_{i}\beta_{i}}{\left|\mu \right|}$$

where *β_i_*(*i* = *x*,*y*,*z*) is given by $\beta_{i}=\left(\frac{1}{3}\right)\sum_{j=x,y,z}\left(\beta_{\mathrm{ijj}}+\beta_{\mathrm{jij}}+\beta_{\mathrm{jji}}\right)$.

The density differences were obtained at the PBE0/6-311++G(d,p) level and are represented with a contour threshold of 0.02 a.u. In these graphs, the blue (purple) zones indicate a density decrease (increase) upon electronic transition. The charge transfer parameters, namely the charge-transfer distance (DCT) and the amount of transferred charge (qCT), have been determined following a Le Bahers’ procedure [34].

The solvent effect on the linear and nonlinear optical properties has been taken into account using the Integral Equation Formalism for the Polarizable Continuum Model (IEF–PCM) [35,36].

The biological activities were simulated using a combination of the 3D/4D QSAR BiS/MC and CoCon algorithms [37,38,39].

## 3. Results and Discussion

### 3.1. Synthetic Procedure and Characterization of Rhodanine-3-Acetic Acid Derivatives

**Figure 1:**
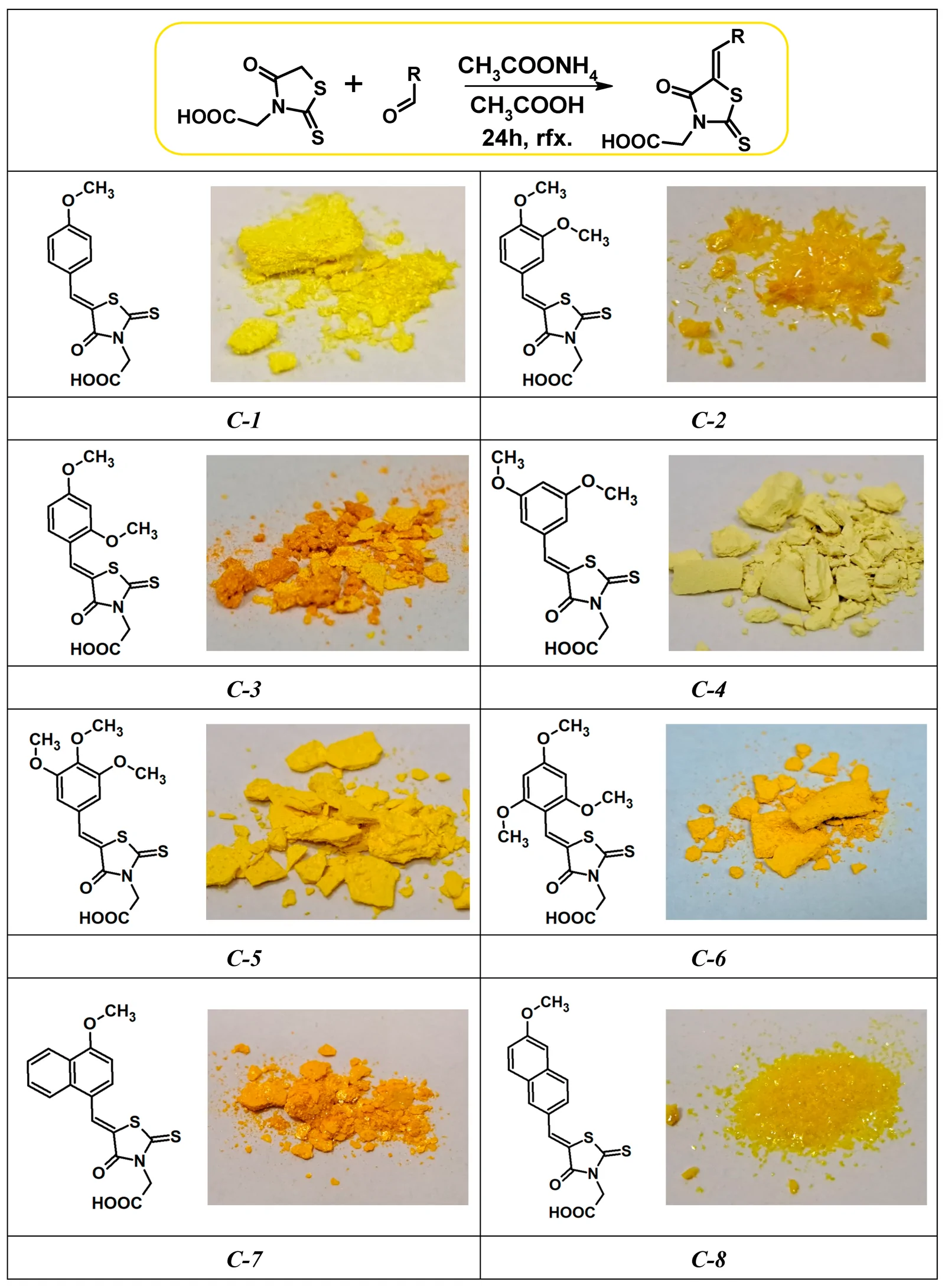
Synthesis scheme and structures of the obtained compounds (**C1**–**C8**) with photographs of the precipitates.

The condensation method was chosen for the synthesis of rhodanine-3-acetic acid derivatives (**C-1**–**C-8**). These reactions involved rhodanine-3-acetic acid and eight aromatic aldehydes bearing a phenyl or naphthalene skeleton, each bearing a methoxy group (Figure 1). The methoxy groups were located in various positions. Furthermore, in the case of the phenyl substituent, the effect of varying the number of methoxy groups (from 1 to 3) was considered. Ammonium acetate was an additional reagent enabling efficient condensation. Acetic acid was used as the solvent. The syntheses were completed by cooling to room temperature and adding distilled water. The resulting precipitates were filtered, thoroughly washed with water, and then dried, yielding yellow or orange powders. The yields of the reactions ranged from 49% to 99% and were largely dependent on the aldehyde used. The structures of the obtained compounds and photographs of the precipitates are shown in Figure 1.

NMR spectroscopic methods confirmed the structures of the obtained rhodanine-3-acetic acid derivatives (**C-1**–**C-8**). To better understand the structural relationships of the compounds under consideration, 2D NMR experiments were also performed, including ^1^H-^1^H COSY, ^1^H-^13^C HMQC, and ^1^H-^13^C HMBC. All NMR spectra are listed in the SI, Section S3 (synthesis and NMR spectra—Section S3). The structures of the analyzed compounds were divided into two parts: the rhodanine-3-acetic acid ring (A) and the substituents (B). Confirming the presence of the rhodanine-3-acetic acid ring in ^1^H NMR spectra is not easy. The structural fragment for the considered compounds (**C-1**–**C-8**) can be observed in the form of signals from protons: A1 from carboxyl groups (-COOH) (in the range from 13.34 to 13.47 ppm), A2 from aliphatic groups (-CH_2_) (in the range from 4.69 to 4.77 ppm), and A8 at double bonds (-CH=C-) (in the range from 7.83 to 8.47 ppm). The signal from protons A8 additionally confirms the positive course of the condensation reaction. Analyzing the ^1^H NMR spectra during the condensation process, we can observe that the characteristic signals from aldehyde groups (-CHO) systematically decrease, while A8 appears. In the analysis of the rhodanine-3-acetic acid ring, we observe a number of signals from different structural fragments in the ^13^C NMR spectra. The C=S moiety, designated A7, is observed at very high values ranging from 193.08 to 194.96 ppm. Carbonyl groups A1 and A4 can be distinguished by detailed analysis of the HMBC spectrum. Interestingly, the signal from carbon A1 (C=O, range from 167.27 to 167.55 ppm) always occurs at slightly higher values than A4 (C=O, range from 165.99 to 167.11 ppm). Signals from carbons A5 and A8 located in the double bond (-CH=C-) occur over a wide range from 118.02 to 134.57 ppm, with significantly lower values than the thiocarbonyl and carbonyl carbons. Aliphatic carbons A2 can be observed as signals at relatively low values ranging from 44.89 to 45.04 ppm. Phenyl (**C-1**–**C-6**) and naphthalene (**C-7**–**C-8**) substituents are observed in ^1^H and ^13^C NMR spectra as standard signals in the ranges 6.34–8.27 ppm (^1^H NMR) and 91.21–165.50 ppm (^13^C NMR). In ^1^H NMR spectra, the multiplicity of signals from hydrogen atoms in the substituent varies due to the presence of varying numbers of methoxy groups and their substitution positions. Methoxy groups appear in the spectra as singlets over narrow ranges. In ^1^H NMR spectra, this ranges from 3.75 to 4.07 ppm, while in ^13^C NMR it ranges from 55.50 to 60.29 ppm. The number of signals from methoxy groups largely depends on the symmetry of the molecules, particularly in **C-2**–**C-6** compounds.

**Figure 2:**
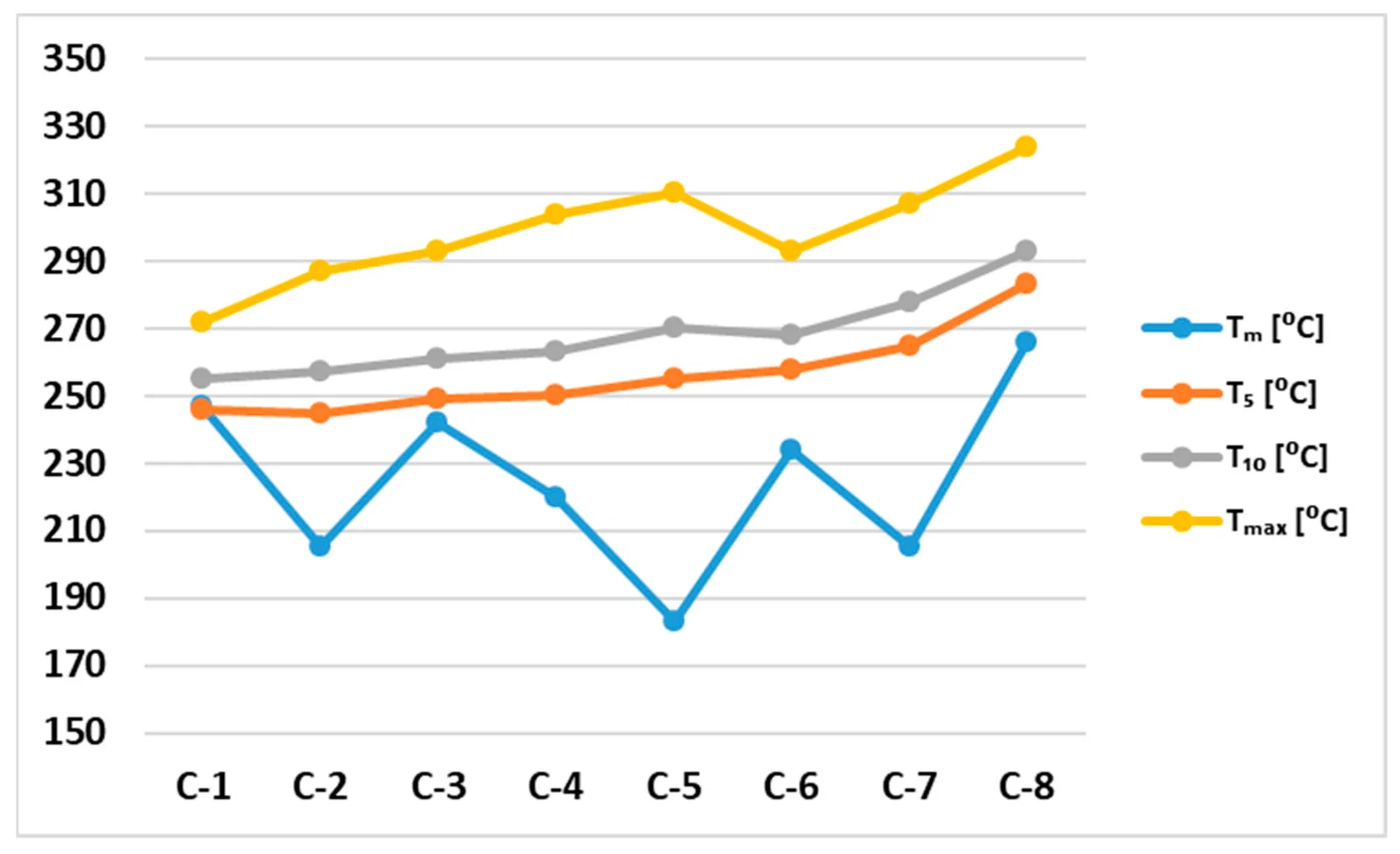
Thermal test results obtained for the C-series.

Determination of the thermal properties of the studied rhodanine-3-acetic acid derivatives (**C-1**–**C-8**) was possible thanks to differential scanning calorimetry (DSC) and thermogravimetric analysis (TGA) measurements (Table 1). All thermal studies were performed under a nitrogen atmosphere. DSC analysis allowed the determination of the melting temperatures (T_m_) for all the derivatives considered. They fell within a wide range of 183–266 °C. In general, increasing the number of methoxy groups in the substituent lowers the melting temperature (T_m_). However, the position of substitution of the mentioned functional group significantly affects this parameter. The highest melting temperatures were observed for derivatives **C-1** (T_m_ = 247 °C) and **C-8** (T_m_ = 266 °C). Increasing the number of methoxy groups to two in the phenyl substituent (**C-2**–**C-4** derivatives) resulted in a decrease of this parameter by 5 °C (**C-3**), 27 °C (**C-4**), and 42 °C (**C-2**) relative to **C-1**. A similar effect was observed for compounds containing three methoxy groups (**C-5**–**C-6**). For the **C-5** and **C-6** derivatives, a decrease in melting point was observed by 64 °C and 13 °C, respectively, compared to **C-1**. In the case of compounds containing naphthalene and one methoxy group in the substituent (**C-7** and **C-8**), a significant difference in melting point was observed. The C-7 derivative showed a decrease in this thermal parameter by 61 °C relative to the **C-8** compound. TGA analysis enabled the determination of thermal parameters such as 5% (T_5_) and 10% (T_10_) mass loss temperatures, as well as the temperature of maximum decomposition rate (T_max_). Analyzing the obtained results, we can see that all derivatives exhibit thermal stability (T_5_) above 240 °C (Figure 2). Furthermore, increasing the number of methoxy groups on the phenyl substituent results in a gradual increase in the considered parameter from 246 °C at **C-1** to 258 °C at **C-6**. The only exception is compound **C-2**, which shows a 1 °C decrease in thermal stability relative to **C-1**. Considering T_5_ for derivatives containing naphthalene in the substituent, we can see a slight difference in thermal stability (by 18 °C) in favor of **C-8** (283 °C) compared to **C-7** (265 °C). The T_max_ for all compounds is observed in the range 272–324 °C and generally increases with the number of methoxy groups. Only the **C-6** derivative shows an exception. All thermal test results are summarized in Table 1, while the DSC (Figures S1–S8) and TGA (Figure S9) thermograms are included in the SI, point 4 (thermal properties).

### 3.2. Theoretical Studies

#### 3.2.1. Computational Characterization of Charge Transfer and Excited-State Properties

In Table S1, the energies of the frontier molecular orbitals HOMO and LUMO calculated for the C-series compounds in various solvents are presented, together with the corresponding conceptual DFT parameters (energy gap—Δ*E*_GAP_; absolute electronegativity—χ; chemical potentials—*μ*; absolute hardness—*η*; absolute softness—*σ*; global electrophilicity—*ω*; global softness—*S*). The collected data indicate a characteristic functional separation between the donor and acceptor regions, consistent with the D–A architecture present in all molecules. The HOMO is mainly localized on the heterocyclic fragment containing N and S atoms, whereas the LUMO is concentrated on the carbonyl groups and the terminal aromatic fragment (Figure S10), which favors the formation of ICT-type electronic transitions. The HOMO energies fall within approximately -6.60 to -6.04 eV, while the LUMO energies range from -2.89 to -2.40 eV. Compounds with a more electron-donating substituent (e.g., **C-6**) display elevated HOMO energies, whereas systems with a stronger acceptor component (**C-4**, **C-5**, **C-8**) exhibit lowered LUMO levels. These changes directly affect the HOMO–LUMO energy gap, which across the entire series spans 3.29–3.76 eV. The lowest Δ*E*_GAP_ is observed for compound **C-7**, indicating the most efficient donor–acceptor coupling and the highest susceptibility to electronic excitation. The influence of the solvent is reflected in the systematic decrease in both orbital energies in more polar environments, with stabilization being more pronounced for the LUMO. This effect results in a slight reduction in Δ*E*_GAP_ and increases the charge-transfer character of the excited state. Differences between toluene and DMSO are the most distinct; in polar solvents, the structures exhibit higher polarizability and an enhanced ability to reorganize electron density. The conceptual DFT parameters further show that the absolute electronegativity *χ* increases with the strengthening of the acceptor component, reaching the highest values for **C-4**, **C-5** and **C-8**. The highest chemical potential, strongest donor character, is found for **C-6**, which is consistent with its elevated HOMO level. The lowest hardness ***η*** and the highest softness **σ** and **S** are noted for **C-7**, a consequence of its smallest energy gap, reflecting its high excitability and strong electronic polarizability. The electrophilicity index *ω* confirms the predominance of acceptor character in **C-4**, **C-5** and **C-8**, while **C-6** remains the most donor-like structure. These energetic descriptors demonstrate that the properties of the C-series acids are strongly modulated by both the nature of the substituents and the solvation environment, and the observed trends are fully consistent with the expected donor–acceptor behavior of these systems.

Table S2 presents the Natural Bond Orbital (NBO) analysis for the C-series compounds in various solvation environments. The obtained bond occupancies, orbital energies, and *sp*-hybridization patterns clearly support the conclusions drawn from the HOMO–LUMO analysis, indicating a consistent electronic description of the donor–acceptor systems. In all cases, distinctly polarized C–N, C–C, and C–O– bonds are observed, with polarity values of approximately 63–67% located on the more electronegative atoms (N or O). This is fully consistent with the orbital picture, in which the HOMO was predominantly localized on the donor fragment containing the nitrogen atom, whereas the acceptor region associated with the C–O– functionality naturally accommodates a substantial portion of the electron density forming the LUMO. The NBO bond polarization indicates that the nitrogen atom in the donor center exhibits *sp*^2^-type hybridization with a pronounced *p*-character (~67%), which stabilizes its role as an electron donor in ICT-type transitions. Likewise, the oxygen atoms in the C–O– units show a strongly polarized character (around 67% on O), confirming their dominant acceptor contribution. The occupancies of the bonding orbitals (typically ~1.97–1.99 e) point to well-localized *σ*-bonds and the absence of significant electronic perturbations in the ground state. In contrast, the high occupancies and negative energies of the lone pairs on O and N atoms demonstrate that these non-bonding orbitals serve as the main electron reservoir capable of being transferred to the acceptor fragment upon excitation. This behavior mirrors the HOMO localization pattern, further confirming that NBO and HOMO–LUMO analyses describe the same dominant donor-to-acceptor (D→A) charge-transfer pathway.

A comparison of individual compounds reveals that substituent effects modulate the local polarization and the energies of NBO orbitals. In **C-4**, **C-5**, and **C-8**, which exhibit a stronger acceptor component, the non-bonding LP(O) orbitals are noticeably lower in energy, and the C–O– bonds display enhanced polarization. This trend is fully consistent with their lower LUMO energies and higher electronegativity derived from the HOMO–LUMO analysis. Conversely, **C-6** shows the highest local electron density on the nitrogen atom and the greatest *p*-character in the hybridization of the donor C–N environment, which correlates with its elevated HOMO energy and more pronounced donor character. The **C-7** derivative, previously identified as having the smallest Δ*E*_GAP_, also exhibits the strongest delocalization of electron density along the C–N and C–C bonds, reinforcing its efficient donor–acceptor electronic coupling. Notably, solvent-induced changes are mild but systematic in both HOMO–LUMO and NBO analyses: increasing solvent polarity leads to a slight increase in C–O– and C–N bond polarization and small shifts in the energies of non-bonding orbitals. This behavior aligns with the stronger stabilization of the LUMO in polar environments (MeOH, MeCN, DMSO), arising naturally from the enhanced polarizability of the local NBO orbitals. The hybridization analysis shows that the donor fragments, N and the adjacent carbon atoms, possess higher *p*-character, promoting electron delocalization and facilitating charge transfer. In contrast, the oxygen atoms in the C–O– units exhibit hybridization in the *sp*^1.8^–*sp*^2^ range, characteristic of fragments that enhance charge-transfer interactions and stabilize ICT-type excited states. This hybridization pattern is fully consistent with the molecular geometries and the reduced Δ*E*_GAP_ previously identified for the C-series.

From the perspective of fluorescence applications, the agreement between NBO and HOMO–LUMO analysis is essential. The strongly polarized C–N and C–O– bonds, the presence of high-energy lone pairs on oxygen, and the clear separation of donor and acceptor centers collectively create a framework favorable for generating ICT-dominated excited states. Since the efficiency of environment-responsive fluorescence depends strongly on the magnitude and direction of charge redistribution, the NBO data confirm the structural characteristics that make the C-series compounds, particularly **C-6**, **C-7**, and the acceptor-enhanced **C-4**, **C-5**, and **C-8**, promising candidates for fluorescent probes.

Figure S11 presents the density-difference (Δρ) maps for compounds **C-1**–**C-8** in the S_1_ excited state. The blue regions correspond to areas of electron depletion on the donor fragment, whereas the purple regions indicate electron accumulation on the acceptor fragment. All molecules display a clear D→A charge-transfer direction; however, the spatial separation between depletion and accumulation zones differs across the series, reflecting variations in ICT strength. Compounds **C-4**, **C-7** and **C-8** exhibit the largest separation between these regions, confirming a strongly polarized excited state, while **C-2**, **C-3**, and **C-6** show a more compact Δρ distribution.

Table S3 summarizes the charge-transfer parameters derived from density difference analysis for compounds **C-1**–**C-8** in solvents of increasing polarity. The values of transferred charge (q_CT_) and charge-transfer distance (D_CT_) confirm the ICT-type nature of the S_1_ state and complement the previously discussed HOMO–LUMO and NBO results. For **C-1**–**C-4**, the q_CT_ values in toluene are high, ranging from 0.830 e (**C-1**) to 0.838 e (**C-4**), indicating a strong ICT component even in a nonpolar environment. In chloroform, q_CT_ decreases slightly for **C-2** (0.492 e) and **C-3** (0.462 e), while it increases markedly for **C-4** (0.889 e). In more polar solvents (THF, MeOH, MeCN, DMSO), the q_CT_ of **C-2** stabilizes between 0.494 and 0.504 e, whereas **C-3** remains nearly constant (0.462–0.468 e). The strongest increase is again observed for **C-4**, reaching 0.930 e in MeOH and MeCN, and 0.928 e in DMSO, confirming pronounced strengthening of charge separation in polar media.

The corresponding D_CT_ values further differentiate between molecules with localized versus spatially extended CT. For **C-1**–**C-3**, D_CT_ increases moderately with solvent polarity, from 1.930 Å (**C-1**) and 1.194 Å (**C-3**) in toluene to 2.579 Å and 1.746 Å, respectively, in DMSO. In contrast, **C-4** exhibits a dramatic elongation of D_CT_, from 1.860 Å in toluene to 3.693 Å in DMSO, indicating substantial spatial separation between donor and acceptor barycenters in the excited state. Analysis of **C-5**–**C-8** reveals consistently large q_CT_ values across all solvents. **C-5** maintains a nearly constant q_CT_ between 0.839 and 0.846 e, suggesting a structurally driven, highly stable CT character. For **C-6**, q_CT_ remains lower (0.455–0.470 e), consistent with its weaker donor strength inferred from orbital analysis. Compounds **C-7** and **C-8** exhibit a clear solvent-dependent enhancement of charge transfer: q_CT_ for **C-7** increases from 0.539 e (toluene) to 0.558 e (MeOH/MeCN), whereas for **C-8** it rises from 0.557 e (toluene) to 0.593 e (DMSO). The behavior of D_CT_ in this subset reinforces the trend: **C-7** shows a continuous increase from 2.690 Å (toluene) to 3.134 Å (DMSO), while **C-8** displays the largest D_CT_ values in the entire series, ranging from 3.441 Å up to 4.126 Å (MeCN) and 4.100 Å (DMSO). These data confirm that **C-7** and especially **C-8** possess highly separated donor–acceptor regions in the excited state.

The q_CT_ parameter exhibits two distinct behavioral patterns across the C-series. For molecules with well-defined donor–acceptor separation (e.g., **C-4**, **C-7**, **C-8**), increasing solvent polarity produces a monotonic rise in q_CT_, reflecting dominant stabilization of the acceptor fragment and reinforcement of the D→A charge-transfer pathway. In contrast, compounds such as **C-2**, **C-3**, and **C-6** display a non-monotonic response, arising from a competition between solvent stabilization of donor and acceptor centers and possible ICT/TICT conformational equilibria. This divergence is consistent with their HOMO–LUMO characteristics and NBO polarization patterns, and indicates that monotonic systems are better suited for polarity-responsive fluorescence sensing, while non-monotonic systems may be more sensitive to subtle microenvironmental effects.

**Figure 3:**
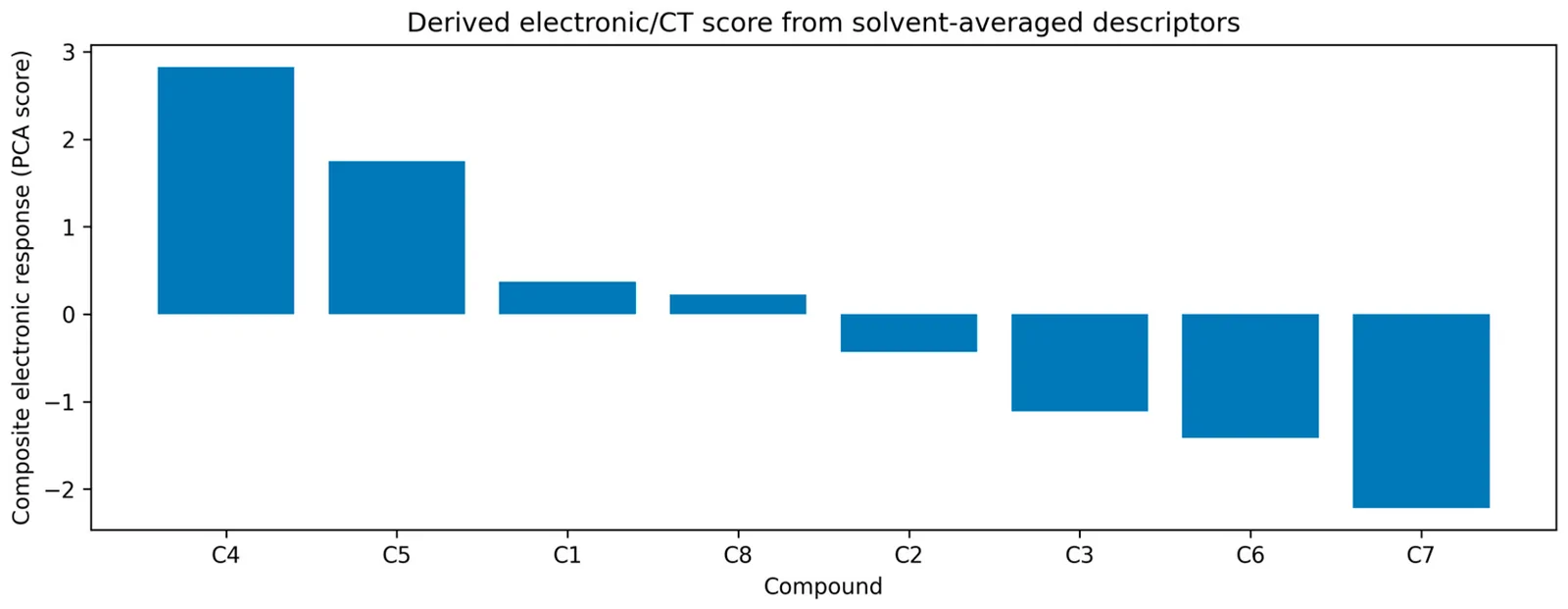
Composite electronic response for compounds **C1**–**C8** obtained from a principal-component score computed on solvent-averaged descriptors (ΔE_GAP_, q_CT_, D_CT_, Δμ_CT-GS_ and vertical λ_abs_). Higher values indicate a stronger overall charge-transfer/electronic response captured consistently across solvents.

Taken together, the q_CT_ and D_CT_ values indicate that **C-4**, **C-7** and **C-8** exhibit the strongest and most spatially extended charge transfer, whereas **C-2**, **C-3** and **C-6** show a more compact CT nature despite consistent electron redistribution. **C-5** remains a structurally stable CT system with high q_CT_ but moderate D_CT_ values. The excellent agreement between q_CT_/D_CT_ trends and previously discussed HOMO–LUMO and NBO analyses reinforces the conclusion that intramolecular charge transfer governs the photophysics of these molecules. This multi-parameter agreement is further condensed in the composite electronic-response metric (Figure 3), which integrates solvent-averaged ΔE_GAP_, q_CT_, D_CT_, Δμ_CT-GS_ and vertical λ_abs_ into a single score describing the overall charge-transfer/electronic response of the C–series. The compounds with the largest q_CT_ and D_CT_ values, **C-4**, **C-7** and **C-8**, emerge as the most promising candidates for polarity-sensitive fluorescence and bioimaging applications, as their excited-state response is strongly modulated by the solvent environment.

Figure S12 presents the spatial distribution of the electrostatic potential (MEP) for compounds **C-1**–**C-8**, mapped onto the electron density surface of the molecules. The most electronegative regions (shown in red), located around the oxygen atoms in the C–O– bonds and in the vicinity of the donor nitrogen center, correspond to the lowest potential values, down to −0.07000 for **C-4**. These areas represent preferential sites for interactions with positively charged species such as protons, metal cations, or positively charged biomolecular fragments. In contrast, the positive potential regions (blue), with values up to +0.07000, appear near hydrogen atoms and less electronegative aromatic fragments, indicating electrostatically neutral or weakly positive domains. The numerical MEP values, from ±0.06592 to ±0.07000, reveal that the compounds with the strongest electrostatic contrast, **C-3** (±0.06932), **C-4** (±0.07000) and **C-8** (±0.06950), exhibit the most pronounced charge polarization. These molecules also showed the highest q_CT/DCT_ values and increased C–N/C–O– bond polarization in the NBO analysis, confirming their dominant ICT character. In contrast, **C-6** (±0.06592) displays the narrowest MEP range, consistent with its weaker charge transfer and more moderate donor–acceptor coupling. The spatial MEP patterns provide additional insights into potential intermolecular interactions. The intense red areas around O and N atoms indicate a strong ability to engage in electrostatic interactions, hydrogen bonding, or binding to receptor sites with positively charged domains. Conversely, the blue regions surrounding alkyl and aromatic fragments mark zones susceptible to interactions with anions or negatively charged protein residues. Importantly, the differences in MEP among the compounds correlate with the trends observed in solvation free energies (ΔGsol, Table S4). Molecules with the most pronounced electrostatic contrast (e.g., **C-4**, **C-7**, **C-8**) experience stronger stabilization in polar solvents, in agreement with their enhanced charge-transfer character and lower LUMO energies. This highlights the key role of charge distribution in determining both the photophysical properties and the interactions of these systems with their local microenvironment.

The analysis of solvation free energies for the tested compounds demonstrates a systematic stabilization of all molecules in solvents of increasing polarity. The strongest solvation effects are observed for **C-4**, **C-7** and **C-8**, which also exhibit the highest MEP values and the most pronounced charge-transfer character. Their strongly polarized electrostatic potential distribution facilitates efficient interactions with polar media, resulting in noticeably more negative ΔGsol values. In contrast, molecules with a less contrasted charge distribution, such as **C-2**, **C-3** and particularly **C-6**, show weaker solvation stabilization, consistent with their more moderate CT character and higher LUMO energies. The convergence of the ΔGsol trends with MEP and charge-transfer parameters confirms that solvation plays a key role in modulating the photophysical properties of the C-series and amplifies substituent-dependent differences between individual compounds.

#### 3.2.2. Linear and Nonlinear Optical Properties

Table S5 presents the absorption maxima determined by TD-DFT as vertical (λ_abs_) values and within the cLR approximation. Analysis of the mean absolute errors clearly demonstrates that the vertical approach provides significantly better agreement with the experimental data. The mean errors for the vertical values amount to 4.12 nm (**C-1**), 3.10 nm (**C-2**), 3.33 nm (**C-3**), 2.72 nm (**C-4**), 3.53 nm (**C-5**), 3.50 nm (**C-6**), 3.87 nm (**C-7**), and 4.63 nm (**C-8**), indicating a stable and consistent quality of description across the entire series. In contrast, the cLR approximation yields substantially larger and more structure-dependent mean errors, reaching 5.20 nm (**C-1**), 11.60 nm (**C-2**), 13.48 nm (**C-3**), 29.75 nm (**C-4**), 1.74 nm (**C-5**), 10.92 nm (**C-6**), 7.39 nm (**C-7**), and 18.16 nm (**C-8**). The largest individual discrepancies within the cLR approach are observed for compound **C-4** in DMSO, where the deviation exceeds 40 nm, as well as for **C-3** and **C-8** in highly polar solvents (MeCN and DMSO). These results clearly indicate that, in such cases, excited-state relaxation described within the cLR framework leads to a significant overestimation of bathochromic shifts. Consequently, further analysis of substituent and solvent effects on the positions of absorption bands was based exclusively on vertical values, which not only reproduce the experimental data more accurately but also preserve the observed structural trends across the series.

Analysis of the absorption band positions reveals that the entire series exhibits distinct yet moderate solvation effects. For most compounds, an increase in solvent polarity results in positive solvatochromism, manifested as a shift of the absorption maximum toward longer wavelengths. This effect is particularly pronounced for compounds **C-7** and **C-8**, for which λ_abs_ increases systematically from nonpolar to highly polar environments, indicating stabilization of an excited state with a significant charge-transfer contribution. In contrast, for compounds **C-1**–**C-3**, changes in absorption maxima are minor and, in some cases, weakly non-monotonic, suggesting a more localized character of the electronic transition and reduced sensitivity to solvation effects. Notably, no clear negative solvatochromism is observed for any of the investigated compounds, confirming that the excited state is at least as polarized as the ground state.

Comparison of absorption maxima among individual molecules reveals a pronounced influence of substituents on band positions. The shortest absorption wavelengths, corresponding to a hypsochromic shift relative to the remaining systems, are observed for compound **C-4**, indicating the weakest effective donor–acceptor coupling within this structure. Compounds **C-1**–**C-3** absorb within a similar wavelength range, forming an intermediate group characterized by moderate bathochromic shifts. The most strongly bathochromically shifted absorption maxima are observed for **C-7** and **C-8**, and to a lesser extent for **C-6**, reflecting an extended π-conjugation and an increased contribution of charge-transfer-type transitions. These differences are preserved in all analyzed solvents, demonstrating that substituent effects dominate over solvent effects and systematically modulate the excitation energy.

The data summarized in Table S6 indicate that the change in dipole moment upon excitation varies significantly across the **C-1**–**C-8** series and correlates directly with the nature of the electronic transitions and the observed solvatochromic trends in the absorption spectra. The smallest Δμ_CT-GS_ values are observed for compounds **C-1**, **C-4**, and **C-7**. For **C-1**, the change in dipole moment in nonpolar solvents remains very small (1.96 D in toluene and 1.57 D in chloroform), with only a moderate increase in polar environments, reaching approximately 4.40 D in MeCN and DMSO. Similarly, compound **C-4** exhibits relatively low and weakly varying Δμ_CT-GS_ values (3.32 D in toluene to ca. 4.40 D in DMSO), indicating a limited redistribution of charge density upon excitation. The most distinctive behavior is observed for **C-7**, which displays the lowest Δμ_CT-GS_ values in the entire series and remains weakly polarized even in polar solvents (2.04–2.88 D), with a more pronounced increase only in DMSO (3.84 D). This behavior suggests a dominant contribution of locally excited or weakly developed charge-transfer states, despite the significantly extended π-conjugation present in the structure of **C-7**. In contrast, compounds **C-5** and **C-6** exhibit the highest Δμ_CT-GS_ values in the series. For **C-5**, a strong and monotonic increase in the dipole moment change with increasing solvent polarity is observed, from 12.72 D in toluene to 14.93 D in DMSO, clearly indicating an excited state of pronounced intramolecular charge-transfer character. A similar, though slightly weaker, trend is found for **C-6** (10.60–12.38 D). Compounds **C-2** and **C-3** occupy an intermediate position, exhibiting Δμ_CT-GS_ values in the range of 8.42–11.81 D that increase systematically with solvent polarity, confirming the presence of a significant but less spatially separated CT component. The observed differences in Δμ_CT-GS_ are closely related to the positions and solvent sensitivity of the absorption maxima. Compounds displaying the largest dipole-moment changes (**C-5**, **C-6**, and partially **C-2** and **C-3**) exhibit the strongest positive solvatochromism, resulting from efficient stabilization of highly polarized excited states in polar media. In contrast, molecules with small Δμ_CT-GS_ values, such as **C-1**, **C-4**, and **C-7**, show a much weaker dependence of absorption band positions on solvent polarity. In particular, the case of **C-7** demonstrates that the bathochromic shift of its absorption maximum arises primarily from extended π-conjugation rather than from an increased polarity of the excited state.

Table S7 summarizes the fluorescence maxima (λ_em_) determined by TD-DFT. Analysis of these data indicates good overall agreement between theory and experiment, although the observed discrepancies depend strongly on both molecular structure and solvation environment. The smallest mean errors are obtained for **C-4** (2.15 nm) and **C-1** (3.66 nm), indicating a very accurate description of excited-state relaxation in these systems. For compounds **C-2**, **C-3**, **C-6**, and **C-7**, the mean deviations fall within 3.84–4.71 nm, whereas the largest discrepancies are observed for **C-5** and **C-8**, with mean errors of 5.28 nm and 5.50 nm, respectively. From a solvent perspective, the largest positive deviations, overestimation of emission wavelengths, are typically observed in toluene and THF, reaching 9.45 nm for **C-3** and 7.33 nm for **C-7**, while in highly polar solvents (MeCN and DMSO) negative deviations are more frequently observed, indicating a slight underestimation of λ_em_ by TD-DFT. This behavior is characteristic of fluorescence calculations and reflects the sensitivity of the geometric relaxation of the lowest excited state to the solvation model.

The positions of fluorescence maxima reveal pronounced structural and solvatochromic trends, analogous to those observed for absorption but with larger shift amplitudes. For compounds **C-1**–**C-3**, fluorescence maxima are located within a narrow range of 454–480 nm and exhibit only a weak, partially non-monotonic dependence on solvent polarity, consistent with a limited charge-transfer character of the excited state. Compound **C-4** shows the shortest emission wavelengths in the entire series (435–442 nm), confirming its more locally excited character and remaining fully consistent with its small Δμ_CT-GS_ values and weak solvatochromic sensitivity discussed above. In contrast, compounds **C-5**–**C-8** display clear positive solvatochromism of fluorescence. For **C-5**, the emission maximum shifts from 468 nm in toluene to 503 nm in DMSO, while for **C-8** it shifts from 477 nm to approximately 503 nm, indicating efficient stabilization of the polarized excited state in polar solvents. The most strongly bathochromically shifted fluorescence maxima are observed for **C-7**, exceeding 508 nm in MeCN and DMSO, which clearly points to a dominant contribution of ICT-type excited states. The persistence of these differences across all solvents demonstrates that substituent effects and the extent of *π*-conjugation play a decisive role in determining emission energies, while the solvent modulates these values through stabilization of the excited state.

In Table S8, the isotropic polarizability (*α*) and the first hyperpolarizability (*β*) are summarized for compounds **C-1**–**C-8** in a series of solvents. Across the entire dataset, a clear monotonic increase in both *α* and *β* is observed with increasing medium polarity; this effect is systematic, yet strongly structure-dependent. The polarizability *α* ranges from 303.85 a.u. (**C-1** in TOL) to 481.67 a.u. (**C-8** in DMSO), and the typical α increments between TOL and DMSO are approximately 60–90 a.u.; e.g., **C-1**: 303.85 a.u. → 367.45 a.u.; **C-8**: 397.09 a.u. → 481.67 au. Even stronger differentiation within the series is provided by the hyperpolarizability *β*. The lowest values are obtained for **C-4** (1952.79 a.u. in TOL and 3437.29 a.u. in DMSO), whereas the highest are found for **C-8** (9745.89 a.u. in TOL and 17,810.46 a.u. in DMSO), corresponding to the largest enhancement of *β* upon moving to a highly polar environment (Δ*β* ≈ 8064.57 a.u.) across the entire series. In every solvent, the same *β* hierarchy is preserved: **C-4** < **C-5** < **C-6** < **C-3** < **C-1** < **C-2** < **C-7** < **C-8,** indicating that the magnitude of the nonlinear response is governed primarily by structural modification, i.e., the extent of conjugation and push–pull character, while the solvent effect acts mainly as an amplifying factor. A characteristic feature is the near-flattening of the solvation effect between MeOH and MeCN, e.g., **C-5**: *β* = 5172.92 a.u. in MeOH and 5172.92 a.u. in MeCN; **C-1**: 7406.70 a.u. vs. 7440.28 a.u., suggesting a comparable efficiency of stabilizing the polarizable component in these two media. When these results are considered alongside the earlier electronic-structure analysis, the highest *β* values for **C-7** and especially **C-8** are consistent with their strongly developed conjugated framework and pronounced donor–acceptor separation. In contrast, the cases of **C-5**–**C-6** show that a large excitation-induced dipole-moment change does not necessarily translate into a maximal *β*; instead, the resonance contribution from delocalization and the effective polarizability of the entire *π*-skeleton remains decisive.

#### 3.2.3. Computational Biointeraction and Toxicity Assessment of **C-1**–**C-8**

Molecular docking of compounds **C-1**–**C-8** with Concanavalin A (ConA) and Human Serum Albumin (HSA) revealed substantial differences in the positioning of ligands within the protein binding pockets, as well as in the intensity and nature of the interactions stabilizing the complexes (Figures S13 and S14 and Tables S9 and S10). In ConA, all ligands reside within a hydrophobic cavity composed mainly of Leu, Phe, and Tyr residues, and their stabilization arises predominantly from van der Waals contacts and aromatic interactions. Only compound **C-5** forms a hydrogen bond, as its donor group adopts a favorable orientation toward a polar residue of the protein. The remaining ligands do not achieve an appropriate donor–acceptor geometry within the ConA pocket. Analysis of the nearest-residue environment shows that members of the C-series interact preferentially with different key basic residues: **C-1** and **C-2** are located near Lys101; **C-5** and **C-6** are near Lys114; and **C-3**, **C-4**, **C-7**, and **C-8** approach Lys116, reflecting subtle differences in steric fit and charge distribution. According to the binding free energies, the most stable complexes are **C-8**–ConA (Δ*G*_b_ = −5.2 kcal/mol) and **C-7**–ConA (−4.6 kcal/mol), indicating that these two ligands best occupy the available pocket volume and make the most effective use of short-range interactions.

In HSA, all ligands localize near the same region of the protein, centered around Cys448, which indicates a strong affinity of the C-series for the FA7/FA3 binding site. FA7 and FA3 constitute major fatty acid binding pockets in HSA and serve as key binding centers for aromatic ligands and numerous drugs, which explains the stable accommodation of the C-series molecules in this region. A substantially greater number of polar contacts is observed here compared to ConA. Hydrogen bonds form only when the geometry of the donor and acceptor atoms becomes favorable after the ligand is fitted into the pocket. Compounds **C-1**, **C-2**, **C-3**, **C-4**, **C-5**, **C-6**, and **C-7** each form at least one such interaction, whereas **C-8**, despite its good steric fit, does not form any hydrogen bonds with HSA. The presence of these interactions, visualized as green dashed lines, is thus a consequence of the local orientation of the functional groups upon binding rather than of intrinsic orbital energies or ground-state charge distribution. The strongest affinity for HSA is exhibited by **C-7** (Δ*G*_b_ = −7.5 kcal/mol), resulting from a combination of favorable steric accommodation and stabilizing hydrogen-bond contacts. Compound **C-8**, although well anchored in the same pocket and rich in hydrophobic interactions, shows a lower affinity (−6.8 kcal/mol) due to the absence of hydrogen bonding. The remaining molecules, particularly **C-2**, **C-3**, and **C-6**, bind more superficially and form fewer electrostatic contacts, yielding less favorable Δ*G*_b_ values.

Comparison of the entire series shows that **C-7** and **C-8** exhibit the highest affinity toward both ConA and HSA, although the mechanisms of stabilization differ: **C-7** benefits from a combination of hydrophobic contacts and hydrogen bonding in HSA, whereas C-8 relies primarily on steric complementarity and van der Waals interactions in both ConA and HSA. These findings highlight that hydrogen bonding is not a universal feature and emerges only at the stage of complex formation, rather than directly from the electronic properties of the molecules. At the same time, the consistency of binding-energy trends with the previously discussed HOMO–LUMO, NBO, and q_CT/DCT_ analyses indicates that the most polarized and spatially separated systems, such as **C-7** and **C-8**, adapt most efficiently to the protein environment and represent the most promising ligands within the entire series.

**Figure 4:**
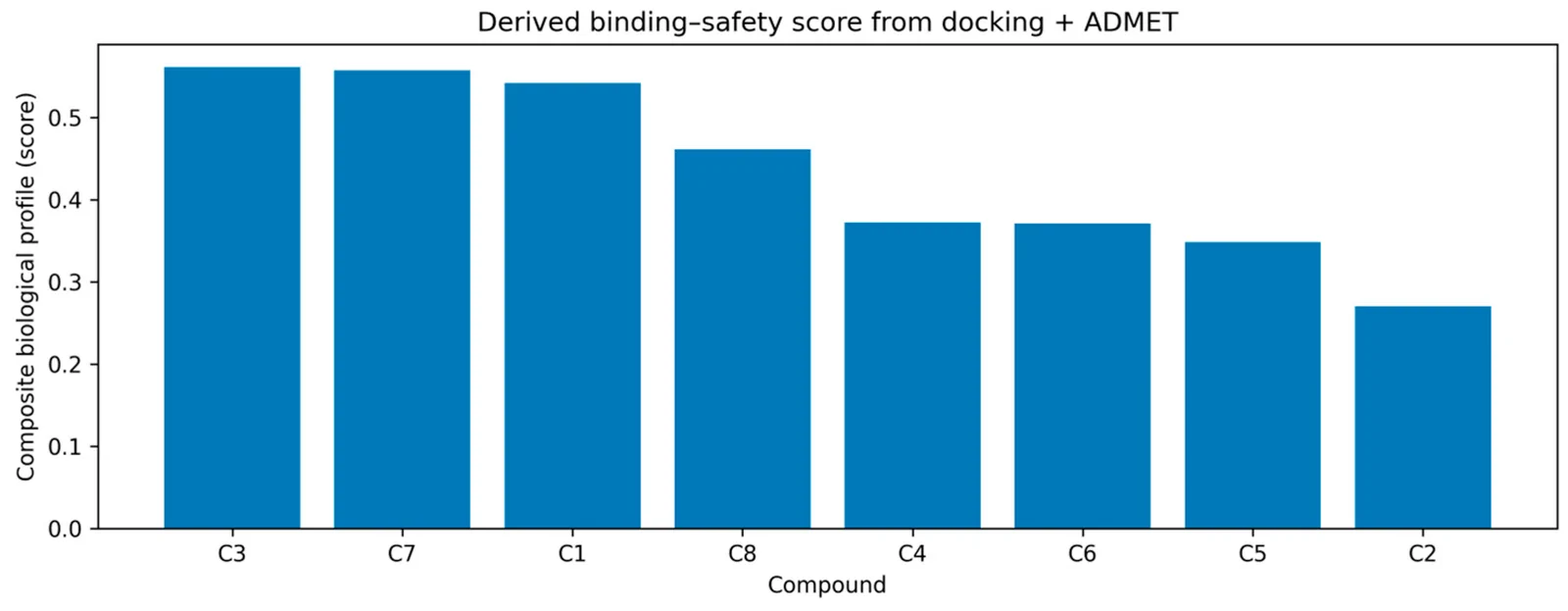
Composite biological profile for compounds **C-1**–**C-8** combining docking-derived affinity (minimum Δ*G*_b_ for ConA and HSA) with predicted safety proxies (logP, logBCF and oral LD_50_). Higher values correspond to a more favorable binding–safety balance.

To further assess the biological applicability of the C-series, in silico physicochemical descriptors related to permeability and bioaccumulation were evaluated. Table S11 summarizes the predicted lipophilicity (logP) and bioaccumulation potential (logBCF) for compounds **C-1**–**C-8**. All molecules fulfill the Lipinski rule of five [40,41], suggesting favorable drug-likeness profiles at the screening stage, exhibiting moderate lipophilicity with logP values between 1.92 and 1.94 for **C-1**–**C-6** and higher values of 3.07 for **C-7** and **C-8**. The logBCF values fall within 0.397–0.887, indicating a low risk of bioaccumulation in biological tissues. This overall docking–ADMET biological profile is summarized in Figure 4. Since none of the compounds exceeds Lipinski’s threshold parameters, the entire series is expected to possess good bioavailability and minimal tendency for excessive lipid accumulation. These parameters are consistent with the previously discussed HOMO–LUMO and MEP analyses, in which the more polarized systems (**C-7** and **C-8**) also exhibited increased lipophilicity, influencing their behavior in biological environments. Notably, these descriptors are predictive in nature and should be interpreted as supportive evidence that requires experimental validation.

The predicted LD_50_ values for different administration routes further confirm the low acute toxicity of the **C**-series (Table S12). For oral administration, LD_50_ values range from 1253 to 2475 mg/kg, classifying the compounds as low-toxicity substances. The lowest value is predicted for **C-2** (1253 mg/kg), whereas **C-1** and **C-3** show the highest tolerability (>2400 mg/kg). As expected, LD_50_ values for intravenous, subcutaneous and intraperitoneal routes are lower than oral values, yet all remain within ranges indicative of moderately low acute toxicity.

The ADMET profile [42] presented in Table S13 demonstrates favorable metabolic properties for the entire series. Compounds **C-1**–**C-8** are predicted to be inactive inhibitors of the major cytochrome P450 isoforms, including CYP1A2, CYP2C19, CYP2D6, CYP3A4 and CYP2E1. Only **C-7** and **C-8** show moderate predicted activity toward CYP2C9 (*p* = 0.60), which may be linked to their more extended aromatic systems and slightly higher logP. Regarding organ toxicity, all compounds show similar trends: nephrotoxicity and respiratory toxicity are predicted as active with moderate probability, while hepatotoxicity, neurotoxicity and cardiotoxicity are generally predicted as inactive or low. These findings suggest that any potential adverse effects would most likely relate to organs sensitive to metabolic load, which is typical for small aromatic molecules.

In the category of end-point toxicity, all compounds are predicted to be non-mutagenic, non-cytotoxic and non-carcinogenic, indicating no genotoxic risk. Immunotoxicity is predicted only for **C-5** (*p* = 0.65), although this remains a single, moderate signal. All compounds are predicted to cross the blood–brain barrier, which, in combination with their pronounced ICT character and high *q*_CT_ values, may be advantageous for applications involving fluorescence imaging of neuronal structures.

Taken together, the logP/logBCF values, LD_50_ predictions and ADMET parameters indicate that the **C**-series exhibits a favorable and consistent safety profile. The absence of significant hepatotoxicity, the low potential for metabolic interactions, and the lack of mutagenic or carcinogenic properties demonstrate that these molecules constitute stable and safe candidates for bioimaging applications. These results complement the earlier photophysical and docking analyses, further supporting the suitability of **C-1**–**C-8** for future biological studies.

### 3.3. Luminescence Studies

**Figure 5:**
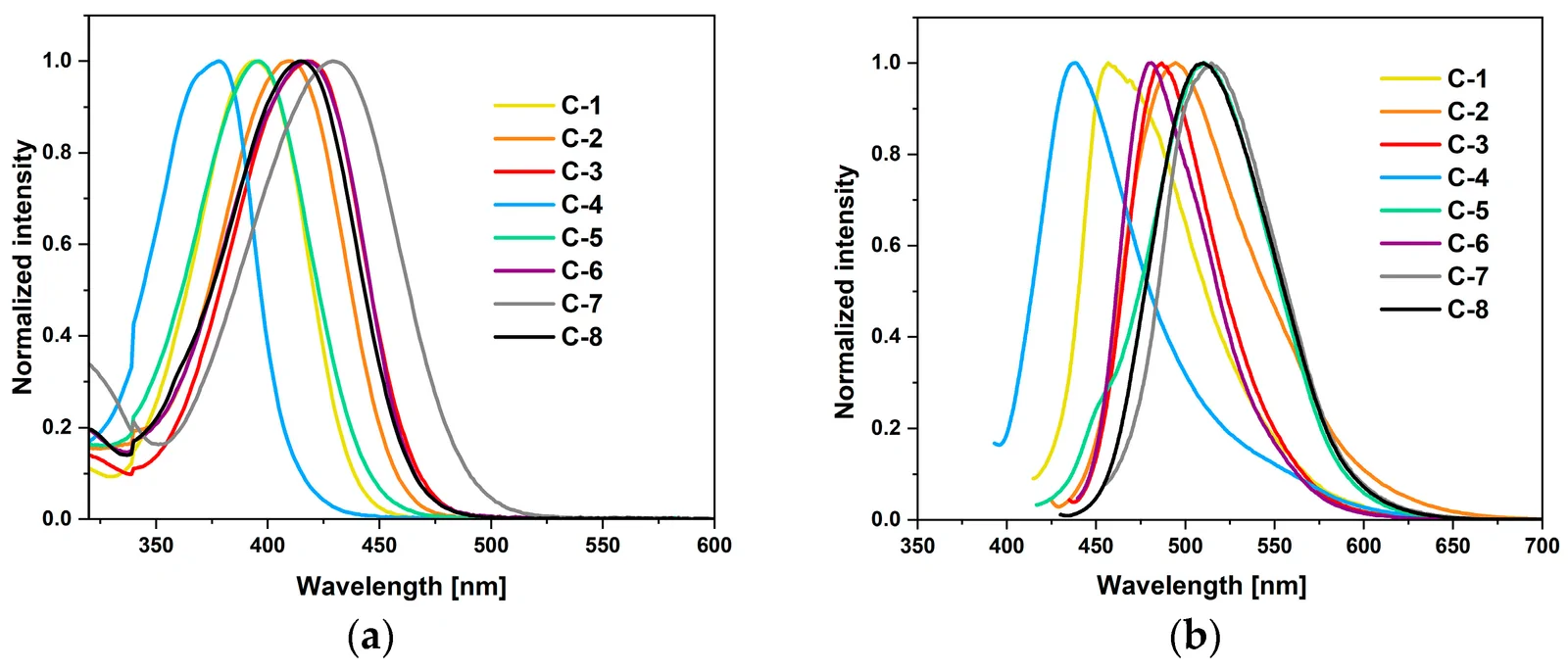
Comparison of (**a**) absorption and (**b**) emission measurement results for the C-series in DMSO.

To determine the effect of methoxy groups on rhodanine-3-acetic acid derivatives (**C-1**–**C-8**), luminescence studies were performed. Six solvents with different dielectric constants were selected for this purpose. This enabled the evaluation of the optical properties of the studied compounds across different measurement media. A concentration of 2.5 × 10^−5^ mol/L was used for the studies. Spectral recording began with UV-Vis measurements. All derivatives exhibited a single distinct absorption peak in the ultraviolet range (Table S14). This band arises from intramolecular charge transfer (ICT) between the substituent and rhodanine-3-acetic acid [43]. This is consistent with the literature reports of compounds with a similar structure [43,44,45,46]. All **C-1**–**C-8** derivatives showed high molar absorption in the range ε = 5.5 × 10^4^–2.3 × 10^4^ M^−1^ ∙ cm^−1^, reflecting the efficient ICT facilitated by the acceptor rhodanine-3-acetic acid ring [43]. The **C-4** compound showed the greatest hypsochromic shift, while the **C-7** derivative showed a bathochromic shift (Table S15). Regardless of the measurement medium used, the results from absorption spectroscopy showed minimal dependence on solvent effects. Therefore, the ground-state dipole moment characteristic does not change with the change in the polarity of the solvent used [47]. To determine the influence of methoxy groups in the presented series, it is worth examining derivatives in the **C-2**–**C-4**, **C-5**–**C-6**, and **C-7**–**C-8** groups. The first group (**C-2**–**C-4**) has a phenyl substituent with two methoxy groups. Substitution at the 3,5-position (**C-4**) causes a hypsochromic shift, which may be related to the negative inductive properties of this group at the meta position [48]. Furthermore, theoretical calculations indicate that this compound exhibits the weakest effective donor–acceptor coupling within this structure. Moreover, it shows relatively low and stable ΔμCT-GS values (ranging from 3.32 D in toluene to approximately 4.40 D in DMSO), indicating limited charge-density redistribution upon excitation. This is consistent with the literature reports [49]. However, if we rearrange one methoxy group from position 5 to position 4 (**C-2**), thereby bringing the two methoxy groups closer together, we observe a bathochromic shift of more than 25 nm. This may be due to the mesomeric effect of the paramethoxy group, which stabilizes the excited state more effectively than -OCH_3_ in the meta position [50]. Changing the position of the methoxy group to 2,4 (**C-3**) causes a further red shift of absorption, but it is not as large as in the previous case (about 10 nm in DMSO) (Figure 5). If we consider compounds with three methoxy groups, we can see that **C-5**, having the considered moiety in the 3,4,5 position, has the same absorption maximum as compound **C-1** (with –OCH_3_ in position 4) (Table 2). This indicates no influence of the substituents in positions 3 and 5. In turn, **C-6** (substitution of the phenyl ring—2,4,6) is significantly more bathochorically shifted than **C-5**. Furthermore, the absorption maximum for **C-6** overlaps with that of **C-3**. This shows that introducing an additional methoxy group at position 6 does not increase the mesomeric effect. As expected, changing the phenyl substituent to a naphthalene substituent allowed for the acquisition of absorption spectra above 400 nm. Interestingly, however, the arrangement of the naphthalene substituent and the position of the attached –OCH_3_ group are crucial. As shown in Table S15, the attachment of the methoxy group at the 4-position of naphthalene (**C-7**) results in a distinct red shift relative to **C-8** (position 6). By varying the substitution of the methoxy group, we can tune the *π*-conjugation and effectively enable modulation of the light absorption capacity [51]. Therefore, the optical band gap was determined for all derivatives, taking the onset of the absorption peak as the starting point. This gap ranged from 2.50 to 3.02 eV. These relatively narrow band gaps can be attributed to the *p-π* resonance, which can increase the degree of coupling and improve electron delocalization [51].

In bioimaging studies, an important factor determining the usefulness of compounds is their stability in solution for as long as possible. For the C-series, spectra were recorded in DMSO at room temperature over a 24-h period. The collected results (Table S16) clearly indicate that all derivatives are stable in the measurement environment, as their absorption profile did not change during the measurements. This allowed all molecules to be qualified for further application studies. Emission measurements were performed based on the obtained absorption spectra. To avoid excimer and concentration effects, dilute solutions (2.5 × 10^−5^ mol/L) were used for the measurements [52]. Broad spectra with very low peak intensities were recorded for the entire series of **C-1**–**C-8** compounds. Their fluorescence peaks in solution occur in the wavelength range from 435 to 515 nm (Table S17). Furthermore, these molecules are characterized by very low quantum yields (Table 2). The weak fluorescence and low yield of the discussed derivatives result from intramolecular charge transfer (ICT) [52]. As with the absorption spectra, the methoxy groups influenced the obtained results. For the **C-4** derivative, results could not be obtained in acetonitrile or methanol. Moreover, the aforementioned compound exhibits the most blue-shifted emission maximum. Calculations confirmed that compound **C-4** exhibits the shortest emission wavelengths in the entire series, confirming its character as being primarily associated with local excitation and aligning fully with the previously discussed low Δμ_CT-GS_ values and weak solvatochromic sensitivity. The largest bathochromic shifts were observed for compounds **C-7** and **C-8**. Comparing the derivatives across the entire group, we can see that the spectra obtained in MeOH were hypsochromically shifted. This may be due to the protic nature of this solvent, which allows for the formation of hydrogen bonds with the molecule of the considered compound. For the C-series, a solvatochromic study was performed based on the shift of the UV–visible and fluorescence absorption maxima with varying solvent polarity [52]. The Stokes shift (Δv) calculated based on the absorption and emission maxima is presented in Table 2. Using the knowledge of the values of this shift, changes in dipole moments were determined using the Lippert–Mataga equation [53] (Table S18). The changes in dipole moments for C-series molecules range from 6.33 D to 7.15 D.

### 3.4. Bioimaging

#### 3.4.1. Preliminary Cytotoxicity

**Figure 6:**
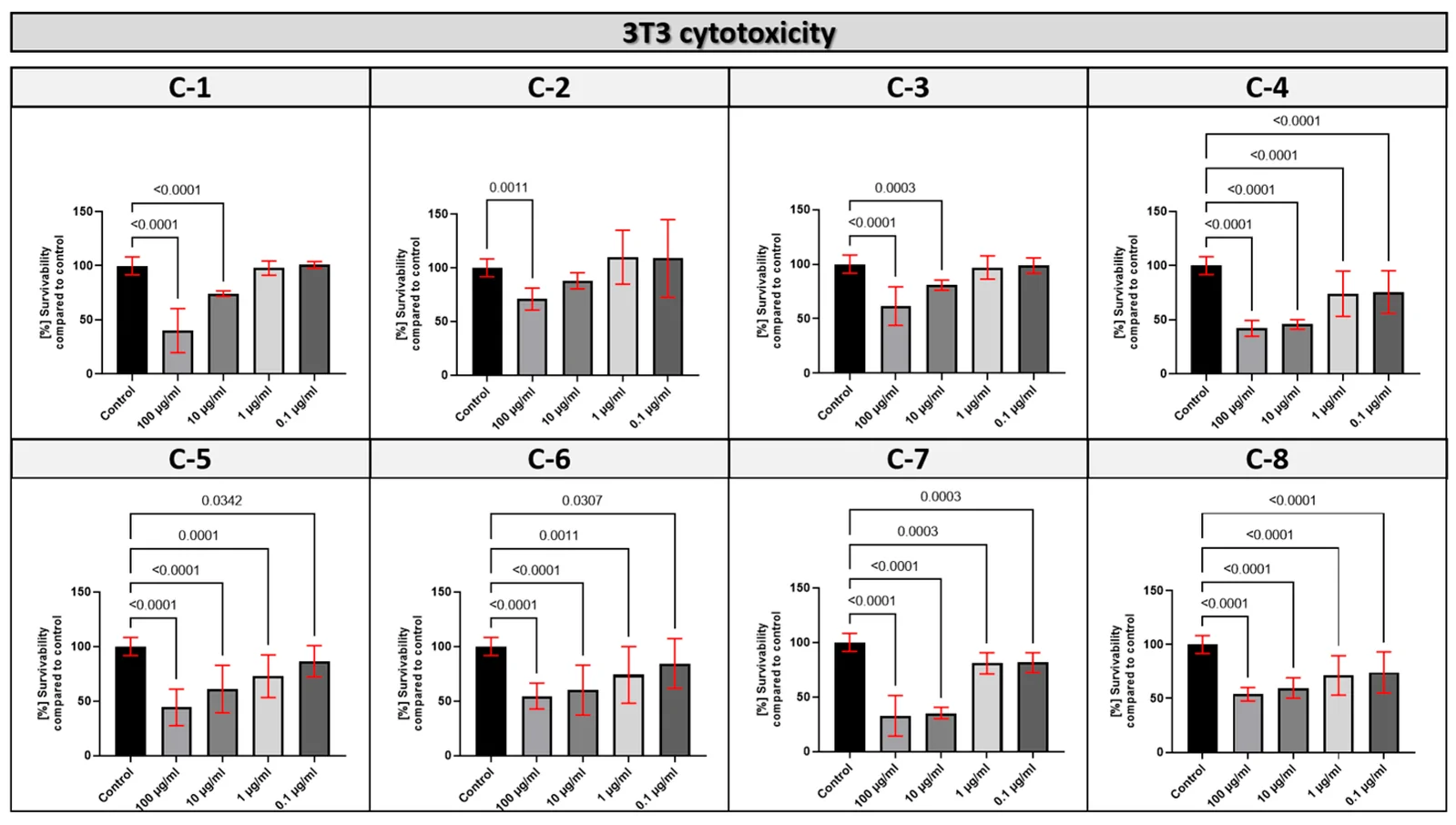
MTT results of 3T3 cells after 24-h exposure to tested compounds (**C-1**–**C-8**). Results are shown as averages and standard deviations on graphs. Statistical analysis results overlaid as comparison brackets. ANOVA with Dunnett’s post hoc analysis performed with Cl 95%.

Compounds **C-4**, **C-5**, **C-6**, **C-7**, and **C-8** induce a statistically significant reduction in cell survival compared to the control group at every tested concentration, from 100 μg/mL down to 0.1 μg/mL (Figure 6). Compounds **C-4**, **C-7**, and **C-8** maintain a relatively high level of statistical significance across the entire dosage spectrum (with viability dropping at 100 μg/mL to 42.00%, 32.73%, and 53.89%, respectively). While **C-5** and **C-6** follow a similarly widespread cytotoxic trend, their statistical significance is slightly lower at the 0.1 μg/mL concentration (where cells retain 86.52% and 84.42% survival). Conversely, compounds **C-1**, **C-2**, and **C-3** exhibit lower cytotoxicity at lower concentrations; 1 μg/mL and 0.1 μg/mL remain statistically indistinguishable from the untreated control group (maintaining strong viability ranges between 96.99% and 109.89%). Compound **C-1** and **C-3** only begin showing significant toxicity when using 10 μg/mL and above (dropping to 74.25% and 80.77% at the 10 μg/mL threshold). Most notably, compound **C-2** establishes itself as the least cytotoxic, demonstrating a significant drop in viability exclusively at the maximum dose of 100 μg/mL (to 70.96%) (Figure 6).

#### 3.4.2. Imaging of Fixed Cells

**Figure 7:**
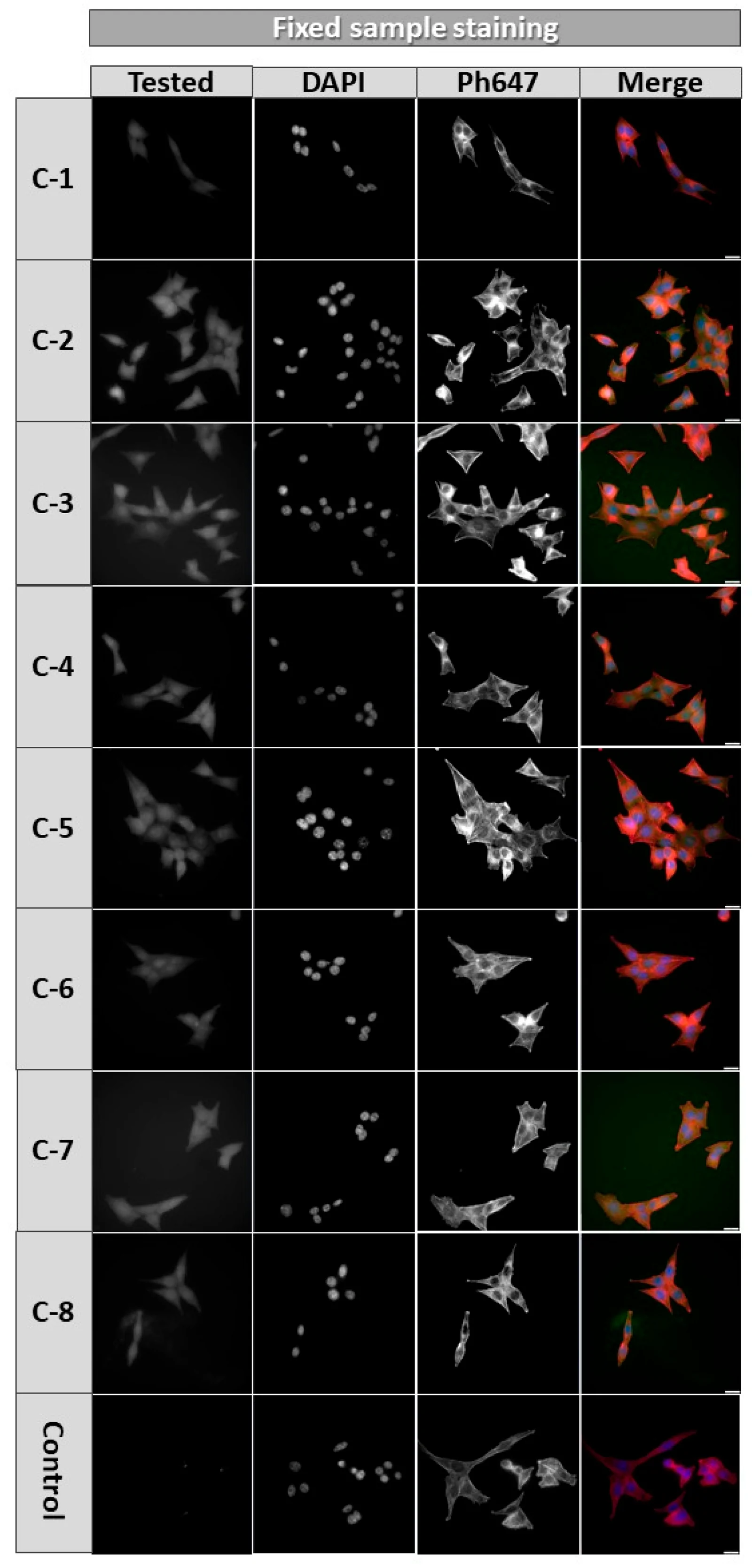
Cell imaging after fixation and staining with the tested compounds (**C-1**–**C-8**). Phalloidin iphluor647 and DAPI staining were used as a contrast. Images are presented as grayscale images, with a merged image with additional pseudocolor provided. The scalebar visible on the Merge image represents 20 μm. Imaging performed on IX83 microscope (Olympus, Tokyo, Japan).

In the fixed-sample staining test, the compounds differ in their specific signal intensity and background noise, as we have observed. Compounds **C-2** and **C-5** yield the best signal characteristics, delivering a high fluorescence signal combined with relatively low background noise to achieve acceptable contrast in the FITC/Alexa488 channel (Figure 7). Compounds **C-4** and **C-6** provide moderate contrast by pairing a medium fluorescence signal with relatively low background levels. Conversely, compounds **C-3** and **C-7** suffer from high background noise, while **C-1** and **C-8** offer overall poor imaging performance due to poor specific target retention. In all cases, the stain was detected in the cytoplasmic region of the cells. Using other stains, we also have confidence that the tested compounds did not specifically stain the nucleus or the F-Actin filaments. We have also determined that all tested compounds were compatible with DAPI and phalloidin iFluor647, as their brightness and background noise were identical to those of the control that was not stained with the tested compounds.

#### 3.4.3. Cell Imaging After Compound Exposure

**Figure 8:**
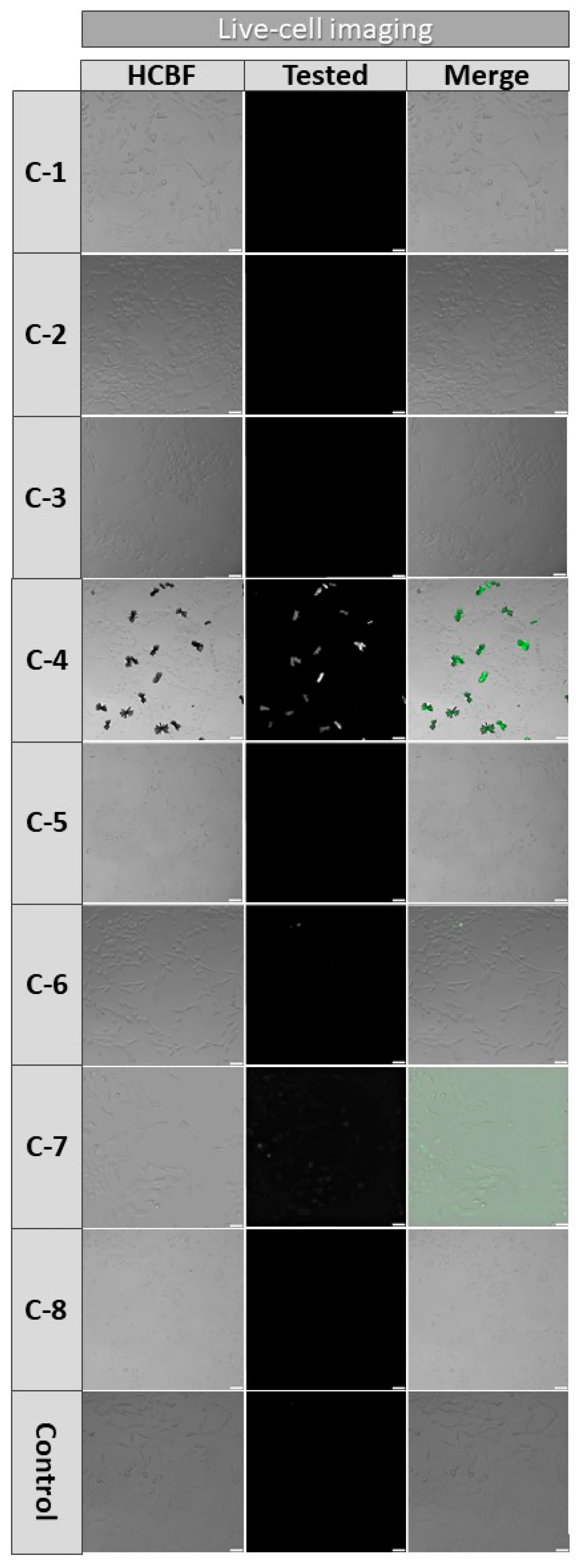
Cell imaging with HCBF (High Contrast Brightfield) and in the FITC/Alexa488 channel after 24 h of exposure to 10 μg/mL of tested compounds. The scalebar visible on the Merge image represents 50 μm. Imaging performed on IX83 microscope (Olympus, Japan).

Evaluation of imaging after 24 h of exposure to 10 μg/mL concentrations of tested compounds was based on fluorescence in the FITC/Alexa488 channel, overlaid with the HCBF channel. The results show that only **C-7** exhibited discernible fluorescence (Figure 8). All tested compounds caused very high background noise before the PBS wash, and **C-7** still exhibited some background after the single wash. Compound **C-4** also caused visible crystal formation in the tested wells. These exhibited fluorescence in the FITC/Alexa488 channel. Severe morphological changes were visible after exposure to compounds **C-4**, **C-5**, and **C-8** (Figure 8).

### 3.5. Antimicrobial Studies

The antimicrobial activity of the newly synthesized compounds **C-1**–**C-8** was tested against 20 reference strains of microorganisms: Gram-positive (eight strains) and Gram-negative (five strains) bacteria, and fungi belonging to *Candida* spp. (seven strains).

As presented in Table 3, these compounds showed some antimicrobial effect, especially towards Gram-positive bacteria. The highest activity was exhibited by compound **C-1** against all cocci and bacilli, with MIC in the range 250–1000 µg/mL and MBC ≥ 2000 µg/mL. This substance indicated a mild effect towards *Staphylococcus aureus* MRSA and *Staphylococcus epidermidis* (MIC = 1000 µg/mL) and a moderate effect against other Gram-positive bacteria (MIC = 250–500 µg/mL). In the case of compound **C-6**, antibacterial activity was shown towards *Enterococcus faecalis* (MIC = 1000 µg/mL), *Micrococcus luteus* and both strains of *Bacillus* spp. (MIC = 500 µg/mL and MBC = 1000–2000 µg/mL). Moreover, *E. faecalis* was sensitive to substances **C-3**, **C-4**, **C-7** and **C-8**. These compounds inhibited the growth of enterococci at MIC = 1000 µg/mL. In turn, *M. luteus* revealed sensitivity to substances **C-4** (MIC = 1000 µg/mL) and **C-7** (MIC = 500 µg/mL). Compound **C-8** also indicated an antibacterial effect against *B. subtilis* (MIC = 1000 µg/mL) and *B. cereus* (MIC = 500 µg/mL). The remaining Gram-positive bacteria were insensitive to the tested compounds (MIC ≥ 2000 µg/mL).

The microorganisms belonging to Gram-negative rods were also insensitive to these substances. The minimal inhibitory concentration values of the studied compounds were 2000 µg/mL or >2000 µg/mL, which is considered no antimicrobial activity. In the case of yeasts from *Candida* species, the results were similar. The tested compounds showed no activity against them. The exception was one substance, namely compound **C-1**, which indicated mild bioactivity with MIC = 1000 µg/mL and MFC = 2000 µg/mL (MFC/MIC = 2) towards two strains: *C. parapsilosis* and *C. lusitaniae* (Table 3).

### 3.6. Anticancer Activity

#### 3.6.1. Anticancer Properties of Compounds **C-1**, **C-2**, **C-3**, **C-4**, **C-5**, **C-6**, **C-7** and **C-8**

Stage I of the studies on the assessment of the anticancer properties of the tested compounds was carried out on various cancer cell lines (SNB-19, C32, Caco-2, MDA-MB-231, A2058, Colo-829, LoVo/DX, Panc-1, Skov3, HT29, A549, SH-4) and the normal fibroblast line HFF-1. In cultures exposed to the tested compounds **C-1**, **C-2**, **C-3**, **C-4**, **C-5**, **C-6**, **C-7** and **C-8** (at concentrations: 0.1–100 µg/mL), the following were determined: the relative number of live cells (WST-1 test), the relative number of dead cells (LDH test) and the relative total number of cells (crystal violet staining). Absorbance values obtained in the WST-1 test were used to determine the LC50 (the concentration of a substance at which half the members of a population are killed after a specified duration of exposure) using the Bioquest AAT LC50 calculator. Based on the obtained results, one compound and one cell line were selected for further study.

In the II stage of the study, the proliferation and migration capabilities of cells from a selected line were evaluated in response to one of the compounds, and the type of cell death occurring was determined. Proliferative and migratory capacities were assessed using a wound healing assay, which measures the rate of cell migration to occupy available growth space under specific culture conditions. Furthermore, the type of cell death occurring in the culture was analyzed using fluorescent staining to distinguish between apoptotic and necrotic cells: DAPI staining (blue light emission), fluorescein-conjugated Annexin V staining (green light emission), and propidium iodide staining (red light emission). Subsequently, the molecular basis of the selected compound’s action was evaluated. The presence of specific proteins associated with cellular stress was determined in cancer cells exposed to the compound (at a concentration of 10 µg/mL) for 24 h. The Proteome Profiler Array Human Cell Stress Array Kit ARY 018 (R&D Systems) was used for the analysis, enabling the determination of changes in the levels of the following proteins: ADAMTS1, Bcl-2, Carbonic Anhydrase IX, Cited-2, COX-2, cytochrome c, Dkk-4, FABP-1, HIF-1α, HIF-2α, phospho-HSP27 (S78/S82), HSP60, HSP70, IDO, phospho-JNK Pan (T183/Y185), NFκB1, p21/CIP1, p27, phospho-p38α (T180/Y182), phospho-p53 (S46), PON1, PON2, PON3, thioredoxin-1, SIRT2, and SOD2.

#### 3.6.2. Effect of Compounds **C-1** to **C-8** on Cancer Cells

**Stage I**

The anticancer properties of compounds **C-1**, **C-2**, **C-3**, **C-4**, **C-5**, **C-6**, **C-7**, and **C-8** were subjected to preliminary analysis using cultures of the following cancer cell lines: SNB-19, C32, Caco-2, MDA-MB-231, A2058, Colo-829, LoVo/DX, Panc-1, Skov3, HT29, A549, and SH-4, as well as normal HFF-1 cells.

**Cell viability**

The first stage of the study involved determining the relative number of viable cells in cultures treated with the test substances using the WST-1 assay (Roche Diagnostics). Based on absorbance measurements reflecting cellular metabolic activity, LC50 values for the tested compounds were determined for each cell line. The AAT Bioquest LC50 calculator was used for this purpose (Table 4).

The calculated LC50 values for the SNB-19, C32, Caco-2, MDA-MB-231, A2058, Co-lo-829, LoVo/DX, Panc-1, Skov3, HT29, A549, SH-4, and HFF-1 cell lines indicate very limited cytotoxic activity of the tested compounds. After a 72-h incubation of cells with compounds **C-2**, **C-6** and **C-7**, no effect of these derivatives on cell numbers was observed within the tested concentration range. The remaining compounds exhibit selective activity against specific cell lines. Compound **C-1** affects the functioning of C32 and A2058 cells; **C-3** acts on the A2058 line; **C-4** on C32 and A549; **C-5** on C32, MDA-MB-231, and A2058; and **C-8** on SNB-19, Caco-2, and A2058. No effect of the tested compounds (at concentrations up to 100 µg/mL) was observed on normal skin fibroblasts (HFF-1 line) or on the cancer cell lines Colo-829, LoVo/DX, Panc, Skov3, HT29, and SH-4. Compounds **C-5** and **C-8** induce the most changes detected by the WST-1 assay, and the melanoma lines A2058 and C32 show the greatest sensitivity to the tested compounds (in the WST-1 assay). However, with melanoma cells, the authors observed—as noted in numerous other studies—low assay reproducibility and frequent result discrepancies, most likely stemming from melanin synthesis and presence. To eliminate these obstacles, the SNB-19 cell line was selected for subsequent stages of the study. The choice of the compound for further testing was also significantly influenced by the results of other assays (LDH and crystal violet staining). While each of these is useful for assessing cell numbers in cultures, only a combined analysis of the data provides a comprehensive picture of the changes intended to inhibit tumor growth.

**Figure 9:**
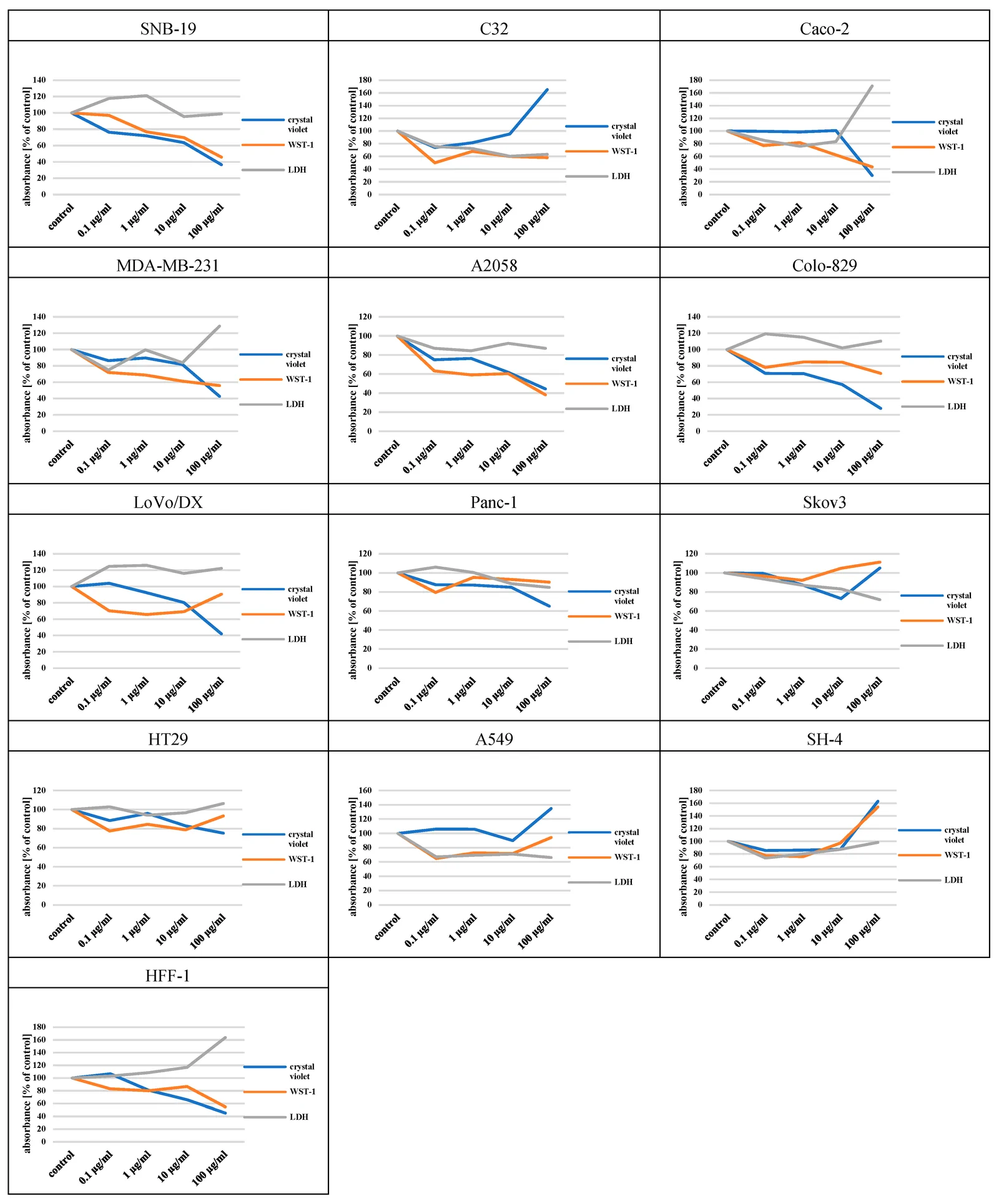
Relative number of live cells in cultures (WST-1), relative number of dead cells in cultures (LDH), and relative total number of cells in cultures (crystal violet staining) in cultures exposed to compound **C-8** for 72 h. All experiments were conducted in at least three replicates, enabling a statistical assessment of the obtained measurement results. The data distributions for each sample met the assumption of normality (verified using the Shapiro–Wilk test), justifying the use of parametric tests for statistical analysis. The control group was compared against the four study groups using Dunnett’s test, following the confirmation of statistically significant differences among the analyzed means (via the F-test in the analysis of variance). Assumptions of variance homogeneity were checked with use of Levene’s test and the Brown–Forsythe test (Table 5). All tests were performed at a significance level of α = 0.05 using Statistica 13.3 software.

The cytotoxicity of the tested compounds (measured by the number of dead cells) was evaluated by quantifying lactate dehydrogenase (LDH) released from dead cells into the culture medium. Cultured cells were also stained with crystal violet, which binds to cellular DNA; absorbance measurements were then used to determine the relative total cell count. For all three assays—WST-1, LDH, and crystal violet staining—absorbance was measured after a 72-h exposure of the cells to the test compounds. Based on the results regarding the relative number of viable cells (WST-1), dead cells (LDH), and total cells (crystal violet staining) following exposure, compound **C-8** was selected for further study. Compound **C-8** affects the largest number of cell lines at relatively low concentrations; for SNB-19 cells, significant changes were observed not only in the WST-1 assay but also—at low **C-8** concentrations—in the LDH and crystal violet staining assays. These results clearly indicate a reduction in cell numbers in the cultures (resulting from the inhibition of proliferation and/or cell death) rather than merely metabolic changes measured by the WST-1 assay (which could stem from either a decrease in cell number or reduced metabolic activity). Figure 9 shows the measurement results obtained from WST-1 and LDH assays and crystal violet staining after 72 h of cell exposure to compound **C-8**.

**Table 5:** Statistical probabilities for the results of ANOVA and Dunnett’s test for SNB-19 cell cultures incubated with compound **C-8** (*p* ≤ 0.05). For ANOVA *p*-values > 0.05, there were no grounds to perform Dunnett’s test (shaded cells).

	SNB-19		C-32		Caco-2		MDA-MB-231		A2058		Colo-829		LoVo/DX		Panc-1		Skov3		HT29		A549		SH-4		HFF-1	
	ANOVA	Dunnett’s Test	ANOVA	Dunnett’s Test	ANOVA	Dunnett’s Test	ANOVA	Dunnett’s Test	ANOVA	Dunnett’s Test	ANOVA	Dunnett’s Test	ANOVA	Dunnett’s Test	ANOVA	Dunnett’s Test	ANOVA	Dunnett’s Test	ANOVA	Dunnett’s Test	ANOVA	Dunnett’s Test	ANOVA	Dunnett’s Test	ANOVA	Dunnett’s Test
**crystal violet**																										
**0.1 μg/mL**	**<0.00001**	**0.00065**	**0.03956**	0.72654	**0.00012**	0.08049	**0.00155**	0.47003	**0.00045**	0.02982	**<0.00001**	**0.00132**	0.10104		0.10554		0.38556		**0.03512**	0.34306	**0.00622**	0.89949	**0.01979**	0.9018	**0.02709**	0.98279
**1 μg/mL**		**0.00017**		0.88649		0.09723		0.68231		0.03914		**0.00121**								0.93745		0.91065		0.91331		0.63321
**10 μg/mL**		**0.00002**		0.99912		**0.00207**		0.24722		0.0021		**0.00007**								0.10479		0.64366		0.93412		0.20451
**100 μg/mL**		**0.00001**		0.09443		**0.00004**		**0.00055**		0.00012		**0.00001**								**0.01827**		**0.01018**		**0.04576**		**0.02948**
**WST-1**																										
**0.1 μg/mL**	**0.00001**	0.92704	**0.00347**	**0.00139**	0.11692		**0.00003**	**0.00047**	**0.00007**	**0.00103**	0.56909		**0.00056**	**0.00183**	0.49056		0.25669		**0.04684**	**0.03487**	**0.00382**	**0.00368**	**0.00002**	**0.07456**	**0.00009**	**0.02729**
**1 μg/mL**		**0.00348**		**0.02467**				**0.00021**		**0.00045**				**0.00064**						0.16632		**0.01743**		**0.05605**		**0.0107**
**10 μg/mL**		**0.00048**		**0.00651**				**0.00004**		**0.00059**				**0.00142**						**0.04572**		**0.01428**		0.99272		**0.08207**
**100 μg/mL**		**0.00001**		**0.0048**				**0.00002**		**0.00002**				0.37463						0.75985		0.86257		**0.00029**		**0.00003**
**LDH**																										
**0.1 μg/mL**	**0.00261**	**0.03536**	**0.00096**	**0.0131**	**0.00007**	0.56639	**0.0001**	**0.01121**	0.2915		0.28112		**0.04745**	**0.03648**	**0.01882**	0.66702	**0.01**	0.70148	0.16139		**0.01848**	**0.01494**	**0.00038**	**0.00036**	**0.00003**	0.98197
**1 μg/mL**		**0.01252**		**0.00609**		0.20502		0.99999						**0.02775**		0.99999		0.17576				**0.02242**		**0.0028**		0.61219
**10 μg/mL**		0.83678		**0.00043**		0.4669		0.10225						0.20485		0.20707		**0.06399**				**0.03038**		**0.04287**		0.12467
**100 μg/mL**		0.99781		**0.0008**		**0.00048**		**0.00463**						**0.05998**		**0.07122**		**0.00354**				**0.01286**		0.97772		**0.00002**

**Stage II**

**Wound healing assay**

**Figure 10:**
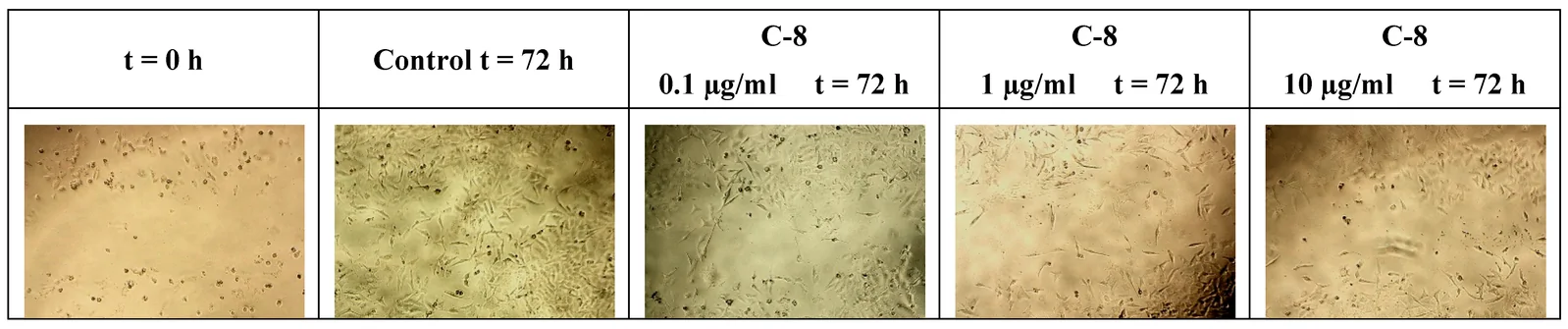
Wound healing assay: cultures of SNB-19 exposed to **C-8** (0.1–10 µg/mL) at t = 0 and t = 72 h.

During the first stage of assessing the anticancer properties of compound **C-8**, a scratch healing test was also performed. Figure 10 shows images of cell cultures exposed to compound **C-8** at concentrations of 0.1–10 µg/mL, taken at times t = 0 and t = 72 h. The rate of cell-free area overgrowth depends on the proliferative activity of the cells in the culture and the toxic effects of the compound on the cells, as well as the ability of the cells to migrate under the given culture conditions.

Images of SNB-19 cell cultures (control and exposed to **C-8** at concentrations of 0.1–10 µg/mL) were taken immediately after scratching and then after 72 h of incubation (Figure 10). Analysis of cell culture images 72 h after scratching indicates a reduction in the proliferative activity and migratory capacity of cells exposed to **C-8** within the tested concentration range. In the control culture, the surface of the culture vessel at the scratch site (initially devoid of cells) was overgrown with cells (only a small number of empty spaces are visible), whereas in cultures supplemented with medium containing **C-8** at concentrations of 0.1–10 µg/mL, extensive cell-free areas were visible.

**DAPI staining**

**Figure 11:**
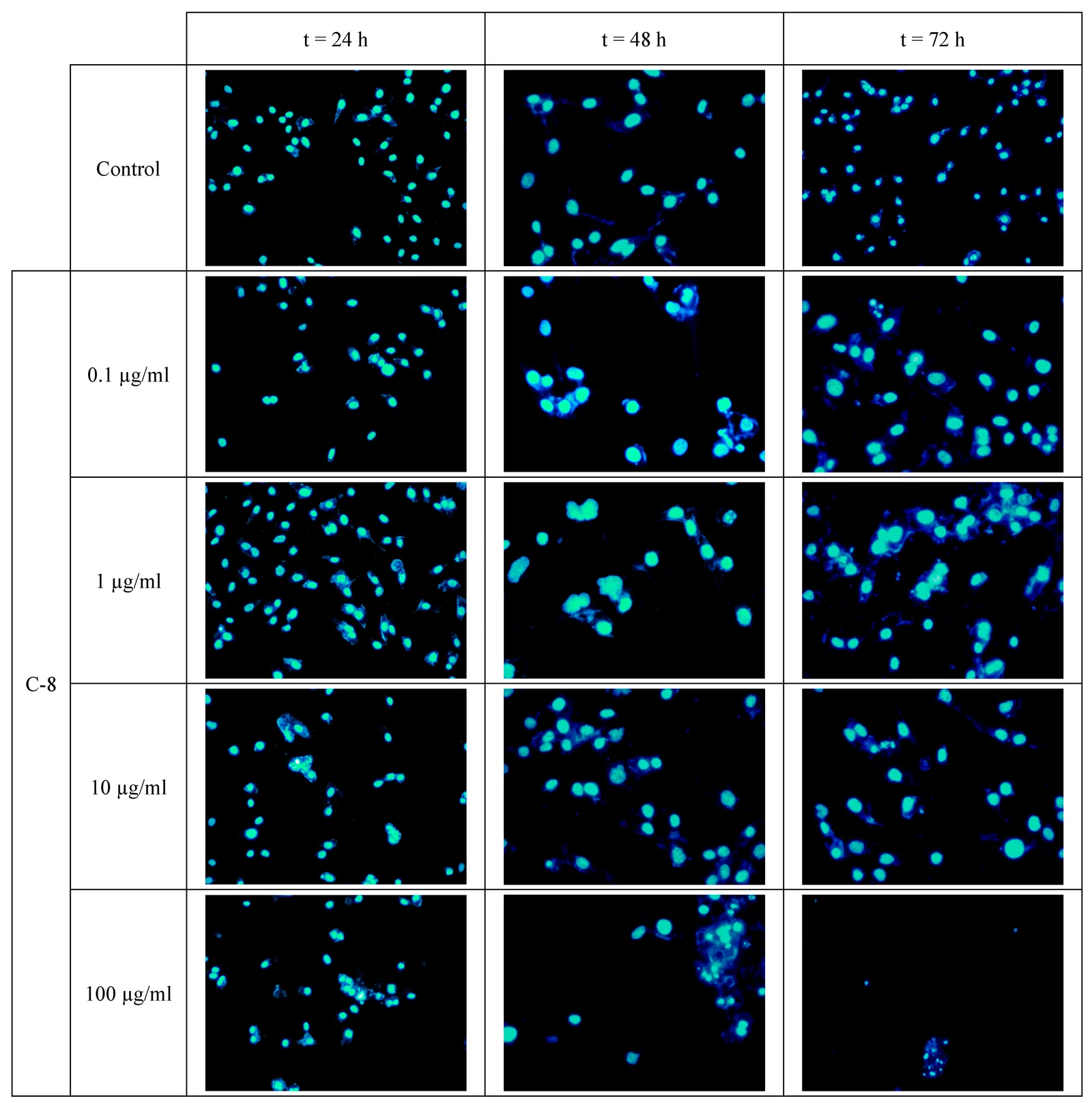
DAPI staining: cultures of SNB-19 exposed to **C-8** (0.1–100 µg/mL) at t = 24, t = 48 h and t = 72 h.

4′,6-diamidino-2-phenylindole (DAPI) is a fluorescent dye that binds strongly to adenine-thymine-rich regions of DNA. It emits characteristic blue fluorescence, enabling the visualization of cell nuclei. DAPI staining allows the identification of apoptotic cells, which are characterized by nuclear morphology different from normal cells, i.e., condensed chromatin and nuclear fragmentation. Figure 11 shows images of DAPI-stained cell cultures exposed to **C-8** at concentrations of 0.1–100 µg/mL, taken at t = 24 h, t = 48 h, and t = 72 h.

Pictures of SNB-19 cell cultures stained with DAPI (control and exposed to **C-8** at concentrations of 0.1–100 µg/mL) were taken after 24, 48 and 72 h of incubation (Figure 11). In the examined cell cultures subjected to 24, 48 and 72 h of incubation with **C-8** at concentrations of 1–100 µg/mL, few apoptotic cells were identified. In the cultures incubated with **C-8** at a concentration of 0.1 µg/mL, only single apoptotic cells were observed, and the staining result was comparable to that of the control cultures.

#### 3.6.3. Annexin V/Propidium Iodide (PI) Staining

**Figure 12:**
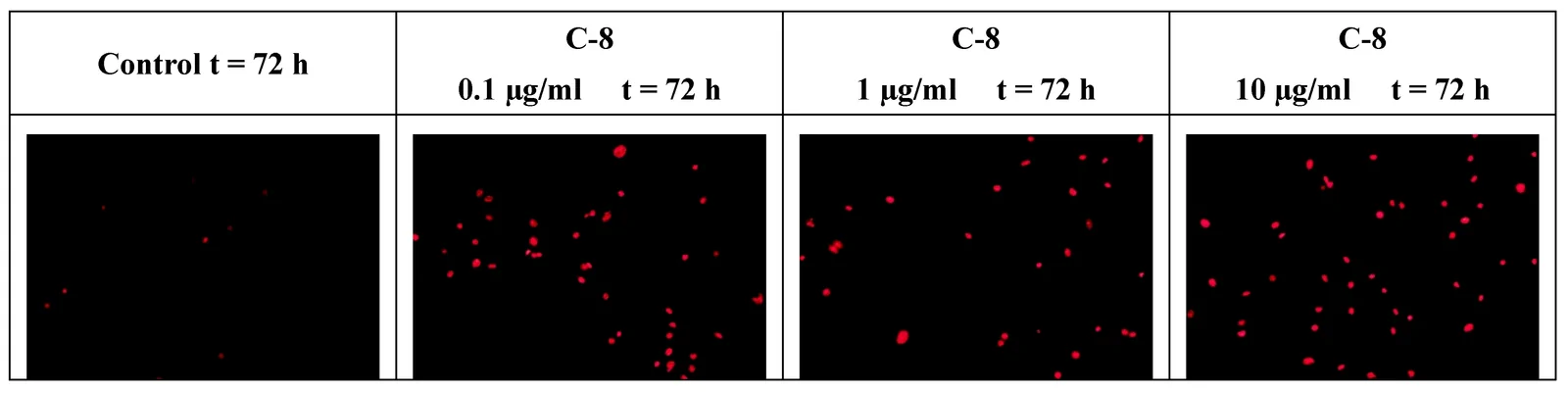
Annexin V/propidium iodide (PI) staining: SNB-19 cultures exposed to **C-8** (0.1–10 µg/mL) at t = 72 h.

Annexin V is a calcium-dependent protein with high affinity for phosphatidylserine, a phospholipid typically located in the inner layer of the cell membrane. During early apoptosis, phosphatidylserine translocates to the outer layer of the cell membrane. Annexin V labeled with the fluorochrome fluorescein (emitting green light) can then bind to phosphatidylserine, thus acting as a marker for cells undergoing early apoptosis. Propidium iodide (PI), in turn, is a marker of late apoptosis and necrosis that intercalates into nucleic acids. It penetrates cells with compromised membrane integrity and binds to DNA, emitting red light. Figure 12 shows images of cell cultures stained with annexin and propidium iodide, exposed to **C-8** at concentrations of 0.1–10 µg/mL, taken at t = 72 h.

Pictures of SNB-19 cell cultures stained with annexin and propidium iodide (control and exposed to **C-8** at concentrations of 0.1–10 µg/mL) were taken after 72 h of incubation (Figure 12). In the examined cell cultures subjected to 72 h of incubation with **C-8** at concentrations of 1–10 µg/mL, numerous red fluorescent cells (propidium iodide positive) were identified, indicating necrotic cell death in the culture. In the control culture, only single red fluorescent cells were identified.

The next step in assessing the anticancer activity of **C-8** involved determining the presence of proteins associated with cellular stress. Changes in the levels of selected cellular stress-related proteins were determined in SNB-19 cells exposed to **C-8** at a concentration of 10 μg/mL for 24 h. The assay was performed using the Proteome Profiler Array Human Cell Stress Array Kit ARY 018 (R&D Systems). The matrix enables simultaneous determination of 26 proteins (simultaneous determination of proteins: ADAMTS1, Bcl-2, Carbonic Anhydrase IX, Cited-2, COX-2, Cytochrome c, Dkk-4, FABP-1, HIF-1α, HIF-2α, Phospho-HSP27 (S78/S82), HSP60, HSP70, IDO, Phospho-JNK Pan (T183/Y185), NFκB1, p21/CIP1, p27, Phospho-p38α (T180/Y182), Phospho-p53 (S46), PON1, PON2, PON3, Thioredoxin-1, SIRT2, SOD2).

**Figure 13:**
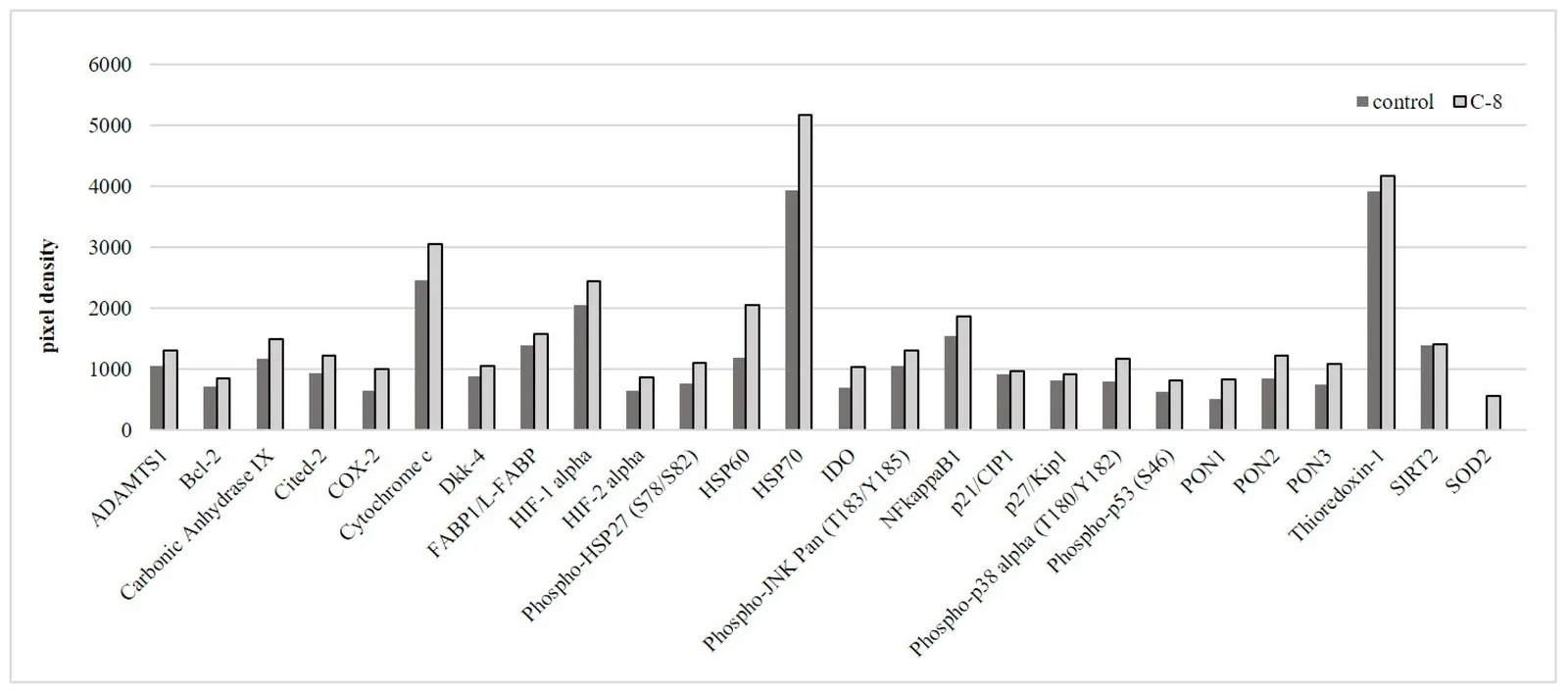
Changes in the expression of selected cellular stress-related proteins in SNB-19 cells treated with compound **C-8**.

Figure 13 shows changes in the levels of proteins associated with cellular stress in SNB-19 cells treated with the compound **C-8** at a concentration of 10 μg/mL. Cellular stress is a state in which a cell is exposed to factors that disrupt its normal functioning and homeostasis (internal balance). Cellular stress occurs when damage or stress exceeds the cell’s normal adaptive capacity. The cell then activates defense mechanisms, including increased production of chaperones; activation of antioxidant enzymes; repair of damaged proteins, DNA, and cell membranes; reduction in energy-consuming processes; and, in cases of severe damage, initiation of apoptosis. HSP60 and HSP70 are heat shock proteins belonging to the chaperone group. Their main function is to protect the cell from the effects of stress and maintain proper protein structure. HSP70 is one of the most important cellular chaperones. Under stressful conditions, HSP70 expression increases dramatically, which is why it is often used as a marker of cellular stress. HSP60, in turn, is found primarily in mitochondria, and its levels increase when the cell experiences stress leading to protein damage or misfolding, particularly in mitochondria. The presence of **C-8** in the culture medium of SNB-19 cells increases the levels of both heat shock proteins, HSP60 and HSP70. A family of enzymes called paraoxonases (PONs) also plays an important role in protecting cells from oxidative stress. This family includes three proteins: paraoxonase 1 (PON-1), paraoxonase 2 (PON-2), and paraoxonase 3 (PON-3). Their primary function is to protect cells from oxidative stress and limit lipid oxidation. In SNB-19 cells treated with **C-8**, a significant increase in PON-1 protein levels was observed, and, to a lesser extent, PON-2 and PON-3. A significant increase in the levels of HIF, which controls the cell’s response to hypoxia-related stress, was also observed in the cultured cells. At the same time, significantly elevated levels of cyclooxygenase 2 (COX-2) were observed. Its primary role in the cell is the production of prostaglandins (especially PGE2), which drive and maintain the body’s inflammatory response. COX-2 is induced primarily by NF-κB and MAPK (p38/JNK) during cellular stress, and the levels of these proteins are also elevated. Changes in selected pro- and antiapoptotic proteins were also observed. Tests revealed an increase in the amount of phosphorylated p53 protein, one of the most important regulators of the cell cycle. In response to cellular stress, p53 protein enables DNA repair or directs cells toward apoptosis. It also activates the transcription of proapoptotic genes, such as p21 and p27, although the test results showed only a slight increase in the levels of these two factors. By influencing the increase in transcription of pro-apoptotic genes, e.g., BAX, the p53 protein induces the mitochondrial apoptosis pathway, during which cytochrome c is released from mitochondria. The test showed an increase in the level of cytochrome c, but at the same time, a high level of the anti-apoptotic protein Bcl-2.

**Figure 14:**
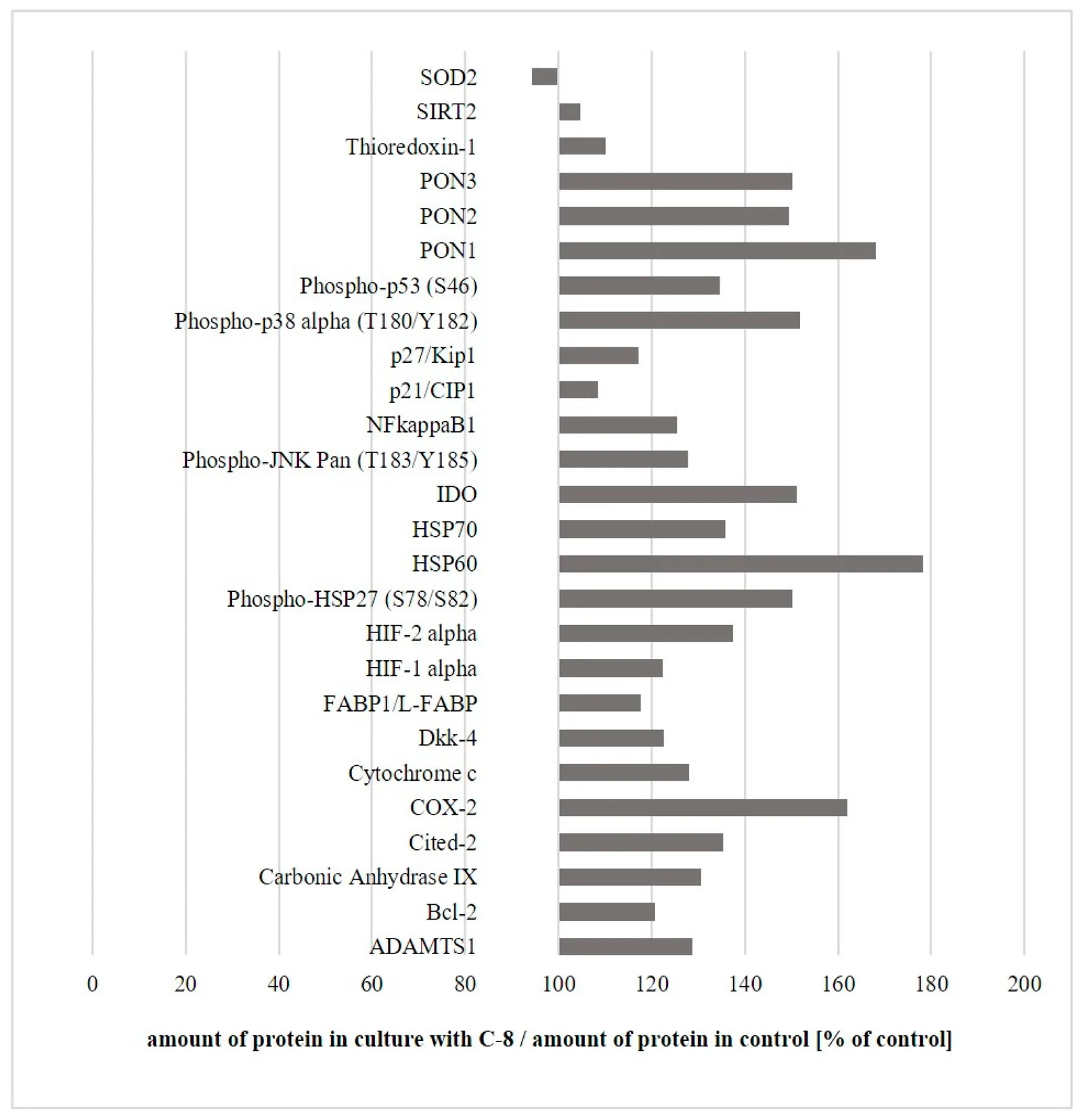
Expression profile of cellular stress proteins in SNB-19 culture exposed to **C-8** compared to individual proteins in the control culture—presented as % of control.

Figure 14 shows the changes in the described proteins in cultures exposed to **C-8** as % of the control (calculated on proteins in control cultures).

## 4. Conclusions

Eight derivatives of rhodanine-3-acetic acid (**C-1**–**C-8**) were obtained in yields ranging from 49% to 99%. The synthesized derivatives differed in the number and position of the methoxy groups in the phenyl (**C-1**–**C-6**) or naphthalene (**C-7**–**C-8**) substituents. In terms of thermal properties, the compounds under consideration were characterized by melting points ranging from 183 °C to 266 °C and thermal stability above 240 °C. An analysis of the **C-1** through **C-6** derivatives reveals that, in general, thermal stability (T_5_, T_10_, and T_max_) increases slightly as the number of methoxy groups in the substituent rises. Minor deviations are observed only for the **C-2** and **C-5** derivatives. A comparison of the **C-7** and **C-8** derivatives indicates that a methoxy group at the 6-position of the naphthalene ring has a much more favorable effect on thermal parameters than one at the 4-position. Furthermore, among all the compounds, **C-8** exhibited the best thermal properties. Compound **C-4** exhibited the largest hypsochromic shift in the absorption and emission spectra. This may be related to the negative inductive properties of –OCH_3_ in the meta position. Compounds with two methoxy groups exhibit a greater effect of substitution position on the location of the absorption and emission maximum than those with three. As expected, replacing the phenyl substituent with a naphthalene allowed for absorption spectra above 400 nm. Interestingly, however, the arrangement of the naphthalene substituent and the position of the attached-OCH_3_ group are crucial. Attaching the methoxy group at the 4-position of naphthalene (**C-7**) causes a distinct red shift relative to **C-8** (position 6). The emission spectra for the **C-7** and **C-8** derivatives were the most red-shifted.

When evaluating our findings, the integration of the 3T3 cytotoxicity profiles with the fixed-sample imaging results clearly points to compound **C-2** as the best candidate for robust, artifact-free cytoplasmic staining. At a concentration of 10 μg/mL, **C-2** achieves excellent contrast in the FITC/Alexa488 channel while maintaining 3T3 metabolic activity statistically unchanged (87.93% survival). Compared to **C-5**, which, despite offering an identically favorable high-signal and low-background imaging profile, triggers widespread cytotoxicity across the entire dosage spectrum. Furthermore, the localization of these stains to the cytoplasm, coupled with their verified compatibility with DAPI and phalloidin iFluor647, demonstrates that they can seamlessly integrate into multi-color multiplexing workflows without causing spectral cross-talk or disrupting the structural evaluation of the nucleus or F-actin filaments. The low live-imaging signal observed exclusively with compound **C-7** on the FITC/Alexa488 channel confirms its distinct capacity to cross intact cell membranes without requiring prior formaldehyde fixation. However, the practical utility of this live-stain behavior is heavily constrained by the compound’s high cytotoxicity at 10 μg/mL, which significantly reduces cell survival. Our results indicated that among the newly synthesized compounds, some showed antimicrobial effects, especially substance **C-1** towards all Gram-positive bacteria. Its activity was mild or moderate against these microorganisms. Bacteria belonging to E. faecalis, M. luteus and B. subtilis were most sensitive to this compound (MIC = 250 µg/mL). In the case of compound **C-6**, antibacterial activity was shown towards Enterococcus faecalis (MIC = 1000 µg/mL), Micrococcus luteus and both strains of Bacillus spp. (MIC = 500 µg/mL). Moreover, E. faecalis was sensitive to substances **C-3**, **C-4**, **C-7** and **C-8**.

Studies conducted on cancer cells indicate very limited cytotoxic activity of the tested compounds against tumors. No effect on the tested cells was observed for compounds **C-2**, **C-6**, and **C-7**. The remaining compounds exhibit selective activity against specific cell lines. Compounds **C-5** and **C-8** demonstrate the broadest spectrum of activity in this regard. Further studies on compound **C-8** and the SNB-19 cell line revealed that, despite the lack of dramatic effects following 72 h of exposure, the compound does induce changes in cell proliferation and migration (even at the lowest tested concentrations, i.e., 0.1 and 1 µg/mL). Changes leading to cell death (apoptosis and necrosis) also occur. Twenty-four hours after exposure to **C-8**, there is an increase in the levels of cellular stress proteins, such as the following: ADAMTS1, Bcl-2, carbonic anhydrase IX, Cited-2, COX-2, cytochrome c, Dkk-4, FABP-1, HIF-1α, HIF-2α, phospho-HSP27 (S78/S82), HSP60, HSP70, IDO, phospho-JNK Pan (T183/Y185), NFκB1, p21/CIP1, p27, phospho-p38α (T180/Y182), phospho-p53 (S46), PON1, PON2, PON3, thioredoxin-1, and SIRT2. In light of the results obtained, it therefore appears that the tested compounds (particularly **C-8**)—while not inducing significant cytotoxic effects in the short term—can, through changes occurring in the cells over a longer period, lead to the inhibition of tumor growth and contribute to its elimination.

## Figures and Tables

**Table 1:** Determined thermal parameters for **C-1**–**C-8** compounds.

Code	T_m_ ^a^ [^o^C]	T_5_ ^b^ [^o^C]	T_10_ ^b^ [^o^C]	T_max_ ^c^ [^o^C]
**C-1**	247	246	255	272
**C-2**	205	245	257	287
**C-3**	242	249	261	293
**C-4**	220	250	263	304
**C-5**	183	255	270	310
**C-6**	234	258	268	293
**C-7**	205	265	278	307
**C-8**	266	283	293	324

^a^ T_m_—temperature at which the sample melts (melting point); ^b^ T_5_ and T_10_—temperatures at which we observe 5% or 10% weight loss; ^c^ T_max_—temperature of maximum decomposition rate.

**Table 2:** Absorption and emission data for **C-1**–**C-8**.

Compound	Solvent	λ_abs_ [nm] (ε, 10^4^ M^−1^ cm^−1^)	λ_em_ [nm]	Stock Shift ^a^ [nm]	QY [%]	Eg (opt) [eV]
**C-1**	Toluene	392 (4.3)	441; 460	49	-	
	THF	389 (5.5)	450	61	-	
	CHCl_3_	394 (4.6)	460	66	-	
	MeCN	388 (4.9)	463	75	-	
	DMSO	393 (4.5)	459	66	0.02	2.82
	MeOH	387 (4.7)	452	65	-	
**C-2**	Toluene	407 (3.5)	440; 464	33; 57	-	
	THF	404 (4.3)	470	66	-	
	CHCl_3_	408 (4.4)	472	64	-	
	MeCN	402 (4.3)	480	78	-	
	DMSO	409 (3.4)	494	85	0.05	2.72
	MeOH	396 (3.4)	474	78	-	
**C-3**	Toluene	407 (3.9)	466	64	-	
	THF	408 (4.1)	470	62	-	
	CHCl_3_	416 (4.3)	479	63	-	
	MeCN	411 (4.2)	480	69	-	
	DMSO	418 (3.9)	487	69	0.03	2.64
	MeOH	408 (3.4)	476	68	-	
**C-4**	Toluene	366 (3.4); 380 (3.6)	435	55	-	
	THF	361 (3.7); 376 (4.0)	440	64	-	
	CHCl_3_	381 (3.7)	437	56	-	
	MeCN	373 (4.1)	-	-	-	
	DMSO	378 (3.9)	442	64	1	3.02
	MeOH	372 (3.9)	-	-		
**C-5**	Toluene	396 (3.3)	439; 461	43	-	
	THF	393 (4.9)	483	90	-	
	CHCl_3_	396 (4.5)	489	93	-	
	MeCN	389 (4.7)	507	118	-	
	DMSO	395 (3.2)	511	116	0.1	2.78
	MeOH	386 (3.6)	485	99	-	
**C-6**	Toluene	404 (2.8)	441 sh; 461	37; 57	-	
	THF	407 (4.1)	469	62	-	
	CHCl_3_	414 (4.9)	477	63	-	
	MeCN	412 (4.0)	476	64	-	
	DMSO	417 (2.8)	480	63	0.04	2.66
	MeOH	408 (2.6)	474	66	-	
**C-7**	Toluene	426 (2.8)	491	65	-	
	THF	423 (2.5)	491	68	-	
	CHCl_3_	432 (2.4)	501	69	-	
	MeCN	425 (2.3)	513	88	-	
	DMSO	430 (2.7)	515	85	0.001	2.50
	MeOH	419 (2.6)	497	78	-	
**C-8**	Toluene	411 (5.0)	471	60	-	
	THF	409 (4.2)	480	71	-	
	CHCl_3_	413 (4.3)	485	72	-	
	MeCN	408 (3.7)	502	94	-	
	DMSO	414 (4.7)	509	95	0.1	2.66
	MeOH	403 (5.3)	496	93	-	

^a^ λ_em_–λ_abs;_ The maximum absorption value was used for emission measurements.

**Table 3:** The activity data of studied compounds **C-1**–**C-8** expressed as MIC (MBC or MFC) [µg/mL] and {MBC/MIC or MFC/MIC ratio} against the reference strains of microorganisms. The standard antibiotics—ciprofloxacin (CIP) and vancomycin (VA *) for bacteria and nystatin (NY **) for fungi—were used as positive controls.

	Species	MIC (MBC) [µg/mL] and {MBC/MIC} or {MFC/MIC) Ratio of the Tested Compounds and Positive Controls								
		C-1	C-2	C-3	C-4	C-5	C-6	C-7	C-8	CIP/ VA */ NY **
Gram-positive bacteria	*Staphylococcus aureus* MSSA ATCC 29213	500 (>2000) {>4}	nd	2000 (>2000) {>1}	2000 (>2000) {>1}	nd	nd	2000 (>2000) {>1}	nd	0.48 (0.48) {1}
	*Staphylococcus* *aureus* MSSA ATCC 25923	500 (2000) {4}	nd	nd	nd	nd	nd	2000 (>2000) {>1}	nd	0.48 (0.48) {1}
	*Staphylococcus aureus* MRSA ATCC 43300	1000 (>2000) {>2}	nd	2000 (>2000) {>1}	2000 (>2000) {>1}	nd	nd	2000 (>2000) {>1}	nd	0.24 (0.24) {1}
	*Staphylococcus* *epidermidis* ATCC 12228	1000 (>2000) {>2}	nd	2000 (>2000) {>1}	nd	nd	nd	2000 (>2000) {>1}	nd	0.12 (0.12) {1}
	*Enterococcus* *faecalis* ATCC 29212	250 (>2000) {>8}	nd	1000 (>2000) {>2}	1000 (>2000) {>2}	nd	1000 (>2000) {>2}	1000 (>2000) {>2}	1000 (>2000) {>2}	0.98 * (1.95) {2}
	*Micrococcus luteus* ATCC 10240	250 (>2000) {>8}	nd	2000 (>2000) {>1}	1000 (>2000) {>2}	nd	500 (1000) {2}	500 (1000) {2}	2000 (>2000) {>1}	0.98 (1.98) {2}
	*Bacillus* *subtilis* ATCC 6633	250 (2000) {8}	nd	2000 (>2000) {>1}	2000 (>2000) {>1}	nd	500 (2000) {4}	2000 (>2000) {>1}	1000 (>2000) {>2}	0.03 (0.03) {1}
	*Bacillus* *cereus* ATCC 10876	500 (2000) {4}	nd	1000 (>2000) {>2}	2000 (>2000) {>1}	nd	500 (1000) {2}	2000 (>2000) {>1}	500 (>2000) {>4}	0.06 (0.12) {2}
Gram-negative bacteria	*Klebsiella* *pneumoniae* ATCC 13883	2000 (>2000) {>1}	nd	2000 (>2000) {>1}	nd	nd	2000 (>2000) {>1}	2000 (>2000) {>1}	2000 (>2000) {>1}	0.12 (0.12) {1}
	*Proteus mirabilis* ATCC 12453	2000 (2000) {1}	nd	2000 (>2000) {>1}	nd	nd	nd	2000 (>2000) {>1}	nd	0.03 (0.03) {1}
	*Salmonella Typhimurium* ATCC 14028	2000 (>2000) {>1}	nd	2000 (>2000) {>1}	nd	nd	nd	nd	2000 (>2000) {>1}	0.06 (0.06) {1}
	*Escherichia* *coli* ATCC 25922	2000 (>2000) {>1}	nd	2000 (>2000) {>1}	nd	nd	nd	nd	nd	0.004 (0.004) {1}
	*Pseudomonas aeruginosa* ATCC 27853	2000 (>2000) {>1}	nd	2000 (>2000) {>1}	nd	nd	2000 (>2000) {>1}	2000 (>2000) {>1}	2000 (>2000) {>1}	0.48 (0.98) {2}
Fungi	*Candida* *albicans* ATCC 10231	2000 (2000) {1}	2000 (>2000) {>1}	nd	2000 (>2000) {>1}	2000 (>2000) {>1}	2000 (>2000) {>1}	nd	2000 (>2000) {>1}	0.48 ** (0.48) {1}
	*Candida* *parapsilosis* ATCC 2201	1000 (2000) {2}	2000 (>2000) {>1}	2000 (>2000) {>1}	2000 (2000) {1}	2000 (>2000) {>1}	2000 (2000) {1}	nd	2000 (>2000) {>1}	0.24 ** (0.48) {2}
	*Candida* *glabrata* ATCC 90030	2000 (2000) {1}	2000 (>2000) {>1}	2000 (>2000) {>1}	2000 (>2000) {>1}	2000 (>2000) {>1}	2000 (>2000) {>1}	nd	nd	0.24 ** (0.48) {2}
	*Candida* *krusei* ATCC 14243	2000 (2000) {1}	2000 (>2000) {>1}	2000 (>2000) {>1}	2000 (>2000) {>1}	2000 (>2000) {>1}	2000 (>2000) {>1}	nd	nd	0.24 ** (0.24) {1}
	*Candida tropicalis* ATCC 1369	2000 (2000) {1}	2000 (>2000) {>1}	nd	nd	2000 (>2000) {>1}	2000 (>2000) {>1}	nd	nd	0.24 ** (0.48) {2}
	*Candida lusitaniae* ATCC 3449	1000 (2000) {2}	2000 (>2000) {>1}	2000 (>2000) {>1}	2000 (>2000) {>1}	2000 (>2000) {>1}	2000 (>2000) {>1}	nd	2000 (>2000) {>1}	0.48 ** (0.98) {2}
	*Candida* *auris* CDC B11903	2000 (2000) {1}	2000 (>2000) {>1}	nd	nd	2000 (>2000) {>1}	2000 (>2000) {>1}	nd	2000 (>2000) {>1}	0.98 ** (0.98) {1}

The ranges of activity: nd—not determined (MIC > 2000 µg/mL); no bioactivity—MIC > 1000 µg/mL; mild bioactivity—MIC = 501–1000 µg/mL; moderate bioactivity—MIC = 126–500 µg/mL: good bioactivity—MIC = 26–125 µg/mL; strong bioactivity—MIC = 10–25 µg/mL; very strong bioactivity—MIC < 10 µg/mL.

**Table 4:** LC50 values for **C-1**, **C-2**, **C-3**, **C-4**, **C-5**, **C-6**, **C-7** and **C-8** were determined for SNB-19, C32, Caco-2, MDA-MB-231, A2058, Colo-829, LoVo/DX, Panc-1, Skov3, HT29, A549, SH-4 and HFF-1 cell lines exposed to the test compounds for 72 h; nd—could not be determined.

LC50 [µg/mL]								
	C-1	C-2	C-3	C-4	C-5	C-6	C-7	C-8
SNB-19	>100	>100	nd	>100	>100	>100	>100	64.92
C32	76.56	>100	nd	14.54	43.38	nd	nd	nd
Caco-2	nd	nd	nd	nd	>100	nd	>100	60.94
MDA-MB-231	>100	nd	nd	nd	82.41	nd	nd	nd
A2058	36.38	nd	13.10	nd	80.58	>100	nd	15.21
Colo-829	>100	>100	>100	nd	>100	>100	nd	nd
LoVo/DX	nd	nd	nd	nd	nd	nd	nd	nd
Panc-1	>100	nd	nd	nd	>100	nd	nd	>100
Skov3	>100	>100	>100	>100	>100	nd	nd	nd
HT29	>100	nd	nd	>100	>100	nd	nd	nd
A549	>100	nd	nd	44.04	>100	nd	nd	nd
SH-4	nd	nd	nd	nd	nd	nd	nd	nd
HFF-1	>100	>100	>100	nd	nd	nd	>100	>100

## Data Availability

The original contributions presented in this study are included in the article/Supplementary Material. Further inquiries can be directed to the corresponding author.

## Supplementary Materials

The following supporting information can be downloaded at https://www.mdpi.com/article/10.3390/ma19204287/s1, Figure S1: DSC thermograms of **C-1**; Figure S2: DSC thermograms of **C-2**; Figure S3: DSC thermograms of **C-3**; Figure S4: DSC thermograms of **C-4**; Figure S5: DSC thermograms of **C-5**; Figure S6: DSC thermograms of **C-6**; Figure S7: DSC thermograms of **C-7**; Figure S8: DSC thermograms of **C-8**; Figure S9: TGA thermogram for **C-1**–**C-8;** Figure S10. HOMO/LUMO plots; Figure S11. Density difference plots; Figure S12. The MEP surfaces; Figure S13. Predicted binding conformations of compounds **C1**–**C8** in Concanavalin A (ConA) from AutoDock simulations (AutoDock 4.2); Figure S14. Predicted binding conformations of compounds **C1**–**C8** in Human Serum Albumin (HSA) from AutoDock simulations; Table S1. The frontier orbital energies in tested solvents for **C-1**–**C-8** compounds. Values are given in [eV]; Table S2. Occupancy (e), energy (a.u.) and polarity (%) of natural bond orbitals (NBOs) and hybrids calculated for investigated complexes; Table S3. CT parameters for the bright low-lying excited state. Values of q_CT_ are given in [e] and DCT in [Å]; Table S4. Free energies of solvation (kcal/mol); Table S5. Linear optical properties (λ_abs_, nm); Table S6. Calculated values of dipole moments (in D) for the ground and CT excited states; Table S7. TD-DFT fluorescence maxima (λ_em_) of compounds **C1**–**C8** in different solvents; Table S8. Nonlinear properties. Values are given in a.u.; Table S9. AutoDock binding free energies (Δ*G*b) for compounds **C1**–**C8** docked to Concanavalin A (ConA); Table S10. AutoDock binding free energies (Δ*G*b) for compounds **C1**–**C8** docked to Human Serum Albumin (HSA); Table S11. Predicted LogP and bioaccumulation factor (Log_BCF_) for compounds **C1**–**C8**; Table S12. Predicted acute toxicity for rats (LD_50_). Values given in mg/kg body weight; Table S13. Predicting the toxicity of tested compounds. PR—prediction (In—inactive; A—active); P%—probability expressed as a percentage; Table S14. Absorption and emission spectra of C-series in various solvents at room temperature; Table S15. Comparison of normalized absorption spectra of compounds **C-1**–**C-8**; Table S16. UV-Vis spectra in DMSO recorded at time intervals at room temperature; Table S17. Comparison of normalized emission spectra of compounds C-series; Table S18. The Lippert–Mataga plots.
