# Supplementary material for: The Influence of the Number and Position of the Methoxy Group on the Physicochemical Properties and Possible Applications of Rhodanine-3-Acetic Acid Derivatives
Source: Materials (Basel). 2026 Oct 11;19(20):4287. doi: 10.3390/ma19204287 (PMC13706306; doi:10.3390/ma19204287)
Supplement: Supplementary file 1 [file materials-19-04287-s001.zip › materials-4523063-supplementary.pdf]

Supporting Information

The influence of the methoxy group on the physicochemical properties and possible applications of rhodanine-3-acetic acid derivatives

Agata Szłapa-Kula [a]*, Anna Biernasiuk [b], Łukasz Kaźmierski [c], Dariusz Kuśmierz [d], Justyna Płonka-Czerw [d], Małgorzata Latocha [d], Martyna Zegarska [e], Slawomir Kula [a] and Przemysław Krawczyk [f]

[a] *University of Silesia in Katowice, Faculty of Science and Technology, Institute of Chemistry, Szkolna 9 St., 40-007 Katowice, Poland*

[b] *Department of Pharmaceutical Microbiology, Faculty of Pharmacy, Medical University of Lublin, Chodźki 1, 20-093 Lublin, Poland*

[c] *Nicolaus Copernicus University, Collegium Medicum, Faculty of Medicine, Department of Urology and Andrology, Karłowicza 24, 85-092 Bydgoszcz, Poland*

[d] *Department of Cell Biology, Faculty of Pharmaceutical Sciences in Sosnowiec, Medical University of Silesia, Jednosci 9, 41-200 Sosnowiec, Poland*

[e] *Antoni Jurasz Univeristy Hospital No. 1 in Bydgoszcz, Marii Skłodowskiej-Curie 9, 85-094 Bydgoszcz, Poland*

[f] *Nicolaus Copernicus University, Collegium Medicum, Faculty of Pharmacy, Department of Physical Chemistry, Kurpińskiego 5, 85-950 Bydgoszcz, Poland*

*Corresponding author at: Institute of Chemistry, Faculty of Science and Technology, University of Silesia, 9 Szkolna Str., 40-006 Katowice, Poland.
E-mail address: agata.szlapa-kula@ud.edu.pl

Table of Contents

1. **Materials** 2
2. **Measurements and general methods** 2
3. **Synthesis and NMR spectra** 4
4. **Thermal properties** 27
5. **DFT calculations** 30
6. **Optical properties** 55
7. **Literature** 62

1. Materials

4-methoxybenzaldehyde (98%, Merck (Sigma-Aldrich)), 3,4-Dimethoxybenzaldehyde (>98%, TCI), 2,4-dimethoxybenzaldehyde (>98%, TCI), 3,5-dimethoxybenzaldehyde (>98%, TCI), 3,4,5-trimethoxybenzaldehyde (>98%, TCI), 2,4,6-trimethoxybenzaldehyde (>98%, TCI), 4-methoxy-1-naphthaldehyde (>97%, TCI), rhodanine-3-acetic acid (>98.0%, TCI), 6-methoxy-2-naphthaldehyde (98%, Merck (Sigma-Aldrich)), ammonium acetate (puriss. p.a., ACS reagent, reag. Ph. Eur., ≥98%, Merck-Sigma-Aldrich), glacial acetic acid (99,5% pure p. a, Chempur), DMSO-d_6 (99.9 atom % D, Merck-Sigma-Aldrich), ethanol (96% pure p. a, Stanlab), acetonitrile (for HPLC-GC, ≥99.8% (GC), Merck-Sigma-Aldrich), chloroform (for HPLC, ≥99.8%, amylene stabilized, Merck-Sigma-Aldrich), methanol (for HPLC, ≥99.9%, Merck-Sigma-Aldrich), DMSO (pure for analysis, Chempur), THF (pure for analysis, Chempur), Toluene (pure for analysis, Chempur).

2. Measurements and general methods

General apparatus

Bruker Avance 500 MHz instrument used to record the NMR spectra in DMSO-d6 (as solvent). Differential scanning calorimetry (DSC) and thermogravimetric analysis (TGA) were performed on the following devices: TA – DSC 25 and TA – TGA 55. UV/Vis spectra were recorded with a Biosens model UV 5600 UV/Vis spectrophotometer. Photoluminescence emission spectra were acquired using Hitachi Fluorescence Spectrophotometer F-7100. The photoluminescence quantum yield of all rhodanine-3-acetic acid derivatives (C1 – C8) was measured using an integrating sphere at room temperature and using the solvent (DMSO) as a blank.

In vitro antimicrobial assay

The examined compounds **C-1 –C-8** were screened *in vitro* for antibacterial and antifungal activities using the broth microdilution method according to European Committee on Antimicrobial Susceptibility Testing (EUCAST) [1] and Clinical and Laboratory Standards Institute guidelines [2] against a panel of 20 reference strains of microorganisms, including Gram-positive bacteria (*Staphylococcus aureus* ATCC 43300 (Methicillin Resistant *S. aureus* – MRSA), *Staphylococcus aureus* ATCC 29213 (Methicillin Susceptible *S. aureus* – MSSA), *Staphylococcus aureus* ATCC 25923 (Methicillin Susceptible *S. aureus* – MSSA), *Staphylococcus epidermidis* ATCC 12228, *Enterococcus faecalis* ATCC 29212, *Micrococcus*

luteus ATCC 10240, *Bacillus subtilis* ATCC 6633 and *Bacillus cereus* ATCC 10876), Gram-negative bacteria (*Escherichia coli* ATCC 25922, *Klebsiella pneumoniae* ATCC 13883, *Proteus mirabilis* ATCC 12453, *Salmonella* Typhimurium ATCC 14028 and *Pseudomonas aeruginosa* ATCC 9027) and fungi belonging to yeasts (*Candida albicans* ATCC 10231, *Candida parapsilosis* ATCC 22019, *Candida glabrata* ATCC 90030, *Candida krusei* ATCC 14243, *Candida tropicalis* ATCC 1369, *Candida lusitaniae* ATCC 3449, and *Candida auris* CDC B11903). The microorganisms came from American Type Culture Collection (ATCC), and CDC (Centers for Disease Control and Prevention) routinely used for the evaluation of antimicrobials. All the used microbial cultures were first subcultured on nutrient agar or Sabouraud agar at 35°C for 18-24 h or 30°C for 24-48 h for bacteria and fungi, respectively.

Samples containing examined compounds **C-1 – C-8** were first dissolved in dimethyl sulfoxide – DMSO (20 mg/mL). Subsequently MIC (Minimal Inhibitory Concentration) of these substances was examined by the microdilution broth method, using their two-fold dilutions in Mueller-Hinton broth (for bacteria) and RPMI 1640 broth with MOPS (for fungi) prepared in 96-well polystyrene plates. Final concentrations of the compounds ranged from 2000 to 15.62 µg/mL. Microbial suspensions were prepared in 0.85% NaCl with an optical density of 0.5 McFarland standard. Next bacterial or fungal suspensions were added per each well containing broth and various concentrations of the examined compounds. After incubation (under the same conditions as before), the MIC was assessed spectrophotometric as the lowest concentration of the samples showing complete bacterial or fungal growth inhibition. Appropriate, growth and sterile controls were carried out. The medium with no tested substances was used also as control. The inhibition of microbial growth was judged by comparison with a control culture prepared without any sample tested. Ciprofloxacin, vancomycin or nystatin (Sigma-Aldrich Chemicals, St. Louis, MO, USA) were used as a reference antibacterial or antifungal compounds, respectively.

The MBC (Minimal Bactericidal Concentration) or MFC (Minimal Fungicidal Concentration) are defined as the lowest concentration of the compounds that is required to kill a particular bacterial or fungal species. MBC or MFC was determined by removing the culture using for MIC determinations from each well and spotting onto appropriate agar medium. Next, the plates were incubated. The lowest compounds concentrations with no visible growth observed were assessed as a bactericidal or fungicidal concentrations. All the experiments were repeated three times and representative data (as mode) are presented [3, 4].

In antimicrobial activity studies, no bioactivity was defined as a MIC > 1000 µg/mL, mild bioactivity as a MIC in the range 501 – 1000 µg/mL, moderate bioactivity with MIC from 126 to 500 µg/mL, good bioactivity as a MIC in the range 26 – 125 µg/mL, strong bioactivity with MIC between 10 and 25 µg/mL and very strong bioactivity as a MIC < 10 µg/mL [5]. The MBC/MIC or MFC/MIC ratios were calculated in order to determine bactericidal/fungicidal (MBC/MIC ≤ 4, MFC/MIC ≤ 4) or bacteriostatic/fungistatic (MBC/MIC > 4, MFC/MIC > 4) effect of the tested compounds.

3. Synthesis and NMR spectra

Preparation of 2-(5-benzylidene-4-oxo-2-thioxothiazolidin-3-yl)acetic acid derivatives

The aldehyde (2.20 mmol), rhodanine-3-acetic acid (0,45 g, 2.30 mmol), ammonium acetate (0,40 g, 5.20 mmol) and acetic acid (30 mL) were introduced into a 100 mL round-bottom flask. The whole was heated at reflux for 24 hours. The resulting precipitate was filtered off and washed thoroughly with water. For purification, it was crystallized in ethanol.

2-[5-(4-methoxybenzylidene)-4-oxo-2-thioxo-thiazolidin-3-yl]-acetic acid (C-1)

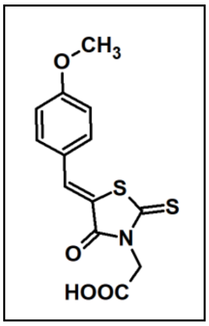

C-1 was obtained as red crystals with **99%** yield. **^{1}H NMR (500 MHz, DMSO) δ** 13.44 (s, 1H, **H**^{A1}), 7.87 (s, 1H, **H**^{A8}), 7.66 (d, *J* = 9.0 Hz, 2H, **H**^{B2}), 7.14 (d, *J* = 8.9 Hz, 2H, **H**^{B3}), 4.74 (s, 2H, **H**^{A2}), 3.85 (s, 3H, **H**^{B7}). **^{13}C NMR (125 MHz, DMSO) δ** 193.12 (**C**^{A7}), 167.34 (**C**^{A1}), 166.44 (**C**^{A4}), 161.78 (**C**^{B4}), 134.19 (**C**^{A8}), 133.14 (**C**^{B2}), 125.39 (**C**^{B1}), 118.47 (**C**^{A5}), 115.25 (**C**^{B3}), 55.64 (**C**^{B7}), 44.99 (**C**^{A2}).

2-[5-(3,4-dimethoxybenzylidene)-4-oxo-2-thioxo-thiazolidin-3-yl]-acetic acid (C-2)

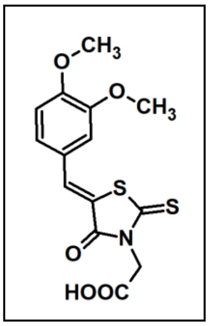

C-2 was obtained as red solid with **49%** yield. **^{1}H NMR (500 MHz, DMSO) δ** 13.43 (s, 1H, **H**^{A1}), 7.85 (s, 1H, **H**^{A8}), 7.28 (dd, *J* = 8.7, 2.1 Hz, 1H, **H**^{B2}), 7.25 (s, 1H, **H**^{B6}), 7.17 (d, *J* = 8.5 Hz, 1H, **H**^{B3}), 4.74 (s, 2H, **H**^{A2}), 3.85 (s, 3H, **H**^{B8}), 3.84 (s, 3H, **H**^{B7}). **^{13}C NMR (125 MHz, DMSO) δ** 193.08 (**C**^{A7}), 167.34 (**C**^{A1}), 166.39 (**C**^{A4}), 151.67 (**C**^{B5}), 149.11 (**C**^{B4}), 134.57 (**C**^{A8}), 125.55 (**C**^{B1}), 125.07 (**C**^{B2}), 118.59 (**C**^{A5}), 113.75 (**C**^{B6}), 112.31 (**C**^{B3}), 55.81 (**C**^{B8}), 55.62 (**C**^{B7}), 44.99 (**C**^{A2}).

2-[5-(2,4-dimethoxybenzylidene)-4-oxo-2-thioxo-thiazolidin-3-yl]-acetic acid (C-3)

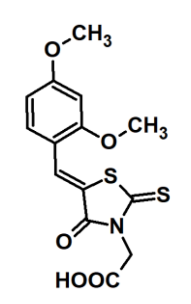

C-3 was obtained as red solid with **88%** yield. **^{1}H NMR (500 MHz, DMSO) δ** 13.41 (s, 1H, **H^{A1}**), 7.93 (s, 1H, **H^{A8}**), 7.43 (d, *J* = 8.7 Hz, 1H, **H^{B2}**), 6.73 (dd, *J* = 8.5, 2.4 Hz, 1H, **H^{B3}**), 6.71 (s, 1H, **H^{B5}**), 4.72 (s, 2H, **H^{A2}**), 3.93 (s, 3H, **H^{B7}**), 3.87 (s, 3H, **H^{B8}**). **^{13}C NMR (125 MHz, DMSO) δ** 193.61 **(C^{A7})**, 167.39 **(C^{A1})**, 166.62 **(C^{A4})**, 164.16 **(C^{B4})**, 160.19 **(C^{B6})**, 132.33 **(C^{B2})**, 129.56 **(C^{A8})**, 118.02 **(C^{A5})**, 114.21 **(C^{B1})**, 107.18 **(C^{B3})**, 98.67 **(C^{B5})**, 55.97 **(C^{B7})**, 55.83 **(C^{B8})**, 44.95 **(C^{A2})**.

2-[5-(3,5-dimethoxybenzylidene)-4-oxo-2-thioxo-thiazolidin-3-yl]-acetic acid (C-4)

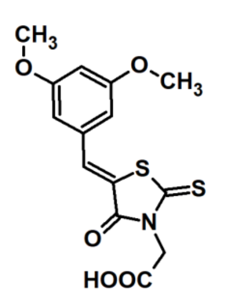

C-4 was obtained as red solid with **80%** yield. **^{1}H NMR (500 MHz, DMSO) δ** 13.47 (s, 1H, **H^{A1}**), 7.83 (s, 1H, **H^{A8}**), 6.80 (s, 2H, **H^{B2,B6}**), 6.69 (s, 1H, **H^{B4}**), 4.74 (s, 2H, **H^{A2}**), 3.81 (s, 6H, **H^{B7,B8}**). **^{13}C NMR (125 MHz, DMSO) δ** 193.16 **(C^{A7})**, 167.27 **(C^{A1})**, 166.25 **(C^{A4})**, 160.98 **(C^{B5,B3})**, 134.66 **(C^{B1})**, 134.00 **(C^{A8})**, 122.52 **(C^{A5})**, 108.33 **(C^{B2,B6})**, 103.34 **(C^{B4})**, 55.55 **(C^{B7,B8})**, 45.03 **(C^{A2})**.

2-[5-(3,4,5-trimethoxybenzylidene)-4-oxo-2-thioxo-thiazolidin-3-yl]-acetic acid (C-5)

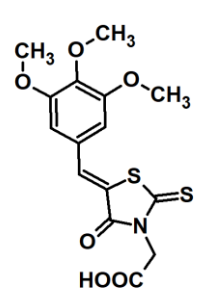

C-5 was obtained as red solid with **65%** yield. **^{1}H NMR (500 MHz, DMSO) δ** 7.85 (s, 1H, **H^{A8}**), 6.98 (s, 2H, **H^{B2,B6}**), 4.75 (s, 2H, **H^{A2}**), 3.86 (s, 6H, **H^{B7,B9}**), 3.75 (s, 3H, **H^{B8}**). **^{13}C NMR (125 MHz, DMSO) δ** 193.11 **(C^{A7})**, 167.30 **(C^{A1})**, 166.31 **(C^{A4})**, 153.35 **(C^{B3,B5})**, 140.20 **(C^{B4})**, 134.34 **(C^{A8})**, 128.30 **(C^{B1})**, 120.69 **(C^{A5})**, 108.29 **(C^{B2,B6})**, 60.29 **(C^{B8})**, 56.11 **(C^{B7,B9})**, 45.04 **(C^{A2}).**

2-[5-(2,4,6-trimethoxybenzylidene)-4-oxo-2-thioxo-thiazolidin-3-yl]-acetic acid (C-6)

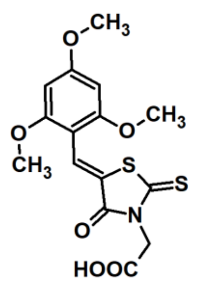

C-6 was obtained as orange solid with **75%** yield. **^{1}H NMR (500 MHz, DMSO) δ** 13.34 (s, 1H, **H^{A1}**), 8.02 (s, 1H, **H^{A8}**), 6.34 (s, 2H, **$H^{B3,B5}$**), 4.69 (s, 2H, **H^{A2}**), 3.91 (s, 6H, **$H^{B7,B9}$**), 3.88 (s, 3H, **H^{B8}**). **^{13}C NMR (125 MHz, DMSO) δ** 194.96 (**C^{A7}**), 167.55 (**C^{A1}**), 167.11 (**C^{A4}**), 165.50 (**C^{B4}**), 160.21 (**$C^{B2,B6}$**), 127.74 (**C^{A8}**), 118.66 (**C^{A5}**), 103.47 (**C^{B1}**), 91.21 (**$C^{B3,B5}$**), 55.97 (**$C^{B7,B9}$**), 55.92 (**C^{B8}**), 44.89 (**C^{A2}**).

2-[5-((4-methoxynaphthalen-1-yl)-methylene)-4-oxo-2-thioxo-thiazolidin-3-yl]-acetic acid (C-7)

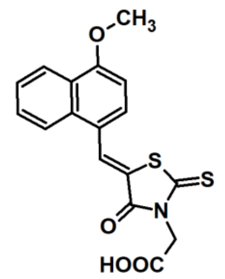

C-7 was obtained as orange solid with **91%** yield. **^{1}H NMR (500 MHz, DMSO) δ** 13.47 (s, 1H, **H^{A1}**), 8.47 (s, 1H, **H^{A8}**), 8.27 (d, J = 8.7 Hz, 1H, **H^{B10}**), 8.21 (d, J = 8.7 Hz, 1H, **H^{B6}**), 7.72 (dd, J = 8.5, 5.6 Hz, 2H, **$H^{B4,B5}$**), 7.66 – 7.62 (m, 1H, **H^{B9}**), 7.19 (d, J = 8.2 Hz, 1H, **H^{B3}**), 4.77 (s, 2H, **H^{A2}**), 4.07 (s, 3H, **H^{B11}**). **^{13}C NMR (125 MHz, DMSO) δ** 193.64 (**C^{A7}**), 167.38 (**C^{A1}**), 165.99 (**C^{A4}**), 157.83 (**C^{B8}**), 132.38 (**C^{B1}**), 130.45 (**C^{A8}**), 129.46 (**C^{B5}**), 128.42 (**C^{B4}**), 126.49 (**C^{B9}**), 124.94 (**C^{B2}**), 123.22 (**C^{B6}**), 122.38 (**C^{B10}**), 121.96 (**C^{A5}**), 121.68 (**C^{B7}**), 104.92 (**C^{B3}**), 56.22 (**C^{B11}**), 45.00 (**C^{A2}**).

2-[5-((6-methoxynaphthalen-2-yl)-methylene)-4-oxo-2-thioxo-thiazolidin-3-yl]-acetic acid (C-8)

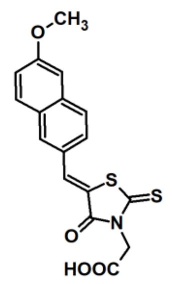

C-8 was obtained as orange solid with **99%** yield. **^{1}H NMR (500 MHz, DMSO) δ** 8.20 (s, 1H, **H^{A8}**), 8.01 (d, J = 9.0 Hz, 1H, **H^{B9}**), 7.99 (s, 1H, **H^{B2}**), 7.96 (d, J = 8.9 Hz, 1H, **H^{B4}**), 7.69 (dd, J = 8.6, 1.9 Hz, 1H, **H^{B5}**), 7.43 – 7.40 (m, 1H, **H^{B7}**), 7.27 (dd, J = 8.9, 2.5 Hz, 1H, **H^{B10}**), 4.76 (s, 2H, **H^{A2}**), 3.92 (s, 3H, **H^{B11}**). **^{13}C NMR (125 MHz, DMSO) δ** 193.26 (**C^{A7}**), 167.34 (**C^{A1}**), 166.45 (**C^{A4}**), 159.42 (**C^{B6}**), 135.51 (**$C^{B8,B3}$**), 134.44 (**C^{B2}**), 132.24 (**C^{A8}**), 130.80 (**C^{B9}**), 128.21 (**C^{B1}**), 128.07 (**C^{B4}**), 127.04 (**C^{B5}**), 120.44 (**C^{A5}**), 119.93 (**C^{B10}**), 106.23 (**C^{B7}**), 55.50 (**C^{B11}**), 45.04 (**C^{A2}**).

NMR Spectra

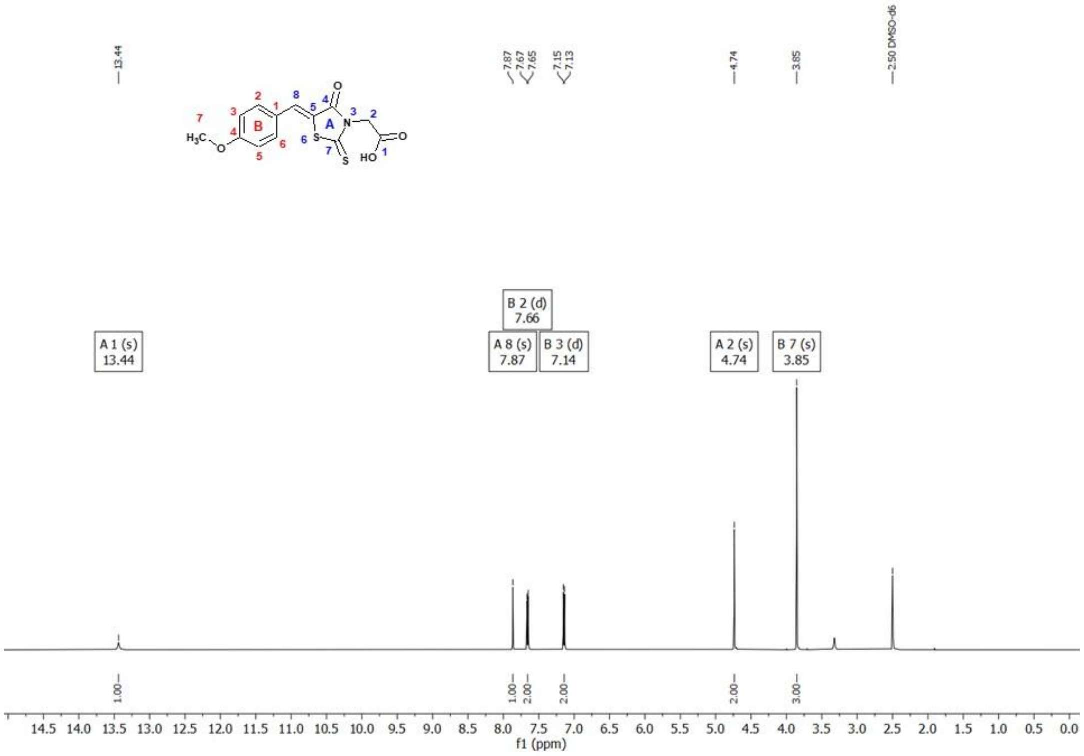

^{1}H NMR of C-1

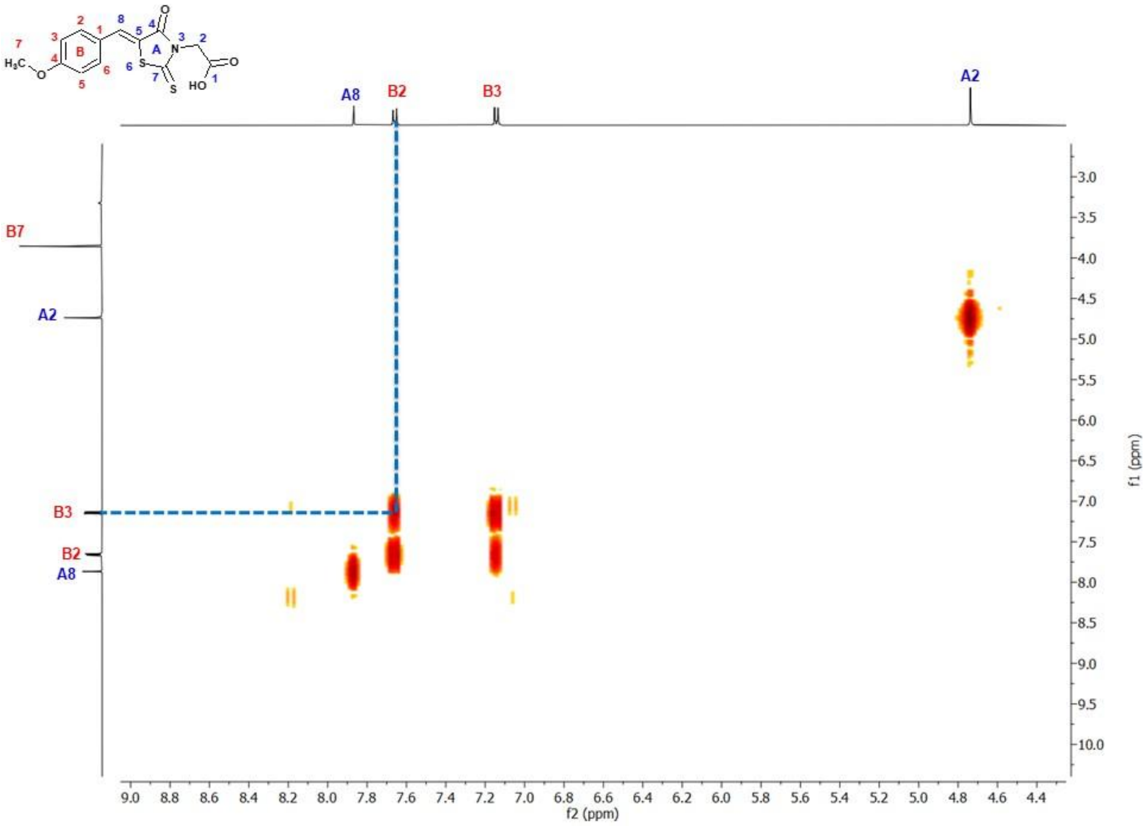

H-H COSY of C-1

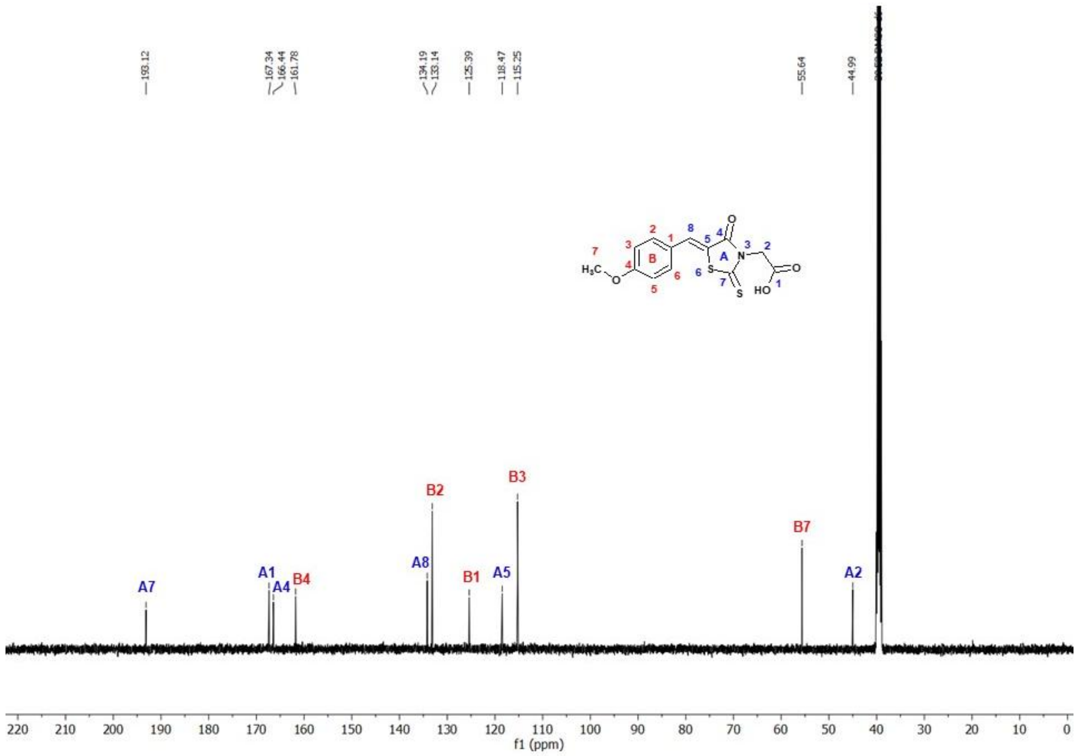

^{13}C NMR of C-1

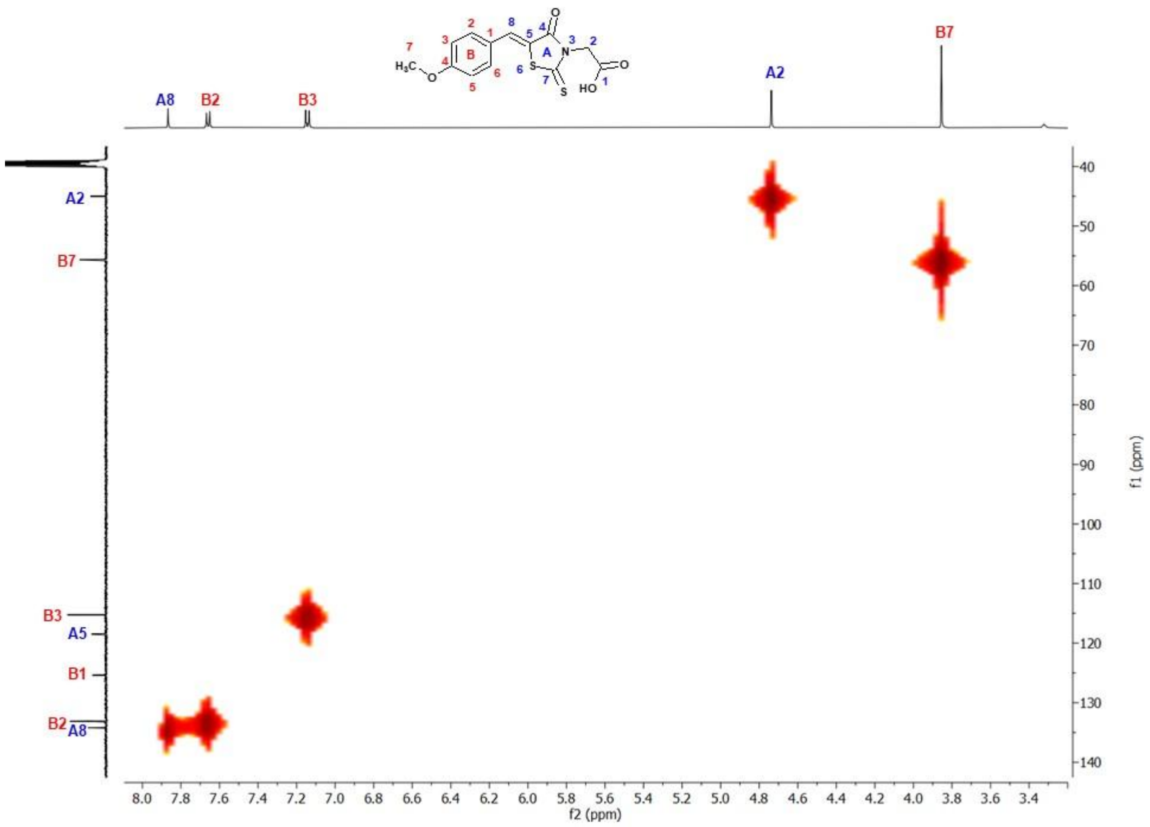

H-C HMQC of C-1

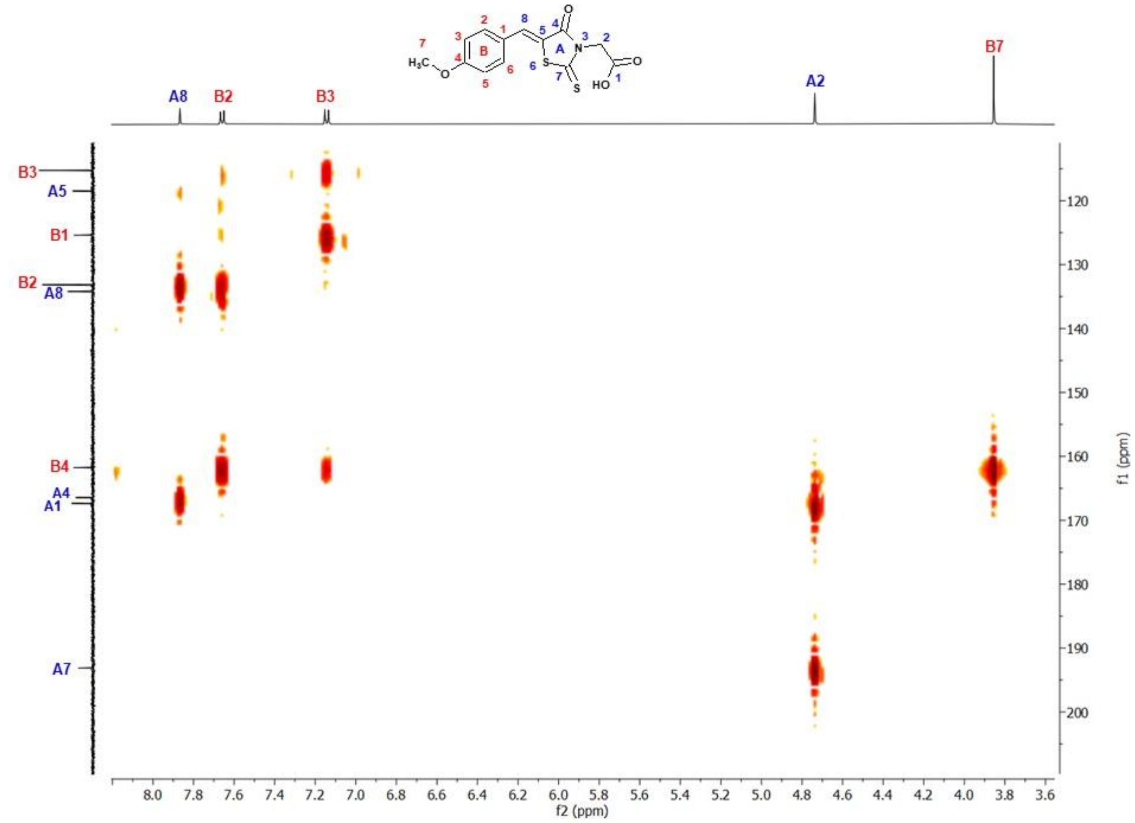

H-C HMBC of C-1

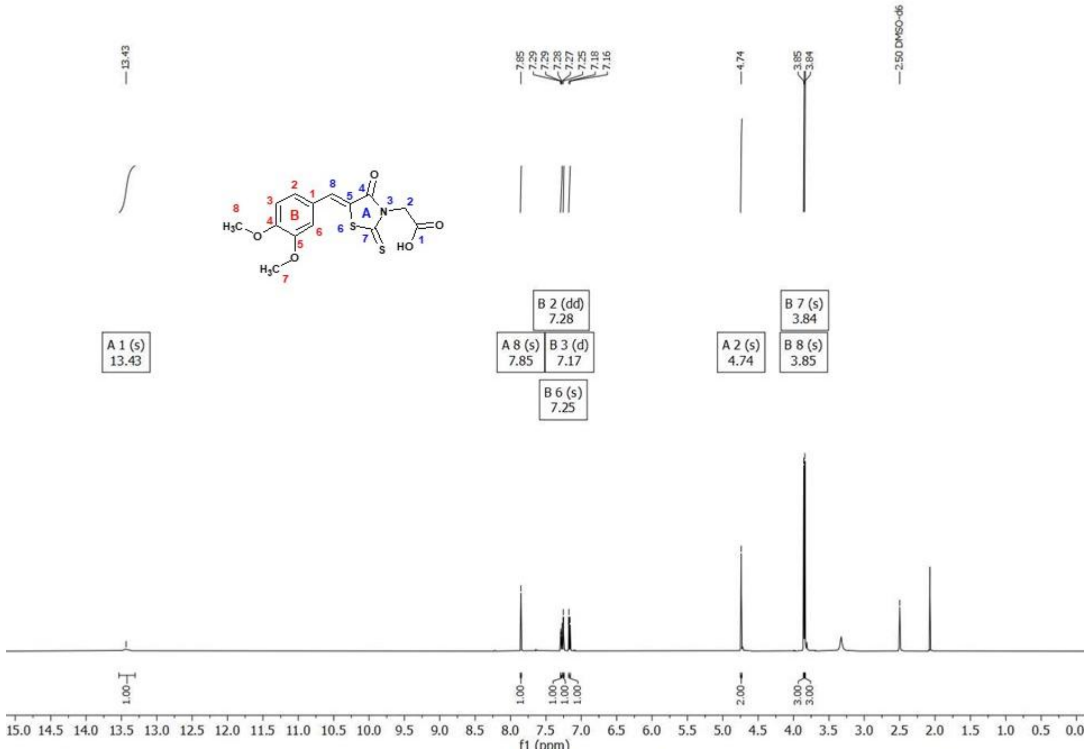

^{1}H NMR of C-2

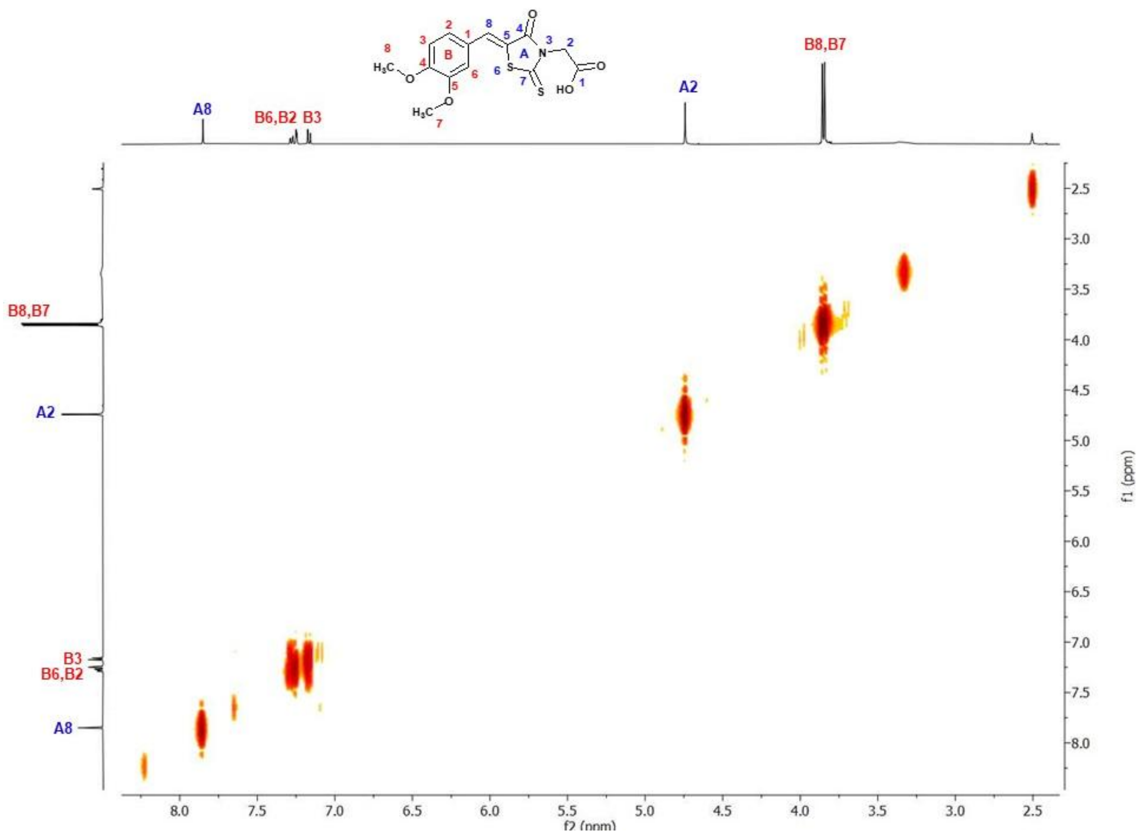

H-H COSY of C-2

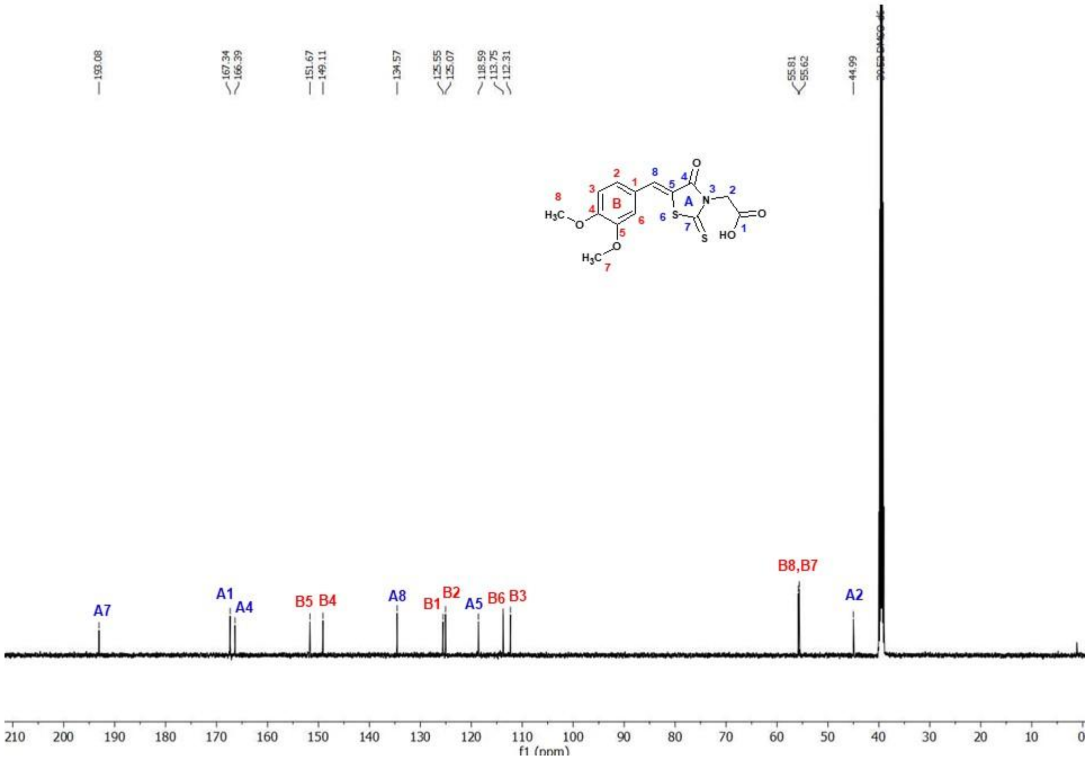

^{13}C NMR of C-2

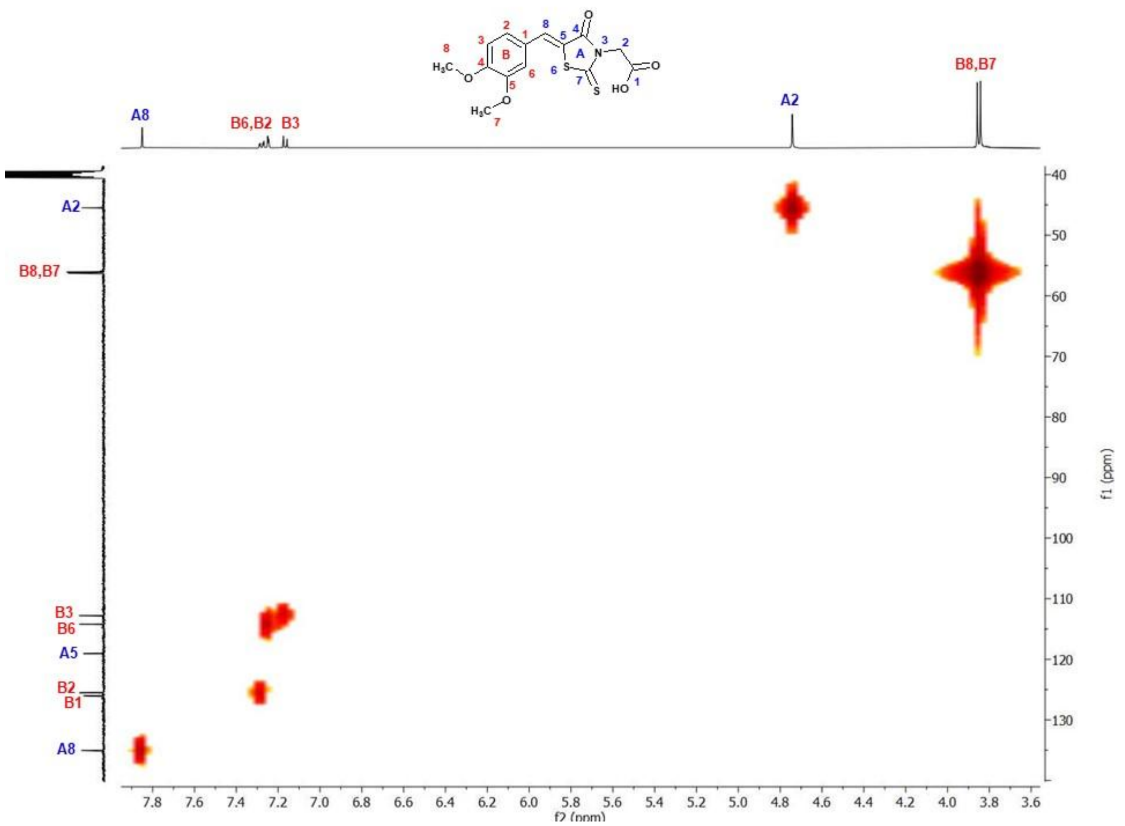

H-C HMQC of C-2

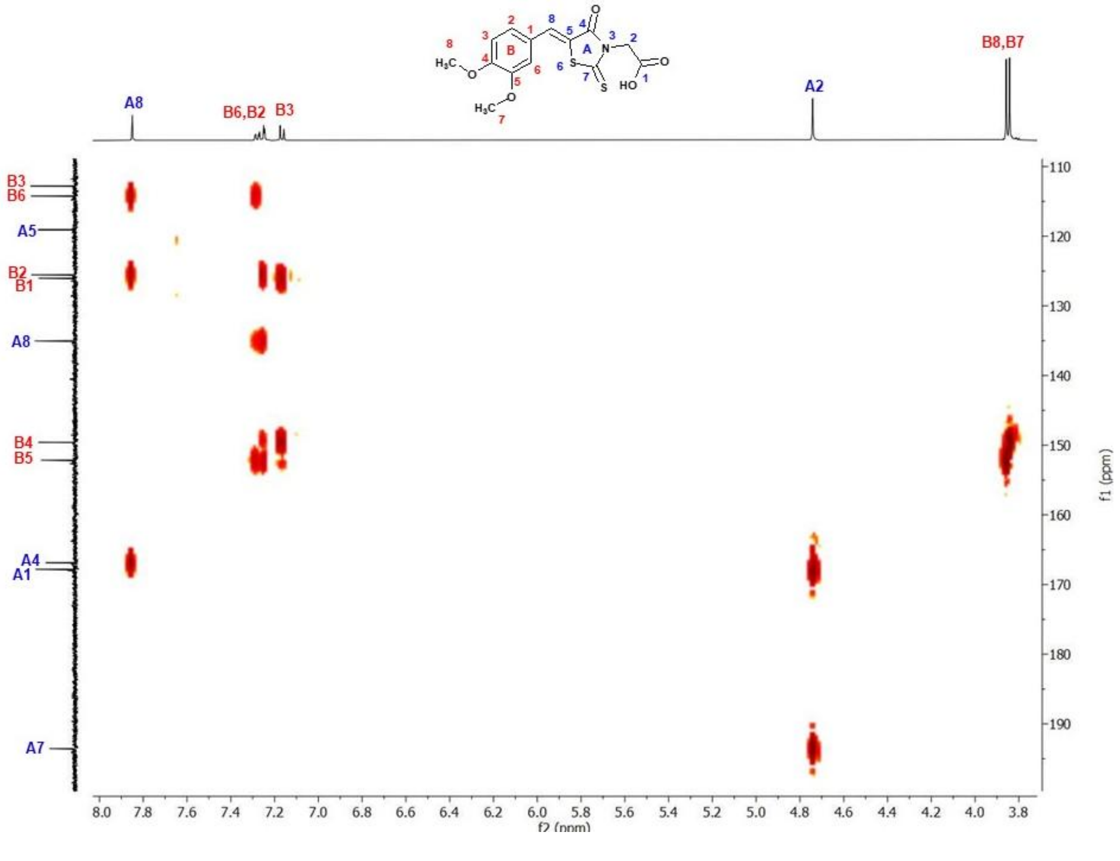

H-C HMBC of C-2

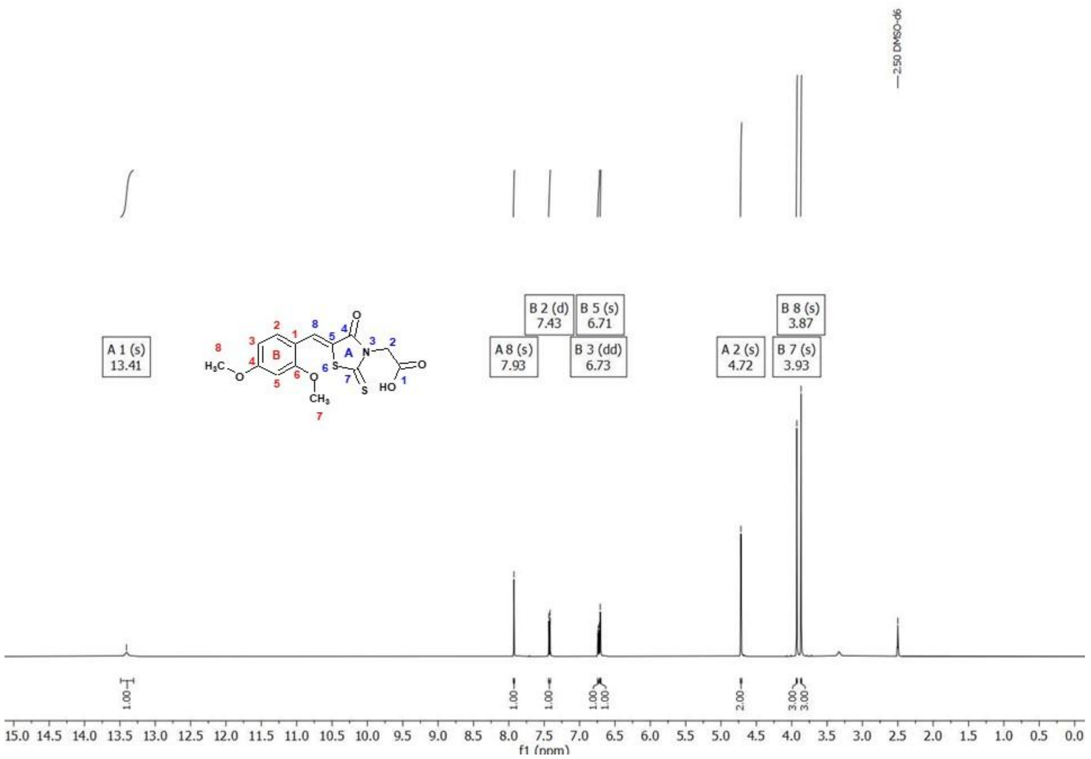

^{1}H NMR of C-3

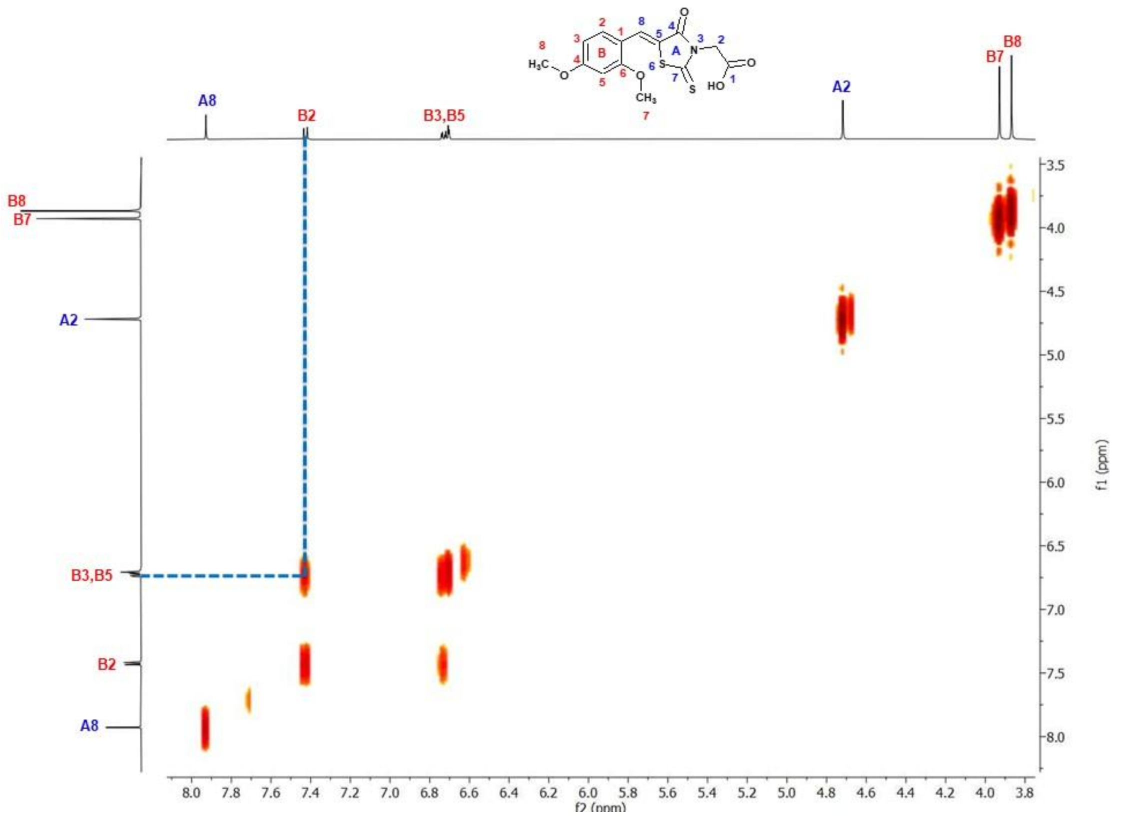

H-H COSY of C-3

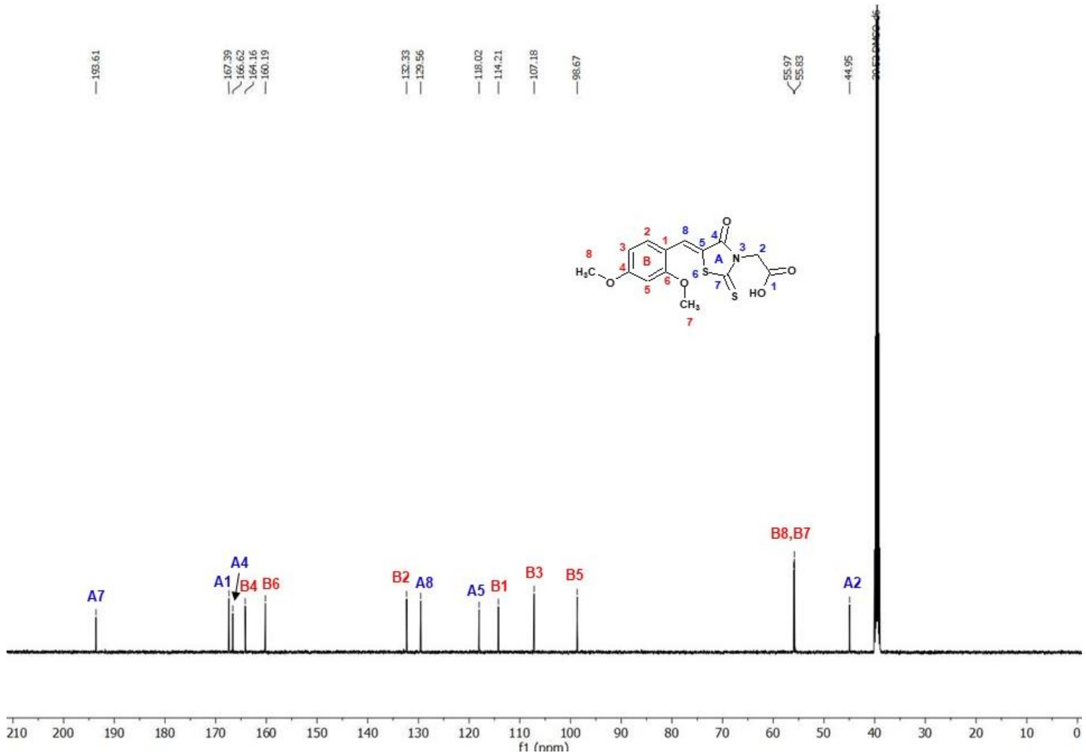

^{13}C NMR of C-3

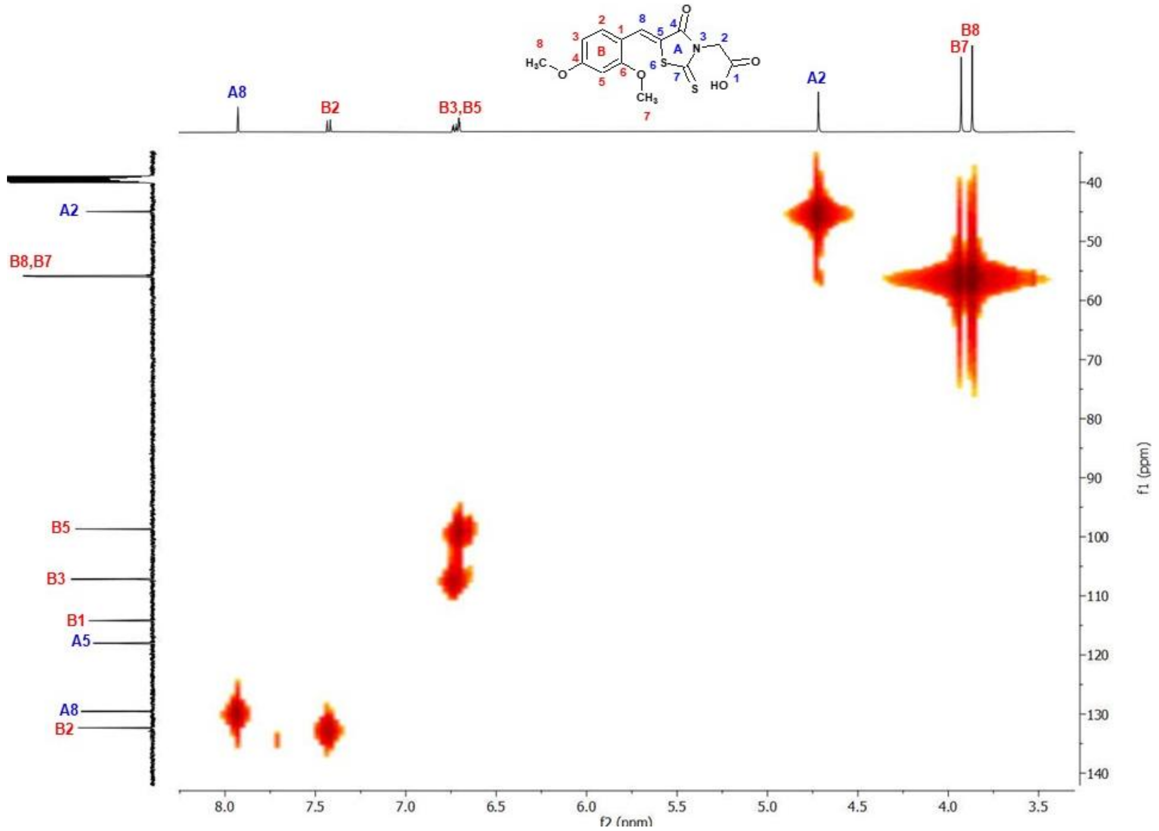

H-C HMQC of C-3

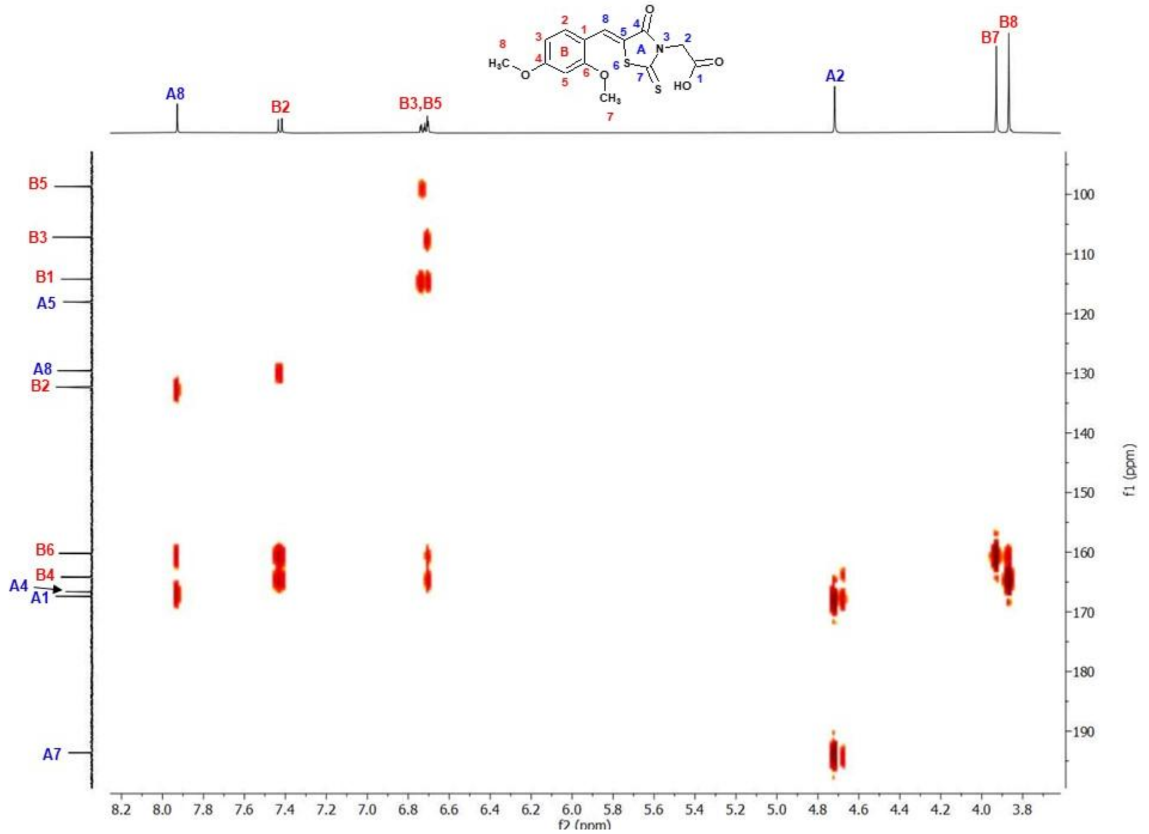

H-C HMBC of C-3

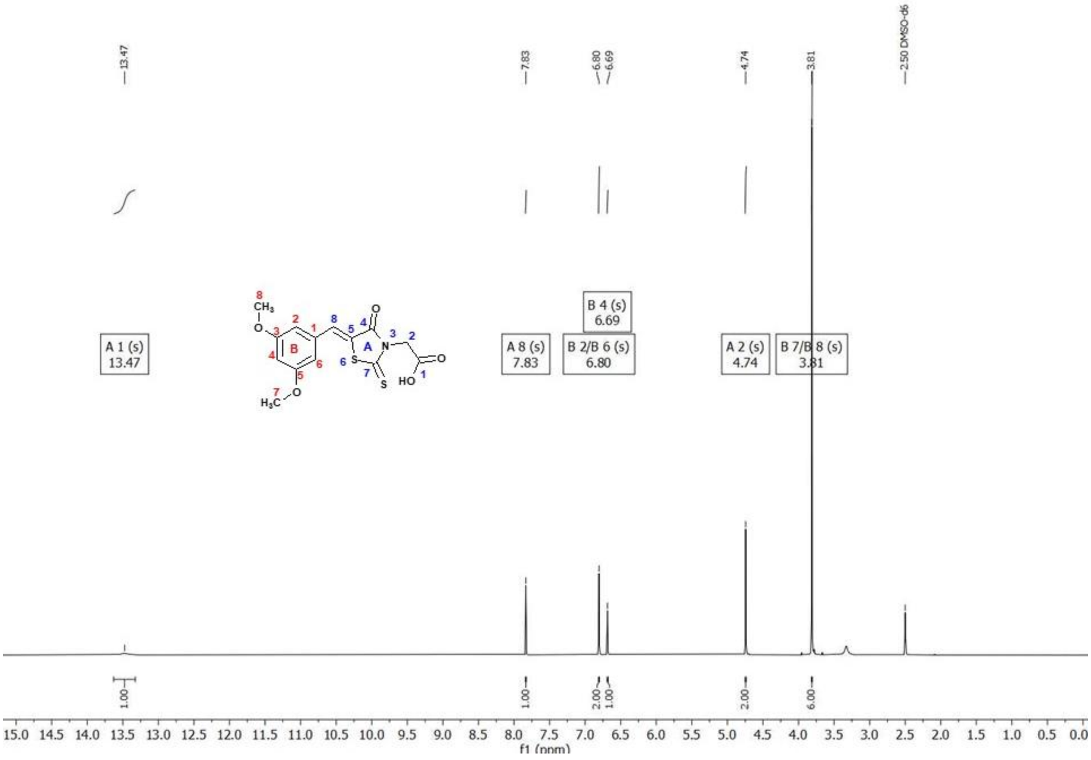

^{1}H NMR of C-4

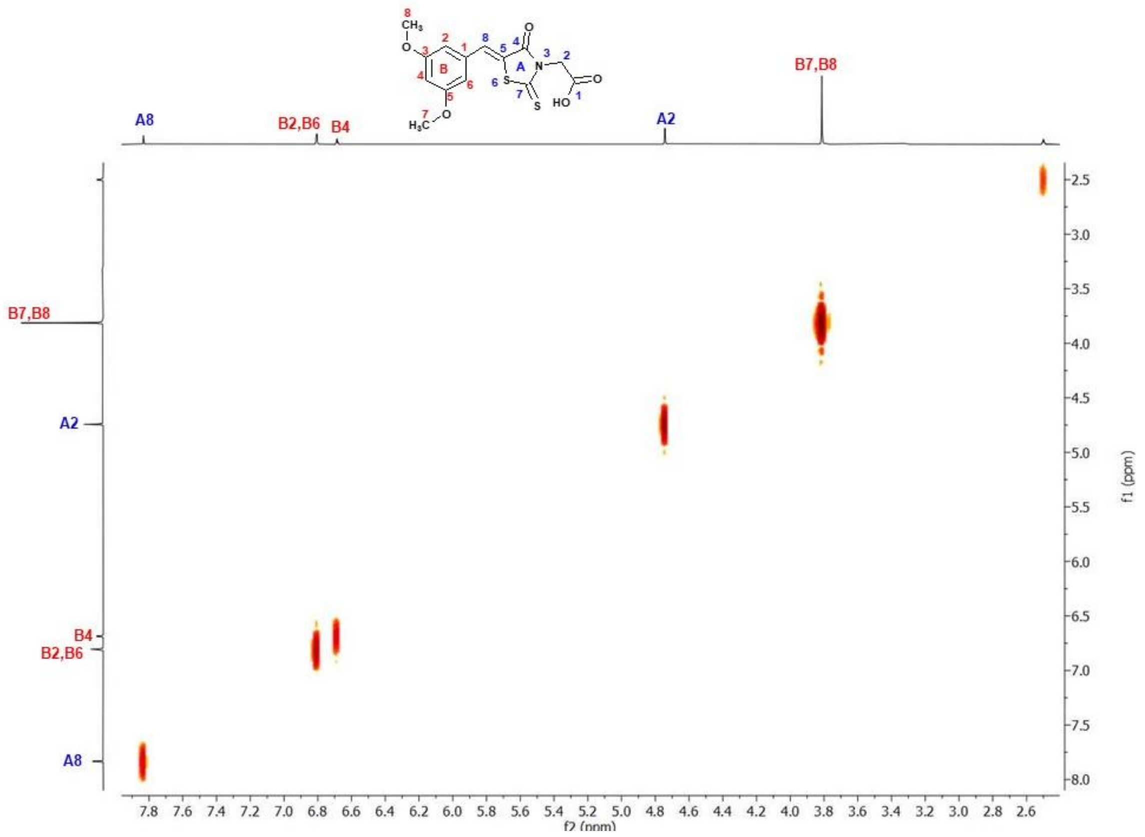

H-H COSY of C-4

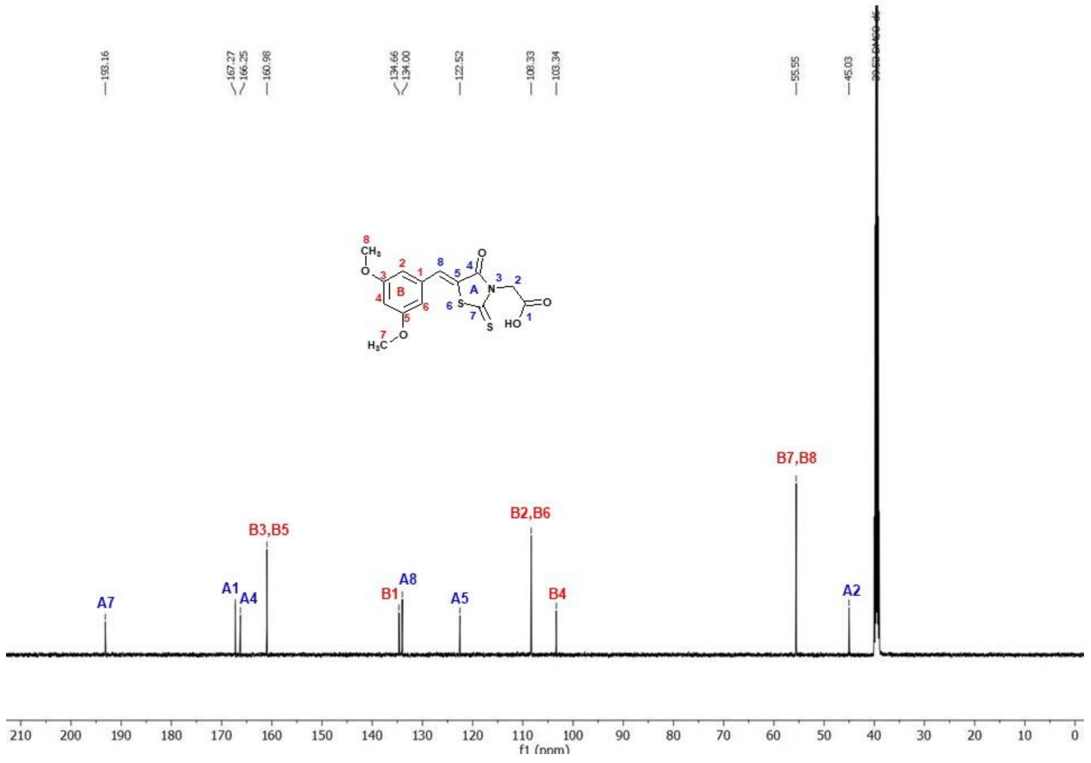

^{13}C NMR of C-4

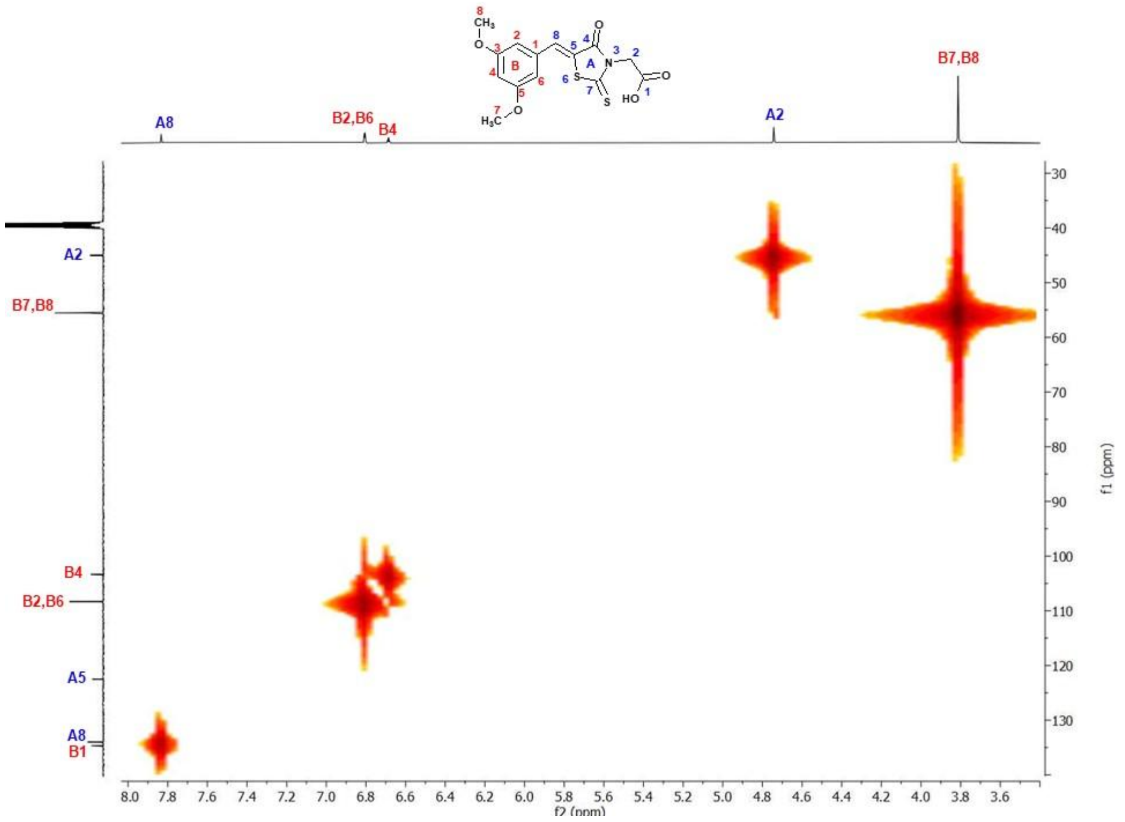

H-C HMQC of C-4

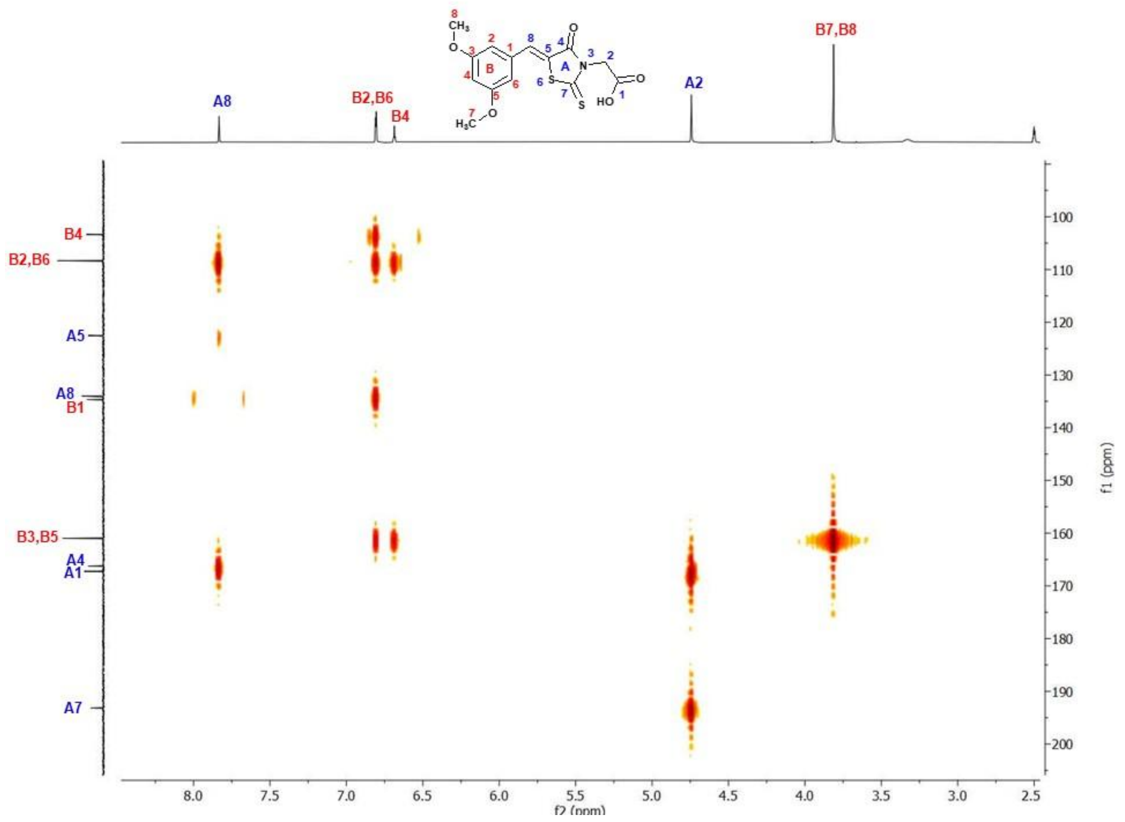

H-C HMBC of C-4

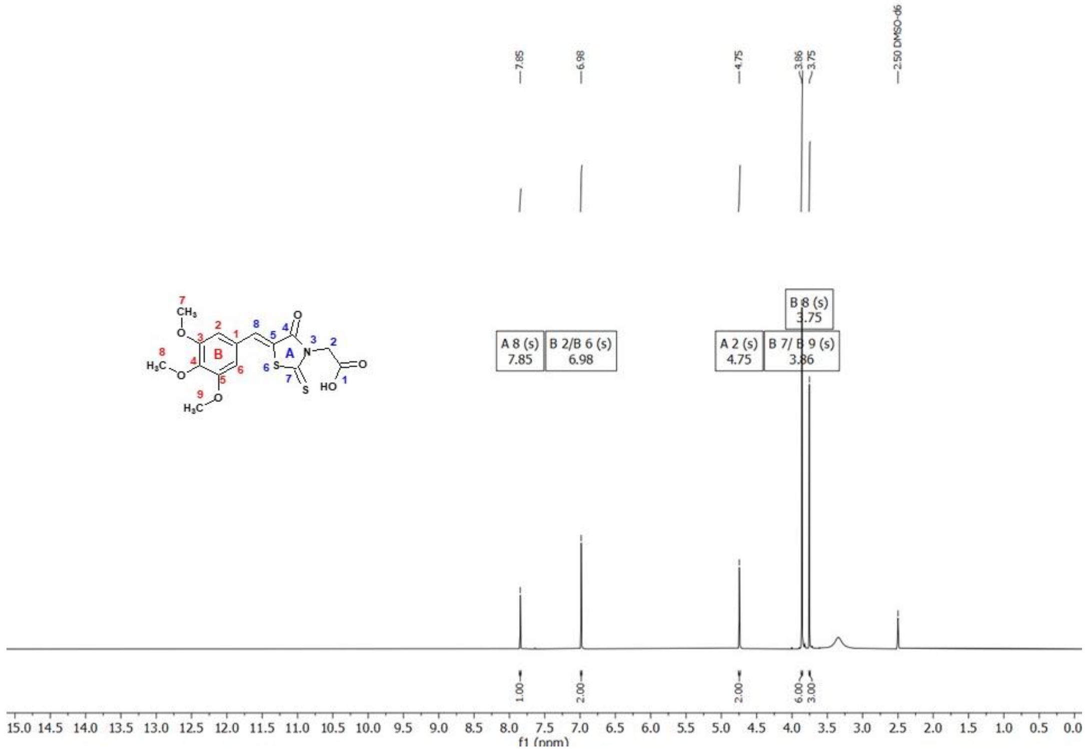

^{1}H NMR of C-5

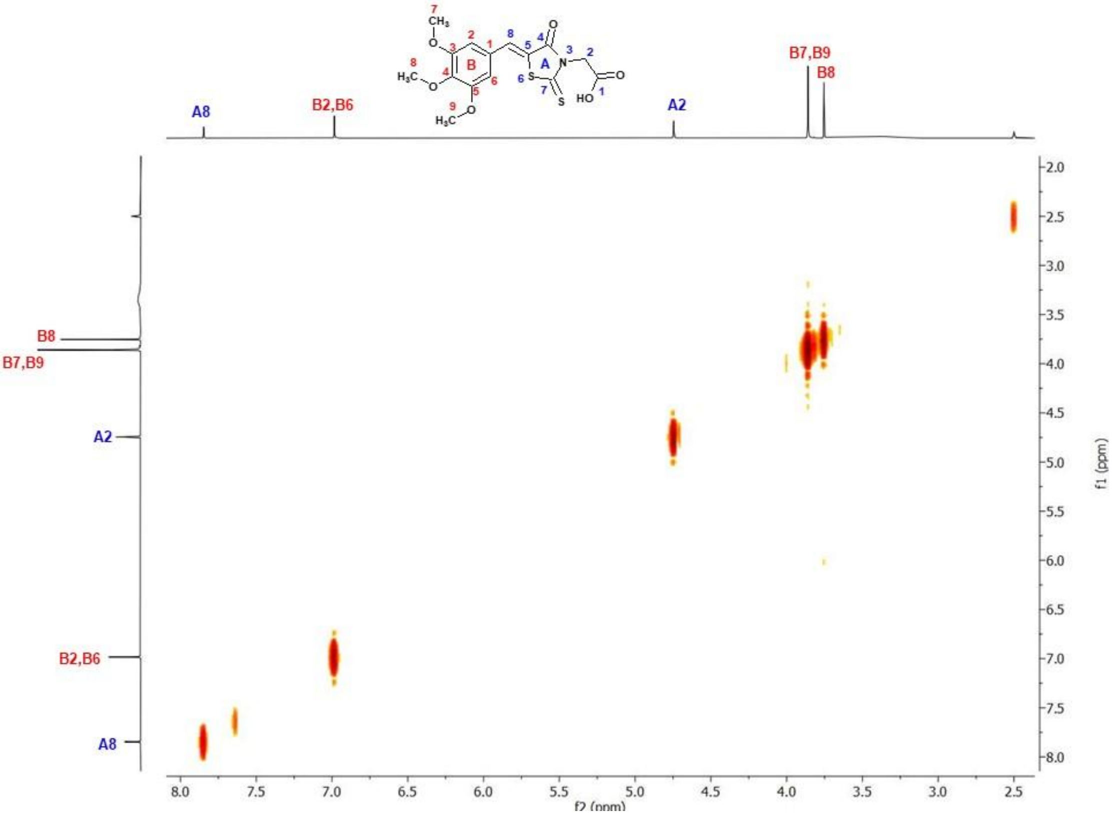

H-H COSY of C-5

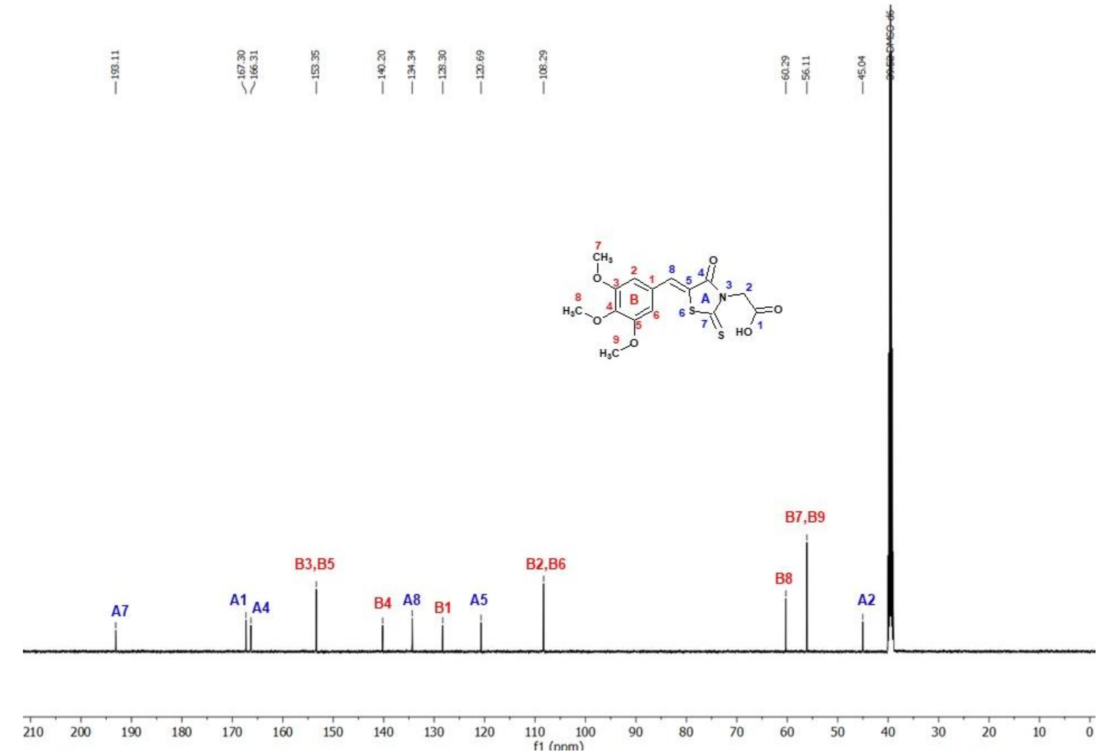

^{13}C NMR of C-5

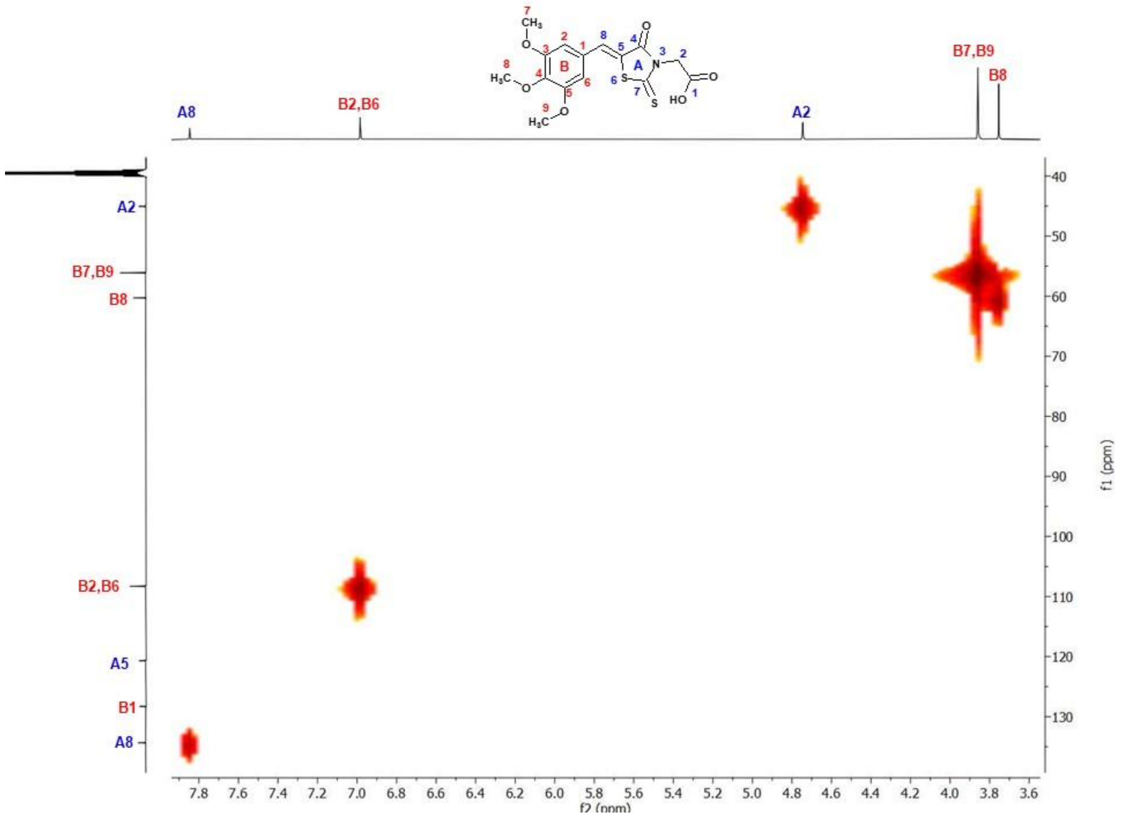

H-C HMQC of C-5

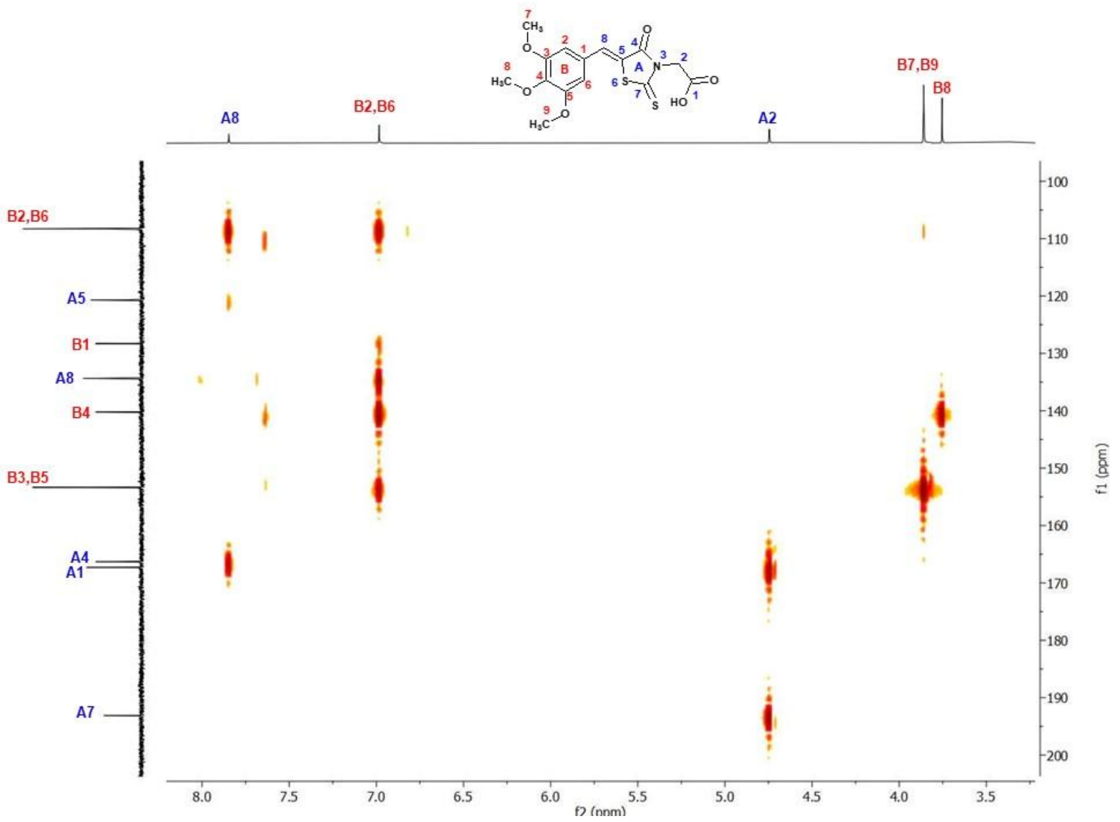

H-C HMBC of C-5

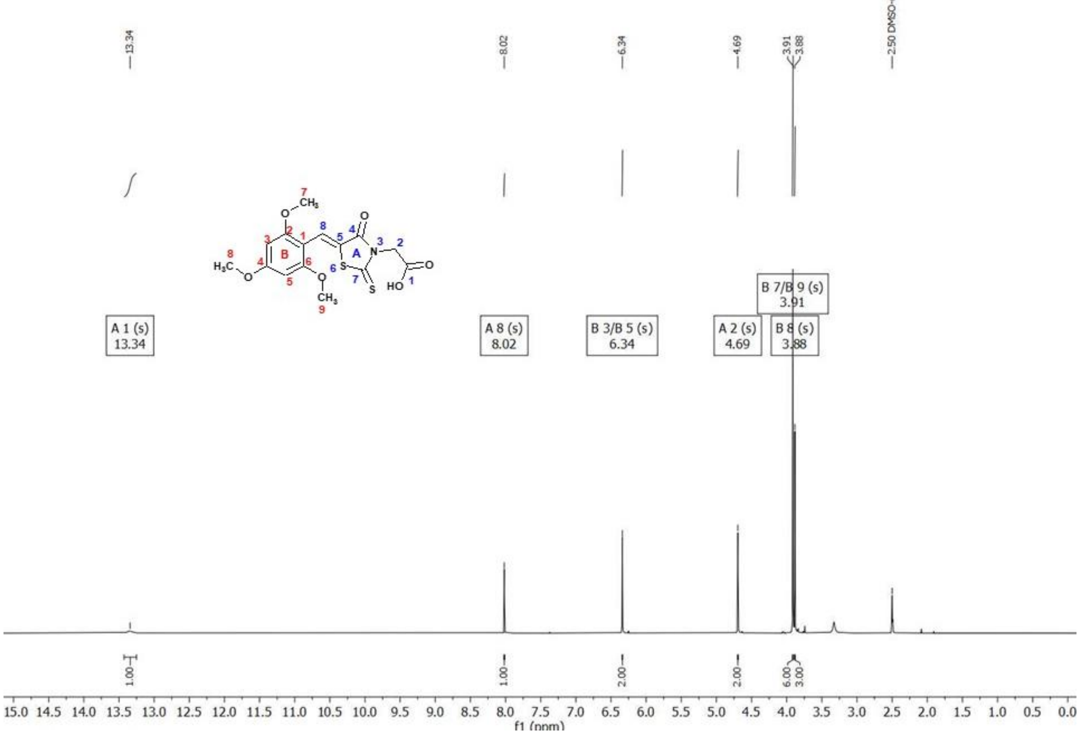

^{1}H NMR of C-6

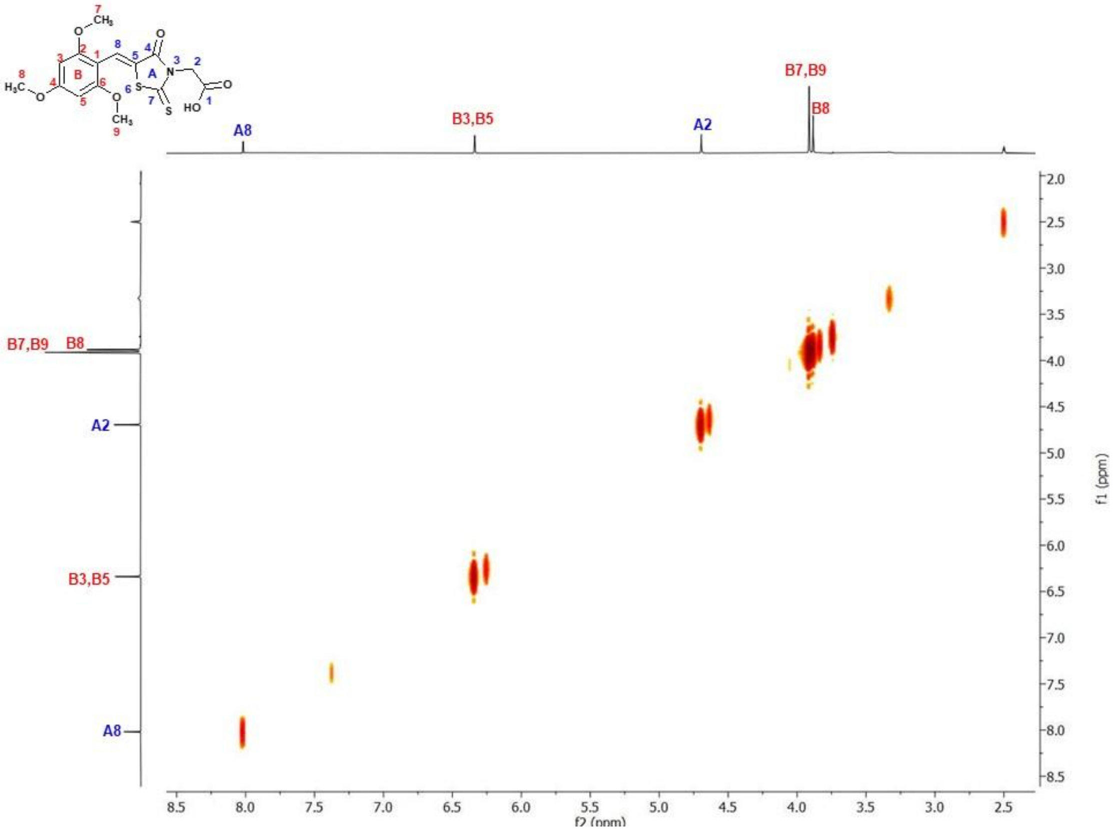

H-H COSY of C-6

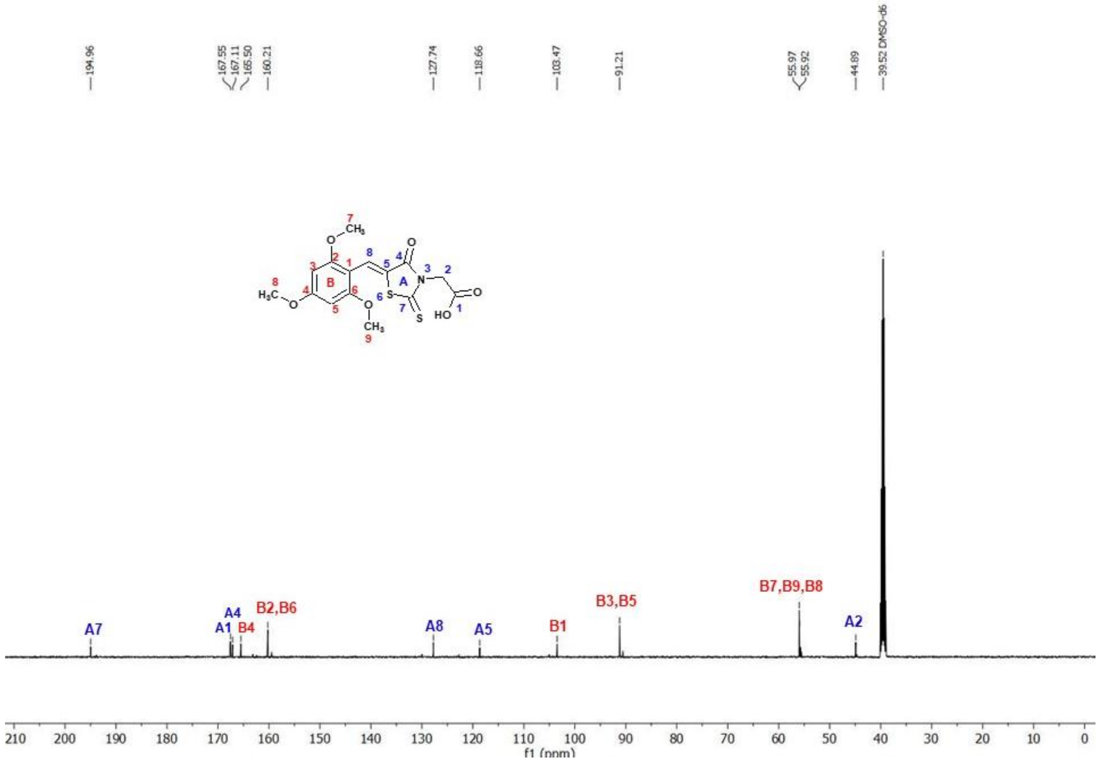

^{13}C NMR of C-6

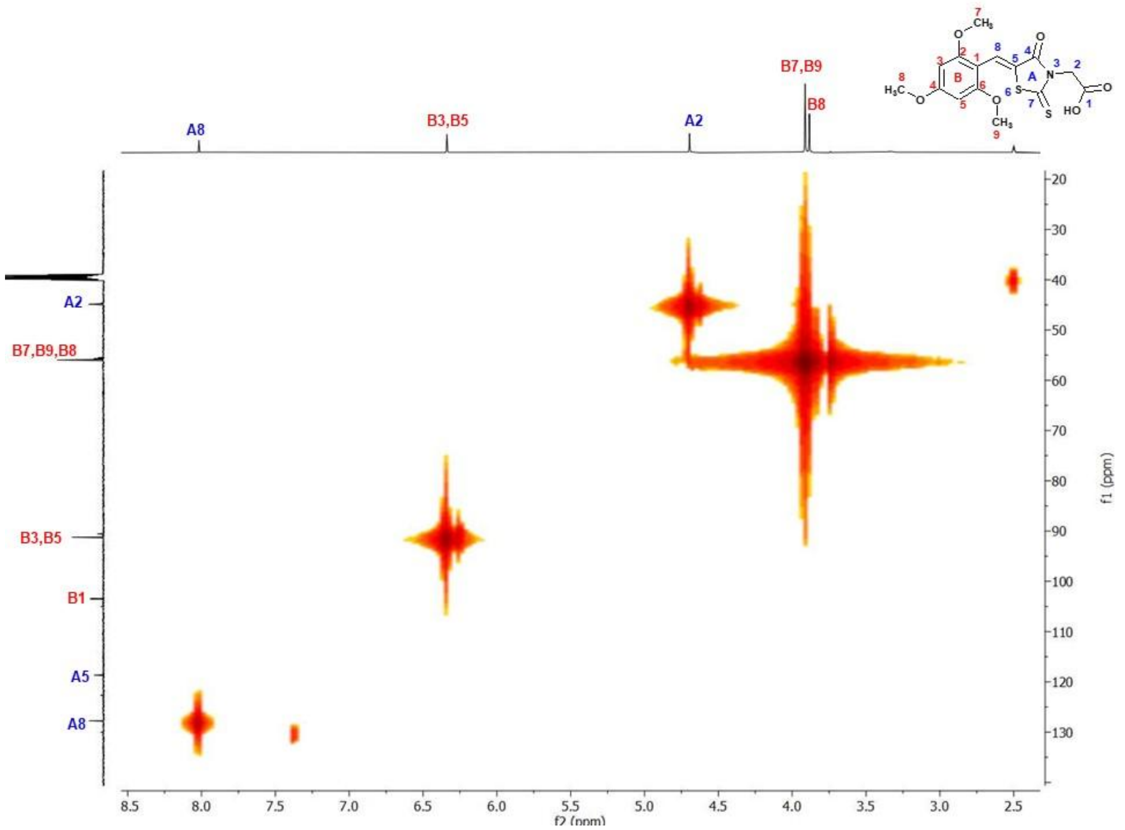

H-C HMQC of C-6

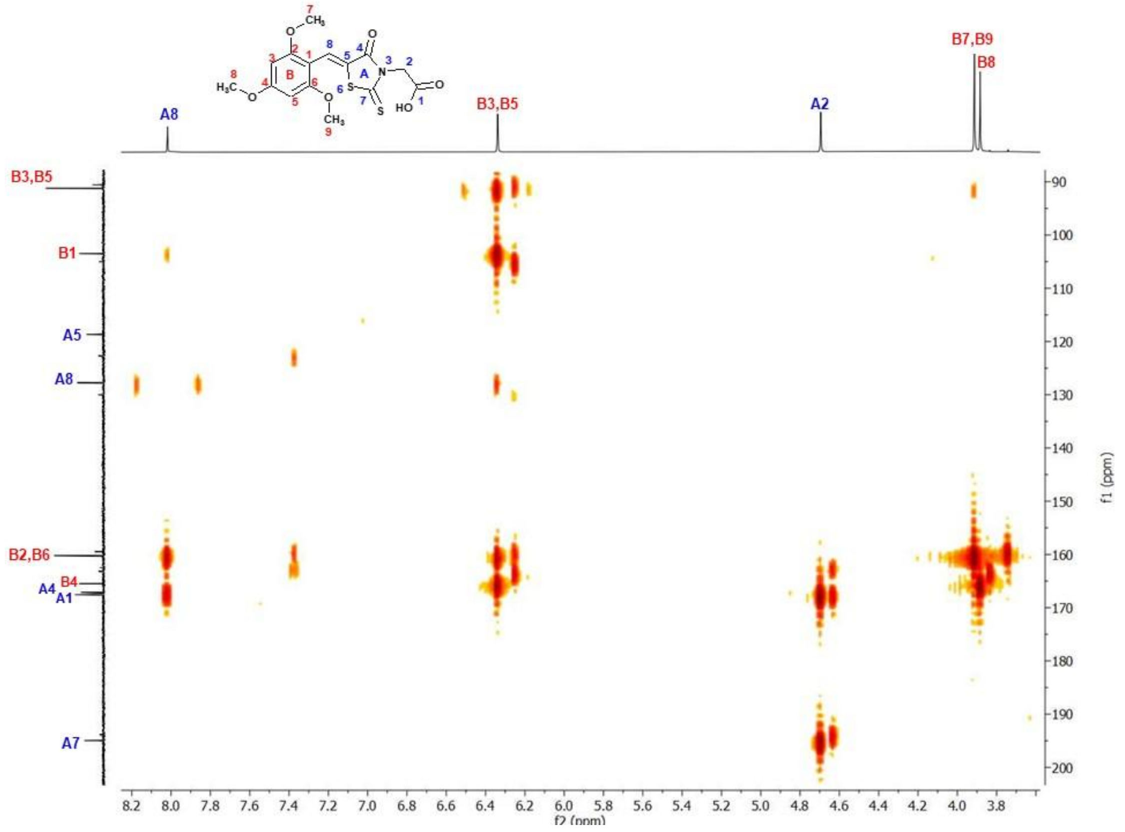

H-C HMBC of C-6

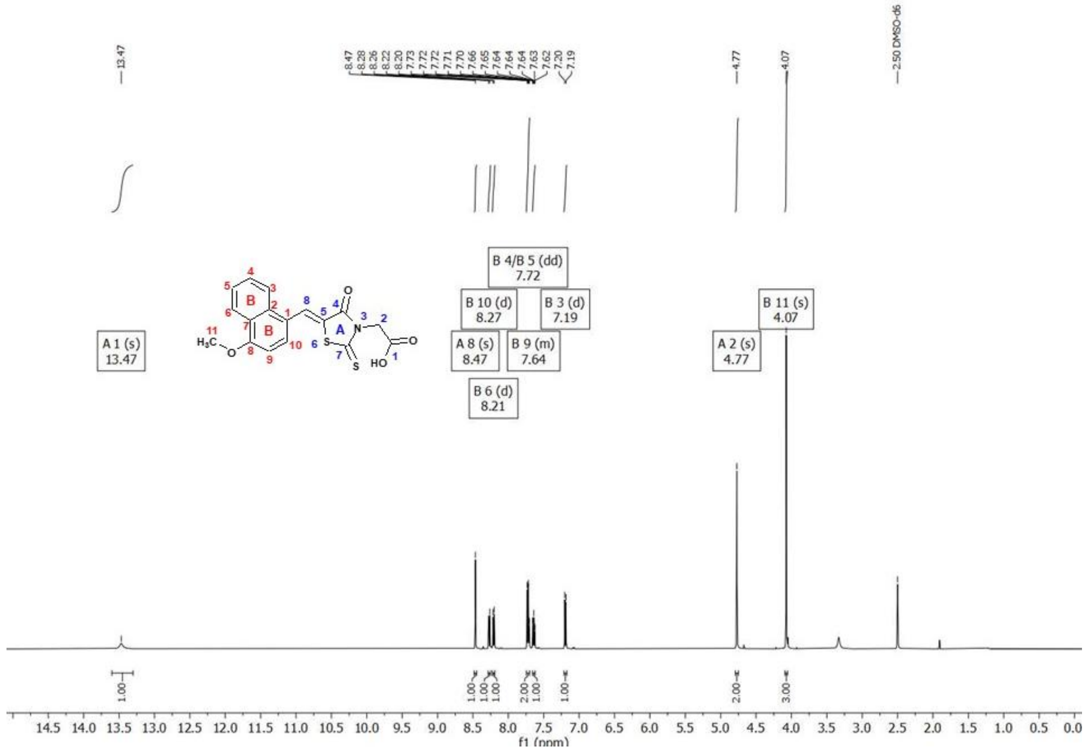

^{1}H NMR of C-7

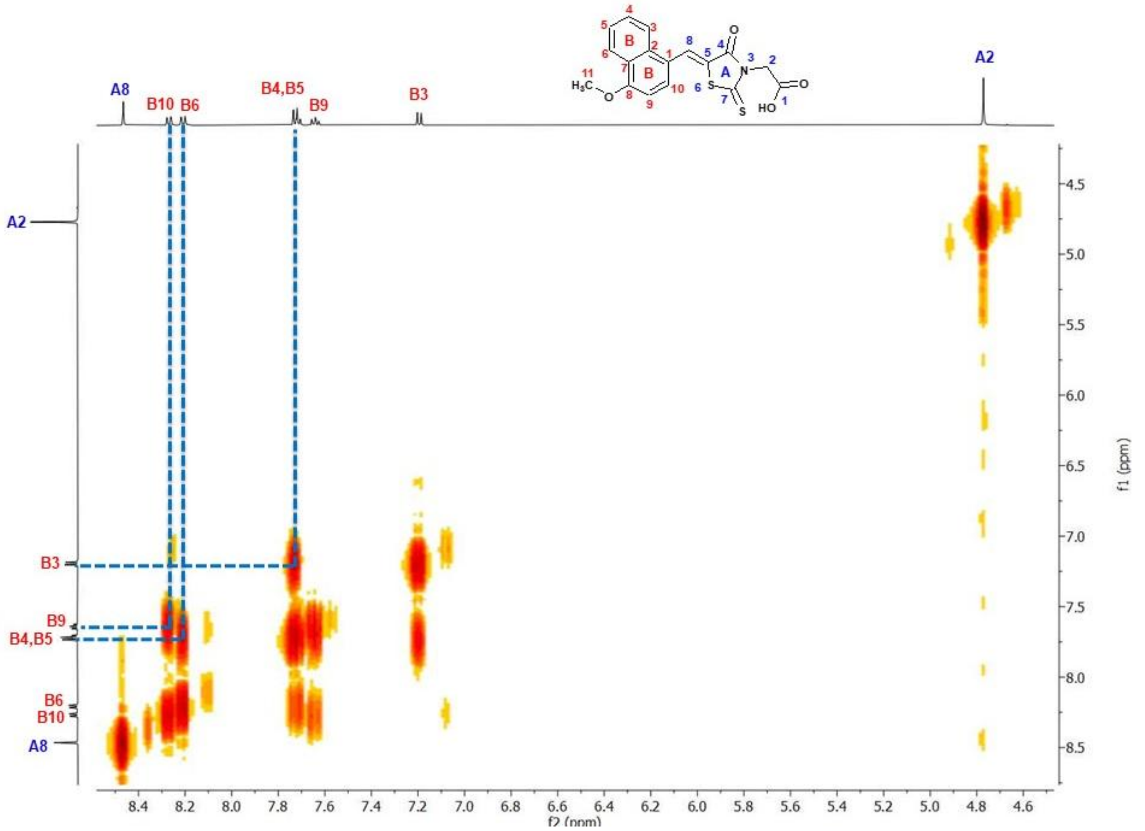

H-H COSY of C-7

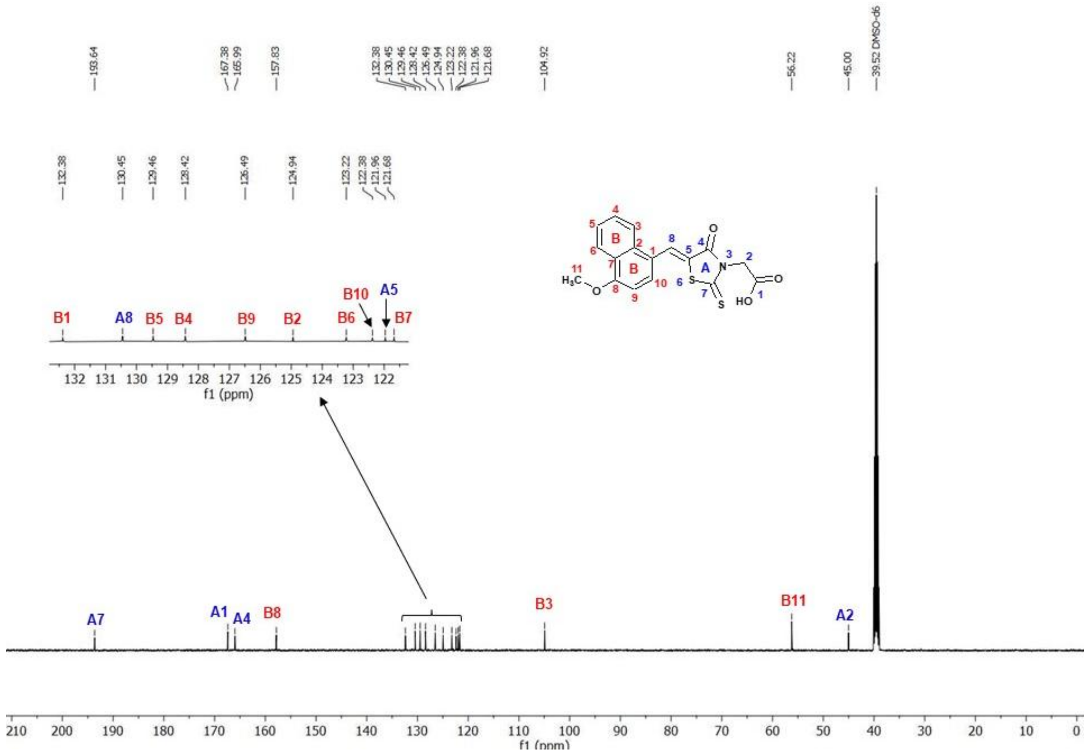

^{13}C NMR of C-7

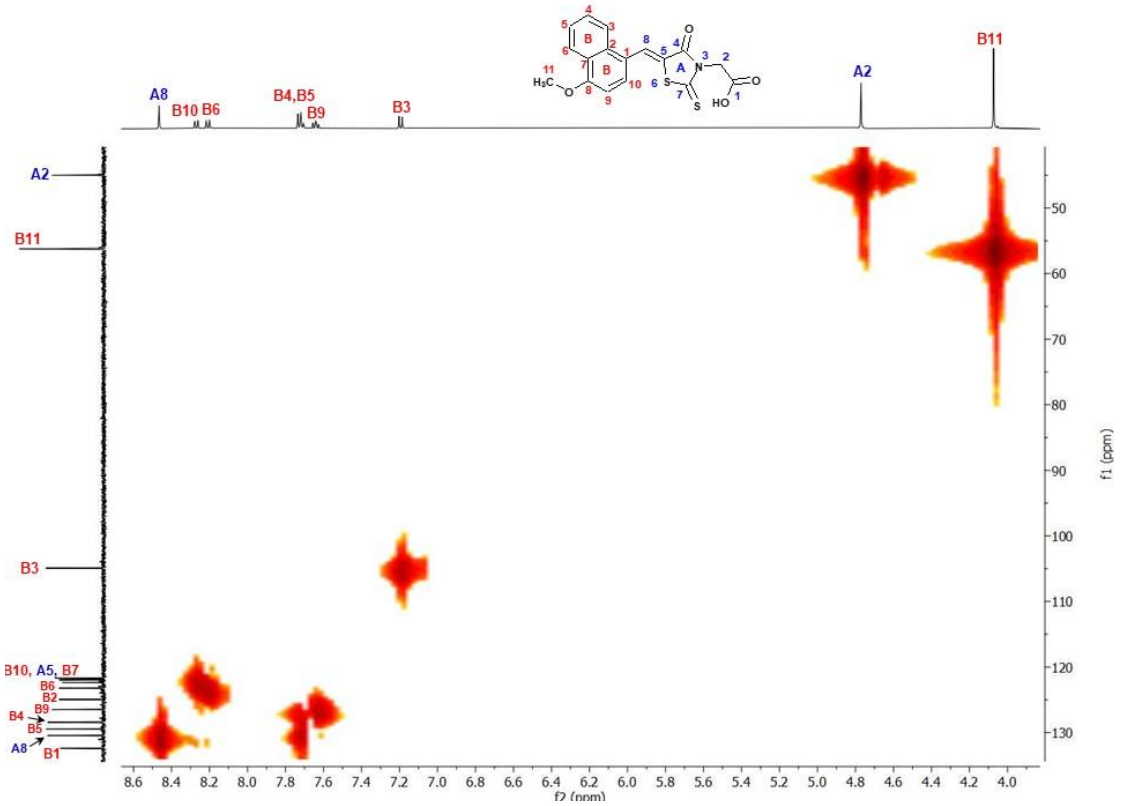

H-C HMQC of C-7

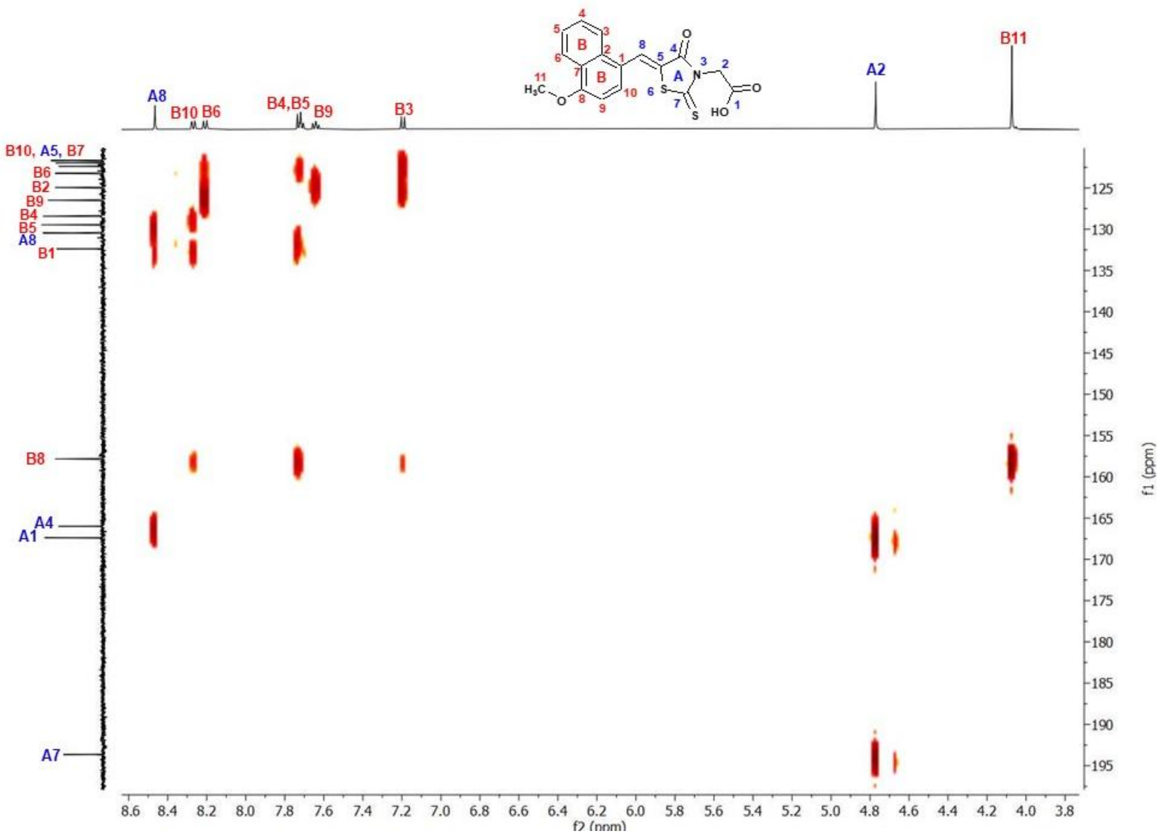

H-C HMBC of C-7

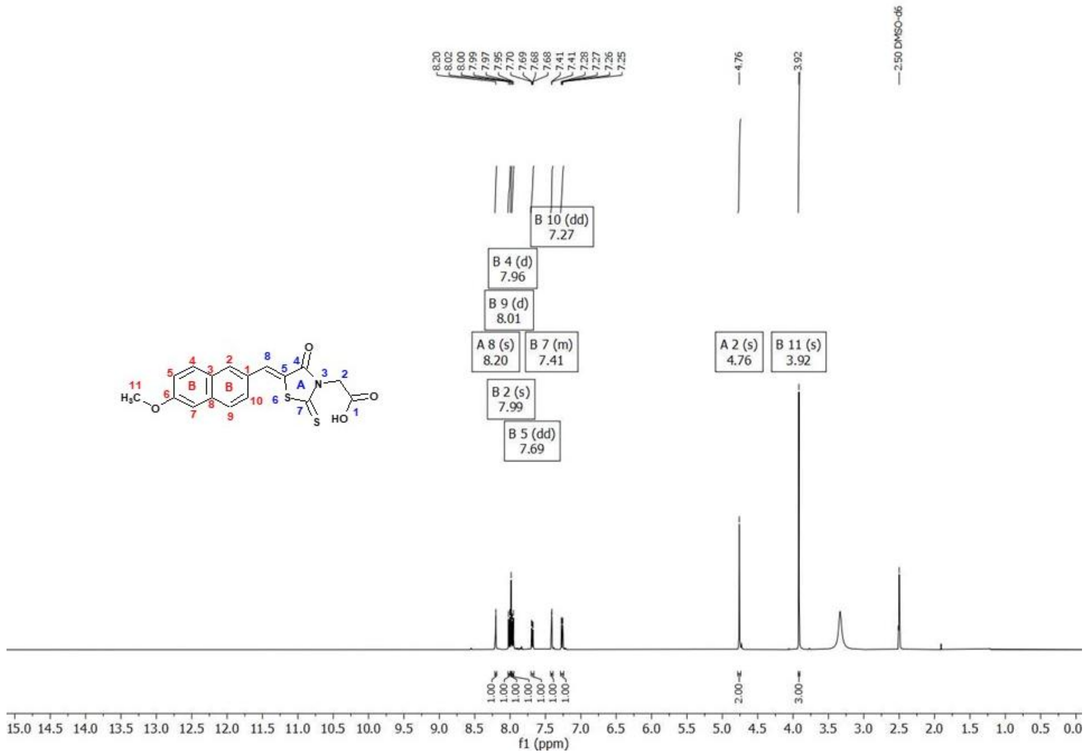

^{1}H NMR of C-8

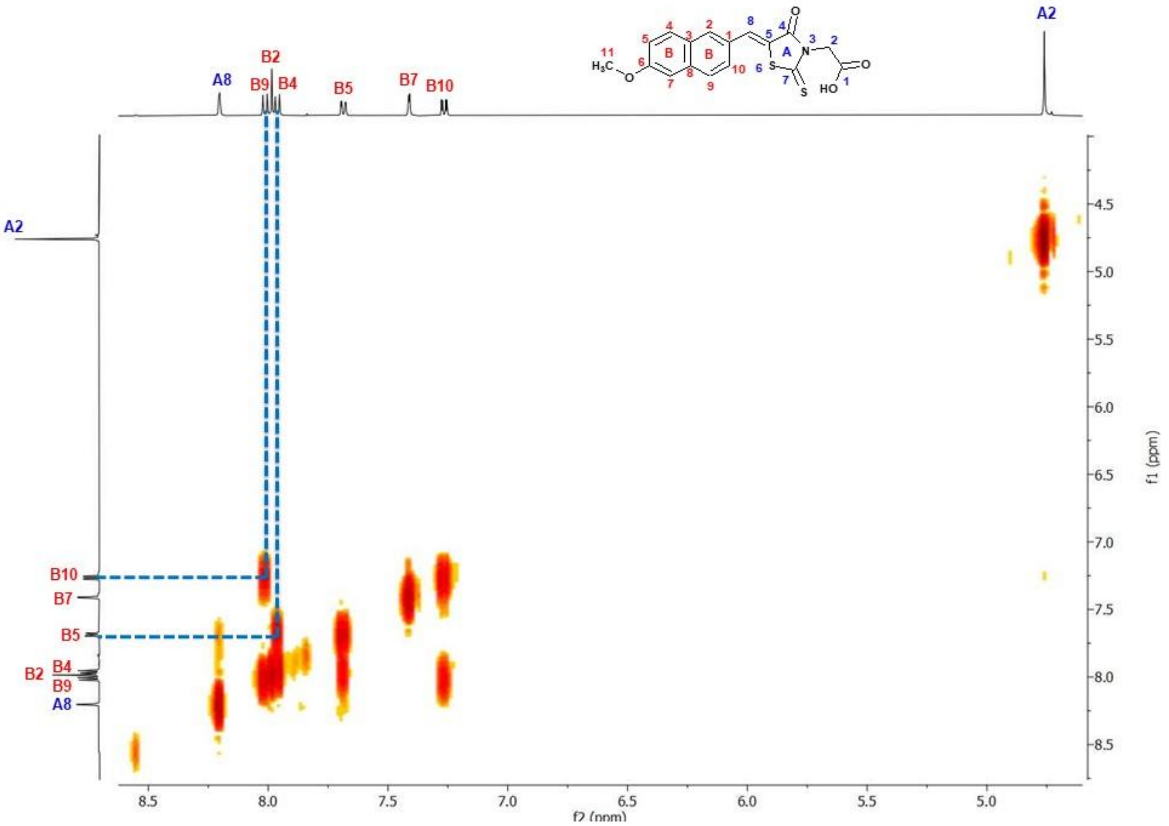

H-H COSY of C-8

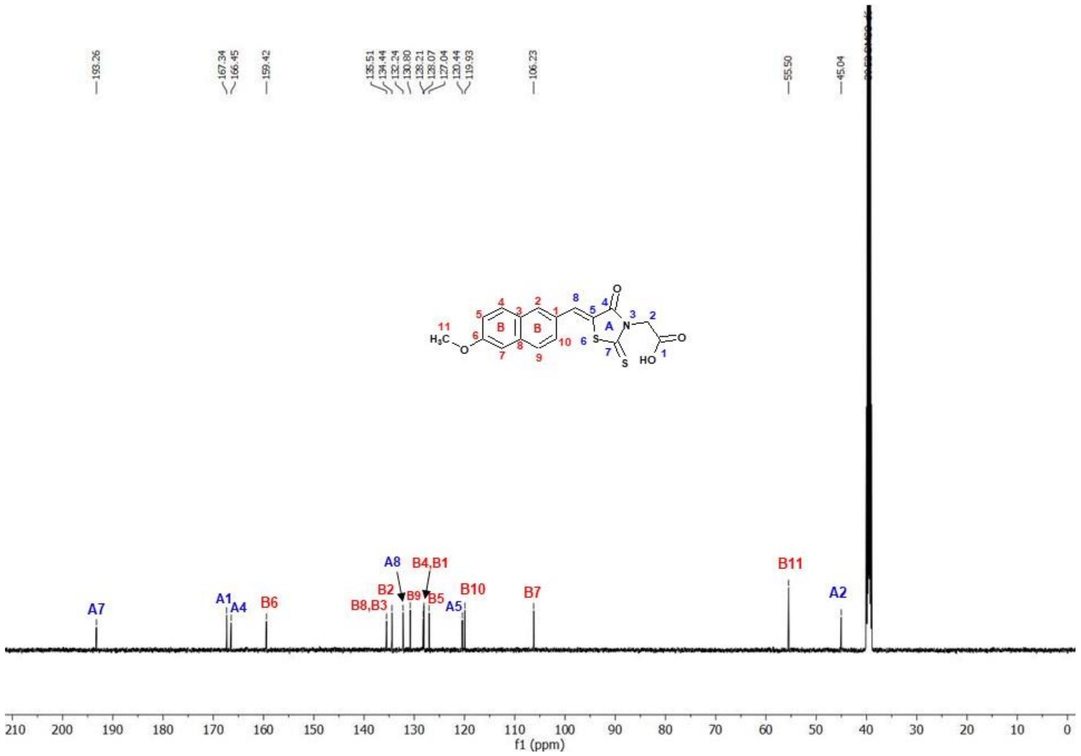

^{13}C NMR of C-8

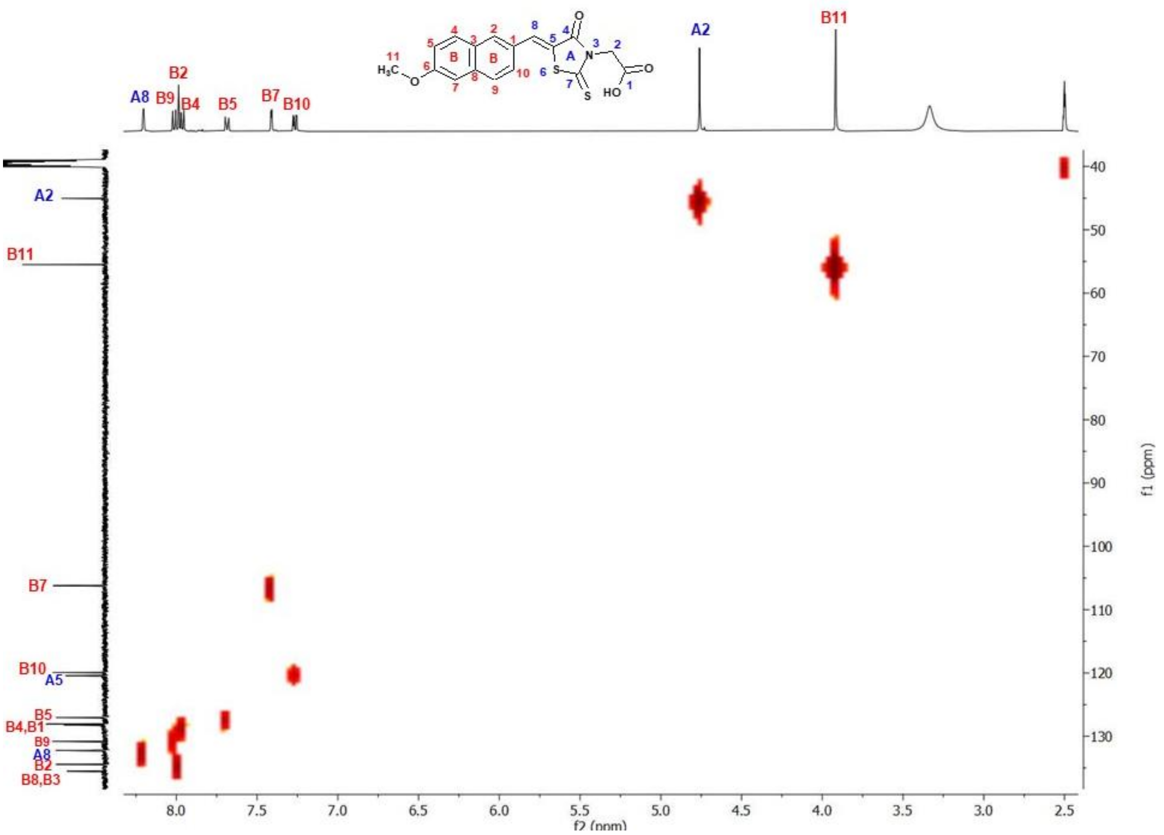

H-C HMQC of C-8

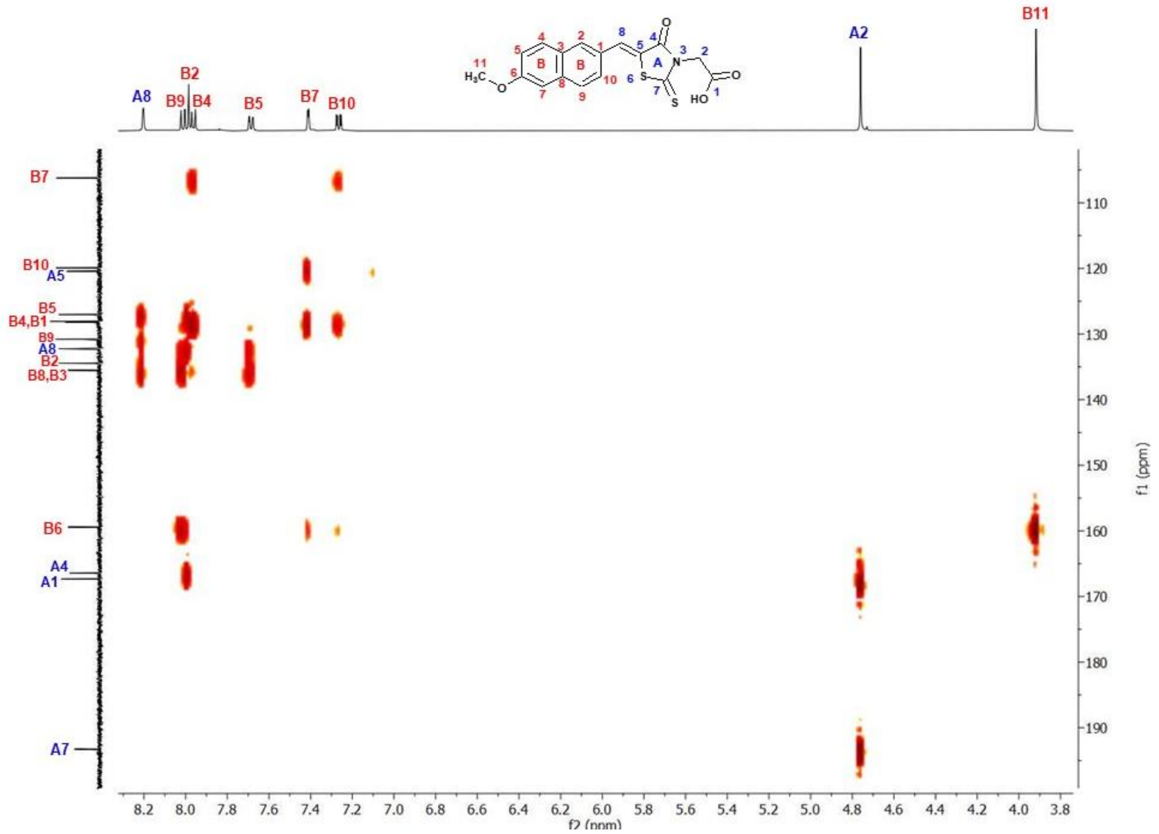

H-C HMBC of C-8

4. Thermal properties

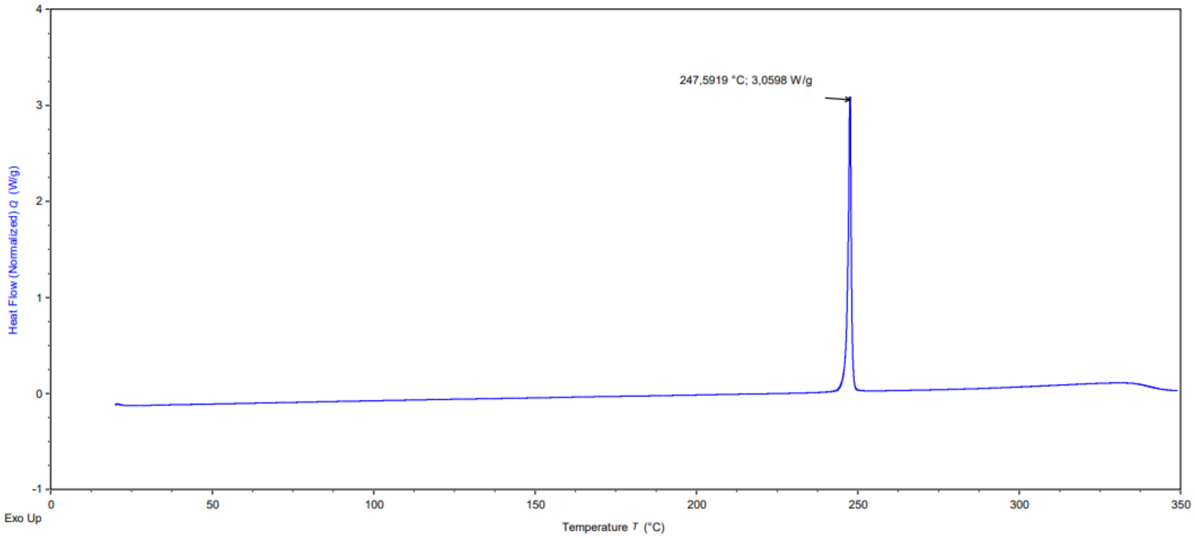

Figure S1. DSC thermograms of **C-1**

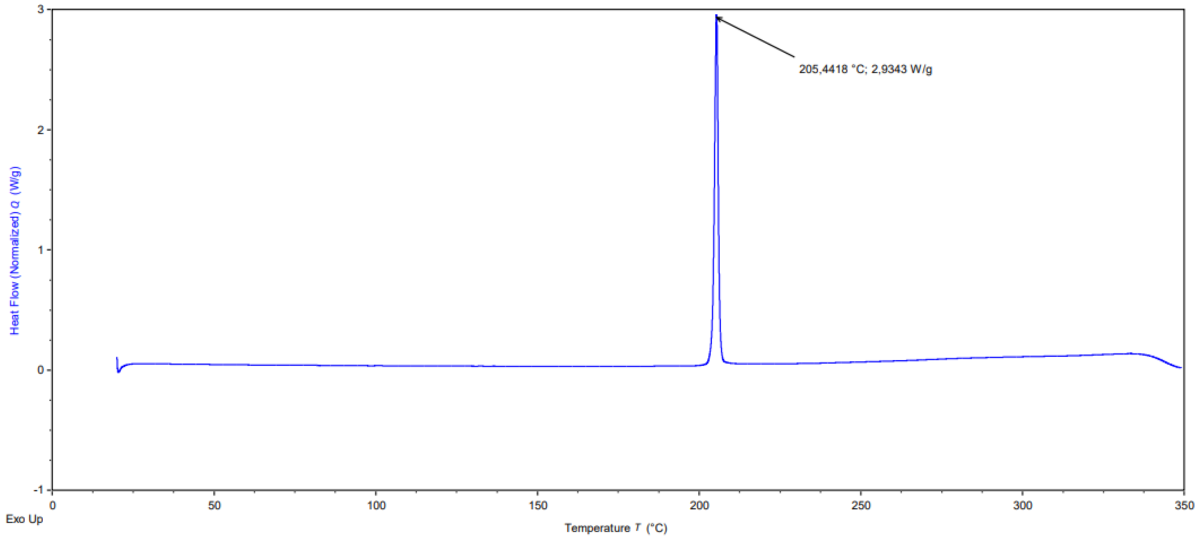

Figure S2. DSC thermograms of **C-2**

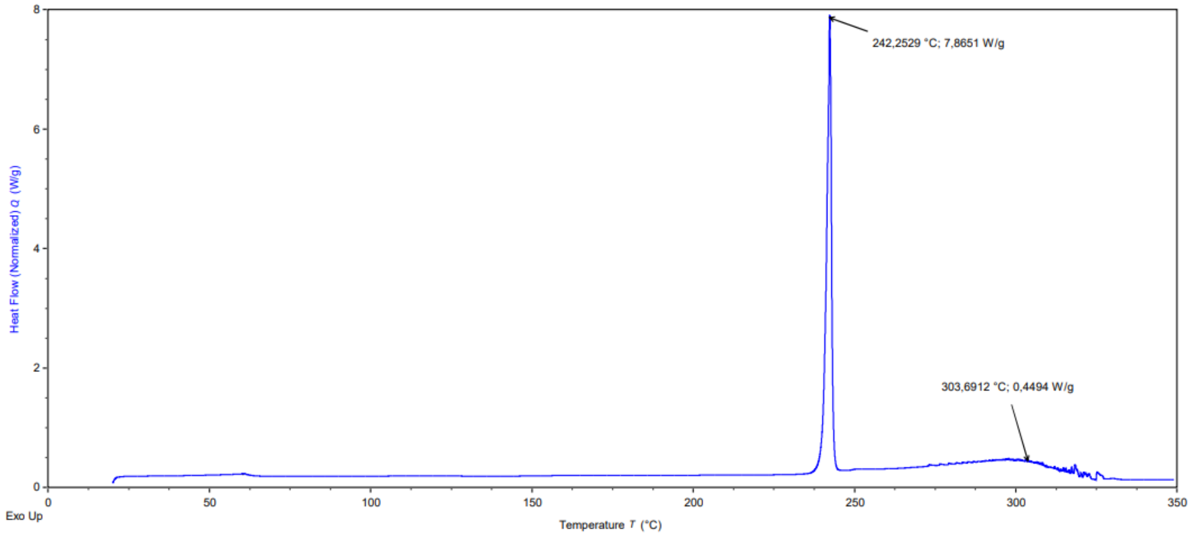

Figure S3. DSC thermograms of **C-3**

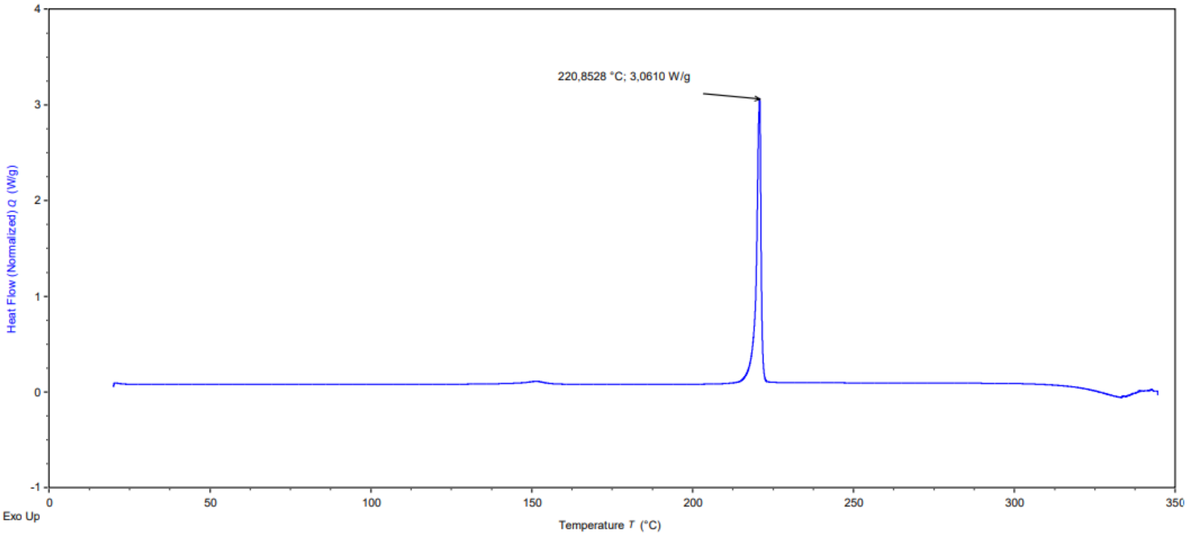

Figure S4. DSC thermograms of **C-4**

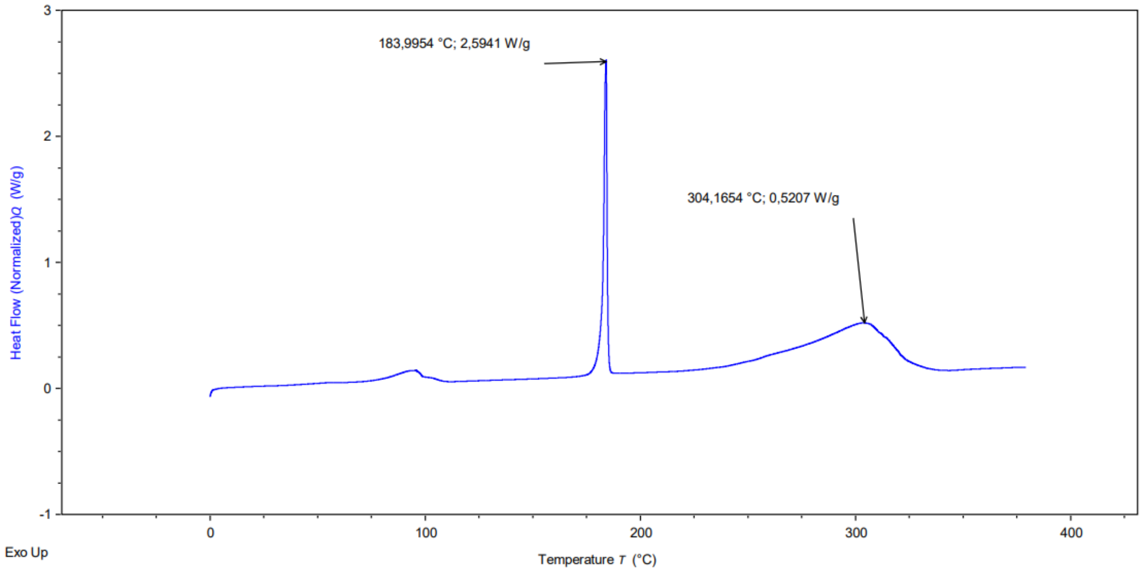

Figure S5. DSC thermograms of **C-5**

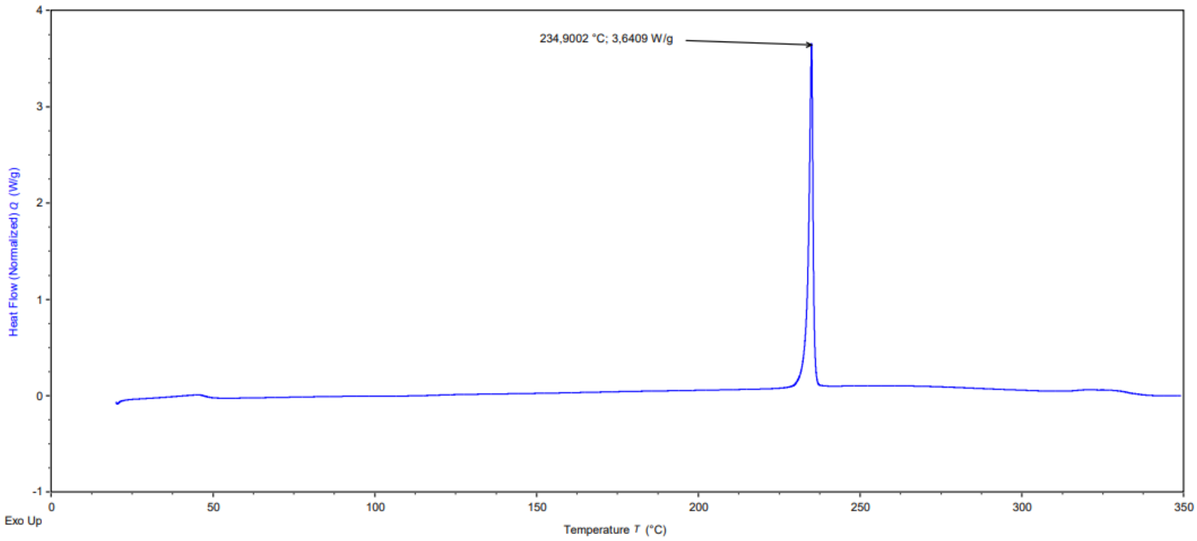

Figure S6. DSC thermograms of **C-6**

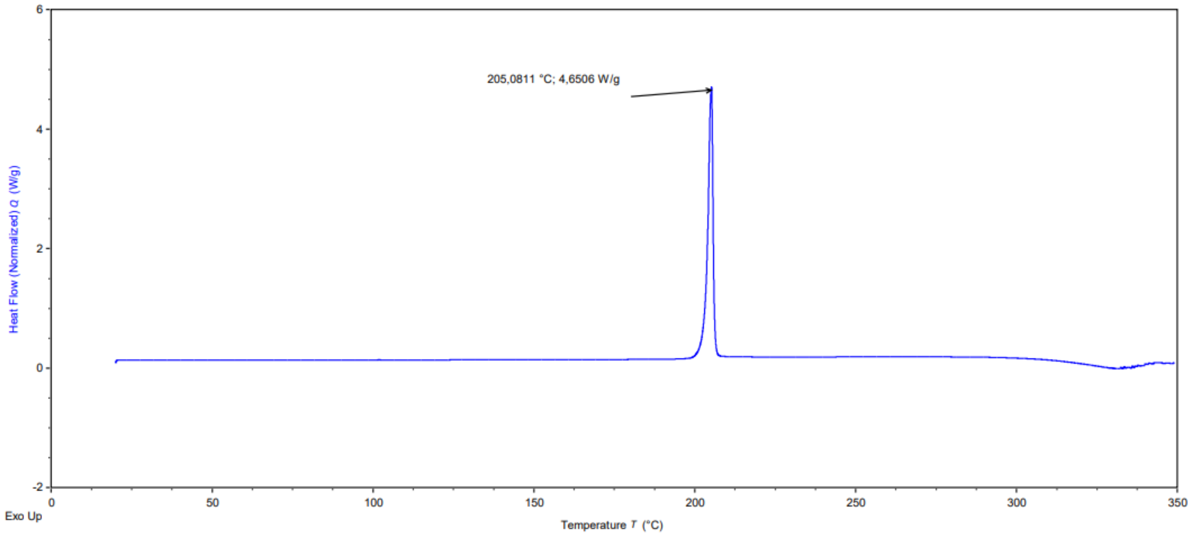

Figure S7. DSC thermograms of **C-7**

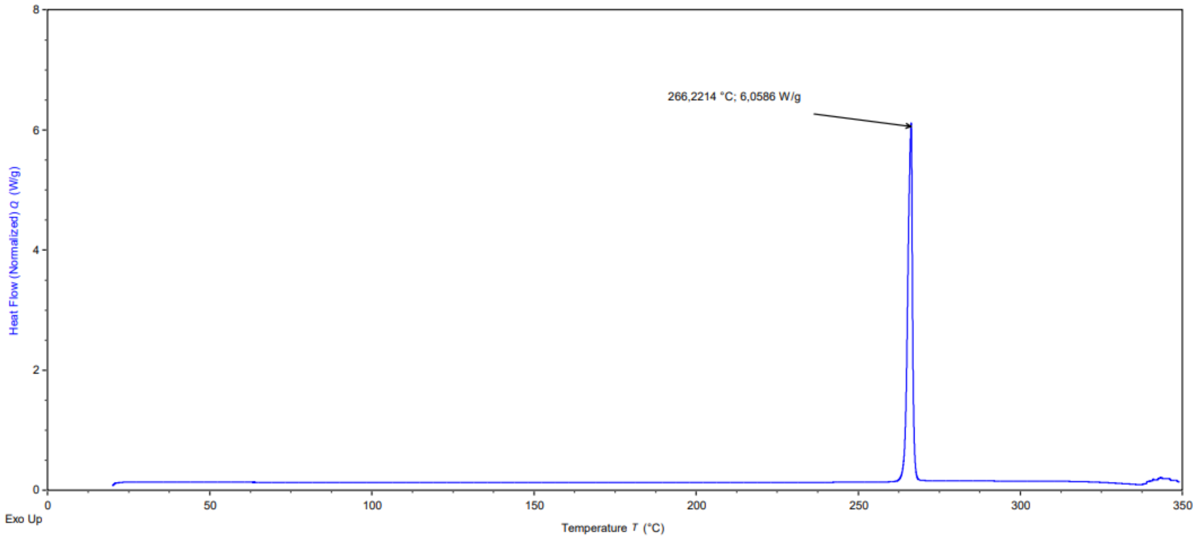

Figure S8. DSC thermograms of **C-8**

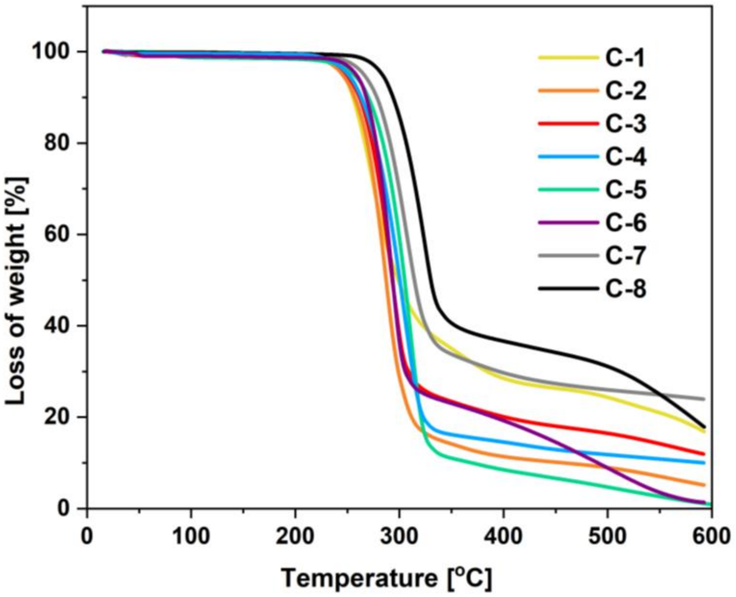

Figure S9. TGA thermogram for **C-1 - C-8**

5. DFT calculations

Theoretical calculations - methodology

All geometrical parameters of investigated molecules in their ground (S_{GS}) and excited (S_{CT}) states were calculated using density functional theory (DFT) approach implemented in Gaussian 16 program package [6] with PBE0/6-311++G(d,p) basis set. In order to verify that all the structures correspond to the minima on the potential energy surface, an analysis of Hessians was performed. The electronic properties were characterized by computations of the vertical absorption, and were obtained using the time-dependent density functional theory (TDDFT/PBE0) [7] and by including the state-specific (SS) corrected linear response (cLR) approach [8]. Based on previous experience with derivatives of this type [9-11], all calculations of linear and nonlinear optical properties were performed using the PBE0 functional.

The dipole moments and polarities of the charge-transfer state (CT) were evaluated by numerical differentiation of the excitation energies (*E*) in the presence of an electric field *F* of 0.001 a.u. strength:

$$\Delta\mu^{i}_{CT-GS} = \frac{E_{CT}(+F^{i})-E_{CT}(-F^{i})}{-2F^{i}} - \frac{E_{GS}(+F^{i})-E_{GS}(-F^{i})}{-2F^{i}} \quad (1)$$

where *i* stands for the Cartesian component of the dipole moment difference.

The isotropic average polarizability (α) and first-order hyperpolarizability (β) were determined based on the Gaussian 16 program and defined as:

$$\alpha = \frac{\alpha_{xx}+\alpha_{yy}+\alpha_{zz}}{3} \quad (2)$$

$$\beta = \sum_{i=x,y,z} \frac{\mu_i \beta_i}{|\mu|} \quad (3)$$

where $\beta_i (i = x, y, z)$ is given by $\beta_i = \left(\frac{1}{3}\right) \sum_{j=x,y,z} \left(\beta_{ijj} + \beta_{jij} + \beta_{jji}\right)$

The density differences were obtained at the PBE0/6-311++G(d,p) level and are represented with a contour threshold of 0.02 a.u. In these graphs, the blue (purple) zones indicate density decrease (increase) upon electronic transition. The charge transfer parameters, namely the charge-transfer distance (D_{CT}) and the amount of transferred charge (q_{CT}), have been determined following a Le Bahers' procedure [12].

The solvent effect on the linear and nonlinear optical properties has been taken into account using the Integral Equation Formalism for the Polarizable Continuum Model (IEF–PCM) [13,14].

The biological activities were simulated using a combination of the 3D/4D QSAR BiS/MC and CoCon algorithms [15-17].

Figure S10. HOMO / LUMO plots

	C1	C2
LUMO		
HOMO		
	C3	C4

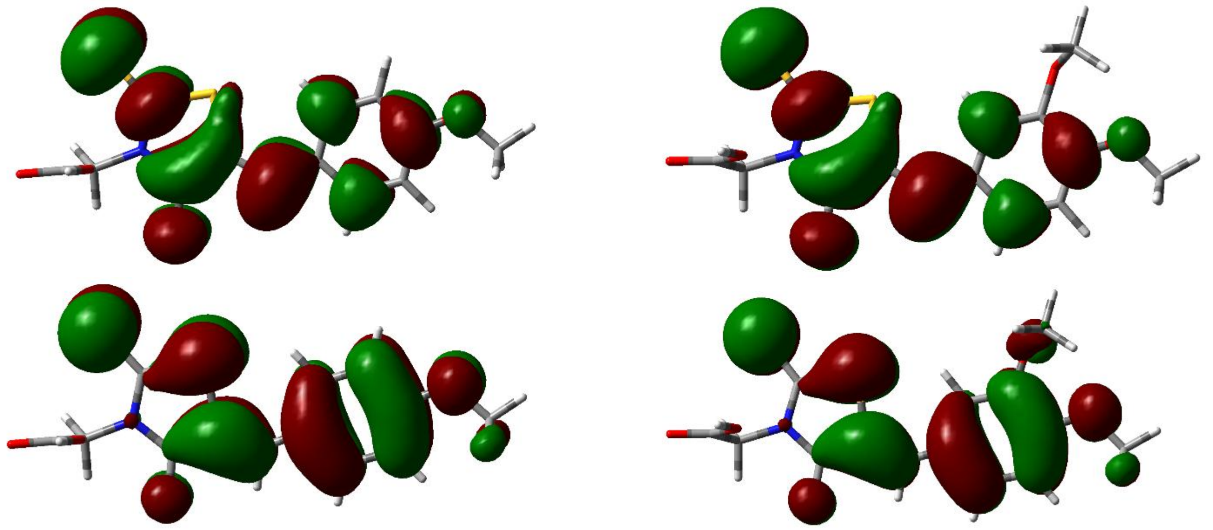

LUMO	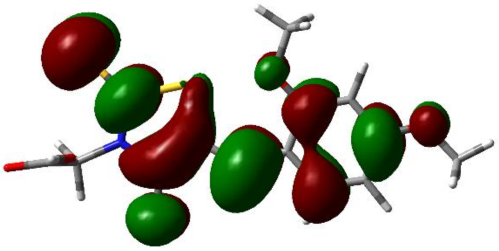	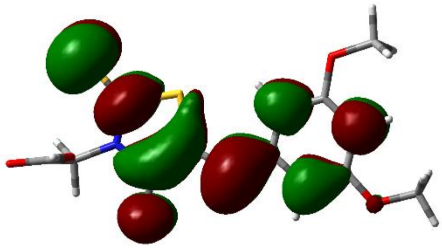
HOMO	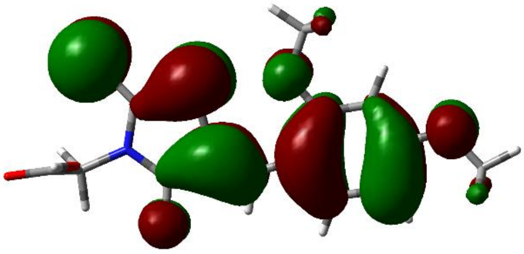	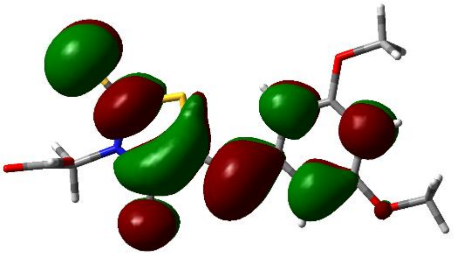
	**C5**	**C6**
LUMO	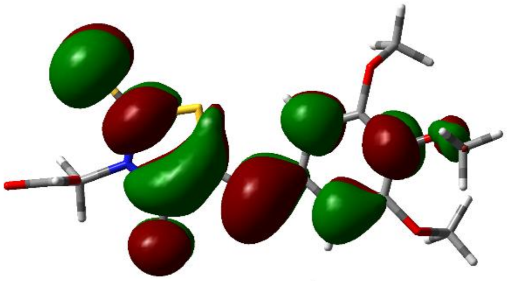	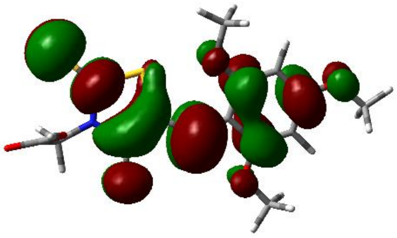
HOMO	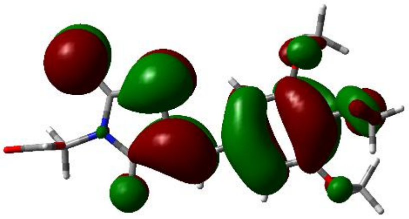	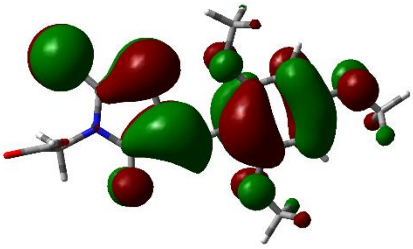
	**C7**	**C8**
LUMO	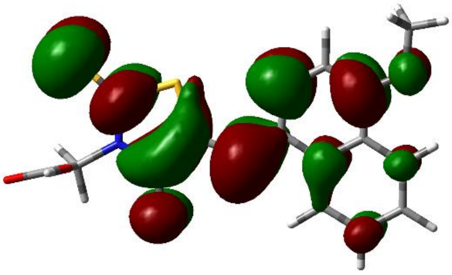	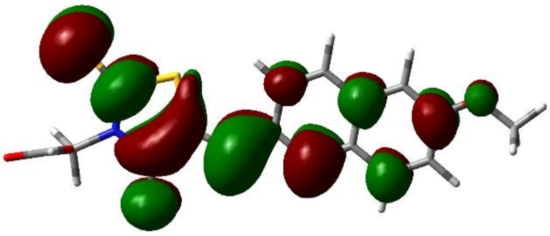
HOMO	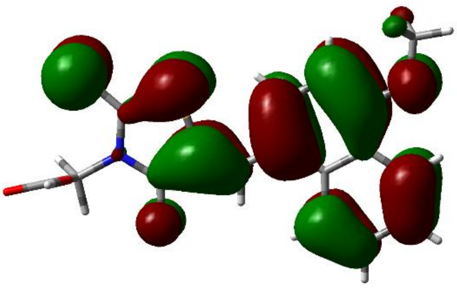	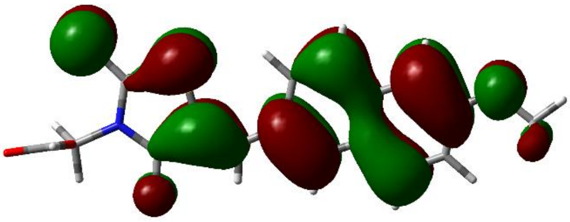

Figure S11. Density difference plots

C1

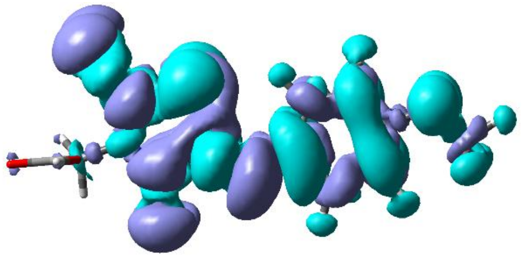

C2

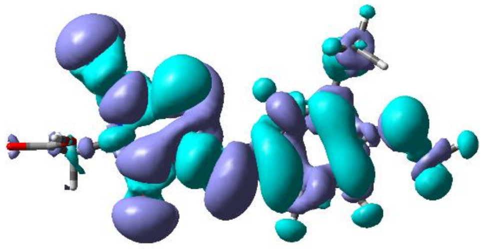

C3

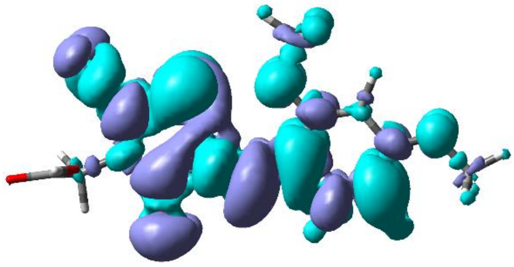

C4

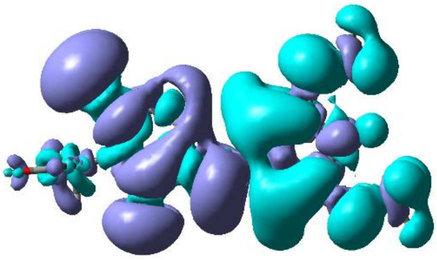

C5

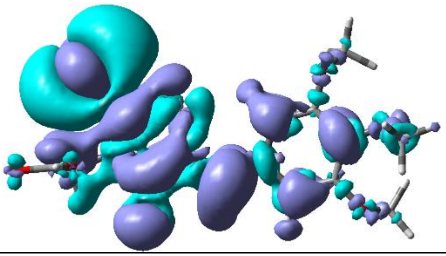

C6

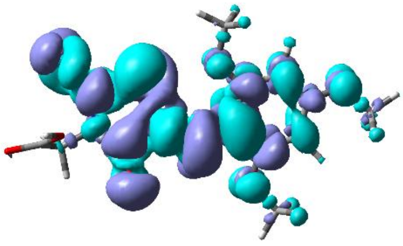

C7

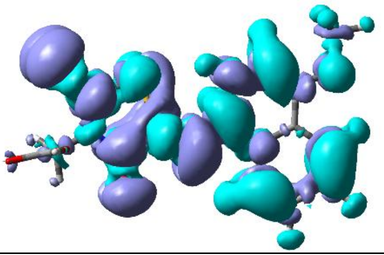

C8

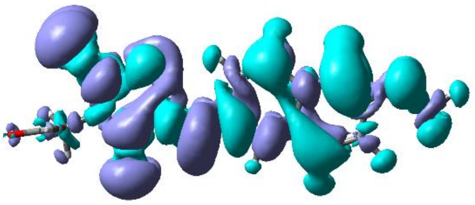

Figure S12. The MEP surfaces

C1
±0,0670

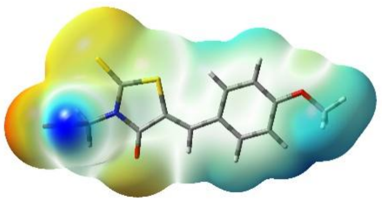

C2
±0,06753

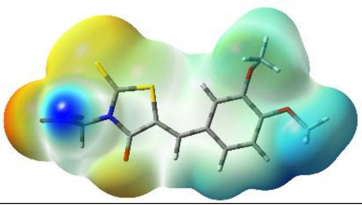

C3
±0,06932

C4
±0,07000

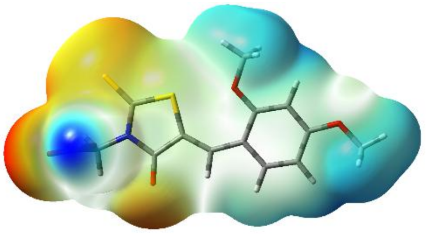

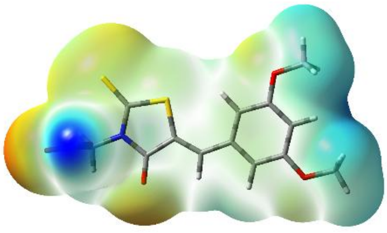

C5
±0,06709

C6
±0,06592

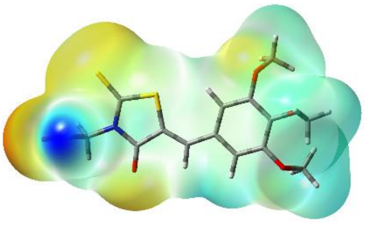

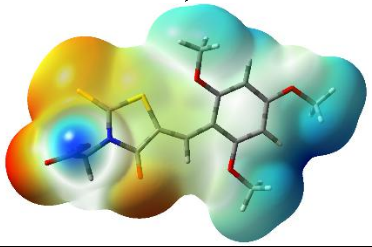

C7
±0,06700

C8
±0,06950

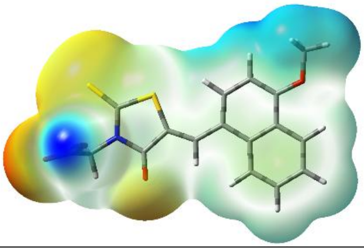

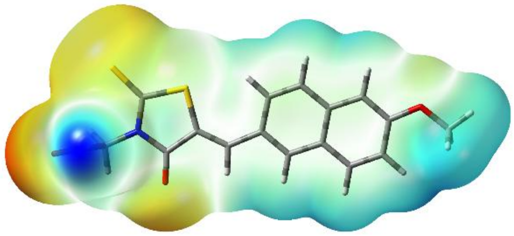

Figure S13. Predicted binding conformations of compounds C1–C8 in Concanavalin A (ConA) from AutoDock simulations.

C1

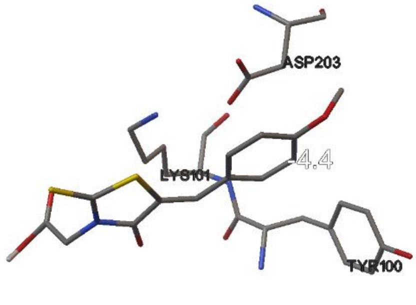

C2

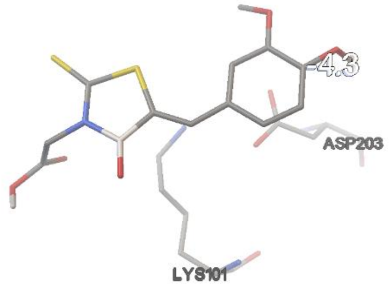

C3

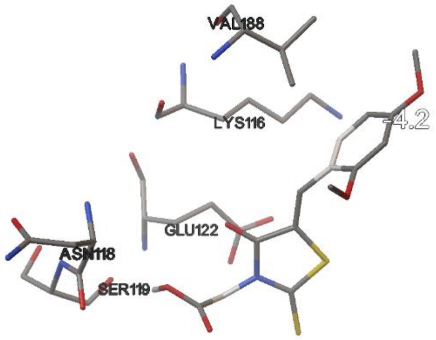

C4

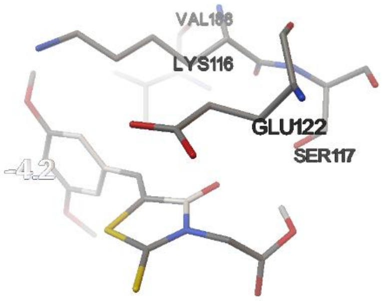

C5

C6

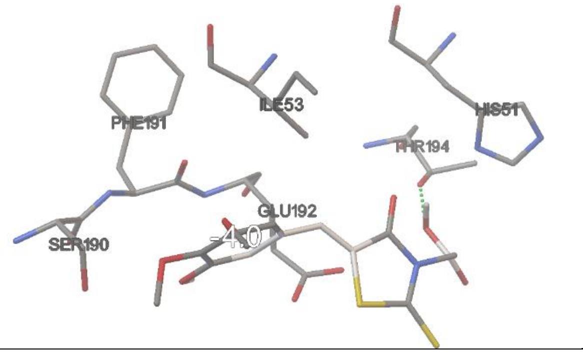

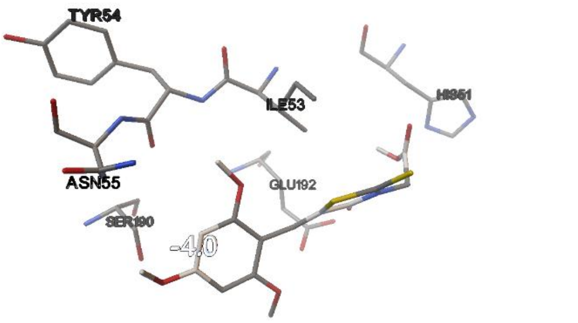

C7 | C8

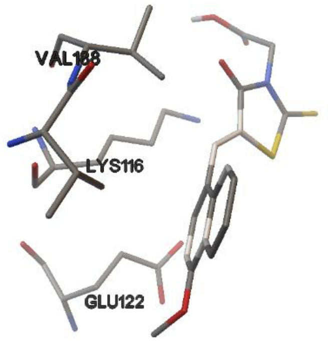

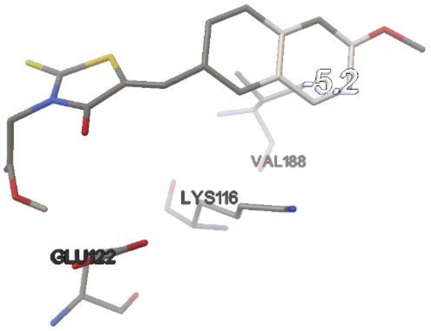

Figure S14. Predicted binding conformations of compounds C1–C8 in Human Serum Albuminum (HSA) from AutoDock simulations.

C1 | C2

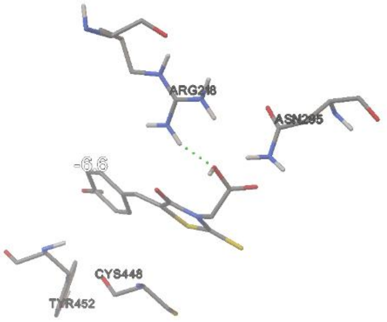

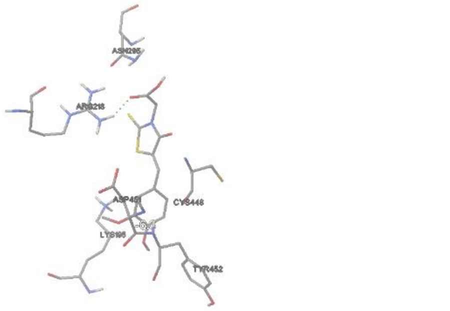

C3 | C4

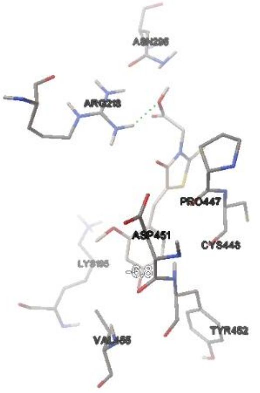

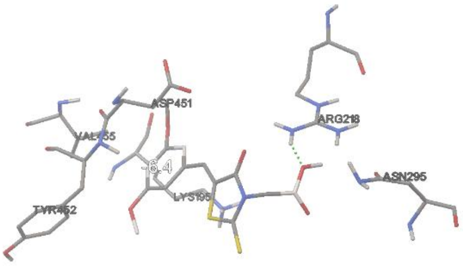

C5 | C6

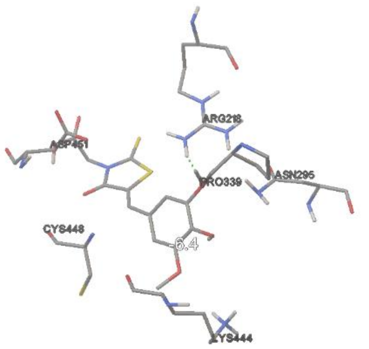

ARG218
ASP451
PRO339
ASN295
CYS448
-6.4
LYS444

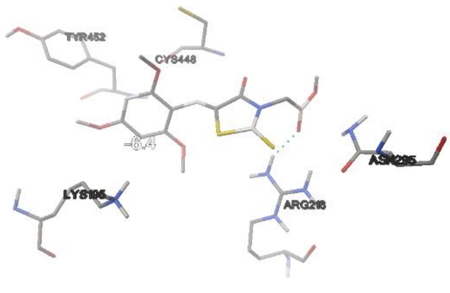

TYR452
CYS448
-6.4
ASN295
ARG218

C7

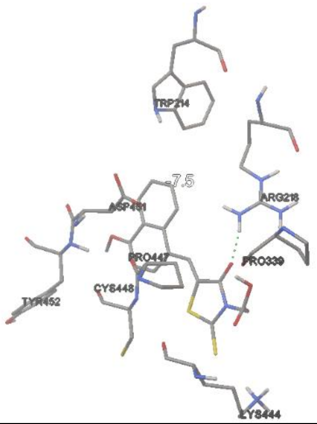

TRP214
-7.5
ARG218
ASP451
PRO447
PRO339
TYR452
CYS448
LYS444

C8

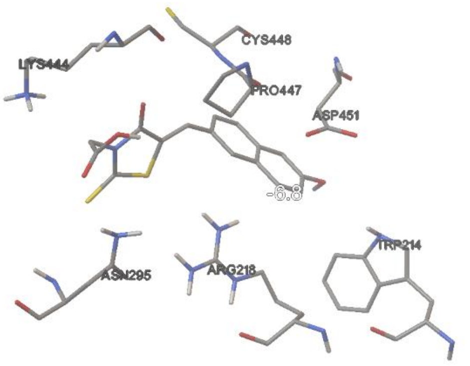

LYS444
CYS448
PRO447
ASP451
-6.8
ASN295
ARG218
TRP214

Table S1. The frontier orbital energies in tested solvents for **C-1-C-8** compounds. Values are given in [eV]

		E_{HOMO}	E_{LUMO}	E_{GAP}	η	μ	χ	σ	*pi*	*S*	ω	ΔNmax
C1	TOL	-6,3961	-2,7097	3,6864	1,8432	-4,5529	4,5529	0,5425	-4,5529	0,9216	5,6230	2,4701
	THF	-6,3988	-2,7540	3,6448	1,8224	-4,5764	4,5764	0,5487	-4,5764	0,9112	5,7462	2,5112
	CHCl3	-6,3972	-2,7399	3,6573	1,8286	-4,5685	4,5685	0,5469	-4,5685	0,9143	5,7068	2,4983
	MeOH	-6,4023	-2,7764	3,6260	1,8130	-4,5893	4,5893	0,5516	-4,5893	0,9065	5,8087	2,5314
	MeCN	-6,4023	-2,7769	3,6254	1,8127	-4,5896	4,5896	0,5517	-4,5896	0,9064	5,8102	2,5319
	DMSO	-6,4026	-2,7783	3,6243	1,8122	-4,5904	4,5904	0,5518	-4,5904	0,9061	5,8140	2,5331
C2	TOL	-6,3631	-2,7165	3,6467	1,8233	-4,5398	4,5398	0,5484	-4,5398	0,9117	5,6517	2,4899
	THF	-6,3738	-2,7690	3,6047	1,8024	-4,5714	4,5714	0,5548	-4,5714	0,9012	5,7972	2,5363
	CHCl3	-6,3697	-2,7527	3,6170	1,8085	-4,5612	4,5612	0,5529	-4,5612	0,9042	5,7518	2,5221
	MeOH	-6,3806	-2,7940	3,5865	1,7933	-4,5873	4,5873	0,5576	-4,5873	0,8966	5,8674	2,5581
	MeCN	-6,3808	-2,7946	3,5862	1,7931	-4,5877	4,5877	0,5577	-4,5877	0,8966	5,8688	2,5585
	DMSO	-6,3811	-2,7962	3,5849	1,7924	-4,5887	4,5887	0,5579	-4,5887	0,8962	5,8735	2,5600
C3	TOL	-6,1653	-2,5170	3,6483	1,8241	-4,3412	4,3412	0,5482	-4,3412	0,9121	5,1656	2,3798
	THF	-6,1958	-2,5910	3,6047	1,8024	-4,3934	4,3934	0,5548	-4,3934	0,9012	5,3546	2,4376
	CHCl3	-6,1857	-2,5676	3,6181	1,8090	-4,3767	4,3767	0,5528	-4,3767	0,9045	5,2943	2,4193
	MeOH	-6,2118	-2,6270	3,5849	1,7924	-4,4194	4,4194	0,5579	-4,4194	0,8962	5,4482	2,4656
	MeCN	-6,2124	-2,6278	3,5846	1,7923	-4,4201	4,4201	0,5579	-4,4201	0,8962	5,4503	2,4661
	DMSO	-6,2135	-2,6302	3,5832	1,7916	-4,4218	4,4218	0,5582	-4,4218	0,8958	5,4567	2,4681
C4	TOL	-6,5683	-2,8000	3,7683	1,8842	-4,6842	4,6842	0,5307	-4,6842	0,9421	5,8227	2,4861
	THF	-6,5651	-2,8615	3,7035	1,8518	-4,7133	4,7133	0,5400	-4,7133	0,9259	5,9984	2,5453
	CHCl3	-6,5645	-2,8422	3,7223	1,8612	-4,7034	4,7034	0,5373	-4,7034	0,9306	5,9430	2,5271
	MeOH	-6,5675	-2,8915	3,6760	1,8380	-4,7295	4,7295	0,5441	-4,7295	0,9190	6,0848	2,5731
	MeCN	-6,5675	-2,8920	3,6755	1,8378	-4,7298	4,7298	0,5441	-4,7298	0,9189	6,0864	2,5737
	DMSO	-6,5678	-2,8942	3,6736	1,8368	-4,7310	4,7310	0,5444	-4,7310	0,9184	6,0927	2,5757
C5	TOL	-6,5912	-2,8360	3,7552	1,8776	-4,7136	4,7136	0,5326	-4,7136	0,9388	5,9165	2,5104
	THF	-6,5983	-2,8751	3,7231	1,8616	-4,7367	4,7367	0,5372	-4,7367	0,9308	6,0262	2,5445
	CHCl3	-6,5961	-2,8632	3,7329	1,8665	-4,7296	4,7296	0,5358	-4,7296	0,9332	5,9925	2,5340

	MeOH	-6,6004	-2,8923	3,7082	1,8541	-4,7464	4,7464	0,5394	-4,7464	0,9270	6,0753	2,5600
	MeCN	-6,6004	-2,8928	3,7076	1,8538	-4,7466	4,7466	0,5394	-4,7466	0,9269	6,0768	2,5605
	DMSO	-6,6007	-2,8939	3,7068	1,8534	-4,7473	4,7473	0,5395	-4,7473	0,9267	6,0799	2,5614
C6	TOL	-6,0464	-2,4024	3,6439	1,8220	-4,2244	4,2244	0,5489	-4,2244	0,9110	4,8973	2,3186
	THF	-6,0940	-2,5113	3,5827	1,7914	-4,3026	4,3026	0,5582	-4,3026	0,8957	5,1673	2,4019
	CHCl3	-6,0790	-2,4773	3,6018	1,8009	-4,2782	4,2782	0,5553	-4,2782	0,9004	5,0816	2,3756
	MeOH	-6,1201	-2,5649	3,5552	1,7776	-4,3425	4,3425	0,5626	-4,3425	0,8888	5,3042	2,4429
	MeCN	-6,1212	-2,5663	3,5549	1,7775	-4,3437	4,3437	0,5626	-4,3437	0,8887	5,3076	2,4438
	DMSO	-6,1231	-2,5701	3,5530	1,7765	-4,3466	4,3466	0,5629	-4,3466	0,8883	5,3174	2,4467
C7	TOL	-6,1601	-2,7535	3,4066	1,7033	-4,4568	4,4568	0,5871	-4,4568	0,8517	5,8307	2,6166
	THF	-6,1579	-2,8033	3,3547	1,6773	-4,4806	4,4806	0,5962	-4,4806	0,8387	5,9845	2,6713
	CHCl3	-6,1577	-2,7875	3,3702	1,6851	-4,4726	4,4726	0,5934	-4,4726	0,8425	5,9357	2,6542
	MeOH	-6,1601	-2,8270	3,3332	1,6666	-4,4936	4,4936	0,6000	-4,4936	0,8333	6,0579	2,6963
	MeCN	-6,1601	-2,8275	3,3326	1,6663	-4,4938	4,4938	0,6001	-4,4938	0,8332	6,0597	2,6969
	DMSO	-6,1465	-2,8580	3,2885	1,6443	-4,5023	4,5023	0,6082	-4,5023	0,8221	6,1640	2,7382
C8	TOL	-6,5683	-2,8000	3,7683	1,8842	-4,6842	4,6842	0,5307	-4,6842	0,9421	5,8227	2,4861
	THF	-6,5651	-2,8615	3,7035	1,8518	-4,7133	4,7133	0,5400	-4,7133	0,9259	5,9984	2,5453
	CHCl3	-6,5645	-2,8422	3,7223	1,8612	-4,7034	4,7034	0,5373	-4,7034	0,9306	5,9430	2,5271
	MeOH	-6,5675	-2,8915	3,6760	1,8380	-4,7295	4,7295	0,5441	-4,7295	0,9190	6,0848	2,5731
	MeCN	-6,5675	-2,8920	3,6755	1,8378	-4,7298	4,7298	0,5441	-4,7298	0,9189	6,0864	2,5737
	DMSO	-6,5678	-2,8942	3,6736	1,8368	-4,7310	4,7310	0,5444	-4,7310	0,9184	6,0927	2,5757

Table S2. Occupancy (e), energy (a.u.) and polarity (%) of natural bond orbitals (NBOs) and hybrids calculated for investigated complexes

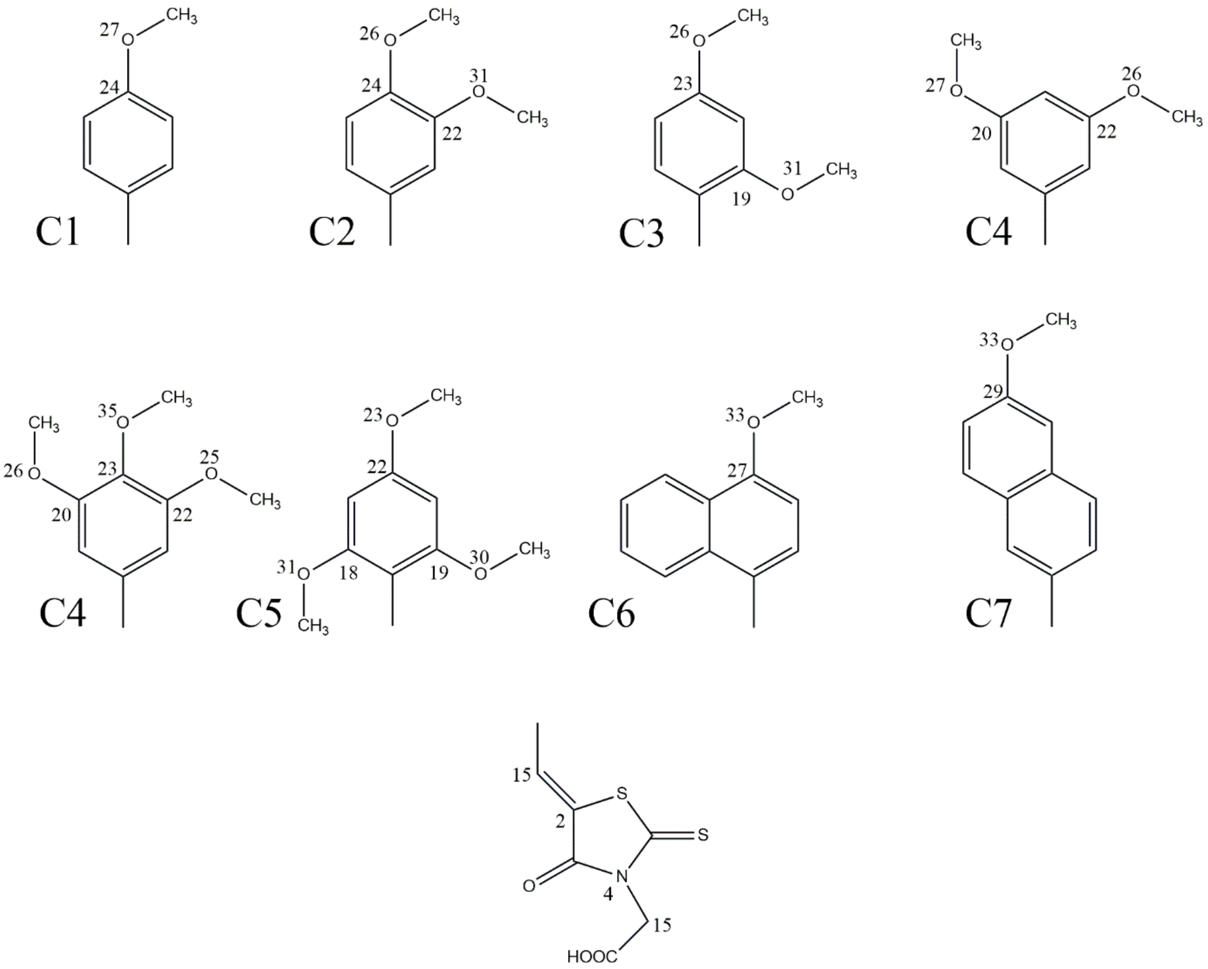

	NBO orbital	Orbital energy	Ocupancy		Charge	Polarity	NBO hybrid	Atomic orbitals
**C1**								
TOL		-0.81440	1.98100	C8	-0.30069	35.89%	$sp^{3.30}$	s(23.21%)p(76.65%)
				N4	-0.50717	64.11%	$sp^{2.06}$	s(32.63%)p(67.33%)
		-0.79899	1.97599	C2	-0.30882	50.80%	$sp^{1.37}$	s(42.18%)p(7.80%)
				C15	-0.10493	49.20%	$sp^{1.69}$	s(7.25%)p(62.73%)
		-0.96136	1.99233	C24	0.35410	32.53%	$sp^{3.00}$	s(24.93%)p(75.00%)
				O27	-0.52190	67.47%	$sp^{1.89}$	s(34.55%)p(65.40%)
$CHCl_3$		-0.81542	1.98097	C8	-0.30076	35.87%	$sp^{3.31}$	s(23.18%)p(76.63%)
				N4	-0.50508	64.13%	$sp^{2.06}$	s(32.63%)p(67.32%)
		-0.79894	1.97603	C2	-0.31142	50.83%	$sp^{1.37}$	s(42.17%)p(57.79%)
				C15	-0.09990	49.17%	$sp^{1.69}$	s(37.16%)p(62.82%)
		-0.96114	1.99242	C24	0.35620	32.54%	$sp^{3.00}$	s(24.97%)p(74.78%)

				O27	-0.52323	67.46%	sp$^{1.88}$	s(34.68%)p(65.23%)
THF		-0.81590	1.98095	C8	-0.30070	35.86%	sp$^{3.31}$	s(23.17%)p(76.64%)
				N4	-0.50409	64.14%	sp$^{2.06}$	s(32.63%)p(67.35%)
		-0.79894	1.97605	C2	-0.31248	50.84%	sp$^{1.37}$	s(42.16%)p(57.79%)
				C15	-0.09768	49.16%	sp$^{1.69}$	s(37.14%)p(62.84%)
		-0.96112	1.99245	C24	0.35705	32.55%	sp$^{2.99}$	s(24.95%)p(75.01%)
				O27	-0.52390	67.45%	sp$^{1.88}$	s(34.72%)p(65.24%)
MeOH		-0.81656	1.98088	C8	-0.30046	35.84%	sp$^{3.31}$	s(23.15%)p(76.83%)
				N4	-0.50246	64.16%	sp$^{2.06}$	s(32.63%)p(67.34%)
		-0.79893	1.97608	C2	-0.31397	50.86%	sp$^{1.37}$	s(42.15%)p(57.81%)
				C15	-0.09428	49.14%	sp$^{1.69}$	s(37.10%)p(62.82%)
		-0.96112	1.9925	C24	0.35824	32.55%	sp$^{2.99}$	s(25.00%)p(74.95%)
				O27	-0.52500	67.45%	sp$^{1.87}$	s(34.78%)p(65.19%)
MeCN		-0.81658	1.98088	C8	-0.30047	35.84%	sp$^{3.31}$	s(23.15%)p(76.81%)
				N4	-0.50243	64.16%	sp$^{2.06}$	s(32.63%)p(67.32%)
		-0.79893	1.97608	C2	-0.31400	50.86%	sp$^{1.37}$	s(42.15%)p(57.80%)
				C15	-0.09421	49.14%	sp$^{1.69}$	s(37.10%)p(62.82%)
		-0.96110	1.99251	C24	0.35825	32.55%	sp$^{2.99}$	s(25.00%)p(74.93%)
				O27	-0.52504	67.45%	sp$^{1.87}$	s(34.78%)p(65.18%)
DMSO		-0.81661	1.98087	C8	-0.30044	35.84%	sp$^{3.31}$	s(23.15%)p(76.71%)
				N4	-0.50231	64.16%	sp$^{2.06}$	s(32.63%)p(67.32%)
		-0.79894	1.97608	C2	-0.31410	50.86%	sp$^{1.37}$	s(42.15%)p(57.84%)
				C15	-0.09396	49.14%	sp$^{1.69}$	s(37.10%)p(62.78%)
		-0.96119	1.99252	C24	0.35835	32.55%	sp$^{2.99}$	s(25.00%)p(74.89%)
				O27	-0.52511	67.45%	sp$^{1.87}$	s(34.78%)p(65.17%)
	NBO orbital	Orbital energy	Ocup ancy		Charge	Polarity	NBO hybrid	Atomic orbitals
**C2**								
TOL		-0.85152	1.98099	C8	-0.30070	35.89%	sp$^{3.30}$	s(23.21%)p(76.67%)
				N4	-0.50735	64.11%	sp$^{2.06}$	s(32.63%)p(67.35%)
		-0.79920	1.97572	C2	-0.30790	50.83%	sp$^{1.37}$	s(42.18%)p(57.80%)
				C15	-0.10566	49.17%	sp$^{1.68}$	s(37.28%)p(62.69%)
		-0.91969	1.98991	C22	0.27500	33.55%	sp$^{2.95}$	s(25.29%)p(74.68%)
				O31	-0.56595	66.45%	sp$^{2.10}$	s(32.21%)p(67.68%)
		-0.96717	1.99148	C24	0.32043	32.71%	sp$^{3.01}$	s(24.91%)p(75.01%)
				O26	-0.52037	67.29%	sp$^{1.91}$	s(34.40%)p(65.52%)
$CHCl_3$		-0.85889	1.98096	C8	-0.30081	35.87%	sp$^{3.31}$	s(23.18%)p(76.60%)

				N4	-0.50524	64.13%	sp$^{2.06}$	s(32.64%)p(67.26%)
		-0.79944	1.97576	C2	-0.31039	50.85%	sp$^{1.37}$	s(42.16%)p(57.74%)
				C15	-0.10083	49.15%	sp$^{1.69}$	s(37.23%)p(62.72%)
		-0.92265	1.98994	C22	0.27299	33.53%	sp$^{2.95}$	s(25.26%)p(74.50%)
				O31	-0.57051	66.47%	sp$^{2.10}$	s(32.21%)p(67.72%)
		-0.96764	1.99156	C24	0.32370	32.74%	sp$^{2.99}$	s(24.98%)p(74.80%)
				O26	-0.52043	67.26%	sp$^{1.90}$	s(34.48%)p(65.45%)
THF		-0.86278	1.98094	C8	-0.30079	35.85%	sp$^{3.31}$	s(23.17%)p(76.79%)
				N4	-0.50425	64.15%	sp$^{2.06}$	s(32.64%)p(67.30%)
		-0.79954	1.97578	C2	-0.31138	50.86%	sp$^{1.37}$	s(42.16%)p(57.79%)
				C15	-0.09869	49.14%	sp$^{1.69}$	s(37.21%)p(62.69%)
		-0.92269	1.98995	C22	0.27200	33.52%	sp$^{2.95}$	s(25.24%)p(74.66%)
				O31	-0.57267	66.48%	sp$^{2.10}$	s(32.20%)p(67.74%)
		-0.96789	1.99160	C24	0.32509	32.75%	sp$^{2.99}$	s(25.00%)p(74.95%)
				O26	-0.52060	67.25%	sp$^{1.89}$	s(34.52%)p(65.41%)
MeOH		-0.86906	1.98088	C8	-0.30063	35.84%	sp$^{3.31}$	s(23.15%)p(76.73%)
				N4	-0.50264	64.16%	sp$^{2.06}$	s(32.64%)p(67.34%)
		-0.79966	1.97581	C2	-0.31276	50.88%	sp$^{1.37}$	s(42.15%)p(57.75%)
				C15	-0.09534	49.12%	sp$^{1.69}$	s(37.18%)p(62.72%)
		-0.92280	1.98997	C22	0.27036	33.50%	sp$^{2.96}$	s(25.21%)p(74.70%)
				O31	-0.57619	66.50%	sp$^{2.10}$	s(32.19%)p(67.71%)
		-0.96834	1.99165	C24	0.32712	32.76%	sp$^{2.98}$	s(25.04%)p(74.76%)
				O26	-0.52107	67.24%	sp$^{1.89}$	s(34.58%)p(65.30%)
MeCN		-0.86908	1.98088	C8	-0.30064	35.84%	sp$^{3.31}$	s(23.15%)p(76.70%)
				N4	-0.50260	64.16%	sp$^{2.06}$	s(32.64%)p(67.26%)
		-0.79967	1.82035	C2	-0.31279	50.88%	sp$^{1.37}$	s(42.15%)p(57.74%)
				C15	-0.09527	49.12%	sp$^{1.69}$	s(37.17%)p(62.72%)
		-0.92282	1.98997	C22	0.27032	33.50%	sp$^{2.96}$	s(25.21%)p(74.68%)
				O31	-0.57627	66.50%	sp$^{2.10}$	s(32.19%)p(67.72%)
		-0.96832	1.99165	C24	0.32715	32.76%	sp$^{2.98}$	s(25.04%)p(74.93%)
				O26	-0.52110	67.24%	sp$^{1.89}$	s(34.58%)p(65.40%)
DMSO		-0.86944	1.98087	C8	-0.30062	35.84%	sp$^{3.31}$	s(23.15%)p(76.77%)
				N4	-0.50249	64.16%	sp$^{2.06}$	s(32.64%)p(67.30%)
		-0.79967	1.97582	C2	-0.31288	50.88%	sp$^{1.37}$	s(42.15%)p(57.80%)
				C15	-0.09503	49.12%	sp$^{1.69}$	s(37.17%)p(62.78%)
		-0.92282	1.98997	C22	0.27020	33.50%	sp$^{2.96}$	s(25.21%)p(74.72%)
				O31	-0.57653	66.50%	sp$^{2.10}$	s(32.19%)p(67.72%)

		1.99166	-0.96834	C24	0.32729	32.76%	$sp^{2.98}$	s(25.04%)p(74.88%)
				O26	-0.52115	67.24%	$sp^{1.89}$	s(34.59%)p(65.35%)
	NBO orbital	Orbital energy	Ocup ancy		Charge	P olarity	NBO hybrid	Atomic orbitals
**C3**								
TOL		1.98177	-0.80980	C8	-0.30313	36.03%	$sp^{3.28}$	s(23.34%)p(76.66%)
				N4	-0.51467	63.97%	$sp^{2.07}$	s(32.61%)p(67.39%)
		1.97619	-0.79816	C2	-0.31369	50.57%	$sp^{1.36}$	s(42.33%)p(57.67%)
				C15	-0.10933	49.43%	$sp^{1.63}$	s(38.04%)p(61.93%)
		1.99179	-0.94500	C19	0.38917	32.24%	$sp^{3.08}$	s(24.45%)p(75.33%)
				O31	-0.53810	67.76%	$sp^{1.86}$	s(34.97%)p(64.96%)
		1.99217	-0.96634	C23	0.37565	32.52%	$sp^{3.01}$	s(24.87%)p(74.90%)
				O26	-0.52350	32.52%	$sp^{1.89}$	s(34.61%)p(76.55%)
$CHCl_3$		1.98174	-0.81180	C8	-0.30315	36.00%	$sp^{3.28}$	s(23.31%)p(67.38%)
				N4	-0.51256	64.00%	$sp^{2.07}$	s(32.60%)p(67.38%)
		1.97659	-0.79734	C2	-0.31748	50.56%	$sp^{1.36}$	s(42.31%)p(57.65%)
				C15	-0.10454	49.44%	$sp^{1.63}$	s(37.97%)p(61.99%)
		1.99181	-0.94225	C19	0.39081	32.24%	$sp^{3.07}$	s(24.49%)p(75.30%)
				O31	-0.53923	67.76%	$sp^{1.85}$	s(35.02%)p(64.91%)
		1.99228	-0.96715	C23	0.37846	32.53%	$sp^{3.00}$	s(24.92%)p(74.86%)
				O26	-0.52407	67.47%	$sp^{1.88}$	s(34.70%)p(65.23%)
THF		1.98171	-0.81270	C8	-0.30310	35.99%	$sp^{3.29}$	s(23.30%)p(76.57%)
				N4	-0.51156	64.01%	$sp^{2.07}$	s(32.60%)p(67.38%)
		1.97659	-0.79715	C2	-0.31928	50.57%	$sp^{1.36}$	s(42.30%)p(57.67%)
				C15	-0.10209	49.43%	$sp^{1.63}$	s(7.94%)p(62.02%)
		1.99183	-0.94306	C19	0.39141	32.25%	$sp^{3.07}$	s(24.50%)p(75.28%)
				O31	-0.53974	67.75%	$sp^{1.85}$	s(35.04%)p(64.89%)
		1.99232	-0.96755	C23	0.37978	32.53%	$sp^{3.00}$	s(24.94%)p(74.84%)
				O26	-0.52431	67.47%	$sp^{1.88}$	s(34.75%)p(65.18%)
MeOH		1.98165	-0.81408	C8	-0.30294	35.98%	$sp^{3.29}$	s(23.28%)p(76.59%)
				N4	-0.50996	64.02%	$sp^{2.07}$	s(32.60%)p(67.38%)
		1.97659	-0.79684	C2	-0.32198	50.59%	$sp^{1.36}$	s(42.27%)p(57.69%)
				C15	-0.09838	49.41%	$sp^{1.64}$	s(37.89%)p(62.07%)
		1.99186	-0.94428	C19	0.39226	32.25%	$sp^{3.07}$	s(24.53%)p(75.26%)
				O31	-0.54054	67.75%	$sp^{1.85}$	s(35.07%)p(64.86%)
		1.99238	-0.96826	C23	0.38180	32.54%	$sp^{3.00}$	s(24.97%)p(74.81%)
				O26	-0.52463	67.46%	$sp^{1.87}$	s(34.81%)p(65.11%)

MeCN		1.98165	-0.81412	C8	-0.30295	35.98%	sp$^{3.29}$	s(23.27%)p(76.59%)
				N4	-0.50992	64.02%	sp$^{2.07}$	s(32.60%)p(67.38%)
		1.97660	-0.79686	C2	-0.32203	50.59%	sp$^{1.36}$	s(42.27%)p(57.69%)
				C15	-0.09831	49.41%	sp$^{1.64}$	s(37.89%)p(62.07%)
		1.99186	-0.94426	C19	0.39227	32.25%	sp$^{3.07}$	s(24.53%)p(75.26%)
				O31	-0.54057	67.75%	sp$^{1.85}$	s(35.07%)p(64.86%)
		1.99238	-0.96824	C23	0.38183	32.54%	sp$^{3.00}$	s(24.97%)p(74.81%)
				O26	-0.52465	67.46%	sp$^{1.87}$	s(34.81%)p(65.12%)
DMSO		1.98165	-0.81421	C8	-0.30293	35.98%	sp$^{3.29}$	s(23.27%)p(76.59%)
				N4	-0.50980	64.02%	sp$^{2.07}$	s(32.60%)p(67.38%)
		1.97660	-0.79684	C2	-0.32222	50.59%	sp$^{1.36}$	s(42.27%)p(57.70%)
				C15	-0.09805	49.41%	sp$^{1.64}$	s(37.89%)p(62.08%)
		1.99186	-0.94434	C19	0.39232	32.25%	sp$^{3.07}$	s(24.53%)p(75.26%)
				O31	-0.54062	67.75%	sp$^{1.85}$	s(35.07%)p(64.86%)
		1.99239	-0.96830	C23	0.38197	32.54%	sp$^{3.00}$	s(24.97%)p(74.81%)
				O26	-0.52467	67.46%	sp$^{1.87}$	s(34.82%)p(65.11%)
	NBO orbital	Orbital energy	Ocup ancy		Charge	P olarity	NBO hybrid	Atomic orbitals
				**C4**				
TOL		1.98092	-0.83977	C8	-0.30063	35.84%	sp$^{3.31}$	s(23.18%)p(76.69%)
				N4	-0.50752	64.16%	sp$^{2.06}$	s(32.67%)p(67.31%)
		1.97599	-0.80374	C2	-0.29437	50.92%	sp$^{1.36}$	s(42.39%)p(57.58%)
				C15	-0.10635	49.08%	sp$^{1.68}$	s(37.31%)p(62.66%)
		1.99175	-0.95044	C20	0.34150	32.67%	sp$^{3.03}$	s(24.74%)p(75.04%)
				O27	-0.53119	67.33%	sp$^{1.94}$	s(34.02%)p(65.91%)
		1.99177	-0.96986	C22	0.34468	32.67%	sp$^{3.04}$	s(24.69%)p(75.10%)
				O26	-0.53079	67.33%	sp$^{1.94}$	s(34.04%)p(65.89%)
$CHCl_3$		1.98089	-0.84022	C8	-0.30075	35.81%	sp$^{3.31}$	s(23.14%)p(76.72%)
				N4	-0.50540	64.19%	sp$^{2.06}$	s(32.68%)p(67.30%)
		1.97604	-0.80464	C2	-0.29556	50.93%	sp$^{1.36}$	s(42.38%)p(57.58%)
				C15	-0.10250	49.07%	sp$^{1.68}$	s(37.28%)p(62.68%)
		1.99183	-0.95022	C20	0.34121	32.69%	sp$^{3.03}$	s(24.78%)p(75.00%)
				O27	-0.53373	67.31%	sp$^{1.93}$	s(34.06%)p(65.87%)
		1.99184	-0.96978	C22	0.34440	32.69%	sp$^{3.04}$	s(24.73%)p(75.06%)
				O26	-0.53353	67.31%	sp$^{1.93}$	s(34.08%)p(65.85%)
THF		1.98086	-0.84040	C8	-0.30075	35.79%	sp$^{3.32}$	s(23.13%)p(76.73%)
				N4	-0.50440	64.21%	sp$^{2.06}$	s(32.68%)p(67.30%)

		1.97606	-0.80507	C2	-0.29598	50.94%	$sp^{1.36}$	s(42.38%)p(57.58%)
				C15	-0.10087	49.06%	$sp^{1.68}$	s(37.27%)p(62.69%)
		1.99186	-0.95020	C20	0.34103	32.70%	$sp^{3.02}$	s(24.79%)p(74.99%)
				O27	-0.53492	67.30%	$sp^{1.93}$	s(34.08%)p(65.85%)
		1.99187	-0.96979	C22	0.34422	49.93%	$sp^{1.65}$	s(37.76%)p(62.21%)
				O26	-0.53482	50.07%	$sp^{1.86}$	s(34.97%)p(64.98%)
MeOH		1.98080	-0.84056	C8	-0.30063	35.77%	$sp^{3.32}$	s(23.11%)p(76.76%)
				N4	-0.50279	64.23%	$sp^{2.06}$	s(32.68%)p(67.30%)
		1.97610	-0.80573	C2	-0.29647	50.95%	$sp^{1.36}$	s(42.38%)p(57.58%
				C15	-0.09840	49.05%	$sp^{1.68}$	s(37.26%)p(62.70%)
		1.99191	-0.95022	C20	0.34072	32.72%	$sp^{3.02}$	s(24.82%)p(74.96%)
				O27	-0.53693	67.28%	$sp^{1.93}$	s(34.11%)p(65.82%)
		1.99191	-0.96985	C22	0.34388	32.72%	$sp^{3.03}$	s(24.77%)p(75.03%)
				O26	-0.53683	67.28%	$sp^{1.93}$	s(34.12%)p(65.81%)
MeCN		1.98080	-0.84058	C8	-0.30064	35.77%	$sp^{3.32}$	s(23.11%)p(76.76%)
				N4	-0.50275	64.23%	$sp^{2.06}$	s(32.68%)p(67.30%)
		1.97610	-0.80574	C2	-0.29647	50.95%	$sp^{1.36}$	s(42.38%)p(57.58%)
				C15	-0.09835	49.05%	$sp^{1.68}$	s(37.26%)p(62.71%)
		1.99191	-0.95021	C20	0.34070	32.72%	$sp^{3.02}$	s(24.82%)p(74.96%)
				O27	-0.53687	67.28%	$sp^{1.93}$	s(34.11%)p(65.82%)
		1.99191	-0.96985	C22	0.34386	32.72%	$sp^{3.03}$	s(24.77%)p(75.03%)
				O26	-0.53699	67.28%	$sp^{1.93}$	s(34.12%)p(65.81%)
DMSO		1.98080	-0.84058	C8	-0.30062	35.77%	$sp^{3.32}$	s(23.11%)p(76.76%)
				N4	-0.50263	64.23%	$sp^{2.06}$	s(32.69%)p(67.30%)
		1.97610	-0.80579	C2	-0.29650	50.95%	$sp^{1.36}$	s(42.38%)p(57.58%)
				C15	-0.09818	49.05%	$sp^{1.68}$	s(37.26%)p(62.71%)
		1.99191	-0.95022	C20	0.34068	32.72%	$sp^{3.02}$	s(24.82%)p(74.96%)
				O27	-0.53701	67.28%	$sp^{1.93}$	s(34.11%)p(65.82%)
		1.99192	-0.96985	C22	0.34383	32.72%	$sp^{3.03}$	s(24.77%)p(75.03%)
				O26	-0.53714	67.28%	$sp^{1.93}$	s(34.12%)p(65.81%)
	NBO orbital	Orbital energy	Ocup ancy		Charge	P olarity	NBO hybrid	Atomic orbitals
**C5**								
TOL		1.98090	-0.94250	C8	-0.30061	35.84%	$sp^{3.31}$	s(23.18%)p(76.69%)
				N4	-0.50726	64.16%	$sp^{2.06}$	s(32.66%)p(67.32%)
		1.97629	-0.80448	C2	-0.29773	50.82%	$sp^{1.36}$	s(42.28%)p(57.69%)
				C15	-0.11078	49.18%	$sp^{1.68}$	s(37.34%)p(62.63%)

		1.98962	-0.92575	C20	0.27455	33.40%	sp$^{3.00}$	s(24.95%)p(74.83%)
				O26	-0.57333	66.60%	sp$^{2.10}$	s(32.21%)p(67.72%)
		1.98984	-0.92291	C22	0.28848	33.51%	sp$^{2.97}$	s(25.16%)p(74.62%)
				O25	-0.56629	66.49%	sp$^{2.10}$	s(32.28%)p(67.65%)
		1.98925	-0.93586	C23	0.25783	33.42%	sp$^{2.99}$	s(25.00%)p(74.78%)
				O35	-0.57329	66.58%	sp$^{2.08}$	s(32.49%)p(67.44%)
$CHCl_3$		1.98088	-0.95500	C8	-0.30072	35.81%	sp$^{3.31}$	s(23.15%)p(76.71%)
				N4	-0.50519	64.19%	sp$^{2.06}$	s(32.67%)p(67.31%)
		1.97633	-0.80470	C2	-0.29925	50.84%	sp$^{1.36}$	s(42.27%)p(57.69%)
				C15	-0.10632	49.16%	sp$^{1.68}$	s(37.31%)p(62.66%)
		1.98969	-0.92564	C20	0.27358	33.41%	sp$^{3.00}$	s(24.97%)p(74.81%)
				O26	-0.57568	66.59%	sp$^{2.10}$	s(32.25%)p(67.68%)
		1.98987	-0.92229	C22	0.28756	33.51%	sp$^{2.97}$	s(25.16%)p(74.63%)
				O25	-0.56953	66.49%	sp$^{2.10}$	s(32.29%)p(67.64%)
		1.98939	-0.93702	C23	0.26135	33.43%	sp$^{2.98}$	s(25.05%)p(74.73%)
				O35	-0.57251	66.57%	sp$^{2.07}$	s(32.60%)p(67.33%)
THF		1.98086	1.98086	C8	-0.30071	35.80%	sp$^{3.32}$	s(23.14%)p(76.73%)
				N4	-0.50421	64.20%	sp$^{2.06}$	s(32.67%)p(67.31%)
		1.97634	-0.80477	C2	-0.29984	50.84%	sp$^{1.37}$	s(42.27%)p(57.70%)
				C15	-0.10430	49.16%	sp$^{1.68}$	s(37.30%)p(62.67%)
		1.98972	-0.92568	C20	0.27307	33.41%	sp$^{2.99}$	s(24.98%)p(74.80%)
				O26	-0.57673	66.59%	sp$^{2.10}$	s(32.26%)p(67.67%)
		1.98988	-0.92206	C22	0.28706	33.50%	sp$^{2.97}$	s(25.15%)p(74.63%)
				O25	-0.57110	66.50%	sp$^{2.10}$	s(32.29%)p(67.64%)
		1.98945	-0.93768	C23	0.26290	33.43%	sp$^{2.98}$	s(25.07%)p(74.71%)
				O35	-0.57206	66.57%	sp$^{2.06}$	s(32.66%)p(67.28%)
MeOH		1.98080	-0.96870	C8	-0.30055	35.79%	sp$^{3.32}$	s(23.12%)p(76.75%)
				N4	-0.50259	64.21%	sp$^{2.06}$	s(32.67%)p(67.31%)
		1.97637	-0.80482	C2	-0.30066	50.86%	sp$^{1.37}$	s(42.26%)p(57.70%)
				C15	-0.10110	49.14%	sp$^{1.68}$	s(37.28%)p(62.69%)
		1.98978	-0.92589	C20	0.27203	33.41%	sp$^{2.99}$	s(24.99%)p(74.79%)
				O26	-0.57835	66.59%	sp$^{2.10}$	s(32.28%)p(67.64%)
		1.98988	-0.92171	C22	0.28612	33.49%	sp$^{2.97}$	s(25.14%)p(74.64%)
				O25	-0.57374	66.51%	sp$^{2.10}$	s(32.28%)p(67.65%)
		1.98956	-0.93902	C23	0.26539	33.43%	sp$^{2.97}$	s(25.11%)p(74.67%)
				O35	-0.57102	66.57%	sp$^{2.05}$	s(32.75%)p(67.18%)
MeCN		1.98080	-0.96870	C8	-0.30056	35.79%	sp$^{3.32}$	s(23.12%)p(76.75%)

				N4	-0.50256	64.21%	sp$^{2.06}$	s(32.67%)p(67.31%)
		1.97637	-0.80483	C2	-0.30067	50.86%	sp$^{1.37}$	s(42.26%)p(57.70%)
				C15	-0.10103	49.14%	sp$^{1.68}$	s(37.27%)p(62.69%)
		1.98978	-0.92588	C20	0.27203	33.41%	sp$^{2.99}$	s(24.99%)p(74.79%)
				O26	-0.57839	66.59%	sp$^{2.10}$	s(32.29%)p(67.64%)
		1.98988	-0.92172	C22	0.28610	33.49%	sp$^{2.97}$	s(25.14%)p(74.64%)
				O25	-0.57380	66.51%	sp$^{2.10}$	s(32.28%)p(67.65%)
		1.98956	-0.93901	C23	0.26542	33.43%	sp$^{2.97}$	s(25.11%)p(74.67%)
				O35	-0.57103	66.57%	sp$^{2.05}$	s(32.75%)p(67.18%)
DMSO		1.98080	-0.96904	C8	-0.30053	35.78%	sp$^{3.32}$	s(23.11%)p(76.75%)
				N4	-0.50244	64.22%	sp$^{2.06}$	s(32.67%)p(67.31%)
		1.97637	-0.80483	C2	-0.30072	50.86%	sp$^{1.37}$	s(42.26%)p(57.70%)
				C15	-0.10080	49.14%	sp$^{1.68}$	s(37.27%)p(62.69%)
		1.98978	-0.92591	C20	0.27196	33.41%	sp$^{2.99}$	s(25.00%)p(74.79%)
				O26	-0.57850	66.59%	sp$^{2.10}$	s(32.29%)p(67.64%)
		1.98988	-0.92169	C22	0.28602	33.49%	sp$^{2.97}$	s(25.14%)p(74.64%)
				O25	-0.57400	66.51%	sp$^{2.10}$	s(32.28%)p(67.65%)
		1.98957	-0.93911	C23	0.26560	33.43%	sp$^{2.97}$	s(25.11%)p(74.67%)
				O35	-0.57095	66.57%	sp$^{2.05}$	s(32.76%)p(67.17%)
	NBO orbital	Orbital energy	Ocup ancy		Charge	P olarity	NBO hybrid	Atomic orbitals
**C6**								
TOL		1.98144	-0.83326	C8	-0.30151	36.06%	sp$^{3.29}$	s(23.30%)p(76.56%)
				N4	-0.50478	63.94%	sp$^{2.07}$	s(32.57%)p(67.41%)
		1.97901	-0.78711	C2	-0.32030	50.89%	sp$^{1.37}$	s(42.24%)p(57.73%)
				C15	-0.11431	49.11%	sp$^{1.62}$	s(38.16%)p(61.80%)
		1.99135	-1.04070	C18	0.40911	32.40%	sp$^{3.08}$	s(24.48%)p(75.30%)
				O31	-0.52881	67.60%	sp$^{1.87}$	s(34.78%)p(65.15%)
		1.99129	-0.98078	C19	0.39798	32.26%	sp$^{3.10}$	s(24.35%)p(75.44%)
				O30	-0.53561	67.74%	sp$^{1.86}$	s(34.93%)p(65.00%)
		1.99206	-0.96717	C22	0.39028	32.51%	sp$^{3.03}$	s(24.77%)p(75.00%)
				O23	-0.52271	67.49%	sp$^{1.89}$	s(34.56%)p(65.37%)
$CHCl_3$		1.98163	-0.89987	C8	-0.30187	36.05%	sp$^{3.29}$	s(23.29%)p(76.57%)
				N4	-0.50776	63.95%	sp$^{2.07}$	s(32.58%)p(67.40%)
		1.97898	-0.78913	C2	-0.32637	50.90%	sp$^{1.37}$	s(42.20%)p(57.77%)
				C15	-0.10969	49.10%	sp$^{1.62}$	s(38.12%)p(61.85%)
		1.99144	-1.21416	C18	0.41194	32.42%	sp$^{3.06}$	s(24.55%)p(75.24%)

				O31	-0.53039	67.58%	sp$^{1.87}$	s(34.85%)p(65.08%)
		1.99132	-0.95877	C19	0.39967	32.27%	sp$^{3.09}$	s(24.38%)p(75.41%)
				O30	-0.53752	67.73%	sp$^{1.86}$	s(34.98%)p(64.95%)
		1.99217	-0.96614	C22	0.39417	32.53%	sp$^{3.02}$	s(24.83%)p(74.95%)
				O23	-0.52300	67.47%	sp$^{1.88}$	s(34.67%)p(65.26%)
THF		1.98221	-0.81992	C8	-0.30797	36.12%	sp$^{3.28}$	s(23.34%)p(76.53%)
				N4	-0.50313	63.88%	sp$^{2.07}$	s(32.52%)p(67.46%)
		1.97638	-0.78656	C2	-0.31697	50.58%	sp$^{1.38}$	s(41.97%)p(58.00%)
				C15	-0.11245	49.42%	sp$^{1.63}$	s(38.00%)p(61.97%)
		1.99149	-0.96649	C18	0.41332	32.42%	sp$^{3.06}$	s(24.58%)p(75.21%)
				O31	-0.53143	67.58%	sp$^{1.86}$	s(34.89%)p(65.04%)
		1.99135	-3.83857	C19	0.40040	32.26%	sp$^{3.09}$	s(24.39%)p(75.40%)
				O30	-0.53843	67.74%	sp$^{1.85}$	s(35.01%)p(64.92%)
		1.99222	-0.96750	C22	0.39609	32.54%	sp$^{3.01}$	s(24.86%)p(74.92%)
				O23	-0.52300	67.46%	sp$^{1.88}$	s(34.72%)p(65.21%)
MeOH		1.98212	-0.82233	C8	-0.30768	36.10%	sp$^{3.28}$	s(23.31%)p(76.56%)
				N4	-0.50130	63.90%	sp$^{2.08}$	s(32.51%)p(67.47%)
		1.97638	-0.78738	C2	-0.32046	50.58%	sp$^{1.38}$	s(41.92%)p(58.04%)
				C15	-0.10960	49.42%	sp$^{1.63}$	s(37.96%)p(62.00%)
		1.99155	-0.96735	C18	0.41517	32.44%	sp$^{3.05}$	s(24.62%)p(75.17%)
				O31	-0.53269	67.56%	sp$^{1.86}$	s(34.94%)p(64.99%)
		1.99138	-3.76280	C19	0.40136	32.26%	sp$^{3.09}$	s(24.41%)p(75.38%)
				O30	-0.53951	67.74%	sp$^{1.85}$	s(35.04%)p(64.89%)
		1.99229	-0.99117	C22	0.39899	32.55%	sp$^{3.01}$	s(24.90%)p(74.88%)
				O23	-0.52299	67.45%	sp$^{1.87}$	s(34.81%)p(65.12%)
MeCN		1.98212	-0.82239	C8	-0.30769	36.10%	sp$^{3.28}$	s(23.31%)p(76.56%)
				N4	-0.50127	63.90%	sp$^{2.08}$	s(32.51%)p(67.47%)
		1.97638	-0.78743	C2	-0.32053	50.58%	sp$^{1.38}$	s(41.92%)p(58.04%)
				C15	-0.10954	49.42%	sp$^{1.63}$	s(37.96%)p(62.01%)
		1.99155	-0.96734	C18	0.41519	32.44%	sp$^{3.05}$	s(24.62%)p(75.17%)
				O31	-0.53273	67.56%	sp$^{1.86}$	s(34.94%)p(64.99%)
		1.99138	-3.76266	C19	0.40136	32.26%	sp$^{3.09}$	s(24.41%)p(75.38%)
				O30	-0.53954	67.74%	sp$^{1.85}$	s(35.04%)p(64.89%)
		1.99229	-0.99117	C22	0.39903	32.55%	sp$^{3.01}$	s(24.90%)p(74.88%)
				O23	-0.52301	67.45%	sp$^{1.87}$	s(34.81%)p(65.12%)
DMSO		1.98211	-0.82260	C8	-0.30768	36.10%	sp$^{3.28}$	s(23.31%)p(76.56%)
				N4	-0.50114	63.90%	sp$^{2.08}$	s(32.51%)p(67.47%)

		1.97638	-0.78750	C2	-0.32076	50.58%	$sp^{1.38}$	s(41.92%)p(58.05%)
				C15	-0.10933	49.42%	$sp^{1.63}$	s(37.96%)p(62.01%)
		1.99155	-0.96739	C18	0.41531	32.44%	$sp^{3.05}$	s(24.62%)p(75.17%)
				O31	-0.53282	67.56%	$sp^{1.86}$	s(34.95%)p(64.98%)
		1.99138	-3.75925	C19	0.40142	32.26%	$sp^{3.09}$	s(24.41%)p(75.38%)
				O30	-0.53963	67.74%	$sp^{1.85}$	s(35.04%)p(64.89%)
		1.99229	-0.99132	C22	0.39922	32.55%	$sp^{3.01}$	s(24.90%)p(74.88%)
				O23	-0.52301	67.45%	$sp^{1.87}$	s(34.81%)p(65.12%)
	NBO orbital	Orbital energy	Ocup ancy		Charge	P olarity	NBO hybrid	Atomic orbitals
**C7**								
TOL		1.98150	-0.81876	C8	-0.30736	36.00%	$sp^{3.29}$	s(23.29%)p(76.58%)
				N4	-0.50067	64.00%	$sp^{2.07}$	s(32.60%)p(67.38%)
		1.82959	-0.31670	C2	-0.31273	51.09%	$sp^{1.36}$	s(42.39%)p(57.59%)
				C15	-0.10730	48.91%	$sp^{1.68}$	s(37.32%)p(62.64%)
		1.99164	-0.96838	C27	0.38705	32.39%	$sp^{3.05}$	s(24.65%)p(75.12%)
				O33	-0.52260	67.61%	$sp^{1.86}$	s(34.89%)p(65.04%)
$CHCl_3$		1.98146	-0.81905	C8	-0.30742	35.98%	$sp^{3.29}$	s(23.26%)p(76.61%)
				N4	-0.49862	64.02%	$sp^{2.07}$	s(32.60%)p(67.38%)
		1.97846	-0.80024	C2	-0.31544	51.13%	$sp^{1.36}$	s(42.38%)p(57.60%)
				C15	-0.10433	48.87%	$sp^{1.68}$	s(37.32%)p(62.64%)
		1.99172	-0.96882	C27	0.38926	32.42%	$sp^{3.04}$	s(24.72%)p(75.05%)
				O33	-0.52270	67.58%	$sp^{1.86}$	s(34.97%)p(64.96%)
THF		1.98142	-0.81942	C8	-0.30731	35.97%	$sp^{3.30}$	s(23.25%)p(76.62%)
				N4	-0.49762	64.03%	$sp^{2.07}$	s(32.60%)p(67.38%)
		1.97847	-0.80028	C2	-0.31628	51.13%	$sp^{1.36}$	s(42.36%)p(57.61%)
				C15	-0.10246	48.87%	$sp^{1.68}$	s(37.31%)p(62.66%)
		1.99176	-0.96913	C27	0.39031	32.43%	$sp^{3.03}$	s(24.74%)p(75.03%)
				O33	-0.52271	67.57%	$sp^{1.85}$	s(35.00%)p(64.93%)
MeOH		1.98132	-0.81993	C8	-0.30698	35.95%	$sp^{3.30}$	s(23.22%)p(76.65%)
				N4	-0.49600	64.05%	$sp^{2.07}$	s(32.60%)p(67.38%)
		1.97847	-0.80036	C2	-0.31734	51.14%	$sp^{1.36}$	s(42.35%)p(57.63%)
				C15	-0.09967	48.86%	$sp^{1.68}$	s(37.28%)p(62.68%)
		1.99182	-0.96965	C27	0.39174	32.45%	$sp^{3.03}$	s(24.78%)p(74.99%)
				O33	-0.52281	67.55%	$sp^{1.85}$	s(35.06%)p(64.87%)
MeCN		1.98132	-0.81995	C8	-0.30699	35.95%	$sp^{3.30}$	s(23.22%)p(76.65%)
				N4	-0.49596	64.05%	$sp^{2.07}$	s(32.60%)p(67.38%)

		1.97848	-0.80037	C2	-0.31736	51.14%	sp$^{1.36}$	s(42.35%)p(57.62%)
				C15	-0.09962	48.86%	sp$^{1.68}$	s(37.28%)p(62.68%)
		1.99182	-0.96962	C27	0.39175	32.45%	sp$^{3.03}$	s(24.78%)p(74.99%)
				O33	-0.52282	67.55%	sp$^{1.85}$	s(35.06%)p(64.87%)
DMSO		1.98084	-0.86487	C8	-0.30034	35.85%	sp$^{3.31}$	s(23.16%)p(76.71%)
				N4	-0.50191	64.15%	sp$^{2.07}$	s(32.62%)p(67.36%)
		1.97666	-0.79557	C2	-0.31036	50.63%	sp$^{1.37}$	s(42.11%)p(57.86%)
				C15	-0.10252	49.37%	sp$^{1.70}$	s(37.01%)p(62.96%)
		1.99084	-0.97179	C27	0.40269	32.45%	sp$^{3.07}$	s(24.53%)p(75.26%)
				O33	-0.52560	67.55%	sp$^{1.84}$	s(35.25%)p(64.68%)
**C7**								
TOL		1.98099	-0.81547	C8	-0.30065	35.88%	sp$^{3.30}$	s(23.20%)p(76.66%)
				N4	-0.50686	64.12%	sp$^{2.06}$	s(32.64%)p(67.35%)
		1.82596	-0.31580	C2	-0.30428	50.76%	sp$^{1.37}$	s(42.26%)p(57.70%)
				C15	-0.10903	49.24%	sp$^{1.69}$	s(37.18%)p(62.78%)
				C29	0.34193	32.55%	sp$^{3.03}$	s(24.75%)p(75.02%)
				O33	-0.52900	67.45%	sp$^{1.91}$	s(34.39%)p(65.54%)
$CHCl_3$		1.98097	-0.81661 5	C8	-0.30076	35.86%	sp$^{3.31}$	s(23.18%)p(76.69%)
				N4	-0.50478	64.14%	sp$^{2.06}$	s(32.64%)p(67.34%)
		1.97672	-0.80017	C2	-0.30644	50.78%	sp$^{1.37}$	s(42.25%)p(57.72%)
				C15	-0.10444	49.22%	sp$^{1.69}$	s(37.14%)p(62.82%)
		1.99209	-0.93647	C29	0.34321	32.56%	sp$^{3.03}$	s(24.77%)p(75.00%)
				O33	-0.53111	67.44%	sp$^{1.90}$	s(34.46%)p(65.47%)
THF		1.98094	-0.81713	C8	-0.30071	35.85%	sp$^{3.31}$	s(23.16%)p(76.70%)
				N4	-0.50379	64.15%	sp$^{2.06}$	s(32.64%)p(67.34%)
		1.97673	-0.80031	C2	-0.30727	50.79%	sp$^{1.37}$	s(42.24%)p(57.72%)
				C15	-0.10245	49.21%	sp$^{1.69}$	s(37.12%)p(62.84%)
		1.99213	-0.93639	C29	0.34370	32.56%	sp$^{3.03}$	s(24.78%)p(74.99%)
				O33	-0.53212	67.44%	sp$^{1.90}$	s(34.49%)p(65.44%)
MeOH		1.98087	-0.81790	C8	-0.30052	35.83%	sp$^{3.32}$	s(23.14%)p(76.72%)
				N4	-0.50218	64.17%	sp$^{2.06}$	s(32.64%)p(67.34%)
		1.97676	-0.80052	C2	-0.30839	50.81%	sp$^{1.37}$	s(42.23%)p(57.73%)
				C15	-0.09941	49.19%	sp$^{1.69}$	s(37.10%)p(62.87%)
		1.99218	-0.93636	C29	0.34435	32.56%	sp$^{3.02}$	s(24.79%)p(74.98%)
				O33	-0.53376	67.44%	sp$^{1.89}$	s(34.54%)p(65.39%)
MeCN		1.98087	-0.82144	C8	-0.30053	35.83%	sp$^{3.32}$	s(23.14%)p(76.72%)
				N4	-0.50214	64.17%	sp$^{2.06}$	s(32.64%)p(67.34%)

		1.97676	-0.80053	C2	-0.30841	50.81%	$sp^{1.37}$	s(42.23%)p(57.73%)
				C15	-0.09935	49.19%	$sp^{1.69}$	s(37.09%)p(62.87%)
		1.99219	-0.93944	C29	0.34435	32.56%	$sp^{3.02}$	s(24.79%)p(74.98%)
				O33	-0.53390	67.44%	$sp^{1.89}$	s(34.54%)p(65.39%)
DMSO		1.98087	-0.82147	C8	-0.30051	35.83%	$sp^{3.32}$	s(23.14%)p(76.72%)
				N4	-0.50202	64.17%	$sp^{2.06}$	s(32.64%)p(67.34%)
		1.97676	-0.80055	C2	-0.30847	50.81%	$sp^{1.37}$	s(42.23%)p(57.73%)
				C15	-0.09913	49.19%	$sp^{1.69}$	s(37.09%)p(62.87%)
		1.99219	-0.93945	C29	0.34439	32.56%	$sp^{3.02}$	s(24.79%)p(74.98%)
				O33	-0.53402	67.44%	$sp^{1.89}$	s(34.54%)p(65.39%)

Table S3. CT parameters for the bright low-lying excited state. Values q_{CT} are given in [e] and D_{CT} in [Å]

	q_{CT}	D_{CT}	q_{CT}	D_{CT}	q_{CT}	D_{CT}	q_{CT}	D_{CT}
	**C1**		**C2**		**C3**		**C4**	
TOL	0.830	1.930	0.832	1.880	0.469	1.194	0.838	1.860
$CHCl_3$	0.828	2.131	0.492	2.419	0.462	1.496	0.889	3.400
THF	0.475	2.404	0.494	2.545	0.463	1.588	0.915	3.561
MeOH	0.480	2.584	0.500	2.706	0.464	1.725	0.930	3.686
MeCN	0.480	2.587	0.500	2.709	0.465	1.731	0.930	3.689
DMSO	0.485	2.579	0.504	2.700	0.468	1.746	0.928	3.693
	**C5**		**C6**		**C7**		**C8**	
TOL	0.846	1.915	0.470	1.268	0.539	2.690	0.557	3.441
$CHCl_3$	0.842	2.095	0.461	1.523	0.549	2.943	0.573	3.771
THF	0.840	2.179	0.460	1.571	0.552	3.054	0.580	3.914
MeOH	0.840	2.313	0.455	1.679	0.558	3.226	0.591	4.125
MeCN	0.840	2.316	0.456	1.686	0.558	3.226	0.592	4.126
DMSO	0.839	2.323	0.460	1.704	0.528	3.134	0.593	4.100

Table S4. Free energies of solvation (kcal/mol)

	ΔG_{sol}			
	**C1**	**C2**	**C3**	**C4**
TOL	-15.55	-18.37	-15.92	-14.94
$CHCl_3$	-18.06	-19.14	-19.53	-16.18
THF	-19.03	-19.94	-19.59	-18.56
MeOH	-20.11	-20.99	-23.53	-20.41
MeCN	-20.68	-21.35	-21.40	-20.41
DMSO	-19.61	-20.02	-20.10	-18.91
	**C5**	**C6**	**C7**	**C8**
TOL	-14.51	-17.77	-18.91	-19.39
$CHCl_3$	-17.12	-19.80	-22.61	-20.91
THF	-18.30	-20.90	-22.24	-22.89
MeOH	-19.60	-22.58	-23.04	-22.14
MeCN	-19.01	-23.22	-23.74	-23.20
DMSO	-17.26	-21.61	-20.12	-21.55

Table S5. Linear optical properties (λ_{abs}, nm)

	λ_{abs}	f	λ_{cLR}	λ_{abs}	f	λ_{cLR}	λ_{abs}	f	λ_{cLR}	λ_{abs}	f	λ_{cLR}
		**C1**			**C2**			**C3**			**C4**	
TOL	398.95	1.0240	407.20	408.55	1.0107	408.37	405.99	0.9183	400.74	376.02	0.8142	388.42
$CHCl_3$	394.35	1.0125	403.95	404.88	0.9957	393.87	408.03	0.9423	395.84	376.16	0.8198	401.29
THF	392.52	1.0031	390.27	405.16	0.9888	391.82	408.37	0.9352	394.99	375.41	0.8201	406.92
MeOH	392.25	0.9819	386.68	404.92	0.9685	388.11	408.25	0.9161	396.54	375.34	0.7997	410.19
MeCN	392.8	0.9862	386.68	405.45	0.9729	388.14	408.78	0.9209	396.65	375.75	0.8028	411.44
DMSO	396.87	1.0203	387.31	409.42	1.0072	388.86	412.81	0.9586	397.34	378.85	0.8264	420.22
		**C5**			**C6**			**C7**			**C8**	
TOL	389.33	0.9625	399.04	408.68	0.9254	398.19	418.92	0.8024	428.67	408.67	1.1938	418.68
$CHCl_3$	390.71	0.9526	396.24	411.94	0.9513	397.22	422.52	0.7922	432.13	412.13	1.1628	425.13
THF	390.82	0.9437	394.34	412.84	0.9517	398.45	423.43	0.7836	432.95	412.95	1.1422	427.58
MeOH	390.51	0.9252	390.33	413.34	0.9420	400.60	424.03	0.7654	433.43	413.43	1.1035	430.62
MeCN	390.97	0.9291	390.79	413.91	0.9467	400.71	424.59	0.7693	434.00	414.00	1.1092	431.07
DMSO	394.43	0.9593	394.70	418.15	0.9828	401.34	430.78	0.8674	438.18	418.18	1.1579	433.89

Table S6. Calculated values of dipole moments (in D) for the ground and CT excited state

	μ_{GS}	μ_{CT}	μ_{GS}	μ_{CT}	μ_{GS}	μ_{CT}	μ_{GS}	μ_{CT}
	**C1**		**C2**		**C3**		**C4**	
TOL	9.03	10.99	9.72	18.14	10.66	19.71	9.46	12.78
$CHCl_3$	9.79	11.36	10.53	19.88	11.59	21.92	10.12	13.86
THF	10.13	12.70	10.88	20.83	12.10	22.89	10.42	14.37
MeOH	10.67	15.06	11.43	21.89	12.66	24.38	10.87	15.20
MeCN	10.68	15.09	11.44	21.92	12.68	24.42	10.88	15.23
DMSO	10.72	15.09	11.48	21.96	12.72	24.53	10.92	15.32
	**C5**		**C6**		**C7**		**C8**	
TOL	7.86	20.58	12.34	22.94	8.34	10.38	9.89	16.20
$CHCl_3$	8.48	22.22	13.50	25.00	8.99	11.43	10.68	19.03
THF	8.77	22.96	14.06	26.07	9.28	12.07	11.03	19.98
MeOH	9.23	24.10	14.91	27.26	9.71	12.59	11.58	20.83
MeCN	9.26	24.13	14.93	27.29	10.08	12.63	11.59	20.88
DMSO	9.28	24.21	14.99	27.37	10.09	13.93	11.63	21.07

Table S7. TD-DFT fluorescence maxima (λ_{em}) of compounds C1–C8 in different solvents.

	**C1**	C2	C3	C4
TOL	464.05	470.30	475.45	439.30
$CHCl_3$	457.67	474.17	476.17	435.53
THF	454.83	474.08	475.98	437.66
MeOH	454.11	473.48	475.12	440.60
MeCN	454.66	474.01	475.66	440.75
DMSO	458.70	477.96	479.64	441.50
	**C5**	C6	C7	C8
TOL	467.99	467.13	498.33	476.99
$CHCl_3$	492.28	472.35	500.72	489.76
THF	489.64	472.40	498.05	484.26
MeOH	485.45	471.93	500.76	490.25
MeCN	500.37	472.50	509.36	495.81
DMSO	503.29	476.69	508.81	502.96

Table S8. Nonlinear properties. Values are given in (a.u.)

	α	β	α	β	α	β	α	β
	**C1**		**C2**		**C3**		**C4**	
TOL	303.85	4236.81	324.29	4391.25	326.13	3954.74	311.44	1952.79
$CHCl_3$	331.21	5596.81	366.55	6469.19	356.17	5338.88	337.06	2577.62
THF	344.17	6275.90	388.89	7611.96	370.53	6051.90	349.28	2883.84
MeOH	365.30	7406.70	389.52	7646.25	394.16	7277.19	369.38	3385.81
MeCN	365.89	7440.28	389.54	7667.12	394.83	7315.23	369.95	3400.46
DMSO	367.45	7524.91	391.17	7730.61	396.58	7408.39	371.44	3437.29
	**C5**		**C6**		**C7**		**C8**	
TOL	336.49	3029.01	345.57	3469.66	367.98	5203.31	397.09	9745.89
$CHCl_3$	364.44	3956.61	377.76	4738.89	402.67	7076.08	433.63	13070.65
THF	377.81	4412.90	393.42	5386.92	419.26	8053.14	450.86	14739.09
MeOH	399.93	5172.92	419.19	6527.54	446.41	9744.53	478.84	17524.27
MeCN	400.55	5172.92	419.90	6565.22	447.18	9794.81	479.62	17604.05
DMSO	402.19	5246.59	421.80	6652.90	456.55	9933.82	481.67	17810.46

Table S9. AutoDock binding free energies (ΔG_b) for compounds C1–C8 docked to Concanavalin A (ConA)

	**C1**	**C2**	**C3**	**C4**	**C5**	**C6**	**C7**	**C8**
30	-2.8	-2.8	-2.5	-2.7	-2.6	-2.6	-2.7	-3.2
35	-3.8	-3.9	-3.8	-3.9	-3.6	-3.7	-4.3	-4.2
36	-3.3	-3.5	-3.4	-3.6	-3.4	-3.2	-4.0	-4.1
39	-3.3	-3.4	-3.4	-3.4	-3.3	-3.0	-3.7	-3.6
46	-3.5	-3.5	-3.5	-3.5	-3.4	-3.4	-4.0	-3.9
59	-3.4	-3.4	-3.3	-3.5	-3.3	-3.2	-3.9	-3.8
101	-4.4	-4.3	-3.9	-4.0	-3.8	-3.6	-4.3	-4.5
114	-4.0	-4.2	-4.0	-4.1	-4.0	-4.0	-4.5	-4.5
116	-4.2	-4.2	-4.2	-4.2	-3.9	-3.7	-4.6	-5.2
135	-2.7	-2.8	-2.9	-2.8	-2.8	-2.9	-3.1	-3.1
138	-3.7	-3.6	-3.6	-3.8	-3.1	-3.4	-4.0	-4.0
200	-2.8	-2.9	-2.8	-2.7	-2.9	-2.8	-3.4	-3.1
TER	-2.9	-3.0	-3.0	-3.0	-2.9	-3.1	-3.5	-3.4

Table S10. AutoDock binding free energies (ΔG_b) for compounds C1–C8 docked to Human serum Albuminum (HSA)

	**C1**	**C2**	**C3**	**C4**	**C5**	**C6**	**C7**	**C8**
34	-4.6	-4.6	-4.7	-4.7	-4.7	-4.5	-5.2	-5.0
53	-3.4	-3.0	-3.7	-3.3	-3.3	-2.7	-4.0	-3.7
62	-3.5	-3.6	-3.8	-3.7	-3.7	-3.7	-4.3	-3.9
75	-4.7	-4.6	-4.6	-4.9	-4.6	-4.4	-5.5	-5.6
90	-2.9	-3.1	-2.8	-3.0	-2.6	-2.7	-3.4	-3.3
91	-5.2	-4.7	-5.0	-4.4	-4.6	-4.5	-5.1	-5.6
101	-4.9	-4.3	-4.8	-4.5	-4.0	-3.1	-3.9	-5.6
124	-4.0	-3.7	-4.3	-4.0	-3.7	-3.2	-4.3	-4.1
168	-4.5	-4.5	-4.2	-4.4	-4.0	-3.6	-4.4	-4.7
169	-4.4	-4.2	-4.2	-4.1	-3.9	-3.3	-4.2	-4.6
177	-4.6	-4.5	-4.5	-4.3	-4.4	-4.2	-4.9	-5.1
200	-5.0	-4.9	-4.6	-4.6	-4.0	-3.5	-4.4	-5.5

245	-4.8	0.9	0.5	0.2	0.7	0.5	0.9	0.4
246	-4.8	-5.2	-5.0	-5.0	-5.0	-4.4	-5.6	-5.9
253	-3.1	-2.6	-1.7	-2.3	-1.5	-1.4	-2.4	3.8
265	-4.1	-4.9	-4.4	-3.9	-3.5	-3.6	-4.0	-4.2
278	-4.3	-4.0	-4.2	-3.8	-3.4	-3.5	-3.9	-4.3
279	-4.3	-4.5	-4.1	-4.6	-4.0	-3.8	-4.6	-4.3
289	-4.5	-4.5	-4.5	-4.7	-4.8	-4.5	-5.1	-5.1
316	-4.7	-4.9	-4.6	-4.7	-4.8	-4.4	-5.1	-5.5
360	-3.9	-4.0	-3.7	-4.4	-3.8	-3.6	-4.3	-3.6
361	-4.6	-4.7	-4.5	-4.7	-4.6	-4.2	-5.1	-5.5
369	-3.6	-3.6	-3.6	-3.9	-3.6	-3.6	-3.7	-4.2
392	-3.3	-2.9	-3.3	-3.1	-2.9	-3.2	-3.6	-3.2
437	-4.3	-4.4	-4.3	-4.6	-4.3	-4.3	-5.0	-5.0
438	-4.4	-4.5	-4.8	-4.1	-3.8	-3.9	-4.9	-5.6
448	-6.6	-6.3	-6.8	-6.4	-6.4	-6.4	-7.5	-6.8
461	-5.4	-3.7	-1.3	-0.9	31.7	-0.1	-2.9	0.9
476	-4.4	-4.2	-4.4	-4.4	-4.0	-3.7	-4.7	-3.8
477	-4.2	-4.5	-4.1	-5.2	-3.7	-3.5	-4.3	-2.6
487	-4.7	-4.7	-4.9	-5.0	-5.0	-4.7	-5.2	-5.2
514	-4.5	-4.5	-4.5	-4.5	-4.3	-4.4	-5.0	-5.2
558	-3.2	-3.0	-3.1	-3.1	-3.1	-2.9	-3.4	-3.3
559	-3.9	-3.9	-4.0	-4.0	-3.9	-3.9	-4.5	-4.3
567	-0.3	-0.3	-0.2	-0.3	-0.2	-0.2	-0.4	-0.3

Table S11. Predicted LogP and bioaccumulation factor (LogBCF) for compounds **C1–C8**.

	logP	logBCF
C1	1.92	0.533
C2	1.93	0.416
C3	1.93	0.460
C4	1.93	0.503
C5	1.94	0.397
C6	1.94	0.468
C7	3.07	0.887
C8	3.07	0.816

Table S12. Predicted acute toxicity for rats (LD_{50}). Values given in mg/kg body weight

Route of administration	**C1**	**C2**	**C3**	**C4**	**C5**	**C6**	**C7**	**C8**
Intraperitoneal	664,50	582,40	767,80	766,00	912,60	761,60	829,10	905,80
Intravenous	342,90	410,02	483,20	448,20	381,40	370,90	391,20	570,90
Oral	2444,00	1253,00	2475,00	1988,00	2019,00	2246,00	1912,00	1387,00
Subcutaneous	1359,00	1770,00	1038,00	1648,00	1864,00	1729,00	825,20	1263,00

Table S13. Predicting the toxicity of tested compounds. PR – prediction (In – inactive; A – active); P% - probability expressed as a percentage

	**C1**		**C2**		**C3**		**C4**		**C5**		**C6**		**C7**		**C8**	
Target	PR	P%	PR	P%	PR	P%	PR	P%	PR	P%	PR	P%	PR	P%	PR	P%
	Metabolism															
Cytochrome CYP1A2	In	0.79	In	0.78	In	0.78	In	0.79	In	0.75	In	0.76	In	0.77	In	0.77
Cytochrome CYP2C19	In	0.79	In	0.82	In	0.83	In	0.82	In	0.84	In	0.83	In	0.7	In	0.7
Cytochrome CYP2C9	In	0.62	In	0.62	In	0.63	In	0.66	In	0.65	In	0.63	A	0.6	A	0.6
Cytochrome CYP2D6	In	0.79	In	0.76	In	0.78	In	0.81	In	0.78	In	0.78	In	0.79	In	0.79
Cytochrome CYP3A4	In	0.79	In	0.77	In	0.77	In	0.77	In	0.77	In	0.76	In	0.55	In	0.55
Cytochrome CYP2E1	In	0.99	In	1	In	1	In	0.99	In	1	In	1	In	0.99	In	0.99
	Organ toxicity															
Hepatotoxicity	In	0.57	In	0.56	In	0.57	In	0.59	In	0.57	In	0.57	A	0.5	A	0.5
Neurotoxicity	In	0.55	In	0.56	In	0.56	In	0.57	In	0.57	In	0.56	In	0.5	In	0.5
Nephrotoxicity	A	0.54	A	0.52	A	0.51	A	0.52	A	0.5	A	0.51	A	0.55	A	0.55
Respiratory toxicity	A	0.70	A	0.72	A	0.7	A	0.7	A	0.71	A	0.7	A	0.74	A	0.74
Cardiotoxicity	In	0.69	In	0.7	In	0.69	In	0.69	In	0.68	In	0.69	In	0.73	In	0.73
	Toxicity end points															
Carcinogenicity	In	0.59	In	0.6	In	0.61	In	0.61	In	0.6	In	0.6	In	0.59	In	0.59
Immunotoxicity	In	0.92	In	0.66	In	0.87	In	0.9	A	0.65	In	0.85	In	0.74	In	0.57
Mutagenicity	In	0.75	In	0.74	In	0.73	In	0.73	In	0.72	In	0.73	In	0.63	In	0.63
Cytotoxicity	In	0.70	In	0.65	In	0.68	In	0.7	In	0.64	In	0.67	In	0.64	In	0.64
BBB-barrier	A	0.53	A	0.54	A	0.53	A	0.51	A	0.53	A	0.52	A	0.5	A	0.5
Ecotoxicity	In	0.67	In	0.66	In	0.66	In	0.66	In	0.66	In	0.64	In	0.63	In	0.63
Clinical toxicity	A	0.67	A	0.66	A	0.69	A	0.68	A	0.65	A	0.66	A	0.7	A	0.7
Nutritional toxicity	A	0.55	A	0.57	A	0.56	A	0.56	A	0.57	A	0.57	A	0.52	A	0.52

6. Optical properties

Table S14. Absorption and emission spectra of C-series in various solvents at room temperature

<table>
<tr>
<td>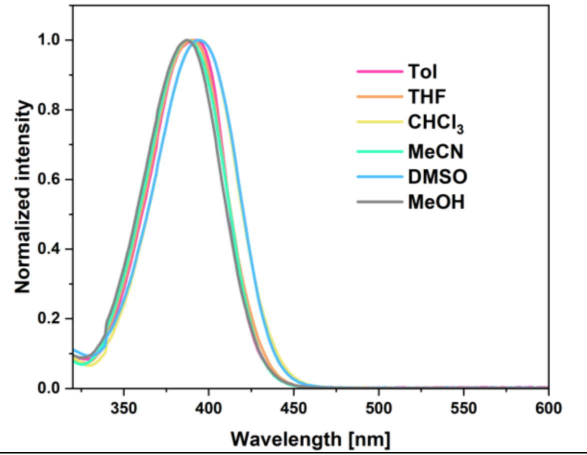
</td>
<td>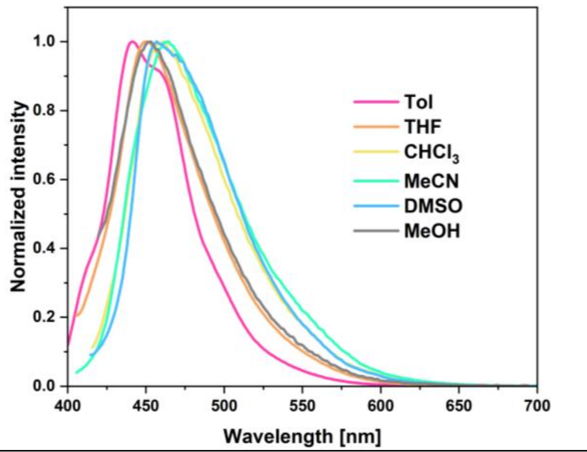
</td>
</tr>
<tr><td>Absorption of C-1</td><td>Emission of C-1</td></tr>
<tr>
<td>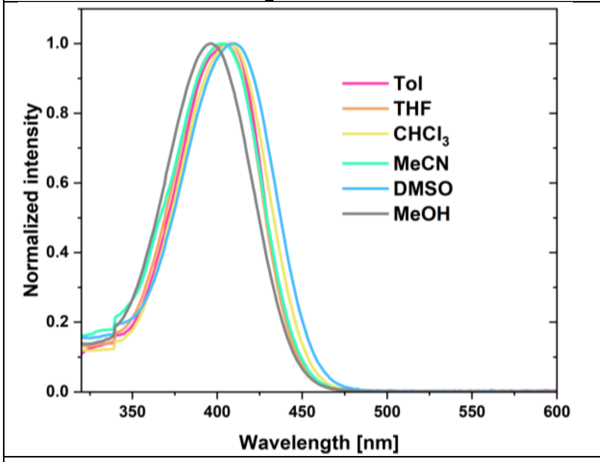
</td>
<td>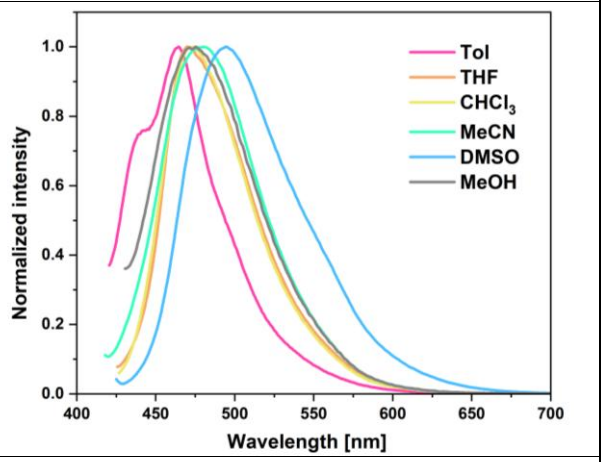
</td>
</tr>
<tr><td>Absorption of C-2</td><td>Emission of C-2</td></tr>
<tr>
<td>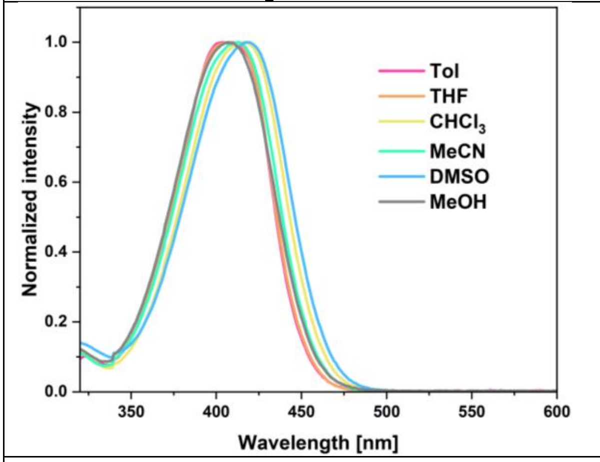
</td>
<td>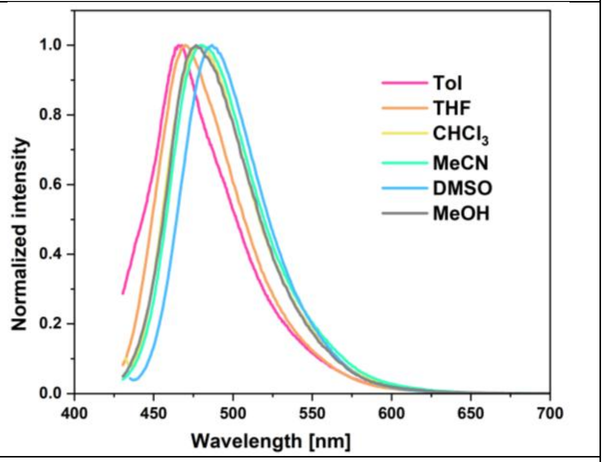
</td>
</tr>
<tr><td>Absorption of C-3</td><td>Emission of C-3</td></tr>
</table>

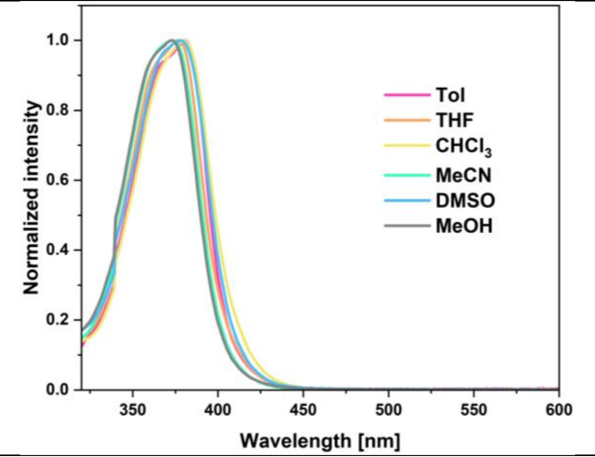

Absorption of C-4

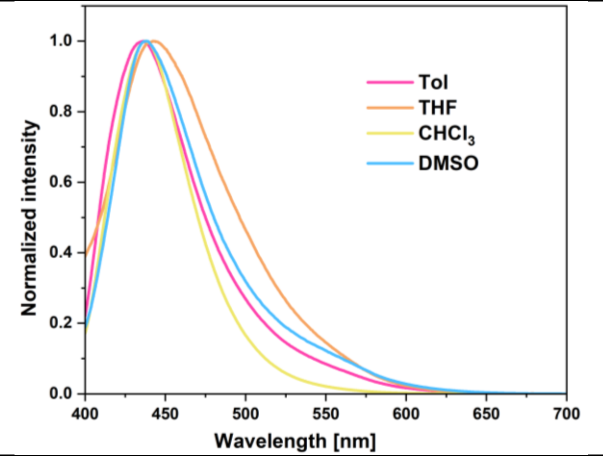

Emission of C-4

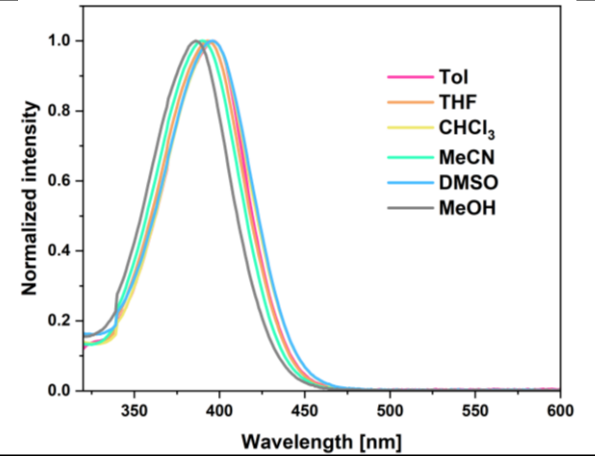

Absorption of C-5

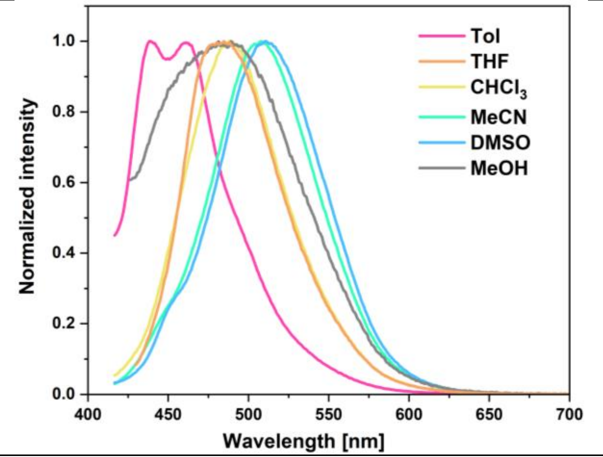

Emission of C-5

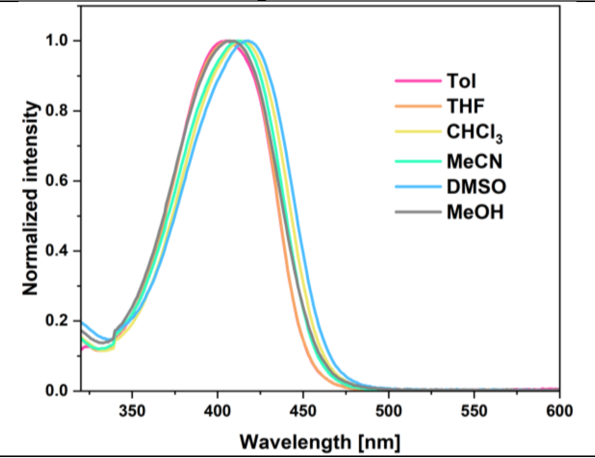

Absorption of C-6

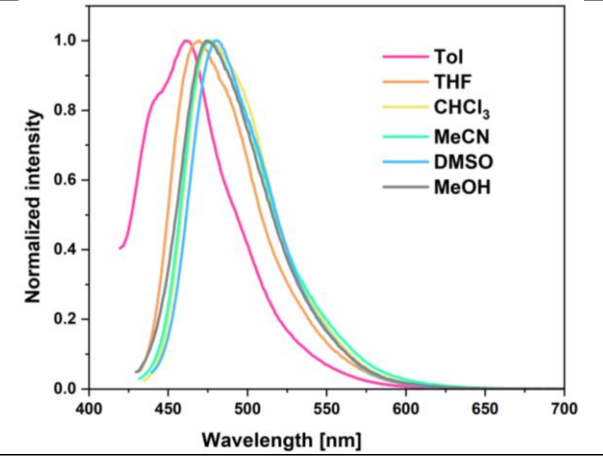

Emission of C-6

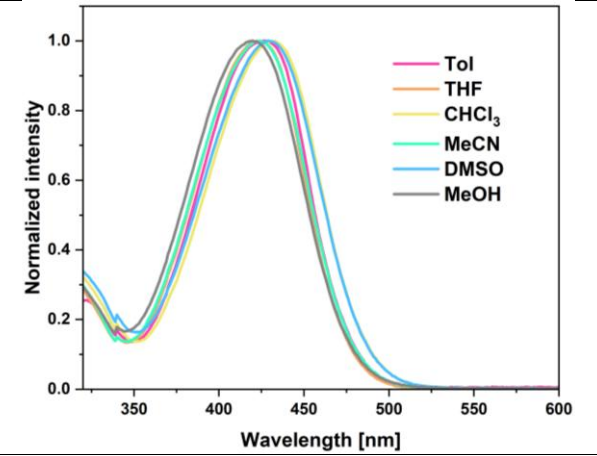

Absorption of C-7

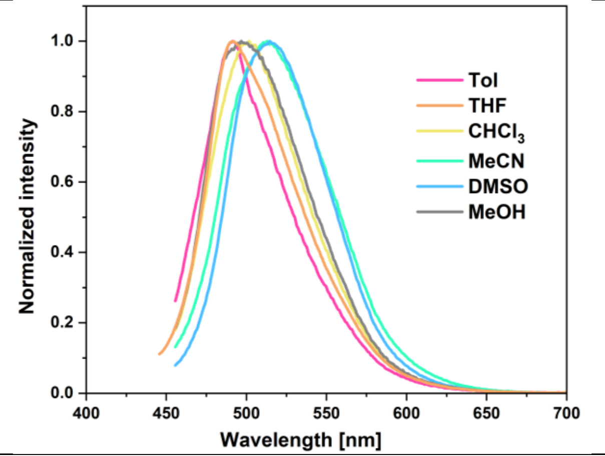

Emission of C-7

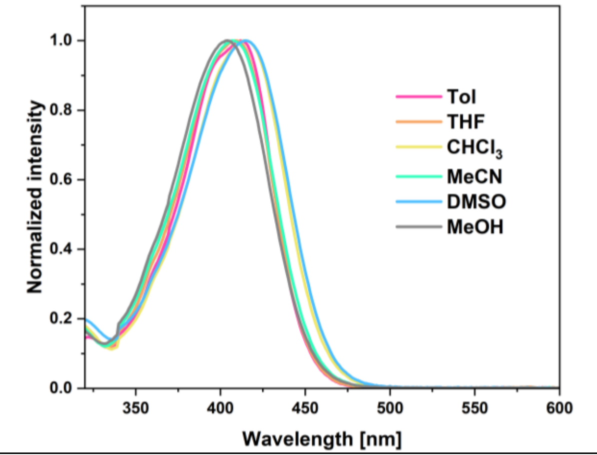

Absorption of C-8

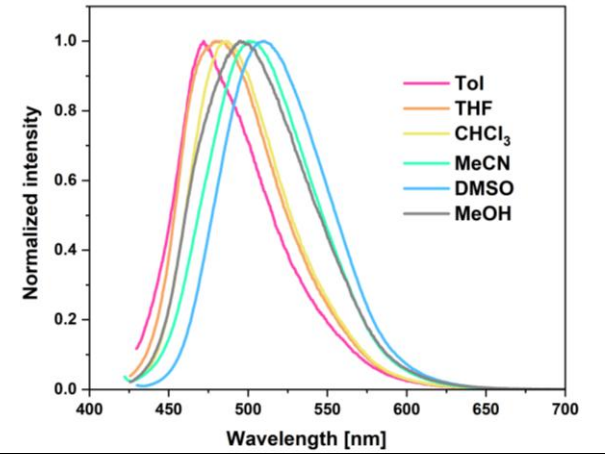

Emission of C-8

Table S15. Comparison of normalized absorption spectra of compounds C-1 – C-8.

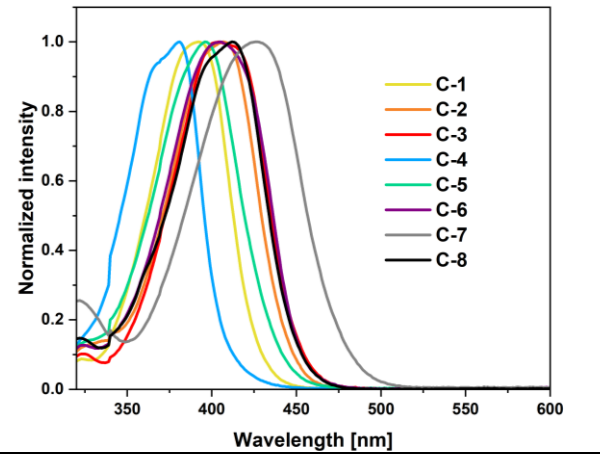

Tol

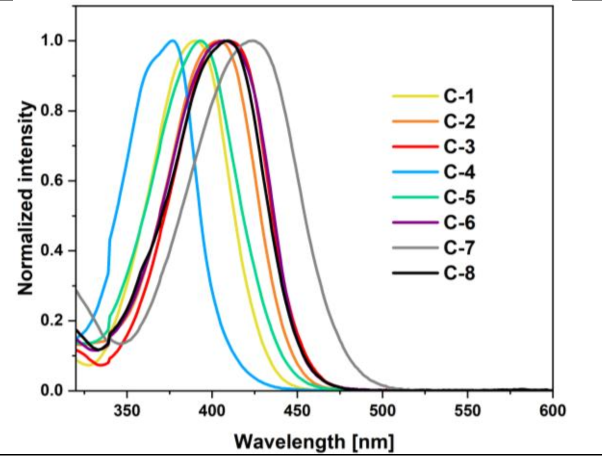

THF

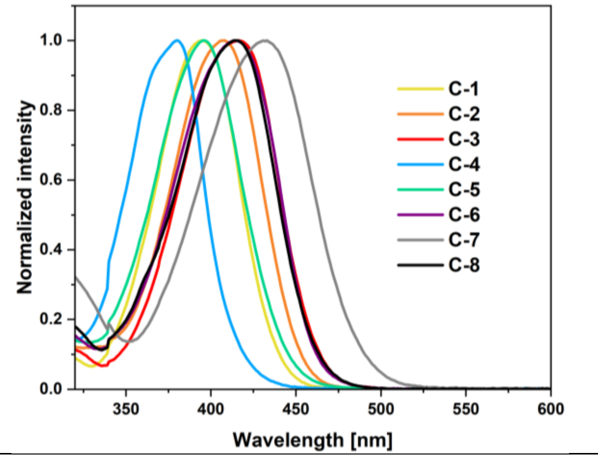

CHCl$_3$

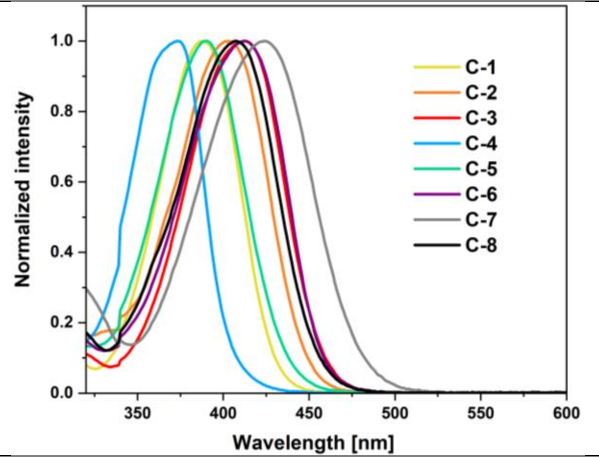

MeCN

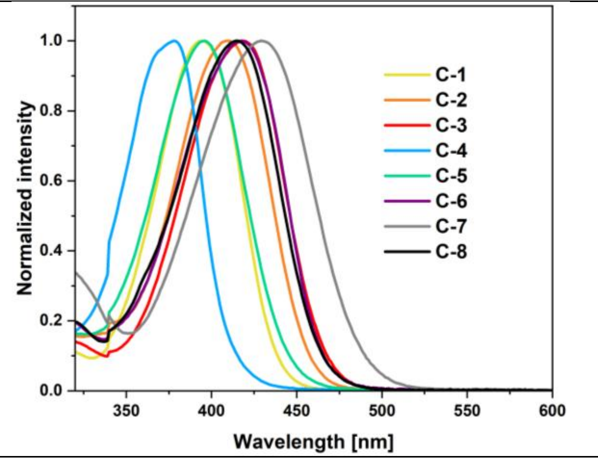

DMSO

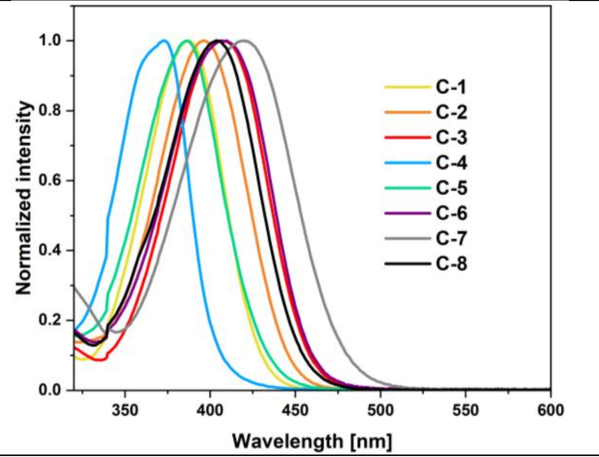

MeOH

Table S16. UV-Vis spectra in DMSO recorded at time intervals at room temperature

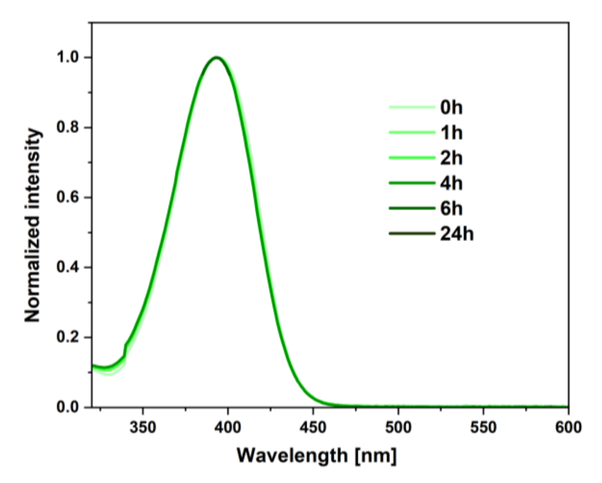

C-1

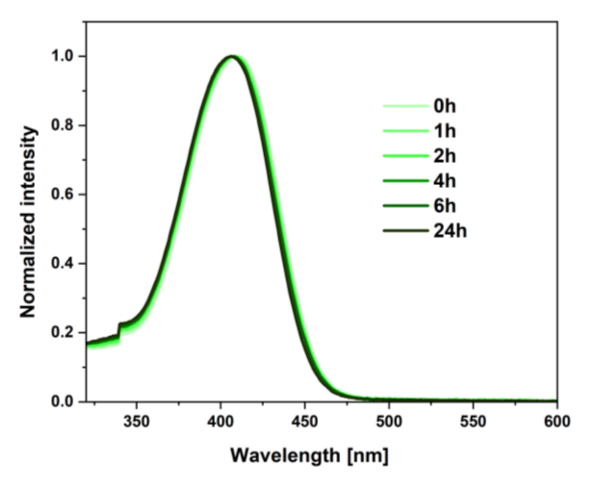

C-2

<table>
<tr><td>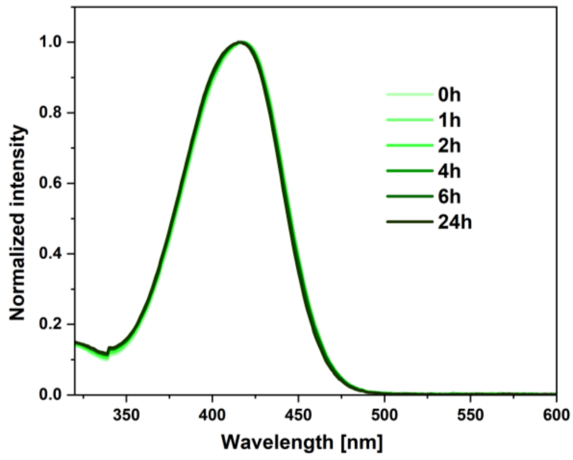
</td><td>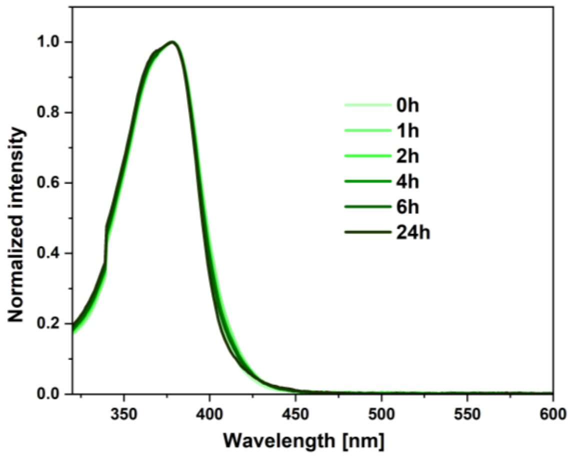
</td></tr>
<tr><td>C-3</td><td>C-4</td></tr>
<tr><td>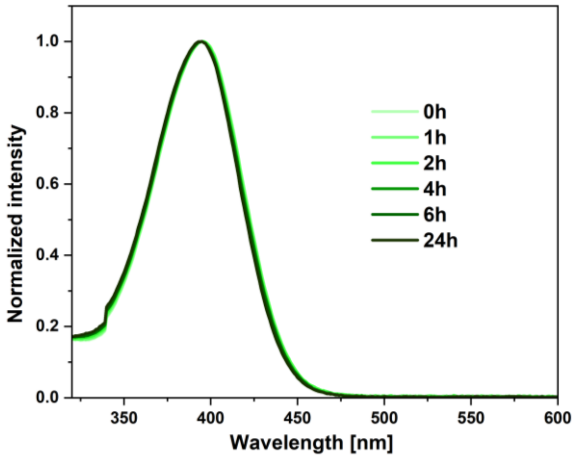
</td><td>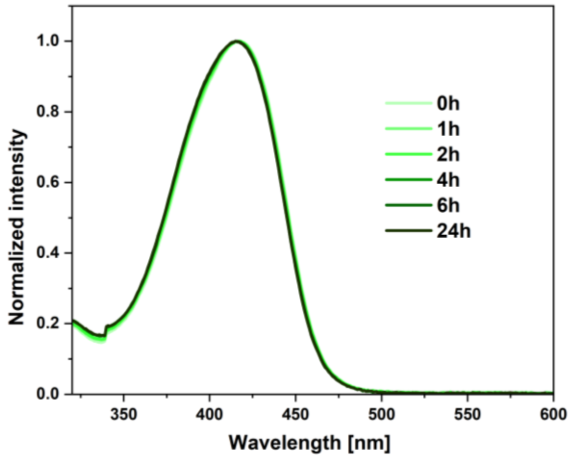
</td></tr>
<tr><td>C-5</td><td>C-6</td></tr>
<tr><td>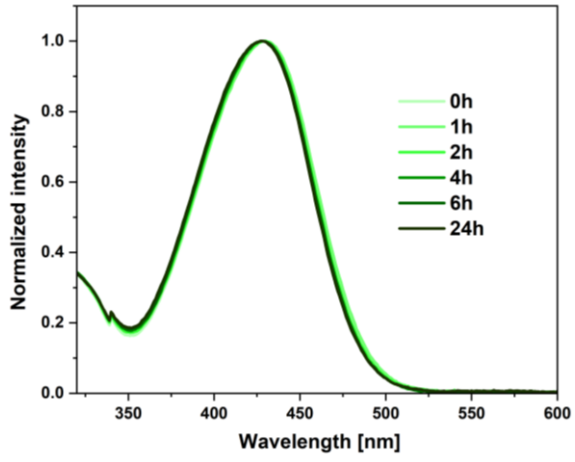
</td><td>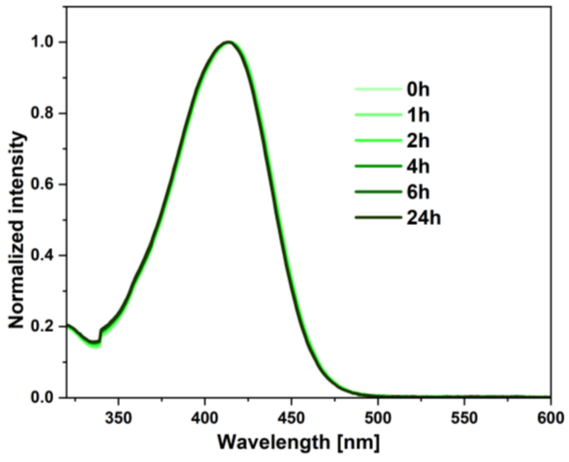
</td></tr>
<tr><td>C-7</td><td>C-8</td></tr>
</table>

Table S17. Comparison of normalized emission spectra of compounds C-series.

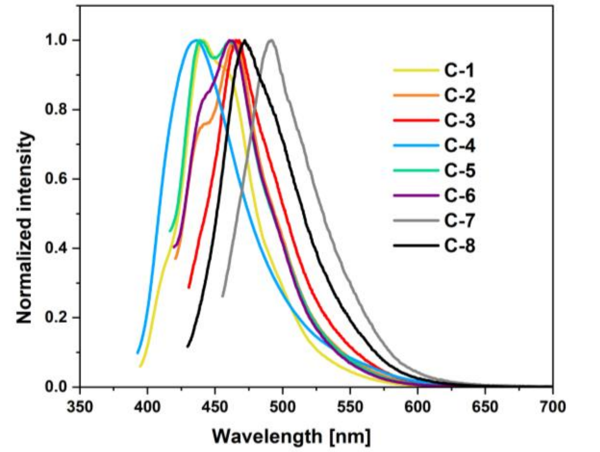

Tol

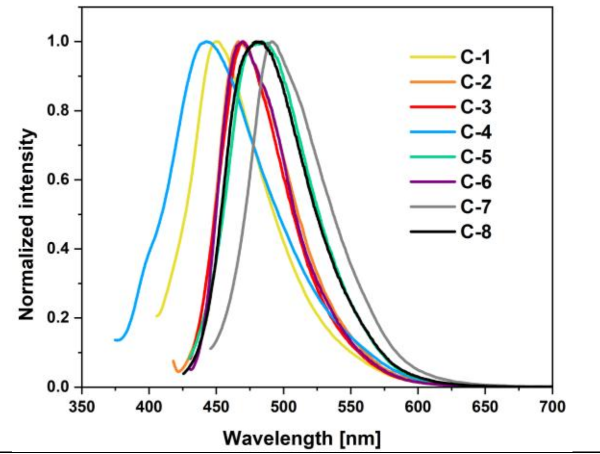

THF

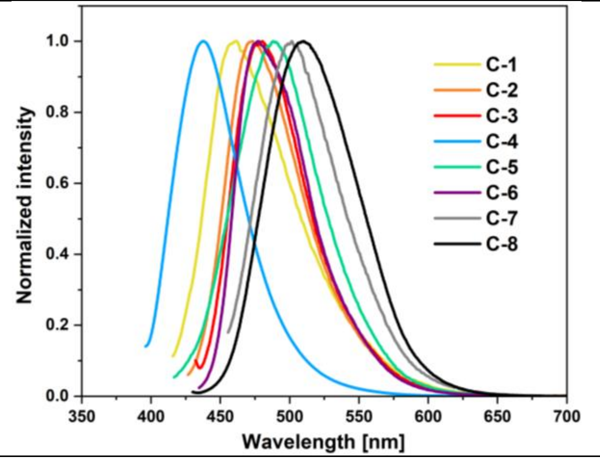

$CHCl_3$

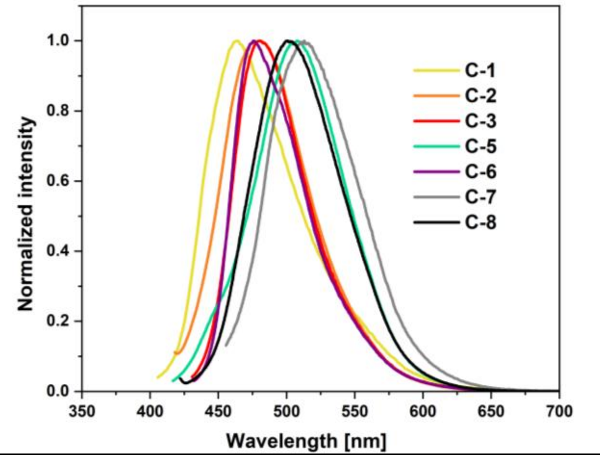

MeCN

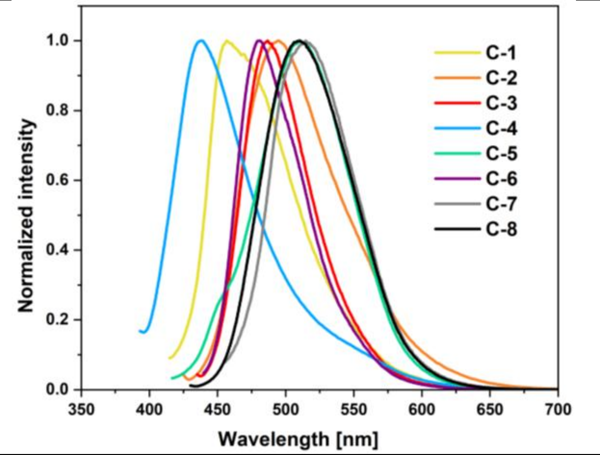

DMSO

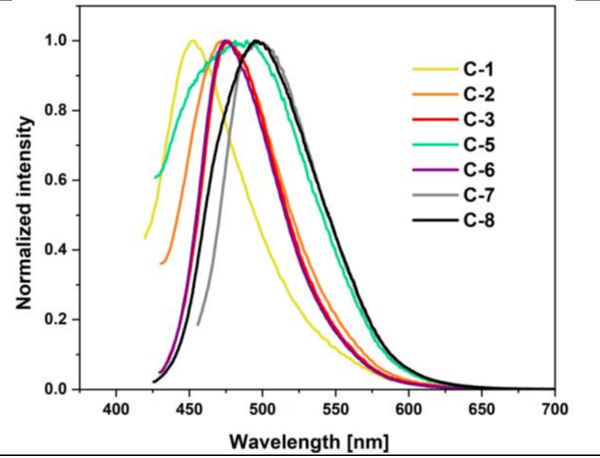

MeOH

Table S18. The Lippert-Mataga plots

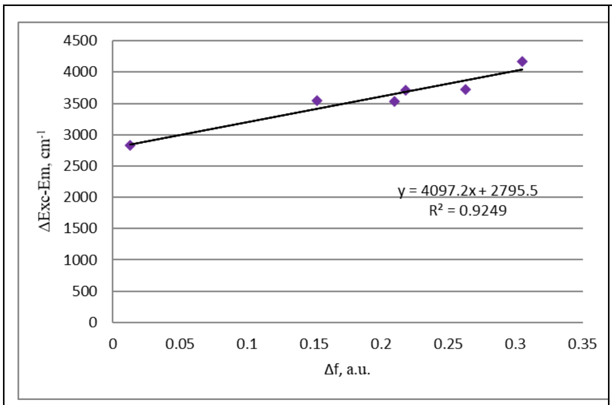

C-1

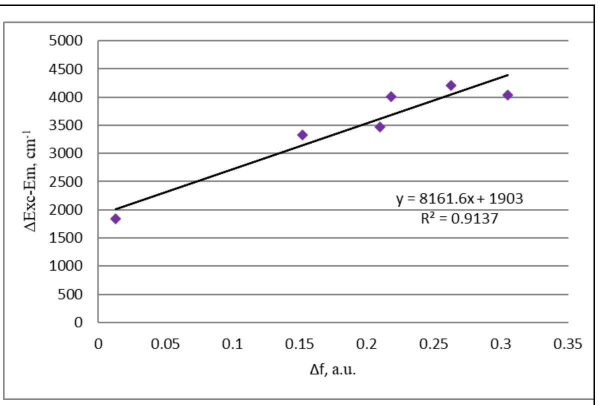

C-2

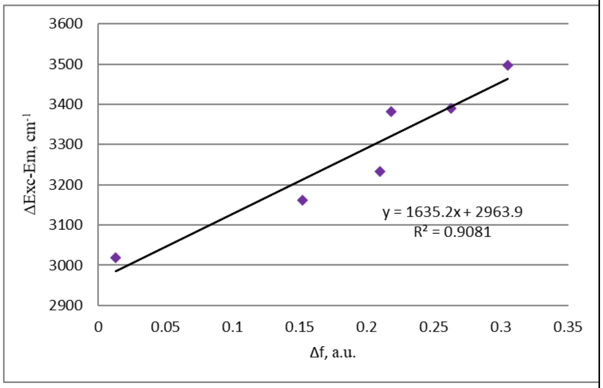

C-3

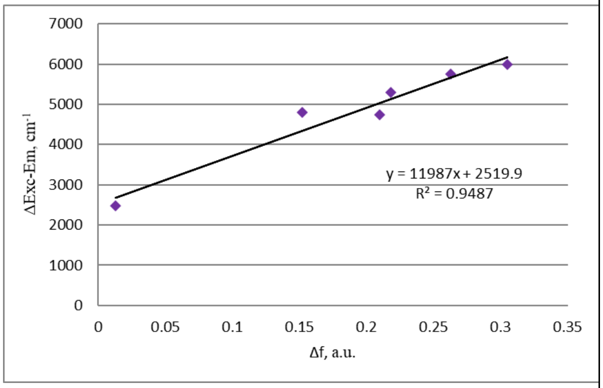

C-5

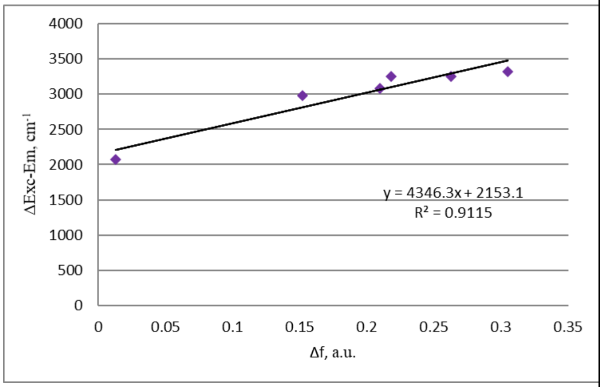

C-6

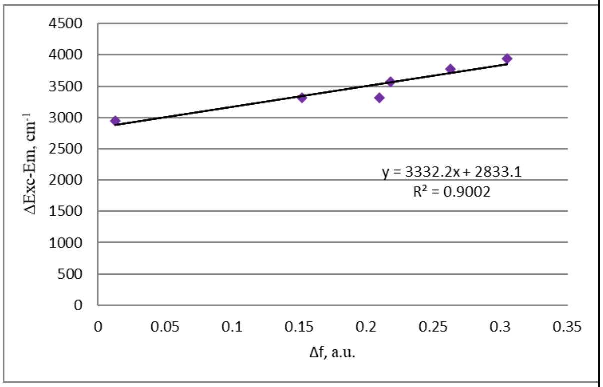

C-7

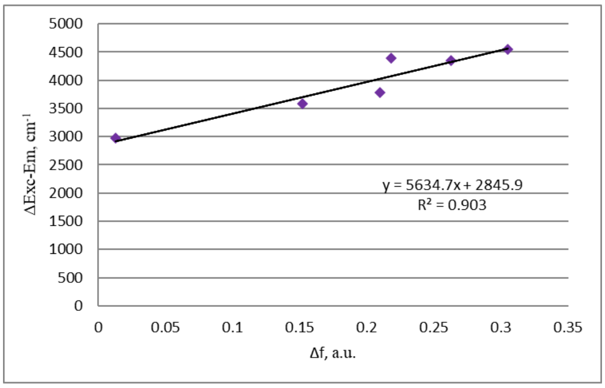

C-8

7. Literature

1. European Committee for Antimicrobial Susceptibility Testing (EUCAST) of the European Society of Clinical Microbiology and Infectious Diseases (ESCMID) Determination of Minimum Inhibitory Concentrations (MICs) of Antibacterial Agents by Broth Dilution. Clinical Microbiology and Infection 2003, 9, ix–xv, doi:10.1046/j.1469-0691.2003.00790.x.

2 Clinical and Laboratory Standards Institute. Reference method for broth dilution antifungal susceptibility testing of yeasts. M27-S4. Clinical and Laboratory Standards Institute, Wayne, PA, USA, 2012.

3 Biernasiuk, A.; Kawczyńska, M.; Berecka-Rycerz, A.; Rosada, B.; Gumieniczek, A.; Malm, A.; Dzitko, K.; Łączkowski, K.Z. Synthesis, Antimicrobial Activity, and Determination of the Lipophilicity of ((Cyclohex-3-Enylmethylene)Hydrazinyl)Thiazole Derivatives. Med Chem Res 2019, 28, 2023–2036, doi:10.1007/s00044-019-02433-2.

4 Wiegand, I.; Hilpert, K.; Hancock, R.E.W. Agar and Broth Dilution Methods to Determine the Minimal Inhibitory Concentration (MIC) of Antimicrobial Substances. Nat Protoc 2008, 3, 163–175, doi:10.1038/nprot.2007.521.

5 O'Donnell, F.; Smyth, T.J.P.; Ramachandran, V.N.; Smyth, W.F. A Study of the Antimicrobial Activity of Selected Synthetic and Naturally Occurring Quinolines. International Journal of Antimicrobial Agents 2010, 35, 30–38, doi:10.1016/j.ijantimicag.2009.06.031.

6 M.J. Frisch, G.W. Trucks, G.B. Schlegel et al. Gaussian 09, Revision A.1, Gaussian, Inc., Wallingford CT, (2009).

7 Adamo, C.; Scuseria, G.E.; Barone, V. Accurate Excitation Energies from Time-Dependent Density Functional Theory: Assessing the PBE0 Model. J. Chem. Phys. 1999, 111, 2889–2899, doi:10.1063/1.479571.

8 Guido, C.; Caprasecca, S. How to Perform Corrected Linear Response Calculations in G09; 2016;

9 Kula, S.; Kaźmierski, Ł.; Filapek, M.; Krawczyk, P. Influence of a Bithiophene Substituent on the Properties of a New Fluorescent Probe – Theoretical and Experimental Study. Journal of Molecular Structure 2023, 1281, 135151, doi:10.1016/j.molstruc.2023.135151.

10 Kula, S.; Krawczyk, P.; Kaźmierski, Ł.; Filapek, M. Synthesis, Physicochemical Characterization and Biological Properties of New 5-(1H-Phenanthro[9,10-d]Imidazol-2-Yl)-Thiophene-2-Carbaldehyde. Journal of Molecular Structure 2022, 1252, 132122, doi:10.1016/j.molstruc.2021.132122.

11 Kula, S.; Krawczyk, P.; Filapek, M.; Maroń, A.M. Influence of N-Donor Substituents on Physicochemical Properties of Phenanthro[9,10-d]Imidazole Derivatives. Journal of Luminescence 2021, 233, 117910, doi:10.1016/j.jlumin.2021.117910.

12 Caricato, M. A Comparison between State-Specific and Linear-Response Formalisms for the Calculation of Vertical Electronic Transition Energy in Solution with the CCSD-PCM Method. J. Chem. Phys. 2013, 139, 044116, doi:10.1063/1.4816482.

13 Le Bahers, T.; Adamo, C.; Ciofini, I. A Qualitative Index of Spatial Extent in Charge-Transfer Excitations. J. Chem. Theory Comput. 2011, 7, 2498–2506, doi:10.1021/ct200308m.

14 Cancès, E.; Mennucci, B.; Tomasi, J. A New Integral Equation Formalism for the Polarizable Continuum Model: Theoretical Background and Applications to Isotropic and Anisotropic Dielectrics. J. Chem. Phys. 1997, 107, 3032–3041, doi:10.1063/1.474659.

15 Potemkin, V.; Grishina, M. Principles for 3D/4D QSAR Classification of Drugs. Drug Discovery Today 2008, 13, 952–959, doi:10.1016/j.drudis.2008.07.006.

16 Potemkin, V.A.; Grishina, M.A. A New Paradigm for Pattern Recognition of Drugs. J Comput Aided Mol Des 2008, 22, 489–505, doi:10.1007/s10822-008-9203-x.

17 Potemkin, V.A.; Pogrebnoy, A.A.; Grishina, M.A. Technique for Energy Decomposition in the Study of “Receptor-Ligand” ComplexesEnergy Decomposition of “Receptor-Ligand” Complexes. J. Chem. Inf. Model. 2009, 49, 1389–1406, doi:10.1021/ci800405n.
